# Glucagon-Like Peptide 1 Receptor Agonists and 13 Obesity-Associated Cancers in Patients With Type 2 Diabetes

**DOI:** 10.1001/jamanetworkopen.2024.21305

**Published:** 2024-07-05

**Authors:** Lindsey Wang, Rong Xu, David C. Kaelber, Nathan A. Berger

**Affiliations:** 1 Center for Science, Health, and Society, Case Western Reserve University School of Medicine, Cleveland, Ohio; 2 Center for Artificial Intelligence in Drug Discovery, Case Western Reserve University School of Medicine, Cleveland, Ohio; 3 Case Comprehensive Cancer Center, Case Western Reserve University School of Medicine, Cleveland, Ohio; 4 Departments of Internal Medicine, Pediatrics, and Population and Quantitative Health Sciences and the Center for Clinical Informatics Research and Education, The MetroHealth System, Cleveland, Ohio

## Abstract

**Importance:**

Thirteen human malignant neoplasms have been identified as obesity-associated cancers (OACs), ie, the presence of excess body fat is associated with increased risk of developing cancer and worse prognosis in patients with these specific tumors. The glucagon-like peptide receptor agonist (GLP-1RA) class of pharmaceuticals are effective agents for the treatment of type 2 diabetes (T2D) and for achieving weight loss, but the association of GLP-1RAs with the incident risk of 13 OACs is unclear.

**Objective:**

To compare the incident risk of each of the 13 OACs in patients with T2D who were prescribed GLP-1RAs vs insulins or metformin.

**Design, Setting, and Participants:**

This retrospective cohort study was based on a nationwide multicenter database of electronic health records (EHRs) of 113 million US patients. The study population included 1 651 452 patients with T2D who had no prior diagnosis of OACs and were prescribed GLP-1RAs, insulins, or metformin during March 2005 to November 2018. Data analysis was conducted on April 26, 2024.

**Exposures:**

Prescription of GLP-1RAs, insulins, or metformin.

**Main Outcomes and Measures:**

Incident (first-time) diagnosis of each of the 13 OACs occurring during a 15-year follow-up after the exposure was examined using Cox proportional hazard and Kaplan-Meier survival analyses with censoring applied. Hazard ratios (HRs), cumulative incidences, and 95% CIs were calculated. All models were adjusted for confounders at baseline by propensity-score matching baseline covariates.

**Results:**

In the study population of 1 651 452 patients with T2D (mean [SD] age, 59.8 [15.1] years; 827 873 [50.1%] male and 775 687 [47.0%] female participants; 5780 [0.4%] American Indian or Alaska Native, 65 893 [4.0%] Asian, 281 242 [17.0%] Black, 13 707 [0.8%] Native Hawaiian or Other Pacific Islander, and 1 000 780 [60.6%] White participants), GLP-1RAs compared with insulin were associated with a significant risk reduction in 10 of 13 OACs, including in gallbladder cancer (HR, 0.35; 95% CI, 0.15-0.83), meningioma (HR, 0.37; 95% CI, 0.18-0.74), pancreatic cancer (HR, 0.41; 95% CI, 0.33-0.50), hepatocellular carcinoma (HR, 0.47; 95% CI, 0.36-0.61), ovarian cancer (HR, 0.52; 95% CI, 0.03-0.74), colorectal cancer (HR, 0.54; 95% CI, 0.46-0.64), multiple myeloma (HR, 0.59; 95% CI, 0.44-0.77), esophageal cancer (HR, 0.60; 95% CI, 0.42-0.86), endometrial cancer (HR, 0.74; 95% CI, 0.60-0.91), and kidney cancer (HR, 0.76; 95% CI, 0.64-0.91). Although not statistically significant, the HR for stomach cancer was less than 1 among patients who took GLP-1RAs compared with those who took insulin (HR, 0.73; 95% CI, 0.51-1.03). GLP-1RAs were not associated with a reduced risk of postmenopausal breast cancer or thyroid cancer. Of those cancers that showed a decreased risk among patients taking GLP-1RAs compared with those taking insulin, HRs for patients taking GLP-1RAs vs those taking metformin for colorectal and gallbladder cancer were less than 1, but the risk reduction was not statistically significant. Compared with metformin, GLP-1RAs were not associated with a decreased risk of any cancers, but were associated with an increased risk of kidney cancer (HR, 1.54; 95% CI, 1.27-1.87).

**Conclusions and Relevance:**

In this study, GLP-1RAs were associated with lower risks of specific types of OACs compared with insulins or metformin in patients with T2D. These findings provide preliminary evidence of the potential benefit of GLP-1RAs for cancer prevention in high-risk populations and support further preclinical and clinical studies for the prevention of certain OACs.

## Key Points

**Question:**

Is there clinical evidence supporting the potential benefits of glucagon-like peptide receptor agonists (GLP-1RAs) for the prevention of 13 obesity-associated cancers (OACs)?

**Findings:**

This cohort study of more than 1.6 million patients with type 2 diabetes (T2D) who had no prior diagnosis of 13 OACs found that patients with T2D treated with GLP-1RAs vs insulin had a significant risk reduction in 10 of 13 OACs, including esophageal, colorectal, endometrial, gallbladder, kidney, liver, ovarian, and pancreatic cancer as well as meningioma and multiple myeloma. No decrease in cancer risk was associated with GLP-1RAs compared with metformin.

**Meaning:**

This study provides clinical data suggesting that GLP-1RAs may reduce the risk of specific OACs compared with insulins.

## Teaser

This cohort study evaluates whether glucagon-like peptide receptor agonists (GLP-1RAs) are associated with risk of obesity-associated cancer among patients with type 2 diabetes compared with insulin or metformin.

## Introduction

Thirteen human malignant neoplasms have been identified as obesity-associated cancers (OAC), ie, the presence of excess body fat is associated with increased risk of developing cancer and worse prognosis in patients with these specific tumors.^1^ Obesity also contributes to insulin resistance and type 2 diabetes (T2D), which may further increase the risk and worsen the prognosis of the OACs.^2,3^

The glucagon-like peptide 1 receptor agonist (GLP-1RA) class of pharmaceuticals are highly effective agents for the treatment of T2D and for achieving weight loss.^4,5,6,7,8,9^ GLP-1RAs have further been shown to reduce the risk of adverse cardiovascular outcomes in patients with obesity^10^ and to contribute to the resolution of nonalcoholic steatohepatitis.^11^ Because of their efficacy in controlling T2D, obesity, and related comorbidities, we hypothesized that these agents might reduce the risk of the OACs. We recently reported that GLP-1RAs were associated with lower risks for colorectal cancer,^12^ an OAC. Otherwise, clinical evidence of the potential clinical benefits of GLP-1RA in preventing OAC has not been systematically assessed. Here we conducted a nationwide multicenter retrospective cohort study in patients with T2D who were prescribed GLP-1RAs vs insulins or metformin to determine whether GLP-1RAs were associated with changes in the risk of each of 13 OACs, including esophageal, breast, colorectal, endometrial, gallbladder, stomach, kidney, ovarian, pancreatic, and thyroid cancer as well as hepatocellular carcinoma, meningioma, and multiple myeloma.^1^

## Methods

### Database

We used the TriNetX platform to access deidentified electronic health records (EHRs) of 113 million patients from 64 health care organizations across 50 states, covering diverse age, racial and ethnic, income, and insurance groups and clinical settings.^13,14^ The platform’s built-in analytic functions allow patient-level analyses, while only reporting population-level data. The platform has been used for retrospective cohort studies.^15,16,17,18,19,20,21,22,23,24,25,26^ Similar to this study, we have examined the association of GLP-1RAs with colorectal cancer incidence in patients with T2D^12^ and the associations of GLP-1RA (semaglutide) with suicidal ideations^27^ and with cannabis use in patients with obesity and those with T2D.^28^ The MetroHealth System institutional review board determined that the research as described in this study was not human participant research and institutional review board approval and informed consent were not required. This cohort study followed the Strengthening the Reporting of Observational Studies in Epidemiology (STROBE) reporting guideline.

Available data elements of EHRs include extensive information on demographics, diagnoses (*International Statistical Classification of Diseases and Related Health Problems, Tenth Revision*), medications (Anatomical Therapeutic Chemical and medical prescription normalized medical prescription or RxNorm), procedures (*Current Procedural Terminology*), laboratory tests (Logical Observation Identifiers Names and Codes), genomics, visits, and socioeconomic and lifestyle information. The data on the analytic platform have been expanded to include oncology-specific data from cancer registry data from North American Association of Central Cancer Registries (NAACCR) records and other data resources.^14^

Self-reported sex, race, and ethnicity data from contributing health care systems are mapped by according to Office of Management and Budget standards into (1) race, American Indian or Alaska Native, Asian, Black or African American, Native Hawaiian or Other Pacific Islander, White, and unknown race; and (2) ethnicity, Hispanic or Latinx, not Hispanic or Latinx, or unknown ethnicity. All covariates are either binary, categorical, or continuous but essentially guaranteed to exist. Age is guaranteed to exist. Missing sex values are represented using “unknown sex.” The missing data for race and ethnicity are presented as “unknown race” or “unknown ethnicity.” For other variables, including medical conditions, procedures, laboratory tests, and socioeconomic determinants of health, the value is either present or absent so missing is not pertinent.

### Study Population

**Figure 1.**
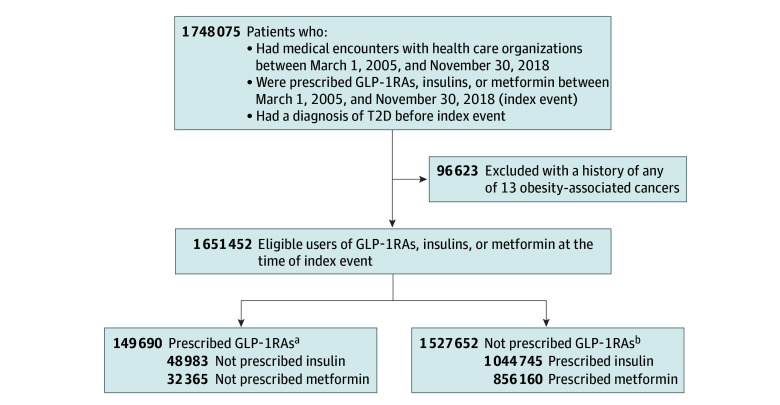
Study Group Selection Flow Diagram T2D indicates type 2 diabetes. ^a^The combined total of patients is not necessarily a sum of the individuals from each of the groups because individuals could be prescribed both glucagon-like peptide 1 receptor agonists (GLP-1RAs) and insulins or metformin during the study period. ^b^The combined total of patients is not necessarily a sum of the individuals from each of the groups because individuals could be prescribed both insulins and metformin during the study period.

The study population comprised 1 651 452 patients with a diagnosis of T2D who had medical encounters with health care organizations and were prescribed GLP-1RAs vs insulin or metformin between March 2005 and November 2018 and had no history of any of the 13 OACs. The study population was divided into exposure and comparison groups. For comparing GLP-1RAs with insulins, the study population was divided into a GLP-1RA/no insulin group (48 983 patients prescribed a GLP-1RA but not insulins) and a insulin/no GLP-1RA group (1 044 745 patients prescribed insulins but not GLP-1RAs). For comparing GLP-1RAs with metformin, the study population was divided into a GLP-1RA/no metformin group (32 365 patients prescribed a GLP-1RA but not metformin) and a metformin/no GLP-1RA group (856 160 patients prescribed metformin but not GLP-1RAs) (Figure 1).

### Statistical Analysis

The 13 OACs are esophageal, breast, colorectal, endometrial, gallbladder, stomach, kidney, ovarian, pancreatic, and thyroid cancer as well as hepatocellular carcinoma, meningioma, and multiple myeloma.^1^ Each of the 13 OACs was examined as a separate outcome in groups that were propensity-score matched for covariates related to the specific OAC. For each OAC outcome, the exposure and comparison groups (ie, GLP-1RA/no insulin vs insulin/no GLP-1RA groups and GLP-1RA/no metformin vs metformin/no GLP-1RA groups) were propensity-score matched (1:1 using nearest neighbor greedy matching) for baseline covariates related to the specific OAC, including demographic characteristics (age, sex, race, and ethnicity); adverse socioeconomic determinants of health; family and personal history of cancer; genetic susceptibility to cancer; preexisting medical conditions, including obesity and overweight; and medical procedures, including cancer screening, bariatric surgery, and prior prescription of antidiabetes medications. Each eligible individual was followed up from the index event (the first prescription of GLP-1RAs, insulins, or metformin during March 2005 to November 2018) until the occurrence of the outcomes, death, loss to follow-up, or 15 years after the index event, whichever occurred first. Cox proportional hazard analyses were used to compare rates of time to events on a daily basis during the follow-up time after the index event. Hazard ratios (HRs) and 95% CIs were calculated. Cumulative incidences were estimated using the Kaplan-Meier survival analysis. All models are adjusted for confounders at baseline by propensity-score matching baseline covariates.

The data were collected and analyzed on April 26, 2024, within the analytics platform. All statistical analyses in this study, including propensity-score matching, Kaplan-Meier survival analysis, and Cox proportional hazard analysis were done using built-in functions within the platform that are implemented using Survival version 3.2-3 in R version 4.0.2 (R Project for Statistical Computing) and libraries and utilities for data science and statistics in Python version 3.7 (Python Software Foundation) and Java version 11.0.16 (Oracle). Details of clinical codes for eligibility criteria, treatment strategies, outcomes, and baseline covariates are in eTable 1 in Supplement 1.

## Results

### Associations of GLP-1RAs With 13 OACs in Patients With T2D Compared With Insulins

The study population included 1 651 452 patients with T2D (mean [SD] age, 59.8 [15.1] years; 827 873 [50.1%] male and 775 687 [47.0%] female participants; 5780 [0.4%] American Indian or Alaska Native, 65 893 [4.0%] Asian, 281 242 [17.0%] Black, 13 707 [0.8%] Native Hawaiian or Other Pacific Islander, and 1 000 780 [60.6%] White participants). For comparing GLP-1RAs with insulins in patients with T2D, the study population included 1 093 728 patients with T2D who had no prior diagnosis of any OAC and were prescribed GLP-1RAs or insulins but not both between March 2005 and November 2018. The GLP-1RA/no insulin group (n = 48 983) compared with the insulin/no GLP-1RA group (n = 1 044 475) was younger; included more women and White participants; had a higher prevalence of family history of cancer, obesity or overweight, medical encounters for cancer screening, and prior prescriptions of other antidiabetic agents, including insulins, metformin, dipeptidyl peptidase 4 (DPP-4) inhibitors, sodium-glucose cotransporter 2 (SGLT2) inhibitors, sulfonylureas, thiazolidinediones, and α-glucosidase inhibitors. For each OAC outcome, the GLP-1RA/no insulin and the insulin/no GLP-1RA groups were separately matched for covariates associated with the OAC. The Table shows the characteristics of the GLP-1RA/no insulin and insulin/no GLP-1RA groups before and after propensity-score matching for covariates related to colorectal cancer. The characteristics of the exposure and comparison groups before and after matching for each of the other 12 OACs are in eTables 2 to 13 in Supplement 1.

**Table.** Characteristics of the GLP-1RA/No Insulin Group and the Insulin/No GLP-1RA Group Before and After Propensity Score Matching for Baseline Covariates Related to Colorectal Cancer

Characteristic^a^	Before propensity-score matching				After propensity-score matching			
	GLP-1RA/no insulin, No. (%) (n = 48 983)	Insulin/no GLP-1RA, No. (%) (n = 1 044 745)	SMD	GLP-1RA/no insulin, No. (%) (n = 48 443)		Insulin/no GLP-1RA, No. (%) (n = 48 443)	SMD
Age at index event, mean (SD), y	55.9 (11.7)	61.5 (15.9)	0.42^b^	55.9 (11.7)		56.2 (13.4)	0.02
Sex							
Female	26 011 (53.1)	476 110 (45.6)	0.15^b^	53.4		54.1	0.02
Male	20 720 (42.3)	541 314 (51.8)	0.19^b^	42.4		41.8	0.01
Unknown	2252 (4.6)	27 321 (2.6)	0.11^b^	4.5		4.1	0.02
Ethnicity							
Hispanic or Latinx	4151 (8.5)	94 136 (9.0)	0.02	8.5		8.3	0.007
Not Hispanic or Latinx	33 188 (67.8)	659 375 (63.1)	0.09	67.8		68.5	0.02
Unknown	11 644 (23.8)	291 234 (27.9)	0.09	23.7		23.2	0.01
Race							
American Indian or Alaska Native	199 (0.4)	3443 (0.3)	0.01	0.4		0.4	0.003
Asian	1204 (2.5)	41 822 (4.0)	0.09	2.5		2.2	0.02
Black or African American	6265 (12.8)	178 267 (17.1)	0.12^b^	12.8		12.2	0.02
Native Hawaiian or Other Pacific Islander	205 (0.4)	10 677 (1.0)	0.07	0.4		0.3	0.03
White	32 592 (66.5)	633 989 (60.7)	0.12^b^	66.6		68.7	0.05
Unknown	7099 (14.5)	142 470 (13.6)	0.03	14.4		13.5	0.03
Adverse socioeconomic determinants of health	686 (1.4)	12 021 (1.2)	0.02	1.4		1.1	0.03
Family history of cancer	2042 (4.2)	19 398 (1.9)	0.14^b^	4.1		3.8	0.02
Family history of cancer of digestive organs	784 (1.6)	6889 (0.7)	0.09	1.6		1.4	0.01
Family history of colonic polyps	149 (0.3)	579 (0.1)	0.06	0.3		0.3	0.006
Genetic susceptibility to cancer	24 (0.0)	156 (0.0)	0.02	0.0		0.0	0.003
Personal history of cancer	1239 (2.5)	38 294 (3.7)	0.07	2.5		2.1	0.03
Preexisting medical conditions, procedures, and medications							
Obesity or overweight	18 401 (37.6)	166 445 (15.9)	0.50^b^	37.1		37.1	<.001
Obesity due to excess calories	9157 (18.7)	69 998 (6.7)	0.37^b^	18.4		18.3	0.003
Obesity, unspecified	13 805 (28.2)	118 555 (11.3)	0.43^b^	27.8		28.2	0.009
Morbid (severe) obesity with alveolar hypoventilation	150 (0.3)	3643 (0.3)	0.007	0.3		0.2	0.02
BMI 30.0-39.0, adult	4204 (8.6)	43 128 (4.1)	0.18^b^	8.3		7.5	0.03
BMI ≥40, adult	3886 (7.9)	33 396 (3.2)	0.21^b^	7.8		7.1	0.03
Overweight defined by *ICD-10* code E66.3	1010 (2.1)	6796 (0.7)	0.12^b^	2.0		1.8	0.02
BMI 25.0-25.9, adult	157 (0.3)	3510 (0.3)	<.001	0.3		0.3	0.01
BMI 26.0-26.9, adult	171 (0.3)	3632 (0.3)	<.001	0.4		0.3	0.007
BMI 27.0-27.9, adult	288 (0.6)	4209 (0.4)	0.03	0.6		0.5	0.008
BMI 28.0-28.9, adult	344 (0.7)	4508 (0.4)	0.04	0.7		0.6	0.02
BMI 29.0-29.9, adult	398 (0.8)	4843 (0.5)	0.04	0.8		0.8	0.002
Alcohol use disorder	563 (1.1)	28 538 (2.7)	0.12^b^	1.2		0.8	0.03
Nicotine dependence	3593 (7.3)	96 860 (9.3)	0.07	7.4		6.4	0.04
Crohn disease	130 (0.3)	3290 (0.3)	0.009	0.3		0.2	0.009
Ulcerative colitis	165 (0.3)	3393 (0.3)	0.002	0.3		0.3	0.004
Cystic fibrosis	<10 (<0.1)	1411 (0.1)	0.04	0.0		0.0	0.004
Colon polyps	2634 (5.4)	31 528 (3.0)	0.12^b^	5.3		4.8	0.02
Benign neoplasm of colon and rectum	3124 (6.4)	37 914 (3.6)	0.13^b^	6.3		5.6	0.03
Encounter for cancer screening	12 272 (25.1)	105 217 (10.1)	0.40^b^	24.6		23.8	0.02
Colonoscopy	3806 (7.8)	38 112 (3.6)	0.18^b^	7.7		6.9	0.03
Bariatric surgery	632 (1.3)	5136 (0.5)	0.09	1.3		1.2	0.003
Metformin	27 075 (55.3)	199 802 (19.1)	0.81^b^	54.8		55.5	0.01
Dipeptidyl peptidase 4 inhibitors	9485 (19.4)	44 595 (4.3)	0.48^b^	18.7		18.9	0.005
Sodium-glucose cotransporter 2 inhibitors	4808 (9.8)	6447 (0.6)	0.42^b^	8.9		7.8	0.04
Sulfonylureas	14 077 (28.7)	125 703 (12.0)	0.42^b^	28.4		29.3	0.02
Thiazolidinediones	4107 (8.4)	35 435 (3.4)	0.21^b^	8.3		8.8	0.02
α-Glucosidase inhibitors	229 (0.5)	1718 (0.2)	0.05	0.5		0.5	0.002
Other blood glucose lowering drugs	614 (1.3)	7048 (0.7)	0.06	1.2		1.2	0.009

Abbreviations: BMI, body mass index (calculated as weight in kilograms divided by height in meters squared); GLP-1RA, glucagon-like peptide 1 receptor agonist; *ICD-10*, *International Statistical Classification of Diseases and Related Health Problems, Tenth Revision*; SMD, standardized mean difference.

^a^ Groups before and after propensity-score matching for risk factors for colorectal cancer are shown. The status of matched variables was based on the presence of related codes anytime to 1 day before the index event (the first prescription of a GLP-1RA or insulin in March 2005 to November 2018). Adverse socioeconomic determinants of health (codes Z55-Z65) include problems related to education and literacy, employment and unemployment, housing and economic circumstances, social environment, upbringing, primary support group including family circumstances, certain psychosocial circumstances, and other psychosocial circumstances. Problems with lifestyle included tobacco use, lack of physical exercise, inappropriate diet and eating habits, high-risk sexual behavior, gambling and betting, and other problems related to lifestyle including antisocial behavior and sleep deprivation.

^b^ SMD greater than 0.10, indicating group imbalance.

**Figure 2.**
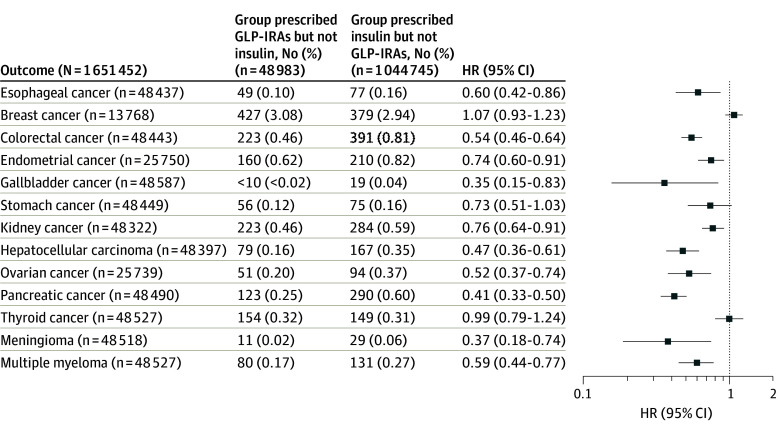
Risk of 13 Obesity-Associated Cancers Among Patients Receiving Glucagon-Like Peptide 1 Receptor Agonists (GLP-1RAs) vs Those Receiving Insulins Patients were followed up for as along as 15 years after the index event for both groups. Hazard ratios (HRs) rates were calculated using a Cox proportional hazards model with censoring applied. Overall risk equals the number of patients with outcomes during the follow-up time window divided by number of patients in the group at the beginning of the time window. For each outcome, the groups were separately propensity-score matched for covariates related to the outcome, and the outcome was compared between the matched groups. Each eligible individual was followed up from the index event until the occurrence of the outcomes, death, loss to follow-up, or 15 years after the index event, whichever occurred first.

**Figure 3.**
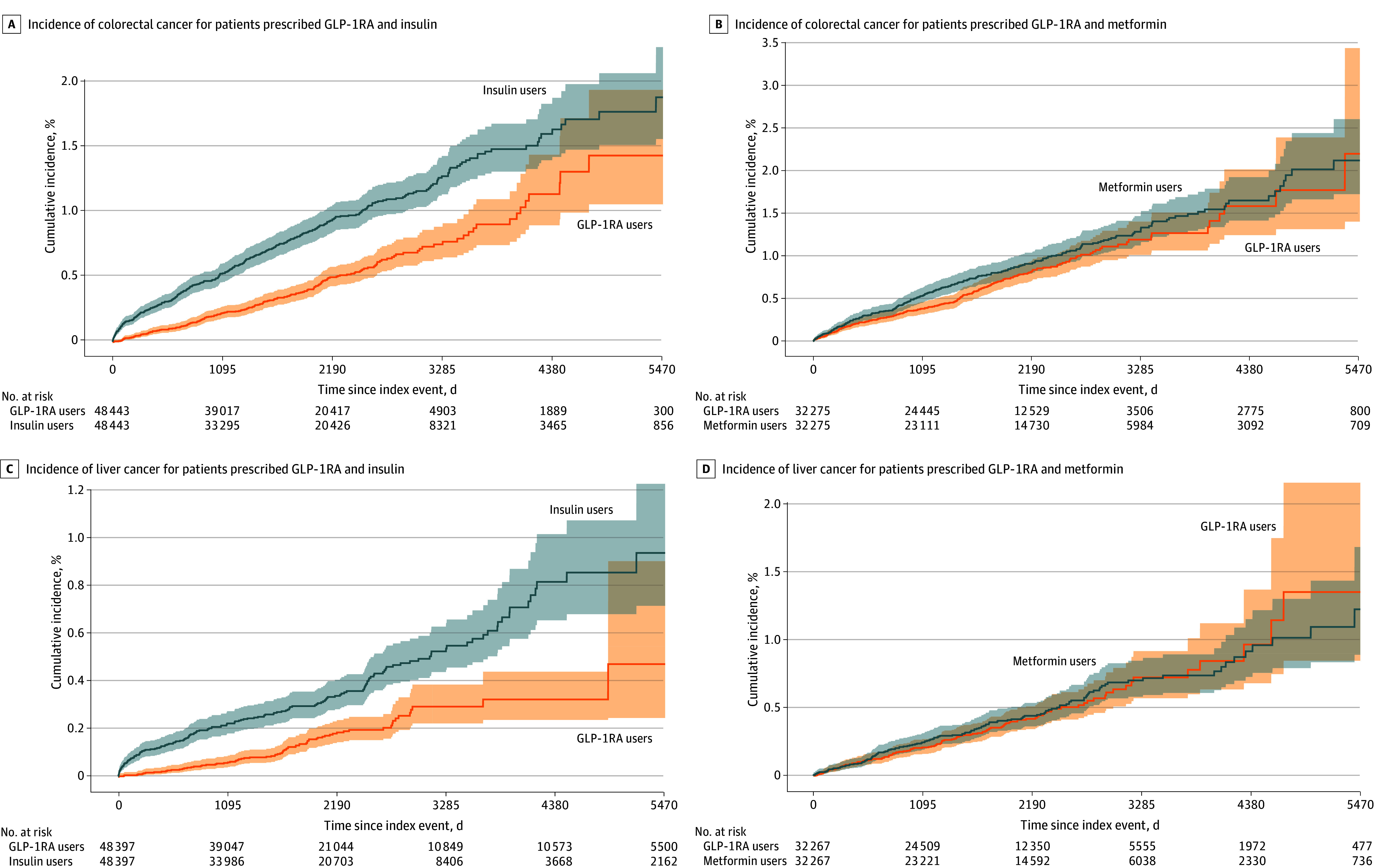
Cumulative Incidences of Colorectal Cancer and Liver Cancer Among Patients Receiving Glucagon-Like Peptide 1 Receptor Agonists (GLP-1RAs) vs Those Receiving Insulins or Metformin During a 15-Year Follow-Up Kaplan-Meier survival analysis was used. Each eligible individual was followed up from the index event until the occurrence of the outcomes, death, loss to follow-up, or 15 years after the index event, whichever occurred first.

Compared with insulins, GLP-1RAs were associated with a significantly lower risk of 10 of the 13 OACs, including gallbladder cancer (HR, 0.35; 95% CI, 0.15-0.83), meningioma (HR, 0.37; 95% CI, 0.18-0.74), pancreatic cancer (HR, 0.41; 95% CI, 0.33-0.50), hepatocellular carcinoma (HR, 0.47; 95% CI, 0.36-0.61), ovarian cancer (HR, 0.52; 95% CI, 0.03-0.74), colorectal cancer (HR, 0.54; 95% CI, 0.46-0.64), multiple myeloma (HR, 0.59; 95% CI, 0.44-0.77), esophageal cancer (HR, 0.60; 95% CI, 0.42-0.86), endometrial cancer (HR, 0.74; 95% CI, 0.60-0.91), and kidney cancer (HR, 0.76; 95% CI, 0.64-0.91). The HR for stomach cancer among patients taking GLP-1RAs vs those taking insulin was less than 1, but it was not statistically significant (HR, 0.73; 95% CI, 0.51-1.03). GLP-1RAs were not associated with risk of postmenopausal breast cancer or thyroid cancer (Figure 2). Figure 3 shows the cumulative incidences of colorectal cancer and liver cancer comparing GLP-1RAs with insulins. The mean (SD) follow-up time for the outcome of colorectal cancer was 2074.7 (435.3) days for the GLP-1RA/no insulin group and 1981.8 (471.1) days for the insulin/no GLP-1RA group. The mean (SD) follow-up time for the outcome of liver cancer was 2023.1 (1112.6) days for the GLP-1RA/no insulin group and 2037.9 (766.4) days for the insulin/no GLP-1RA group.

### Associations of GLP-1RAs With 13 OACs in Patients With T2D Compared With Metformin

**Figure 4.**
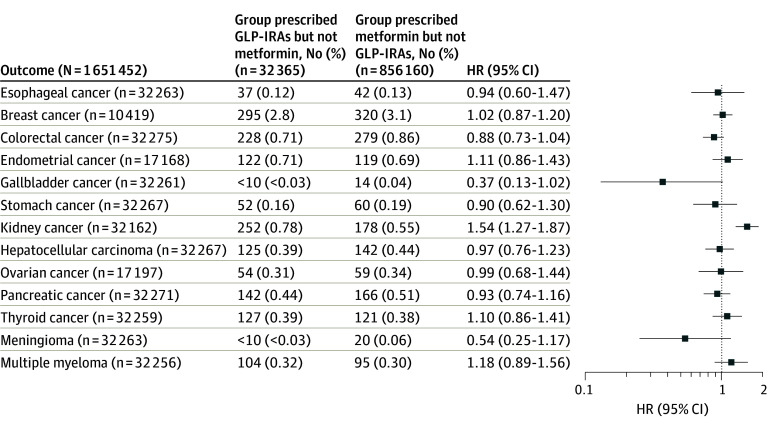
Risk of 13 Obesity-Associated Cancers Among Patients Receiving Glucagon-Like Peptide 1 Receptor Agonists (GLP-1RAs) vs Those Receiving Metformin Patients were followed up for as long as 15 years after the index event for both groups. Hazard ratios (HRs) were calculated using a Cox proportional hazards model with censoring applied. Overall risk equals the number of patients with outcomes during the follow-up time window divided by the number of patients in the group at the beginning of the time window. For each outcome, the groups were separately propensity-score matched for covariates related to the outcome, and the outcome was compared between the matched groups. Each eligible individual was followed up from the index event until the occurrence of the outcomes, death, loss to follow-up, or 15 years after the index event, whichever occurred first.

For comparing GLP-1RAs with metformin in patients with T2D, the study population included 888 525 patients with T2D who had no prior diagnosis of any OAC and were prescribed GLP-1RAs or metformin but not both between March 2005 and November 2018. For each OAC outcome, the GLP-1RA/no metformin group (n = 32 365) and the metformin/no GLP-1RA group (n = 856 160) were separately matched for covariates related to the OAC (eTables 14-26 in Supplement 1). Compared with metformin, GLP-1RAs were not associated with a lower risk of colorectal cancer, gallbladder cancer, and meningioma but were associated with an increased risk of kidney cancer (Figure 4). Figure 3 shows the cumulative incidences of colorectal cancer and liver cancer by comparing GLP-1RAs with metformin. The mean (SD) follow-up time for the outcome of colorectal cancer was 1967.2 (592.2) days for the GLP-1RA/no metformin group and 2101.6 (576.0) days for metformin/no GLP-1RA group. The mean (SD) follow-up time for the outcome of liver cancer was 1970.9 (426.0) days for the GLP-1RA/no metformin group and 2129.8 (514.7) days for metformin/no GLP-1RA group.

## Discussion

Using a data platform^29^ to analyze more than 15 years of longitudinal EHRs of a US population-based cohort of more than 100 million individuals, we found that in patients with T2D who had no history of any OAC, GLP-1RAs compared with insulins were associated with a significant risk reduction in 10 of 13 OACs, including esophageal, colorectal, kidney, pancreatic, gallbladder, ovarian, endometrial, and liver cancers as well as meningioma and multiple myeloma. Decreased risk reduction that did not reach statistical significance was also noted for stomach cancer. Of those cancers that showed decreased risk of GLP-1RAs compared with insulin, risk reduction was also noted for GLP-1RAs relative to metformin for colorectal cancer, gallbladder, and meningiomas, although these findings were not statistically significant.

Our observations on the reduction in the incidence of OACs in patients with T2D treated with GLP-1RAs compare favorably with the OAC-reducing effects of intensive lifestyle intervention (ILI) observed in the Look AHEAD trial (Action for Health in Diabetes)^30^ and with the results of metabolic-bariatric surgery as recently reported in the SPLENDID (Surgical Procedure and Long-term Effectiveness In Neoplastic Disease Incidence and Death) trial.^31^ The Look AHEAD study, a randomized clinical trial in which 4859 patients with T2D and overweight or obesity (age, 45-76 years; median follow-up, 11 years) were randomized to an ILI or diabetes support and education group, found a 16% reduction in risk for OAC (HR, 0.84; 95% CI, 0.68-1.04).^30^ The SPLENDID trial, a matched cohort study, compared 5053 patients with obesity with 25 265 nonsurgical matched controls, with a median age of 46 years and median follow-up of 6.1 years, showed an OAC risk reduction of 32%, (HR, 0.68; 95% CI, 0.53-0.87).^31^

A recent 9-year follow-up population-based historical cohort study^32^ conducted in Israel reported a decrease (although not statistically significant) in incidence of pancreatic cancer (HR, 0.50; 95% CI, 0.15-1.71) in patients with T2D treated with GLP-1RAs compared with insulin.^32^ Our US population-based study, with 15 years of follow-up and a larger sample size, now extends these observations, suggesting that treatment of patients with T2D with GLP-1RAs vs insulin is associated with a significantly decreased incidence of pancreatic cancer (HR, 0.41; 95% CI, 0.33-0.50).

In contrast to the risk reduction shown for most of the OACs, thyroid cancer showed no statistically different risk in patients treated with GLP-1RAs compared with insulins. Studies in rodents indicate that GLP-1RAs promote thyroid C-cell hyperplasia and medullary thyroid carcinoma (MTC) by a GLP-1R mediated increase in calcitonin synthesis.^33^ High levels of fasting serum insulin and insulin resistance are associated with an increased risk of thyroid cancer.^34^ Although clinical evidence for an association of thyroid cancer with the use of GLP-1RAs has been reported as inconclusive,^35^ the findings from our study together with previous reports of insulins promoting cancer growth suggest that GLP-1RAs might be associated with increased risk of thyroid cancer. Our results are further supported by a recent report^36^ by the French National Health Cancer Data System showing that the use of GLP-1RAs for 1 to 3 years was associated with increased risk of all thyroid cancers (adjusted HR, 1.78; 95% CI 1.04-3.05).^36^ These studies support the package warnings included with GLP-1RAs that these agents are contraindicated in patients with multiple endocrine neoplasia syndrome type 2 and that patients should be counseled regarding the potential risk of MTC and symptoms of thyroid tumors.

Kidney cancers showed an increased risk with GLP-1RA treatment relative to that with metformin (HR, 1.54; 95% CI 1.27-1.87) but a decrease relative to insulin (HR, 0.76; 95% CI 0.64-0.91). GLP-1RAs have direct effects on kidney function mediated by GLP-1Rs in renal vasculature; however, these are not associated with increased mitogenesis,^37^ and to our knowledge, there have been no previous reports of kidney cancers with the use of GLP1-RAs. These divergent risks require further clinical and mechanistic studies for full evaluation. Nonetheless, they suggest the need for continued monitoring in patients being treated with GLP-1RAs.

Our study, with follow-up over 15 years, found no signs of increase or decrease in risk for breast cancer in postmenopausal women with T2D being treated with GLP-1RAs compared with those being treated with insulin or metformin. GLP-1RAs have been shown to reduce the growth of murine and human breast cancer cell lines in vitro and in vivo murine models.^38^ However, a meta-analysis of more than 50 randomized clinical trials, evaluating GLP-1RAs in women aged between 45 to 70 years and followed up from 24 weeks to 7.5 years, showed no differences in benign, premalignant, or malignant breast neoplasms in patients treated with GLP-1RAs compared with other antidiabetic agents or placebos.^39^ A more recent population-based cohort study of 44 984 women 40 years and older treated with GLP-1RAs or other antidiabetic agents for a mean of 3.5 years showed no overall significant difference in the risk for breast cancer occurrence. However, an increased risk (HR, 2.66; 95% CI, 1.32-5.38) was noted for those treated between 2 to 3 years with a return to null after more than 3 years’ treatment.^40^ Interestingly, the SPLENDID trial of bariatric surgery for weight reduction, which found an overall 32% risk reduction for OACs, showed no significant difference among women for incidence of overall or postmenopausal breast cancer.^31^ This lack of effect on breast cancer risk needs to be further investigated to determine the impact of longer duration of therapy as well as to more fully understand the relation between GLP-1RAs and estrogen metabolism. The lack of breast cancer risk reduction by GLP-1RAs and the similar lack of protection by bariatric surgery may also suggest the possibility that factors determining the incidence of breast cancer in patients with overweight or obesity may have been initiated long before intervention with GLP-1RAs and/or bariatric surgery and therefore require earlier intervention to affect risk reduction. The concept that early intervention might reduce breast cancer incidence is supported by the observation that both pregnancy and breastfeeding reduce the incidence of breast cancer.^41,42^

### Limitations

Our study has several limitations. First, this is a retrospective observational study of patient EHRs, which has inherent limitations including overdiagnosis, underdiagnosis, and misdiagnosis; unmeasured or uncontrolled confounders; and biases. Although we controlled for an extensive list of variables, these limitations and biases could not be fully eliminated; therefore, no causal inferences can be drawn. Second, patients in our study represented those who had medical encounters with health care systems contributing to the data platform. Although both the exposure and comparison groups were drawn from the same EHR database and from the same time period, which should not significantly affect the HR calculations, results from the platform need to be validated in other EHR databases and analytics platforms. Third, the status of incident cancer was based on the presence of first-ever diagnosis codes of OACs documented in patient EHRs, which also included oncology-specific data from cancer registry data, such as NAACCR records. However, it is unknown how well cancer diagnoses are captured in patient EHRs. For this study, the main interest was the relative risk (or HR) of cancer diagnosis. Since all patients in the study population were drawn from the same health care organizations in the data platform, cancer underdiagnosis, misdiagnosis, or overdiagnosis should not have a substantial impact on the relative risk analysis. Fourth, the built-in functions did not allow us to control for variables (eg, weight loss) that occurred after the index event and to identify individual patient data, which precludes our ability to correlate risk reduction with a degree of weight loss, which was demonstrated to be particularly important in the SPLENDID bariatric study.^25^ In addition, we could not explicitly control for health care utilization and insurance type although the study population included patients who had medical encounters with health care organizations and were withdrawn from the same 64 health care organizations in the network. Finally, due to the lack of patients’ medication adherence information in EHRs, we used intention-to-treat (medication prescriptions) as a causal contrast of interest regardless of whether the individuals adhered to their medications and the duration of the medication use.

## Conclusions

In this study of patients with T2D who were cancer free at baseline, taking GLP-1RAs compared with insulin was associated with a lower risk of 10 of 13 OACs. The potential cancer-preventative effects of OACs by GLP-1RAs warrant further long-term studies as well as studies of individual newer and possibly more effective antidiabetic and weight loss agents as well as those with multihormone agonist activities. Studies are also warranted to evaluate the preventive effects of these agents on non-OACs. In addition, the associations of the GLP-1RA targeted pharmacologic agents with cancer risk should be compared with the use of ILI and metabolic-bariatric surgery for the control of obesity and diabetes. As noted previously, it will be important to correlate these associations with the control of T2D and obesity. Moreover, given that T2D and overweight or obesity have negative impacts on patients during cancer therapy, GLP-1RAs should be evaluated for control of these comorbid conditions during cancer therapy as well as for secondary prevention to delay cancer recurrence.

## Notes

Supplement 1. **eAppendix.** Database **eTable 1.** Clinical diagnosis, and other codes used in the platform that are used to determine the status of variables for study population definitions, exposures, outcomes, and those for propensity-score matching for groups **eTable 2.** Characteristics of the GLP-1RA/no insulin group and insulin/no GLP-1RA group before and after matched for baseline covariates related to esophageal cancer for the study populations of patients with T2D and no history of any OAC **eTable 3.** Characteristics of the GLP-1RA/no insulin group and insulin/no GLP-1RA group before and after matched for covariates related to breast cancer for the study populations of women (age 55 and older) with T2D and no history of any OAC **eTable 4.** Characteristics of the GLP-1RA/no insulin group and insulin/no GLP-1RA group before and after matched for covariates related to endometrial cancer for the study populations of women with T2D and no history of any OAC **eTable 5.** Characteristics of the GLP-1RA/no insulin group and insulin/no GLP-1RA group before and after matched for covariates related to gallbladder cancer for the study populations of patients with T2D and no history of any OAC **eTable 6.** Characteristics of the GLP-1RA/no insulin group and insulin/no GLP-1RA group before and after matched for covariates related to stomach cancer for the study populations of patients with T2D and no history of any OAC **eTable 7.** Characteristics of the GLP-1RA/no insulin group and insulin/no GLP-1RA group before and after matched for covariates related to kidney cancer for the study populations of patients with T2D and no history of any OAC **eTable 8.** Characteristics of the GLP-1RA/no insulin group and insulin/no GLP-1RA group before and after matched for covariates related to liver cancer for the study populations of patients with T2D and no history of any OAC **eTable 9.** Characteristics of the GLP-1RA/no insulin group and insulin/no GLP-1RA group before and after matched for covariates related to ovarian cancer for the study populations of women with T2D and no history of any OAC **eTable 10.** Characteristics of the GLP-1RA/no insulin group and insulin/no GLP-1RA group before and after matched for covariates related to pancreatic cancer for the study populations of patients with T2D and no history of any OAC **eTable 11.** Characteristics of the GLP-1RA/no insulin group and insulin/no GLP-1RA group before and after matched for covariates related to thyroid cancer for the study populations of patients with T2D and no history of any OAC **eTable 12.** Characteristics of the GLP-1RA/no insulin group and insulin/no GLP-1RA group before and after matched for covariates related to meningioma for the study populations of patients with T2D and no history of any OAC **eTable 13.** Characteristics of the GLP-1RA/no insulin group and insulin/no GLP-1RA group before and after matched for covariates related to multiple myeloma for the study populations of patients with T2D and no history of any OAC **eTable 14.** Characteristics of the GLP-1RA/no metformin group and metformin/no GLP-1RA group before and after matched for covariates related to esophageal cancer for the study populations of patients with T2D and no history of any OAC **eTable 15.** Characteristics of the GLP-1RA/no metformin group and metformin/no GLP-1RA group before and after matched for covariates related to breast cancer for the study populations of women (age 55 and older) with T2D and no history of any OAC **eTable 16.** Characteristics of the GLP-1RA/no metformin group and metformin/no GLP-1RA group before and after matched for covariates related to colorectal cancer for the study populations of patients with T2D and no history of any OAC **eTable 17.** Characteristics of the GLP-1RA/no metformin group and metformin/no GLP-1RA group before and after matched for covariates related to endometrial cancer for the study populations of women with T2D and no history of any OAC **eTable 18.** Characteristics of the GLP-1RA/no metformin group and metformin/no GLP-1RA group before and after matched for covariates related to gallbladder cancer for the study populations of patients with T2D and no history of any OAC **eTable 19.** Characteristics of the GLP-1RA/no metformin group and metformin/no GLP-1RA group before and after matched for covariates related to stomach cancer for the study populations of patients with T2D and no history of any OAC **eTable 20.** Characteristics of the GLP-1RA/no metformin group and metformin/no GLP-1RA group before and after matched for covariates related to kidney cancer for the study populations of patients with T2D and no history of any OAC **eTable 21.** Characteristics of the GLP-1RA/no metformin group and metformin/no GLP-1RA group before and after matched for covariates related to liver cancer for the study populations of patients with T2D and no history of any OAC **eTable 22.** Characteristics of the GLP-1RA/no metformin group and metformin/no GLP-1RA group before and after matched for covariates related to ovarian cancer for the study populations of women with T2D and no history of any OAC **eTable 23.** Characteristics of the GLP-1RA/no metformin group and metformin/no GLP-1RA group before and after matched for covariates related to pancreatic cancer for the study populations of patients with T2D and no history of any OAC **eTable 24.** Characteristics of the GLP-1RA/no metformin group and metformin/no GLP-1RA group before and after matched for covariates related to thyroid cancer for the study populations of patients with T2D and no history of any OAC **eTable 25.** Characteristics of the GLP-1RA/no metformin group and metformin/no GLP-1RA group before and after matched for covariates related to meningioma for the study populations of patients with T2D and no history of any OAC **eTable 26.** Characteristics of the GLP-1RA/no metformin group and metformin/no GLP-1RA group before and after matched for covariates related to multiple myeloma for the study populations of patients with T2D and no history of any OAC

Supplement 2.
Data Sharing Statement
