# Error in Figure

**DOI:** 10.1001/jamanetworkopen.2025.19224

**Published:** 2025-06-04

In the Original Investigation titled “Glucagon-Like Peptide 1 Receptor Agonists and 13 Obesity-Associated Cancers in Patients With Type 2 Diabetes,”^1^ published July 5, 2024, there was an error in Figure 3. Panel D should have been labeled “Incidence of liver cancer for patients prescribed GLP-1RA and metformin.” This article has been corrected.^1^
