# Supplementary material for: Glucagon-Like Peptide 1 Receptor Agonists and 13 Obesity-Associated Cancers in Patients With Type 2 Diabetes
Source: JAMA Netw Open. 2024 Jul 5;7(7):e2421305. doi: 10.1001/jamanetworkopen.2024.21305 (PMC11227080; doi:10.1001/jamanetworkopen.2024.21305)
Supplement: Supplement 1. — eAppendix. Database eTable 1. Clinical diagnosis, and other codes used in the platform that are used to determine the status of variables for study population definitions, exposures, outcomes, and those for propensity-score matching for groups eTable 2. Characteristics of the GLP-1RA/no insulin group and insulin/no GLP-1RA group before and after matched for baseline covariates related to esophageal cancer for the study populations of patients with T2D and no history of any OAC eTable 3. Characteristics of the GLP-1RA/no insulin group and insulin/no GLP-1RA group before and after matched for covariates related to breast cancer for the study populations of women (age 55 and older) with T2D and no history of any OAC eTable 4. Characteristics of the GLP-1RA/no insulin group and insulin/no GLP-1RA group before and after matched for covariates related to endometrial cancer for the study populations of women with T2D and no history of any OAC eTable 5. Characteristics of the GLP-1RA/no insulin group and insulin/no GLP-1RA group before and after matched for covariates related to gallbladder cancer for the study populations of patients with T2D and no history of any OAC eTable 6. Characteristics of the GLP-1RA/no insulin group and insulin/no GLP-1RA group before and after matched for covariates related to stomach cancer for the study populations of patients with T2D and no history of any OAC eTable 7. Characteristics of the GLP-1RA/no insulin group and insulin/no GLP-1RA group before and after matched for covariates related to kidney cancer for the study populations of patients with T2D and no history of any OAC eTable 8. Characteristics of the GLP-1RA/no insulin group and insulin/no GLP-1RA group before and after matched for covariates related to liver cancer for the study populations of patients with T2D and no history of any OAC eTable 9. Characteristics of the GLP-1RA/no insulin group and insulin/no GLP-1RA group before and after matched for covariates related to ovarian [file jamanetwopen-e2421305-s001.pdf]

Supplemental Online Content

Wang L, Xu R, Kaelber DC, Berger NA. Glucagon-like peptide 1 receptor agonists and 13 obesity-associated cancers in patients with type 2 diabetes. *JAMA Netw Open.* 2024;7(7):e2421305. doi:10.1001/jamanetworkopen.2024.21305

eAppendix. Database

eTable 1. Clinical diagnosis, and other codes used in the platform that are used to determine the status of variables for study population definitions, exposures, outcomes, and those for propensity-score matching for groups

eTable 2. Characteristics of the GLP-1RA/no insulin group and insulin/no GLP-1RA group before and after matched for baseline covariates related to esophageal cancer for the study populations of patients with T2D and no history of any OAC

eTable 3. Characteristics of the GLP-1RA/no insulin group and insulin/no GLP-1RA group before and after matched for covariates related to breast cancer for the study populations of women (age 55 and older) with T2D and no history of any OAC

eTable 4. Characteristics of the GLP-1RA/no insulin group and insulin/no GLP-1RA group before and after matched for covariates related to endometrial cancer for the study populations of women with T2D and no history of any OAC

eTable 5. Characteristics of the GLP-1RA/no insulin group and insulin/no GLP-1RA group before and after matched for covariates related to gallbladder cancer for the study populations of patients with T2D and no history of any OAC

eTable 6. Characteristics of the GLP-1RA/no insulin group and insulin/no GLP-1RA group before and after matched for covariates related to stomach cancer for the study populations of patients with T2D and no history of any OAC

eTable 7. Characteristics of the GLP-1RA/no insulin group and insulin/no GLP-1RA group before and after matched for covariates related to kidney cancer for the study populations of patients with T2D and no history of any OAC

eTable 8. Characteristics of the GLP-1RA/no insulin group and insulin/no GLP-1RA group before and after matched for covariates related to liver cancer for the study populations of patients with T2D and no history of any OAC

eTable 9. Characteristics of the GLP-1RA/no insulin group and insulin/no GLP-1RA group before and after matched for covariates related to ovarian cancer for the study populations of women with T2D and no history of any OAC

eTable 10. Characteristics of the GLP-1RA/no insulin group and insulin/no GLP-1RA group before and after matched for covariates related to pancreatic cancer for the study populations of patients with T2D and no history of any OAC

© 2024 Wang L et al. *JAMA Network Open.*

eTable 11. Characteristics of the GLP-1RA/no insulin group and insulin/no GLP-1RA group before and after matched for covariates related to thyroid cancer for the study populations of patients with T2D and no history of any OAC

eTable 12. Characteristics of the GLP-1RA/no insulin group and insulin/no GLP-1RA group before and after matched for covariates related to meningioma for the study populations of patients with T2D and no history of any OAC

eTable 13. Characteristics of the GLP-1RA/no insulin group and insulin/no GLP-1RA group before and after matched for covariates related to multiple myeloma for the study populations of patients with T2D and no history of any OAC

eTable 14. Characteristics of the GLP-1RA/no metformin group and metformin/no GLP-1RA group before and after matched for covariates related to esophageal cancer for the study populations of patients with T2D and no history of any OAC

eTable 15. Characteristics of the GLP-1RA/no metformin group and metformin/no GLP-1RA group before and after matched for covariates related to breast cancer for the study populations of women (age 55 and older) with T2D and no history of any OAC

eTable 16. Characteristics of the GLP-1RA/no metformin group and metformin/no GLP-1RA group before and after matched for covariates related to colorectal cancer for the study populations of patients with T2D and no history of any OAC

eTable 17. Characteristics of the GLP-1RA/no metformin group and metformin/no GLP-1RA group before and after matched for covariates related to endometrial cancer for the study populations of women with T2D and no history of any OAC

eTable 18. Characteristics of the GLP-1RA/no metformin group and metformin/no GLP-1RA group before and after matched for covariates related to gallbladder cancer for the study populations of patients with T2D and no history of any OAC

eTable 19. Characteristics of the GLP-1RA/no metformin group and metformin/no GLP-1RA group before and after matched for covariates related to stomach cancer for the study populations of patients with T2D and no history of any OAC

eTable 20. Characteristics of the GLP-1RA/no metformin group and metformin/no GLP-1RA group before and after matched for covariates related to kidney cancer for the study populations of patients with T2D and no history of any OAC

eTable 21. Characteristics of the GLP-1RA/no metformin group and metformin/no GLP-1RA group before and after matched for covariates related to liver cancer for the study populations of patients with T2D and no history of any OAC

eTable 22. Characteristics of the GLP-1RA/no metformin group and metformin/no GLP-1RA group before and after matched for covariates related to ovarian cancer for the study populations of women with T2D and no history of any OAC

eTable 23. Characteristics of the GLP-1RA/no metformin group and metformin/no GLP-1RA group before and after matched for covariates related to pancreatic cancer for the study populations of patients with T2D and no history of any OAC

eTable 24. Characteristics of the GLP-1RA/no metformin group and metformin/no GLP-1RA group before and after matched for covariates related to thyroid cancer for the

study populations of patients with T2D and no history of any OAC

eTable 25. Characteristics of the GLP-1RA/no metformin group and metformin/no GLP-1RA group before and after matched for covariates related to meningioma for the study populations of patients with T2D and no history of any OAC

eTable 26. Characteristics of the GLP-1RA/no metformin group and metformin/no GLP-1RA group before and after matched for covariates related to multiple myeloma for the study populations of patients with T2D and no history of any OAC

This supplemental material has been provided by the authors to give readers additional information about their work.

© 2024 Wang L et al. *JAMA Network Open.*

eAppendix. Database

The data used in this study were collected and analyzed on April 26, 2024 within the TriNetX Analytics platform based on the"Research US Collaborative Network". We used the TriNetX platform to access aggregated and de-identified electronic health records (EHRs) of 113 million patients from 64 healthcare organizations in the US across 50 states, covering diverse geographic regions (22% Northeast, 16% Midwest, 39% South, 13% West, 10% Unspecified), age, race/ethnic, income and insurance groups and clinical setting. TriNetX, LLC is compliant with the Health Insurance Portability and Accountability Act (HIPAA). Any data displayed on the TriNetX Platform in aggregate form, or any patient level data provided in a data set generated by the TriNetX Platform only contains de-identified data as per the de-identification standard defined in Section §164.514(a) of the HIPAA Privacy Rule. TriNetX built-in analytic functions (e.g., incidence, prevalence, outcomes analysis, survival analysis, propensity score matching) allow for patient-level analyses, while only reporting population-level data. The MetroHealth System, Cleveland OH, IRB determined research in the way described here, is not Human Subject Research and therefore IRB is not required.

TriNetX is a platform that de-identifies and aggregates electronic health record (EHR) data from contributing healthcare systems, most of which are large academic medical institutions with both inpatient and outpatient facilities at multiple locations, across all 50 states in the US. TriNetX Analytics provides web-based and secure access to patient EHR data from hospitals, primary care, and specialty treatment providers, covering diverse geographic locations, age groups, racial and ethnic groups, income levels and insurance types including various commercial insurances, governmental insurance (Medicare and Medicaid), self-pay/uninsured, worker compensation insurance, military/VA insurance among others.

Self-reported sex (female, male), race and ethnicity data in TriNetX comes from the underlying clinical EHR systems of the contributing healthcare systems. TriNetX maps race and ethnicity data from the contributing healthcare systems to the following categories: (1) Race: Asian, American Indian or Alaskan Native, Black or African American, Native Hawaiian or Other, White, Unknown race; and (2) Ethnicity: Hispanic or Latino, Not Hispanic or Latino, Unknown Ethnicity.

TriNetX completes an intensive data preprocessing stage to minimize missing values. TriNetX maps the data to a consistent clinical data model with a consistent semantic meaning so that the data can be queried consistently regardless of the underlying data source. All covariates are either binary, categorical (which expands to a set of binary columns), or continuous but essentially guaranteed to exist. Age is guaranteed to exist. Missing sex values are represented using "Unknown Sex". The missing data for race and ethnicity are presented as "Unknown race" or "Unknown Ethnicity". For other variables including medical conditions, procedures, lab tests and socio-economic determinant health, the value is either present or absent so "missing" is not pertinent.

© 2024 Wang L et al. *JAMA Network Open.*

eTable 1. Clinical diagnosis, and other codes used in the platform that are used to determine the status of variables for study population definitions, exposures, outcomes, and those for propensity-score matching for groups

**Study population**	T2D	Type 2 diabetes mellitus (International Classification of Diseases, Tenth Revision (ICD-10 or ICD-10 code: E11)	present/absent
**Exposure** (the first prescription of GLP-1RA, insulins or metformin in 4/2005-11/2018	GLP-1RAs	A10BJGlucagon-like peptide-1 (GLP-1) analogues (The Anatomical Therapeutic Chemical (ATC) code: A10BJ)	present/absent
	Insulins	INSULINS AND ANALOGUES (ATC code: A10A)	present/absent
	Metformin	Metformin (RxNorm code: 6809)	present/absent
**Outcomes (13 OACs) (15-year follow-up following the exposure or index event)**	Esophageal cancer	Malignant neoplasm of esophagus (ICD-10 code: C15)	present/absent
	Breast cancer	Malignant neoplasm of breast (ICD-10 code: C50)	
	Colorectal cancer	• Malignant neoplasm of colon (ICD-10 code: C18)<br>• Malignant neoplasm of rectosigmoid junction (ICD-10 code: C19)<br>• Malignant neoplasm of rectum (ICD-10 code: C20)	
	Endometrial cancer	Malignant neoplasm of endometrium (ICD-10 code: C54.1)	
	Gallbladder cancer	Malignant neoplasm of gallbladder (ICD-10 code: C23)	
	Stomach cancer	Malignant neoplasm of stomach (ICD-10 code: C16)	
	Kidney cancer	Malignant neoplasm of kidney, except renal pelvis (ICD-10 code: C64)	
	Ovarian cancer	Malignant neoplasm of ovary (ICD-10 code: C56)	
	Pancreatic cancer	Malignant neoplasm of pancreas (ICD-10 code: C25)	
	Thyroid cancer	Malignant neoplasm of thyroid gland (ICD-10 code: C73)	

© 2024 Wang L et al. *JAMA Network Open.*

	Hepatocellular carcinoma	Liver cell carcinoma (ICD-10 code: C22.0)	
	Meningioma	Malignant neoplasm of meninges (ICD-10 code: C70)	
	Multiple myeloma	Multiple myeloma (ICD-10 code: C90.0)	
**Demographics**<br><br>**(variables to be propensity-score matched)**	Age at the index event	Age	continuous
	Female	F	present/absent
	Male	M	present/absent
	Asian	Asian (Demographics: 2028-9)	present/absent
	American Indian or Alaska Native	American Indian or Alaska Native (Demographics: 1002--5)	Ik8ju76543e2w1aq`
	Black or African American	Black or African American (Demographics: 2054-5)	present/absent
	Native Hawaiian or Other Pacific Islander	Native Hawaiian or Other Pacific Islander (Demographics: 2076-8)	
	White	White (Demographics: 2106-3)	present/absent
	Hispanic/Latino	Hispanic or Latino (Demographics: 2135-2)	present/absent
	Not Hispanic or Latino	Not Hispanic or Latino (Demographics: 2186-5)	present/absent
	Unknown race	Unknown Race (Demographics: 2131-1)	present/absent
	Unknown ethnicity	Unknown Ethnicity (Demographics: UN)	present/absent
**Adverse socioeconomic determinants of health, lifestyle factors, medical conditions, medication**	Adverse socioeconomic and psychosocial circumstances	Persons with potential health hazards related to socioeconomic and psychosocial circumstances (ICD-10 code: Z55-Z65)<br>• Problems related to education and literacy (ICD-10 code: Z55)<br>• Problems related to employment and unemployment (ICD-10 code: Z56)<br>• Problems related to housing and economic	present/absent

© 2024 Wang L et al. *JAMA Network Open.*

**prescriptions, (anytime to 1 day before the index event)**		circumstances (ICD-10 code: Z59)<br>• Problems related to social environment (ICD-10 code: Z60)<br>• Problems related to upbringing (ICD-10 code: Z62)<br>• Other problems related to primary support group, including family circumstances (ICD-10 code: Z63)<br>• Problems related to certain psychosocial circumstances ICD-10 code: Z64)<br>• Problems related to other psychosocial circumstances (ICD-10 code: Z65)	
	Family history of cancer	Family history of primary malignant neoplasm (ICD-10 code: Z80)	present/absent
	Family history of cancer of digestive organs	Family history of malignant neoplasm of digestive organs (ICD-10 code: Z80.0)	
	Family history of colonic polyps	Family history of colonic polyps (ICD-10 code: Z83.71)	
	Genetic susceptibility to cancer	Genetic susceptibility to malignant neoplasm (ICD-10 code: Z15.0)	present/absent
	Personal history of cancer	Personal history of malignant neoplasm (ICD-10 code: Z85)	present/absent
	Obesity/overweight	Overweight and obesity (ICD-10 code: E66)<br>Body mass index [BMI] 40 or greater, adult (ICD-10 code: Z68.4)<br>Body mass index [BMI] 30-39, adult (ICD-10 code: Z68.3)	present/absent

		Body mass index [BMI] 25.0-25.9, adult (ICD-10 code: Z68.25)<br>Body mass index [BMI] 26.0-26.9, adult (ICD-10 code: Z68.26)<br>Body mass index [BMI] 27.0-27.9, adult (ICD-10 code: Z68.27)<br>Body mass index [BMI] 28.0-28.9, adult (ICD-10 code: Z68.28)<br>Body mass index [BMI] 29.0-29.9, adult (ICD-10 code: Z68.29)	
	Alcohol related disorders	Alcohol related disorders (ICD-10 code: F10)	present/absent
	Nicotine dependence	Nicotine dependence (ICD-10 code: F17)	present/absent
	Crohn's disease	Crohn's disease [regional enteritis] (ICD-10 code: K50)	present/absent
	Ulcerative colitis	Ulcerative colitis (ICD-10 code: K51)	present/absent
	Cystic fibrosis	Cystic fibrosis (ICD-10 code: E84)	present/absent
	Colon polyps	Polyp of colon (ICD-10 code: K63.5)	present/absent
	Benign neoplasm of colon and rectum	Benign neoplasm of colon, rectum, anus and anal canal (ICD-10 code: D12)	
	Bariatric surgery	Bariatric surgery status (ICD-10 code: Z98.84)	present/absent
	Encounter for screening for malignant neoplasms	Encounter for screening for malignant neoplasms (ICD-10 code: Z12)	present/absent
	Colonoscopy	Colonoscopy (The Current Procedural Terminology (CPT) code: 1022231)	
	DPP-4 inhibitors	Dipeptidyl peptidase 4 (DPP-4) inhibitors (ATC code: A10BH)	present/absent

© 2024 Wang L et al. *JAMA Network Open.*

	SGLT2 inhibitors	Sodium-glucose co-transporter 2 (SGLT2) inhibitors (ATC code: A10BK)	present/absent
	Sulfonylureas	Sulfonylureas (ATC code: A10BB)	present/absent
	Thiazolidinediones	Thiazolidinediones (ATC code: A10BG)	present/absent
	Alpha glucosidase inhibitors	Alpha glucosidase inhibitors (ATC code: A10BF)	present/absent
	Other blood glucose lowering drugs	Other blood glucose lowering drugs, excl. insulins (ATC code: A10BJ)	present/absent

© 2024 Wang L et al. *JAMA Network Open.*

eTable 2. Characteristics of the GLP-1RA/no insulin group and insulin/no GLP-1RA group before and after matched for baseline covariates related to esophageal cancer for the study populations of patients with T2D and no history of any OAC

Group 1 (N = 48,983) and group 2 (N = 1,044,745) characteristics before propensity score matching

Demographics

Group			Mean ± SD	Patients	% of Group	P-Value	SMD
1<br>2	AI	Age at Index	55.9 +/- 11.7<br>61.8 +/- 15.9	48,983<br>1,044,745	100%<br>100%	<0.001	0.420
1<br>2	2106-3	White		32,592<br>633,989	66.5%<br>60.7%	<0.001	0.122
1<br>2	1002-5	American Indian or Alaska Native		199<br>3,443	0.4%<br>0.3%	0.004	0.013
1<br>2	UNK	Unknown Race		7,099<br>142,470	14.5%<br>13.6%	<0.001	0.025
1<br>2	F	Female		26,011<br>476,110	53.1%<br>45.6%	<0.001	0.151
1<br>2	2076-8	Native Hawaiian or Other Pacific Islander		205<br>10,677	0.4%<br>1.0%	<0.001	0.071
1<br>2	UN	Unknown Gender		2,252<br>27,321	4.6%<br>2.6%	<0.001	0.106
1<br>2	2186-5	Not Hispanic or Latino		33,188<br>659,375	67.8%<br>63.1%	<0.001	0.098
1<br>2	2135-2	Hispanic or Latino		4,151<br>94,136	8.5%<br>9.0%	<0.001	0.019
1<br>2	2054-5	Black or African American		6,265<br>178,267	12.8%<br>17.1%	<0.001	0.120
1<br>2	M	Male		20,720<br>541,314	42.3%<br>51.8%	<0.001	0.191
1<br>2	2028-9	Asian		1,204<br>41,822	2.5%<br>4.0%	<0.001	0.087

Diagnosis

Group			Mean ± SD	Patients	% of Group	P-Value	SMD
1<br>2	Z55-Z65	Persons with potential health hazards related to socioeconomic and		686<br>12,021	1.4%<br>1.2%	<0.001	0.022

© 2024 Wang L et al. *JAMA Network Open.*

		psychosocial circumstances				
1<br>2	E66	Overweight and obesity	18,401<br>166,445	37.6%<br>15.9%	<0.001	0.504
1<br>2	Z68.3	Body mass index [BMI] 30-39, adult	4,204<br>43,128	8.6%<br>4.1%	<0.001	0.183
1<br>2	Z68.4	Body mass index [BMI] 40 or greater, adult	3,886<br>33,396	7.9%<br>3.2%	<0.001	0.208
1<br>2	Z68.25	Body mass index [BMI] 25.0-25.9, adult	157<br>3,510	0.3%<br>0.3%	0.563	0.003
1<br>2	Z68.26	Body mass index [BMI] 26.0-26.9, adult	171<br>3,632	0.3%<br>0.3%	0.957	<0.001
1<br>2	Z68.27	Body mass index [BMI] 27.0-27.9, adult	288<br>4,209	0.6%<br>0.4%	<0.001	0.026
1<br>2	Z68.28	Body mass index [BMI] 28.0-28.9, adult	344<br>4,508	0.7%<br>0.4%	<0.001	0.036
1<br>2	Z68.29	Body mass index [BMI] 29.0-29.9, adult	398<br>4,843	0.8%<br>0.5%	<0.001	0.044
1<br>2	F10	Alcohol related disorders	563<br>28,538	1.1%<br>2.7%	<0.001	0.115
1<br>2	Z80	Family history of primary malignant neoplasm	2,042<br>19,398	4.2%<br>1.9%	<0.001	0.136
1<br>2	Z15.0	Genetic susceptibility to malignant neoplasm	24<br>156	0.0%<br>0.0%	<0.001	0.019
1<br>2	Z12	Encounter for screening for malignant neoplasms	12,272<br>105,217	25.1%<br>10.1%	<0.001	0.402
1<br>2	Z85	Personal history of malignant neoplasm	1,239<br>38,294	2.5%<br>3.7%	<0.001	0.066
1<br>2	Z98.84	Bariatric surgery status	632<br>5,136	1.3%<br>0.5%	<0.001	0.085
1<br>2	F17	Nicotine dependence	3,593<br>96,860	7.3%<br>9.3%	<0.001	0.070

© 2024 Wang L et al. *JAMA Network Open.*

1<br>2	E66.0	Obesity due to excess calories	9,157<br>69,998	18.7%<br>6.7%	<0.001	0.366
1<br>2	E66.2	Morbid (severe) obesity with alveolar hypoventilation	150<br>3,643	0.3%<br>0.3%	0.118	0.007
1<br>2	E66.3	Overweight	1,010<br>6,796	2.1%<br>0.7%	<0.001	0.122
1<br>2	E66.8	Other obesity	235<br>1,298	0.5%<br>0.1%	<0.001	0.065
1<br>2	E66.9	Obesity, unspecified	13,805<br>118,555	28.2%<br>11.3%	<0.001	0.433
1<br>2	Z72.0	Tobacco use	1,064<br>13,876	2.2%<br>1.3%	<0.001	0.064
1<br>2	K21	Gastro-esophageal reflux disease	9,681<br>149,344	19.8%<br>14.3%	<0.001	0.146
1<br>2	K22.7	Barrett's esophagus	399<br>4,995	0.8%<br>0.5%	<0.001	0.042
1<br>2	K22.0	Achalasia of cardia	32<br>783	0.1%<br>0.1%	0.446	0.004
1<br>2	D69.41	Evans syndrome	0<br>33	0%<br>0.0%	0.214	0.008
1<br>2	D50.1	Sideropenic dysphagia	86<br>1,038	0.2%<br>0.1%	<0.001	0.021
1<br>2	S27.81	Injury of esophagus (thoracic part)	0<br>55	0%<br>0.0%	0.108	0.010
1<br>2	C34	Malignant neoplasm of bronchus and lung	67<br>10,154	0.1%<br>1.0%	<0.001	0.113
1<br>2	C06	Malignant neoplasm of other and unspecified parts of mouth	10<br>1,096	0.0%<br>0.1%	<0.001	0.034
1<br>2	C32	Malignant neoplasm of larynx	10<br>1,426	0.0%<br>0.1%	<0.001	0.041
1<br>2	B97.7	Papillomavirus as the cause of diseases classified elsewhere	114<br>900	0.2%<br>0.1%	<0.001	0.037
1<br>2	R87.81	High risk human papillomavirus (HPV) DNA test positive from female genital organs	158<br>830	0.3%<br>0.1%	<0.001	0.054

© 2024 Wang L et al. *JAMA Network Open.*

1<br>2	Z80.0	Family history of malignant neoplasm of digestive organs		784<br>6,889	1.6%<br>0.7%	<0.00<br>1	0.089
1<br>2	D10	Benign neoplasm of mouth and pharynx		60<br>1,059	0.1%<br>0.1%	0.153	0.006
1<br>2	D13.0	Benign neoplasm of esophagus		11<br>252	0.0%<br>0.0%	0.816	0.001

Medication

Group			Mean ± SD	Patients	% of Group	P-Value	SMD
1<br>2	A10B<br>A	Biguanides		27,075<br>199,802	55.3%<br>19.1%	<0.00<br>1	0.806
1<br>2	A10B<br>B	Sulfonylureas		14,077<br>125,703	28.7%<br>12.0%	<0.00<br>1	0.424
1<br>2	A10B<br>F	Alpha glucosidase inhibitors		229<br>1,718	0.5%<br>0.2%	<0.00<br>1	0.054
1<br>2	A10B<br>G	Thiazolidinediones		4,107<br>35,435	8.4%<br>3.4%	<0.00<br>1	0.213
1<br>2	A10B<br>H	Dipeptidyl peptidase 4 (DPP-4) inhibitors		9,485<br>44,595	19.4%<br>4.3%	<0.00<br>1	0.481
1<br>2	A10B<br>K	Sodium-glucose co-transporter 2 (SGLT2) inhibitors		4,808<br>6,447	9.8%<br>0.6%	<0.00<br>1	0.423
1<br>2	A10B<br>X	Other blood glucose lowering drugs, excl. insulins		614<br>7,048	1.3%<br>0.7%	<0.00<br>1	0.059

Group 1 (N = 48,437) and group 2 (N = 48,437) characteristics after propensity score matching

Demographics

Group			Mean ± SD	Patients	% of Group	P-Value	SMD
1<br>2	AI	Age at Index	55.9 +/- 11.7<br>56.2 +/- 13.4	48,437<br>48,437	100%<br>100%	<0.00<br>1	0.022
1<br>2	2106-3	White		32,239<br>33,166	66.6%<br>68.5%	<0.00<br>1	0.041
1<br>2	1002-5	American Indian or Alaska Native		196<br>196	0.4%<br>0.4%	1	<0.001
1<br>2	UNK	Unknown Race		6,973<br>6,605	14.4%<br>13.6%	0.001	0.022
1<br>2	F	Female		25,706<br>26,204	53.1%<br>54.1%	0.001	0.021

© 2024 Wang L et al. *JAMA Network Open.*

1<br>2	2076-8	Native Hawaiian or Other Pacific Islander		204<br>163	0.4%<br>0.3%	0.032	0.014
1<br>2	UN	Unknown Gender		2,163<br>1,997	4.5%<br>4.1%	0.009	0.017
1<br>2	2186-5	Not Hispanic or Latino		32,828<br>33,324	67.8%<br>68.8%	0.001	0.022
1<br>2	2135-2	Hispanic or Latino		4,113<br>4,002	8.5%<br>8.3%	0.198	0.008
1<br>2	2054-5	Black or African American		6,223<br>5,878	12.8%<br>12.1%	0.001	0.022
1<br>2	M	Male		20,568<br>20,236	42.5%<br>41.8%	0.031	0.014
1<br>2	2028-9	Asian		1,195<br>1,080	2.5%<br>2.2%	0.015	0.016

Diagnosis

Group			Mean ± SD	Patients	% of Group	P-Value	SMD
1<br>2	Z55-Z65	Persons with potential health hazards related to socioeconomic and psychosocial circumstances		677<br>546	1.4%<br>1.1%	<0.001	0.024
1<br>2	E66	Overweight and obesity		17,983<br>18,020	37.1%<br>37.2%	0.806	0.002
1<br>2	Z68.3	Body mass index [BMI] 30-39, adult		4,040<br>3,695	8.3%<br>7.6%	<0.001	0.026
1<br>2	Z68.4	Body mass index [BMI] 40 or greater, adult		3,779<br>3,456	7.8%<br>7.1%	<0.001	0.025
1<br>2	Z68.25	Body mass index [BMI] 25.0-25.9, adult		156<br>138	0.3%<br>0.3%	0.293	0.007
1<br>2	Z68.26	Body mass index [BMI] 26.0-26.9, adult		171<br>146	0.4%<br>0.3%	0.160	0.009
1<br>2	Z68.27	Body mass index [BMI] 27.0-27.9, adult		284<br>213	0.6%<br>0.4%	0.001	0.021

© 2024 Wang L et al. *JAMA Network Open.*

1<br>2	Z68.28	Body mass index [BMI] 28.0-28.9, adult	332<br>282	0.7%<br>0.6%	0.043	0.013
1<br>2	Z68.29	Body mass index [BMI] 29.0-29.9, adult	382<br>340	0.8%<br>0.7%	0.117	0.010
1<br>2	F10	Alcohol related disorders	562<br>444	1.2%<br>0.9%	<0.001	0.024
1<br>2	Z80	Family history of primary malignant neoplasm	1,993<br>1,795	4.1%<br>3.7%	0.001	0.021
1<br>2	Z15.0	Genetic susceptibility to malignant neoplasm	22<br>10	0.0%<br>0.0%	0.034	0.014
1<br>2	Z12	Encounter for screening for malignant neoplasms	11,921<br>11,527	24.6%<br>23.8%	0.003	0.019
1<br>2	Z85	Personal history of malignant neoplasm	1,229<br>1,075	2.5%<br>2.2%	0.001	0.021
1<br>2	Z98.84	Bariatric surgery status	614<br>588	1.3%<br>1.2%	0.450	0.005
1<br>2	F17	Nicotine dependence	3,571<br>3,141	7.4%<br>6.5%	<0.001	0.035
1<br>2	E66.0	Obesity due to excess calories	8,908<br>8,817	18.4%<br>18.2%	0.450	0.005
1<br>2	E66.2	Morbid (severe) obesity with alveolar hypoventilation	149<br>131	0.3%<br>0.3%	0.281	0.007
1<br>2	E66.3	Overweight	980<br>877	2.0%<br>1.8%	0.016	0.016
1<br>2	E66.8	Other obesity	219<br>205	0.5%<br>0.4%	0.496	0.004
1<br>2	E66.9	Obesity, unspecified	13,461<br>13,612	27.8%<br>28.1%	0.280	0.007
1<br>2	Z72.0	Tobacco use	1,040<br>924	2.1%<br>1.9%	0.008	0.017
1<br>2	K21	Gastro-esophageal reflux disease	9,520<br>9,180	19.7%<br>19.0%	0.006	0.018
1<br>2	K22.7	Barrett's esophagus	390<br>382	0.8%<br>0.8%	0.773	0.002
1<br>2	K22.0	Achalasia of cardia	31<br>23	0.1%<br>0.0%	0.276	0.007

© 2024 Wang L et al. *JAMA Network Open.*

1<br>2	D69.41	Evans syndrome		0<br>0	0%<br>0%	--	--
1<br>2	D50.1	Sideropenic dysphagia		83<br>80	0.2%<br>0.2%	0.814	0.002
1<br>2	S27.81	Injury of esophagus (thoracic part)		0<br>10	0%<br>0.0%	0.002	0.020
1<br>2	C34	Malignant neoplasm of bronchus and lung		67<br>69	0.1%<br>0.1%	0.864	0.001
1<br>2	C06	Malignant neoplasm of other and unspecified parts of mouth		10<br>28	0.0%<br>0.1%	0.003	0.019
1<br>2	C32	Malignant neoplasm of larynx		10<br>15	0.0%<br>0.0%	0.317	0.006
1<br>2	B97.7	Papillomavirus as the cause of diseases classified elsewhere		114<br>108	0.2%<br>0.2%	0.687	0.003
1<br>2	R87.81	High risk human papillomavirus (HPV) DNA test positive from female genital organs		154<br>117	0.3%<br>0.2%	0.024	0.014
1<br>2	Z80.0	Family history of malignant neoplasm of digestive organs		764<br>654	1.6%<br>1.4%	0.003	0.019
1<br>2	D10	Benign neoplasm of mouth and pharynx		59<br>59	0.1%<br>0.1%	1	<0.001
1<br>2	D13.0	Benign neoplasm of esophagus		11<br>21	0.0%<br>0.0%	0.077	0.011
**Medication**							
Group			Mean ± SD	Patients	% of Group	P-Value	SMD
1<br>2	A10BA	Biguanides		26,535<br>27,001	54.8%<br>55.7%	0.003	0.019
1<br>2	A10BB	Sulfonylureas		13,752<br>14,240	28.4%<br>29.4%	0.001	0.022
1<br>2	A10BF	Alpha glucosidase inhibitors		221<br>198	0.5%<br>0.4%	0.260	0.007
1<br>2	A10BG	Thiazolidinediones		4,019<br>4,180	8.3%<br>8.6%	0.063	0.012
1<br>2	A10BH	Dipeptidyl peptidase 4 (DPP-4) inhibitors		9,079<br>9,086	18.7%<br>18.8%	0.954	<0.001

© 2024 Wang L et al. *JAMA Network Open.*

1<br>2	A10B<br>K	Sodium-glucose co-transporter 2 (SGLT2) inhibitors	4,290<br>3,835	8.9%<br>7.9%	<0.001	0.034
1<br>2	A10B<br>X	Other blood glucose lowering drugs, excl. insulins	604<br>585	1.2%<br>1.2%	0.579	0.004

© 2024 Wang L et al. *JAMA Network Open.*

eTable 3. Characteristics of the GLP-1RA/no insulin group and insulin/no GLP-1RA group before and after matched for covariates related to breast cancer for the study populations of women (age 55 and older) with T2D and no history of any OAC

Group 1 (N = 13,865) and group 2 (N = 284,997) characteristics before propensity score matching

Demographics

Group			Mean ± SD	Patients	% of Group	P-Value	SMD
1<br>2	AI	Age at Index	63.5 +/- 6.2<br>67.4 +/- 7.5	13,865<br>284,997	100%<br>100%	<0.001	0.566
1<br>2	2106-3	White		9,776<br>174,355	70.5%<br>61.2%	<0.001	0.198
1<br>2	1002-5	American Indian or Alaska Native		50<br>874	0.4%<br>0.3%	0.264	0.009
1<br>2	UNK	Unknown Race		1,328<br>31,022	9.6%<br>10.9%	<0.001	0.043
1<br>2	2076-8	Native Hawaiian or Other Pacific Islander		48<br>2,664	0.3%<br>0.9%	<0.001	0.074
1<br>2	UN	Unknown Ethnicity		2,902<br>75,794	20.9%<br>26.6%	<0.001	0.133
1<br>2	2186-5	Not Hispanic or Latino		9,979<br>184,217	72.0%<br>64.6%	<0.001	0.158
1<br>2	2135-2	Hispanic or Latino		984<br>24,986	7.1%<br>8.8%	<0.001	0.062
1<br>2	2054-5	Black or African American		2,027<br>54,813	14.6%<br>19.2%	<0.001	0.123
1<br>2	2028-9	Asian		279<br>12,141	2.0%<br>4.3%	<0.001	0.129

Diagnosis

Group			Mean ± SD	Patients	% of Group	P-Value	SMD
1<br>2	Z55-Z65	Persons with potential health hazards related to socioeconomic and psychosocial circumstances		196<br>2,666	1.4%<br>0.9%	<0.001	0.044
1<br>2	E66	Overweight and obesity		4,915<br>51,523	35.4%<br>18.1%	<0.001	0.400

© 2024 Wang L et al. *JAMA Network Open.*

1<br>2	Z68.3	Body mass index [BMI] 30-39, adult	983<br>11,795	7.1%<br>4.1%	<0.00<br>1	0.128
1<br>2	Z68.4	Body mass index [BMI] 40 or greater, adult	857<br>10,539	6.2%<br>3.7%	<0.00<br>1	0.115
1<br>2	Z68.25	Body mass index [BMI] 25.0-25.9, adult	32<br>663	0.2%<br>0.2%	0.965	<0.001
1<br>2	Z68.26	Body mass index [BMI] 26.0-26.9, adult	39<br>662	0.3%<br>0.2%	0.244	0.010
1<br>2	Z68.27	Body mass index [BMI] 27.0-27.9, adult	63<br>803	0.5%<br>0.3%	<0.00<br>1	0.029
1<br>2	Z68.28	Body mass index [BMI] 28.0-28.9, adult	72<br>920	0.5%<br>0.3%	<0.00<br>1	0.030
1<br>2	Z68.29	Body mass index [BMI] 29.0-29.9, adult	75<br>989	0.5%<br>0.3%	<0.00<br>1	0.029
1<br>2	F10	Alcohol related disorders	84<br>3,218	0.6%<br>1.1%	<0.00<br>1	0.056
1<br>2	Z80	Family history of primary malignant neoplasm	791<br>7,754	5.7%<br>2.7%	<0.00<br>1	0.149
1<br>2	Z15.0	Genetic susceptibility to malignant neoplasm	10<br>60	0.1%<br>0.0%	<0.00<br>1	0.024
1<br>2	Z12	Encounter for screening for malignant neoplasms	4,506<br>44,384	32.5%<br>15.6%	<0.00<br>1	0.404
1<br>2	Z85	Personal history of malignant neoplasm	421<br>10,433	3.0%<br>3.7%	<0.00<br>1	0.035
1<br>2	Z98.84	Bariatric surgery status	234<br>2,038	1.7%<br>0.7%	<0.00<br>1	0.089
1<br>2	E66.0	Obesity due to excess calories	2,298<br>22,234	16.6%<br>7.8%	<0.00<br>1	0.271
1<br>2	E66.2	Morbid (severe) obesity with alveolar hypoventilation	40<br>1,313	0.3%<br>0.5%	0.003	0.028
1<br>2	E66.3	Overweight	262<br>1,855	1.9%<br>0.7%	<0.00<br>1	0.111

1<br>2	E66.8	Other obesity		64<br>367	0.5%<br>0.1%	<0.001	0.061
1<br>2	E66.9	Obesity, unspecified		3,747<br>36,255	27.0%<br>12.7%	<0.001	0.364
1<br>2	O92.7	Other and unspecified disorders of lactation		0<br>10	0%<br>0.0%	0.485	0.008
1<br>2	Z79.890	Hormone replacement therapy		88<br>1,050	0.6%<br>0.4%	<0.001	0.038
1<br>2	Z92.23	Personal history of estrogen therapy		10<br>111	0.1%<br>0.0%	0.058	0.014
1<br>2	Z98.82	Breast implant status		10<br>75	0.1%<br>0.0%	0.002	0.021
1<br>2	Z80.3	Family history of malignant neoplasm of breast		392<br>3,435	2.8%<br>1.2%	<0.001	0.116
1<br>2	R92	Abnormal and inconclusive findings on diagnostic imaging of breast		1,289<br>11,640	9.3%<br>4.1%	<0.001	0.210
1<br>2	D24	Benign neoplasm of breast		113<br>1,201	0.8%<br>0.4%	<0.001	0.050
1<br>2	N60	Benign mammary dysplasia		496<br>4,263	3.6%<br>1.5%	<0.001	0.133
1<br>2	N62	Hypertrophy of breast		79<br>529	0.6%<br>0.2%	<0.001	0.063
1<br>2	N63	Unspecified lump in breast		700<br>7,212	5.0%<br>2.5%	<0.001	0.132
1<br>2	N64	Other disorders of breast		759<br>7,195	5.5%<br>2.5%	<0.001	0.151
1<br>2	N61	Inflammatory disorders of breast		70<br>781	0.5%<br>0.3%	<0.001	0.037
1<br>2	D05	Carcinoma in situ of breast		31<br>381	0.2%<br>0.1%	0.005	0.021
**Procedure**							
Group			Mean ± SD	Patients	% of Group	P-Value	SMD
1<br>2	C1789	Prosthesis, breast (implantable)		0<br>11	0%<br>0.0%	0.464	0.009

Group			Mean ± SD	Patients	% of Group	P-Value	SMD
1<br>2	58300	Insertion of intrauterine device (IUD)		18<br>66	0.1%<br>0.0%	<0.001	0.039
1<br>2	1001	Radiation		28<br>1,571	0.2%<br>0.6%	<0.001	0.057

Medication

Group			Mean ± SD	Patients	% of Group	P-Value	SMD
1<br>2	A10BA	Biguanides		7,389<br>59,166	53.3%<br>20.8%	<0.001	0.716
1<br>2	A10BB	Sulfonylureas		4,083<br>35,332	29.4%<br>12.4%	<0.001	0.429
1<br>2	A10BF	Alpha glucosidase inhibitors		64<br>488	0.5%<br>0.2%	<0.001	0.052
1<br>2	A10BG	Thiazolidinediones		1,173<br>9,921	8.5%<br>3.5%	<0.001	0.211
1<br>2	A10BH	Dipeptidyl peptidase 4 (DPP-4) inhibitors		2,792<br>14,092	20.1%<br>4.9%	<0.001	0.471
1<br>2	A10BK	Sodium-glucose co-transporter 2 (SGLT2) inhibitors		1,294<br>1,768	9.3%<br>0.6%	<0.001	0.409
1<br>2	A10BX	Other blood glucose lowering drugs, excl. insulins		198<br>2,014	1.4%<br>0.7%	<0.001	0.070
1<br>2	HS200	CONTRACEPTIVES,SYSTEMIC		143<br>535	1.0%<br>0.2%	<0.001	0.109

Group 1 (N = 13,768) and group 2 (N = 13,768) characteristics after propensity score matching

Demographics

Group			Mean ± SD	Patients	% of Group	P-Value	SMD
1<br>2	AI	Age at Index	63.6 +/- 6.2<br>63.5 +/- 6.3	13,768<br>13,768	100%<br>100%	0.545	0.007
1<br>2	2106-3	White		9,696<br>9,941	70.4%<br>72.2%	0.001	0.039
1<br>2	1002-5	American Indian or Alaska Native		50<br>48	0.4%<br>0.3%	0.840	0.002
1<br>2	UNK	Unknown Race		1,322<br>1,234	9.6%<br>9.0%	0.068	0.022

Group	Code	Description	Mean ± SD	Patients	% of Group	P-Value	SMD
1<br>2	2076-8	Native Hawaiian or Other Pacific Islander		48<br>36	0.3%<br>0.3%	0.190	0.016
1<br>2	UN	Unknown Ethnicity		2,889<br>2,862	21.0%<br>20.8%	0.689	0.005
1<br>2	2186-5	Not Hispanic or Latino		9,901<br>10,010	71.9%<br>72.7%	0.142	0.018
1<br>2	2135-2	Hispanic or Latino		978<br>896	7.1%<br>6.5%	0.050	0.024
1<br>2	2054-5	Black or African American		2,017<br>1,940	14.6%<br>14.1%	0.186	0.016
1<br>2	2028-9	Asian		278<br>237	2.0%<br>1.7%	0.068	0.022
**Diagnosis**							
Group			Mean ± SD	Patients	% of Group	P-Value	SMD
1<br>2	Z55-Z65	Persons with potential health hazards related to socioeconomic and psychosocial circumstances		193<br>166	1.4%<br>1.2%	0.151	0.017
1<br>2	E66	Overweight and obesity		4,850<br>4,776	35.2%<br>34.7%	0.350	0.011
1<br>2	Z68.3	Body mass index [BMI] 30-39, adult		966<br>855	7.0%<br>6.2%	0.007	0.032
1<br>2	Z68.4	Body mass index [BMI] 40 or greater, adult		848<br>761	6.2%<br>5.5%	0.025	0.027
1<br>2	Z68.25	Body mass index [BMI] 25.0-25.9, adult		32<br>19	0.2%<br>0.1%	0.068	0.022
1<br>2	Z68.26	Body mass index [BMI] 26.0-26.9, adult		39<br>38	0.3%<br>0.3%	0.909	0.001
1<br>2	Z68.27	Body mass index [BMI] 27.0-27.9, adult		63<br>57	0.5%<br>0.4%	0.583	0.007
1<br>2	Z68.28	Body mass index [BMI] 28.0-28.9, adult		70<br>71	0.5%<br>0.5%	0.933	0.001

© 2024 Wang L et al. *JAMA Network Open.*

1<br>2	Z68.29	Body mass index [BMI] 29.0-29.9, adult	74<br>72	0.5%<br>0.5%	0.868	0.002
1<br>2	F10	Alcohol related disorders	83<br>58	0.6%<br>0.4%	0.035	0.025
1<br>2	Z80	Family history of primary malignant neoplasm	777<br>723	5.6%<br>5.3%	0.152	0.017
1<br>2	Z15.0	Genetic susceptibility to malignant neoplasm	10<br>10	0.1%<br>0.1%	1	<0.001
1<br>2	Z12	Encounter for screening for malignant neoplasms	4,437<br>4,292	32.2%<br>31.2%	0.060	0.023
1<br>2	Z85	Personal history of malignant neoplasm	417<br>380	3.0%<br>2.8%	0.184	0.016
1<br>2	Z98.84	Bariatric surgery status	230<br>203	1.7%<br>1.5%	0.191	0.016
1<br>2	E66.0	Obesity due to excess calories	2,271<br>2,200	16.5%<br>16.0%	0.246	0.014
1<br>2	E66.2	Morbid (severe) obesity with alveolar hypoventilation	40<br>40	0.3%<br>0.3%	1	<0.001
1<br>2	E66.3	Overweight	258<br>247	1.9%<br>1.8%	0.621	0.006
1<br>2	E66.8	Other obesity	63<br>68	0.5%<br>0.5%	0.661	0.005
1<br>2	E66.9	Obesity, unspecified	3,692<br>3,719	26.8%<br>27.0%	0.714	0.004
1<br>2	O92.7	Other and unspecified disorders of lactation	0<br>0	0%<br>0%	--	--
1<br>2	Z79.890	Hormone replacement therapy	88<br>70	0.6%<br>0.5%	0.151	0.017
1<br>2	Z92.23	Personal history of estrogen therapy	10<br>10	0.1%<br>0.1%	1	<0.001
1<br>2	Z98.82	Breast implant status	10<br>10	0.1%<br>0.1%	1	<0.001
1<br>2	Z80.3	Family history of malignant neoplasm of breast	385<br>355	2.8%<br>2.6%	0.264	0.013

© 2024 Wang L et al. *JAMA Network Open.*

1<br>2	R92	Abnormal and inconclusive findings on diagnostic imaging of breast		1,274<br>1,194	9.3%<br>8.7%	0.091	0.020
1<br>2	D24	Benign neoplasm of breast		113<br>91	0.8%<br>0.7%	0.122	0.019
1<br>2	N60	Benign mammary dysplasia		491<br>448	3.6%<br>3.3%	0.153	0.017
1<br>2	N62	Hypertrophy of breast		78<br>68	0.6%<br>0.5%	0.407	0.010
1<br>2	N63	Unspecified lump in breast		686<br>649	5.0%<br>4.7%	0.299	0.013
1<br>2	N64	Other disorders of breast		747<br>686	5.4%<br>5.0%	0.098	0.020
1<br>2	N61	Inflammatory disorders of breast		68<br>70	0.5%<br>0.5%	0.864	0.002
1<br>2	D05	Carcinoma in situ of breast		31<br>27	0.2%<br>0.2%	0.599	0.006

Procedure

Group			Mean ± SD	Patients	% of Group	P-Value	SMD
1<br>2	C1789	Prosthesis, breast (implantable)		0<br>10	0%<br>0.1%	0.002	0.038
1<br>2	58300	Insertion of intrauterine device (IUD)		18<br>18	0.1%<br>0.1%	1	<0.001
1<br>2	1001	Radiation		28<br>25	0.2%<br>0.2%	0.680	0.005

Medication

Group			Mean ± SD	Patients	% of Group	P-Value	SMD
1<br>2	A10BA	Biguanides		7,292<br>7,344	53.0%<br>53.3%	0.530	0.008
1<br>2	A10BB	Sulfonylureas		4,021<br>4,148	29.2%<br>30.1%	0.094	0.020
1<br>2	A10BF	Alpha glucosidase inhibitors		64<br>56	0.5%<br>0.4%	0.464	0.009
1<br>2	A10BG	Thiazolidinediones		1,162<br>1,184	8.4%<br>8.6%	0.635	0.006

© 2024 Wang L et al. *JAMA Network Open.*

1<br>2	A10B<br>H	Dipeptidyl peptidase 4 (DPP-4) inhibitors	2,714<br>2,789	19.7%<br>20.3%	0.258	0.014
1<br>2	A10B<br>K	Sodium-glucose co-transporter 2 (SGLT2) inhibitors	1,197<br>1,046	8.7%<br>7.6%	0.001	0.040
1<br>2	A10B<br>X	Other blood glucose lowering drugs, excl. insulins	194<br>215	1.4%<br>1.6%	0.295	0.013
1<br>2	HS200	CONTRACEPTIVE S,SYSTEMIC	139<br>135	1.0%<br>1.0%	0.808	0.003

© 2024 Wang L et al. *JAMA Network Open.*

eTable 4. Characteristics of the GLP-1RA/no insulin group and insulin/no GLP-1RA group before and after matched for covariates related to endometrial cancer for the study populations of women with T2D and no history of any OAC

Group 1 (N = 26,011) and group 2 (N = 476,110) characteristics before propensity score matching

Demographics

Group			Mean ± SD	Patients	% of Group	P-Value	SMD
1<br>2	AI	Age at Index	54.9 +/- 11.9<br>61.7 +/- 16.7	26,011<br>476,110	100%<br>100%	<0.001	0.469
1<br>2	2106-3	White		17,165<br>282,423	66.0%<br>59.3%	<0.001	0.138
1<br>2	1002-5	American Indian or Alaska Native		123<br>1,701	0.5%<br>0.4%	0.003	0.018
1<br>2	UNK	Unknown Race		2,772<br>55,979	10.7%<br>11.8%	<0.001	0.035
1<br>2	2076-8	Native Hawaiian or Other Pacific Islander		105<br>4,943	0.4%<br>1.0%	<0.001	0.075
1<br>2	UN	Unknown Ethnicity		5,078<br>124,092	19.5%<br>26.1%	<0.001	0.156
1<br>2	2186-5	Not Hispanic or Latino		18,384<br>304,990	70.7%<br>64.1%	<0.001	0.142
1<br>2	2135-2	Hispanic or Latino		2,549<br>47,028	9.8%<br>9.9%	0.682	0.003
1<br>2	2054-5	Black or African American		4,436<br>95,352	17.1%<br>20.0%	<0.001	0.077
1<br>2	2028-9	Asian		619<br>19,593	2.4%<br>4.1%	<0.001	0.098

Diagnosis

Group			Mean ± SD	Patients	% of Group	P-Value	SMD
1<br>2	Z55-Z65	Persons with potential health hazards related to socioeconomic and psychosocial circumstances		468<br>5,762	1.8%<br>1.2%	<0.001	0.048
1<br>2	E66	Overweight and obesity		10,477<br>87,968	40.3%<br>18.5%	<0.001	0.493

© 2024 Wang L et al. *JAMA Network Open.*

1<br>2	Z68.3	Body mass index [BMI] 30-39, adult	1,815<br>17,667	7.0%<br>3.7%	<0.00<br>1	0.146
1<br>2	Z68.4	Body mass index [BMI] 40 or greater, adult	2,154<br>18,645	8.3%<br>3.9%	<0.00<br>1	0.183
1<br>2	Z68.25	Body mass index [BMI] 25.0-25.9, adult	56<br>1,000	0.2%<br>0.2%	0.857	0.001
1<br>2	Z68.26	Body mass index [BMI] 26.0-26.9, adult	50<br>959	0.2%<br>0.2%	0.747	0.002
1<br>2	Z68.27	Body mass index [BMI] 27.0-27.9, adult	87<br>1,140	0.3%<br>0.2%	0.003	0.018
1<br>2	Z68.28	Body mass index [BMI] 28.0-28.9, adult	107<br>1,266	0.4%<br>0.3%	<0.00<br>1	0.025
1<br>2	Z68.29	Body mass index [BMI] 29.0-29.9, adult	119<br>1,376	0.5%<br>0.3%	<0.00<br>1	0.028
1<br>2	Z80	Family history of primary malignant neoplasm	1,430<br>11,367	5.5%<br>2.4%	<0.00<br>1	0.160
1<br>2	Z15.0	Genetic susceptibility to malignant neoplasm	18<br>100	0.1%<br>0.0%	<0.00<br>1	0.023
1<br>2	Z12	Encounter for screening for malignant neoplasms	7,877<br>65,044	30.3%<br>13.7%	<0.00<br>1	0.410
1<br>2	Z85	Personal history of malignant neoplasm	593<br>14,844	2.3%<br>3.1%	<0.00<br>1	0.052
1<br>2	Z98.84	Bariatric surgery status	474<br>3,408	1.8%<br>0.7%	<0.00<br>1	0.099
1<br>2	E66.0	Obesity due to excess calories	5,392<br>39,813	20.7%<br>8.4%	<0.00<br>1	0.356
1<br>2	E66.2	Morbid (severe) obesity with alveolar hypoventilation	82<br>1,919	0.3%<br>0.4%	0.029	0.015
1<br>2	E66.3	Overweight	535<br>3,181	2.1%<br>0.7%	<0.00<br>1	0.120
1<br>2	E66.8	Other obesity	134<br>702	0.5%<br>0.1%	<0.00<br>1	0.064

© 2024 Wang L et al. *JAMA Network Open.*

1<br>2	E66.9	Obesity, unspecified		7,879<br>61,596	30.3%<br>12.9%	<0.001	0.431
1<br>2	Z79.890	Hormone replacement therapy		137<br>1,374	0.5%<br>0.3%	<0.001	0.037
1<br>2	Z92.23	Personal history of estrogen therapy		10<br>124	0.0%<br>0.0%	0.233	0.007
1<br>2	E28.2	Polycystic ovarian syndrome		979<br>2,780	3.8%<br>0.6%	<0.001	0.219
1<br>2	Z80.41	Family history of malignant neoplasm of ovary		104<br>554	0.4%<br>0.1%	<0.001	0.056
1<br>2	Z80.49	Family history of malignant neoplasm of other genital organs		38<br>315	0.1%<br>0.1%	<0.001	0.025
1<br>2	N85.0	Endometrial hyperplasia		182<br>1,527	0.7%<br>0.3%	<0.001	0.053
1<br>2	D25	Leiomyoma of uterus		1,031<br>8,571	4.0%<br>1.8%	<0.001	0.130
1<br>2	D26	Other benign neoplasms of uterus		45<br>377	0.2%<br>0.1%	<0.001	0.026
1<br>2	D27	Benign neoplasm of ovary		95<br>902	0.4%<br>0.2%	<0.001	0.033
1<br>2	Z80.3	Family history of malignant neoplasm of breast		736<br>5,122	2.8%<br>1.1%	<0.001	0.127
**Procedure**							
Group			Mean ± SD	Patients	% of Group	P-Value	SMD
1<br>2	58300	Insertion of intrauterine device (IUD)		306<br>825	1.2%<br>0.2%	<0.001	0.123
1<br>2	1001	Radiation		53<br>2,303	0.2%<br>0.5%	<0.001	0.048
**Medication**							
Group			Mean ± SD	Patients	% of Group	P-Value	SMD
1<br>2	A10BA	Biguanides		14,271<br>93,949	54.9%<br>19.7%	<0.001	0.780
1<br>2	A10BB	Sulfonylureas		6,822<br>56,352	26.2%<br>11.8%	<0.001	0.373

© 2024 Wang L et al. *JAMA Network Open.*

1<br>2	A10BF	Alpha glucosidase inhibitors		112<br>784	0.4%<br>0.2%	<0.001	0.049
1<br>2	A10BG	Thiazolidinediones		1,834<br>15,689	7.1%<br>3.3%	<0.001	0.170
1<br>2	A10BH	Dipeptidyl peptidase 4 (DPP-4) inhibitors		4,637<br>20,235	17.8%<br>4.3%	<0.001	0.444
1<br>2	A10BK	Sodium-glucose co-transporter 2 (SGLT2) inhibitors		2,219<br>2,614	8.5%<br>0.5%	<0.001	0.391
1<br>2	A10BX	Other blood glucose lowering drugs, excl. insulins		297<br>3,287	1.1%<br>0.7%	<0.001	0.047
1<br>2	HS200	CONTRACEPTIVES,SYSTEMIC		1,345<br>5,117	5.2%<br>1.1%	<0.001	0.237
1<br>2	10324	tamoxifen		37<br>287	0.1%<br>0.1%	<0.001	0.026

Group 1 (N = 25,750) and group 2 (N = 25,750) characteristics after propensity score matching

Demographics

Group			Mean ± SD	Patients	% of Group	P-Value	SMD
1<br>2	AI	Age at Index	54.9 +/- 11.9<br>54.9 +/- 13.6	25,750<br>25,750	100%<br>100%	0.621	0.004
1<br>2	2106-3	White		16,960<br>17,446	65.9%<br>67.8%	<0.001	0.040
1<br>2	1002-5	American Indian or Alaska Native		122<br>118	0.5%<br>0.5%	0.796	0.002
1<br>2	UNK	Unknown Race		2,753<br>2,611	10.7%<br>10.1%	0.041	0.018
1<br>2	2076-8	Native Hawaiian or Other Pacific Islander		105<br>98	0.4%<br>0.4%	0.623	0.004
1<br>2	UN	Unknown Ethnicity		5,052<br>4,841	19.6%<br>18.8%	0.018	0.021
1<br>2	2186-5	Not Hispanic or Latino		18,166<br>18,423	70.5%<br>71.5%	0.013	0.022
1<br>2	2135-2	Hispanic or Latino		2,532<br>2,486	9.8%<br>9.7%	0.494	0.006
1<br>2	2054-5	Black or African American		4,407<br>4,265	17.1%<br>16.6%	0.094	0.015

© 2024 Wang L et al. *JAMA Network Open.*

1<br>2	2028-9	Asian		616<br>507	2.4%<br>2.0%	0.001	0.029

Diagnosis

Group			Mean ± SD	Patients	% of Group	P-Value	SMD
1<br>2	Z55-Z65	Persons with potential health hazards related to socioeconomic and psychosocial circumstances		462<br>447	1.8%<br>1.7%	0.616	0.004
1<br>2	E66	Overweight and obesity		10,268<br>10,283	39.9%<br>39.9%	0.893	0.001
1<br>2	Z68.3	Body mass index [BMI] 30-39, adult		1,777<br>1,633	6.9%<br>6.3%	0.011	0.022
1<br>2	Z68.4	Body mass index [BMI] 40 or greater, adult		2,098<br>1,888	8.1%<br>7.3%	0.001	0.031
1<br>2	Z68.25	Body mass index [BMI] 25.0-25.9, adult		56<br>48	0.2%<br>0.2%	0.432	0.007
1<br>2	Z68.26	Body mass index [BMI] 26.0-26.9, adult		50<br>40	0.2%<br>0.2%	0.291	0.009
1<br>2	Z68.27	Body mass index [BMI] 27.0-27.9, adult		87<br>55	0.3%<br>0.2%	0.007	0.024
1<br>2	Z68.28	Body mass index [BMI] 28.0-28.9, adult		106<br>79	0.4%<br>0.3%	0.047	0.018
1<br>2	Z68.29	Body mass index [BMI] 29.0-29.9, adult		118<br>98	0.5%<br>0.4%	0.173	0.012
1<br>2	Z80	Family history of primary malignant neoplasm		1,399<br>1,312	5.4%<br>5.1%	0.086	0.015
1<br>2	Z15.0	Genetic susceptibility to malignant neoplasm		17<br>11	0.1%<br>0.0%	0.257	0.010
1<br>2	Z12	Encounter for screening for malignant neoplasms		7,704<br>7,448	29.9%<br>28.9%	0.013	0.022

© 2024 Wang L et al. *JAMA Network Open.*

1<br>2	Z85	Personal history of malignant neoplasm		590<br>525	2.3%<br>2.0%	0.049	0.017
1<br>2	Z98.84	Bariatric surgery status		455<br>437	1.8%<br>1.7%	0.543	0.005
1<br>2	E66.0	Obesity due to excess calories		5,269<br>5,205	20.5%<br>20.2%	0.484	0.006
1<br>2	E66.2	Morbid (severe) obesity with alveolar hypoventilation		82<br>60	0.3%<br>0.2%	0.064	0.016
1<br>2	E66.3	Overweight		514<br>449	2.0%<br>1.7%	0.034	0.019
1<br>2	E66.8	Other obesity		130<br>104	0.5%<br>0.4%	0.088	0.015
1<br>2	E66.9	Obesity, unspecified		7,716<br>7,843	30.0%<br>30.5%	0.223	0.011
1<br>2	Z79.890	Hormone replacement therapy		136<br>105	0.5%<br>0.4%	0.045	0.018
1<br>2	Z92.23	Personal history of estrogen therapy		10<br>10	0.0%<br>0.0%	1	<0.001
1<br>2	E28.2	Polycystic ovarian syndrome		938<br>928	3.6%<br>3.6%	0.814	0.002
1<br>2	Z80.41	Family history of malignant neoplasm of ovary		99<br>86	0.4%<br>0.3%	0.338	0.008
1<br>2	Z80.49	Family history of malignant neoplasm of other genital organs		37<br>34	0.1%<br>0.1%	0.722	0.003
1<br>2	N85.0	Endometrial hyperplasia		180<br>164	0.7%<br>0.6%	0.387	0.008
1<br>2	D25	Leiomyoma of uterus		1,018<br>948	4.0%<br>3.7%	0.107	0.014
1<br>2	D26	Other benign neoplasms of uterus		43<br>35	0.2%<br>0.1%	0.365	0.008
1<br>2	D27	Benign neoplasm of ovary		94<br>64	0.4%<br>0.2%	0.017	0.021
1<br>2	Z80.3	Family history of malignant neoplasm of breast		724<br>660	2.8%<br>2.6%	0.081	0.015
**Procedure**							
Group			Mean ± SD	Patients	% of Group	P-Value	SMD

1<br>2	58300	Insertion of intrauterine device (IUD)		290<br>256	1.1%<br>1.0%	0.144	0.013
1<br>2	1001	Radiation		53<br>51	0.2%<br>0.2%	0.844	0.002
**Medication**							
Group			Mean ± SD	Patients	% of Group	P-Value	SMD
1<br>2	A10BA	Biguanides		14,013<br>14,280	54.4%<br>55.5%	0.018	0.021
1<br>2	A10BB	Sulfonylureas		6,700<br>6,948	26.0%<br>27.0%	0.013	0.022
1<br>2	A10BF	Alpha glucosidase inhibitors		109<br>107	0.4%<br>0.4%	0.892	0.001
1<br>2	A10BG	Thiazolidinediones		1,799<br>1,858	7.0%<br>7.2%	0.311	0.009
1<br>2	A10BH	Dipeptidyl peptidase 4 (DPP-4) inhibitors		4,454<br>4,607	17.3%<br>17.9%	0.077	0.016
1<br>2	A10BK	Sodium-glucose co-transporter 2 (SGLT2) inhibitors		1,978<br>1,699	7.7%<br>6.6%	<0.001	0.042
1<br>2	A10BX	Other blood glucose lowering drugs, excl. insulins		291<br>293	1.1%<br>1.1%	0.934	0.001
1<br>2	HS200	CONTRACEPTIVES,SYSTEMIC		1,299<br>1,190	5.0%<br>4.6%	0.025	0.020
1<br>2	10324	tamoxifen		37<br>24	0.1%<br>0.1%	0.096	0.015

© 2024 Wang L et al. *JAMA Network Open.*

eTable 5. Characteristics of the GLP-1RA/no insulin group and insulin/no GLP-1RA group before and after matched for covariates related to gallbladder cancer for the study populations of patients with T2D and no history of any OAC

Group 1 (N = 48,983) and group 2 (N = 1,044,745) characteristics before propensity score matching

Demographics

Group			Mean ± SD	Patients	% of Group	P-Value	SMD
1<br>2	AI	Age at Index	55.9 +/- 11.7<br>61.8 +/- 15.9	48,983<br>1,044,745	100%<br>100%	<0.001	0.420
1<br>2	2106-3	White		32,592<br>633,989	66.5%<br>60.7%	<0.001	0.122
1<br>2	UNK	Unknown Race		7,099<br>142,470	14.5%<br>13.6%	<0.001	0.025
1<br>2	F	Female		26,011<br>476,110	53.1%<br>45.6%	<0.001	0.151
1<br>2	UN	Unknown Gender		2,252<br>27,321	4.6%<br>2.6%	<0.001	0.106
1<br>2	2186-5	Not Hispanic or Latino		33,188<br>659,375	67.8%<br>63.1%	<0.001	0.098
1<br>2	2135-2	Hispanic or Latino		4,151<br>94,136	8.5%<br>9.0%	<0.001	0.019
1<br>2	2054-5	Black or African American		6,265<br>178,267	12.8%<br>17.1%	<0.001	0.120
1<br>2	M	Male		20,720<br>541,314	42.3%<br>51.8%	<0.001	0.191
1<br>2	2028-9	Asian		1,204<br>41,822	2.5%<br>4.0%	<0.001	0.087

Diagnosis

Group			Mean ± SD	Patients	% of Group	P-Value	SMD
1<br>2	Z55-Z65	Persons with potential health hazards related to socioeconomic and psychosocial circumstances		686<br>12,021	1.4%<br>1.2%	<0.001	0.022
1<br>2	E66	Overweight and obesity		18,401<br>166,445	37.6%<br>15.9%	<0.001	0.504

© 2024 Wang L et al. *JAMA Network Open.*

1<br>2	Z68.3	Body mass index [BMI] 30-39, adult	4,204<br>43,128	8.6%<br>4.1%	<0.001	0.183
1<br>2	Z68.4	Body mass index [BMI] 40 or greater, adult	3,886<br>33,396	7.9%<br>3.2%	<0.001	0.208
1<br>2	Z68.25	Body mass index [BMI] 25.0-25.9, adult	157<br>3,510	0.3%<br>0.3%	0.563	0.003
1<br>2	Z68.26	Body mass index [BMI] 26.0-26.9, adult	171<br>3,632	0.3%<br>0.3%	0.957	<0.001
1<br>2	Z68.27	Body mass index [BMI] 27.0-27.9, adult	288<br>4,209	0.6%<br>0.4%	<0.001	0.026
1<br>2	Z68.28	Body mass index [BMI] 28.0-28.9, adult	344<br>4,508	0.7%<br>0.4%	<0.001	0.036
1<br>2	Z68.29	Body mass index [BMI] 29.0-29.9, adult	398<br>4,843	0.8%<br>0.5%	<0.001	0.044
1<br>2	F17	Nicotine dependence	3,593<br>96,860	7.3%<br>9.3%	<0.001	0.070
1<br>2	E66.0	Obesity due to excess calories	9,157<br>69,998	18.7%<br>6.7%	<0.001	0.366
1<br>2	E66.2	Morbid (severe) obesity with alveolar hypoventilation	150<br>3,643	0.3%<br>0.3%	0.118	0.007
1<br>2	E66.3	Overweight	1,010<br>6,796	2.1%<br>0.7%	<0.001	0.122
1<br>2	E66.8	Other obesity	235<br>1,298	0.5%<br>0.1%	<0.001	0.065
1<br>2	E66.9	Obesity, unspecified	13,805<br>118,555	28.2%<br>11.3%	<0.001	0.433
1<br>2	Z72.0	Tobacco use	1,064<br>13,876	2.2%<br>1.3%	<0.001	0.064
1<br>2	Q44.5	Other congenital malformations of bile ducts	11<br>396	0.0%<br>0.0%	0.083	0.009
1<br>2	K82.4	Cholesterolosis of gallbladder	99<br>1,160	0.2%<br>0.1%	<0.001	0.023
1<br>2	K83.01	Primary sclerosing cholangitis	10<br>87	0.0%<br>0.0%	0.005	0.010

© 2024 Wang L et al. *JAMA Network Open.*

1<br>2	A01.0	Typhoid fever		10<br>23	0.0%<br>0.0%	<0.00<br>1	0.017
1<br>2	Z85.09	Personal history of malignant neoplasm of other digestive organs		10<br>256	0.0%<br>0.0%	0.571	0.003
1<br>2	D13.5	Benign neoplasm of extrahepatic bile ducts		27<br>472	0.1%<br>0.0%	0.314	0.004
**Medication**							
Group			Mean ± SD	Patients	% of Group	P-Value	SMD
1<br>2	A10B<br>A	Biguanides		27,075<br>199,802	55.3%<br>19.1%	<0.00<br>1	0.806
1<br>2	A10B<br>B	Sulfonylureas		14,077<br>125,703	28.7%<br>12.0%	<0.00<br>1	0.424
1<br>2	A10B<br>F	Alpha glucosidase inhibitors		229<br>1,718	0.5%<br>0.2%	<0.00<br>1	0.054
1<br>2	A10B<br>G	Thiazolidinediones		4,107<br>35,435	8.4%<br>3.4%	<0.00<br>1	0.213
1<br>2	A10B<br>H	Dipeptidyl peptidase 4 (DPP-4) inhibitors		9,485<br>44,595	19.4%<br>4.3%	<0.00<br>1	0.481
1<br>2	A10B<br>K	Sodium-glucose co-transporter 2 (SGLT2) inhibitors		4,808<br>6,447	9.8%<br>0.6%	<0.00<br>1	0.423
1<br>2	A10B<br>X	Other blood glucose lowering drugs, excl. insulins		614<br>7,048	1.3%<br>0.7%	<0.00<br>1	0.059
**Group 1 (N = 48,587) and group 2 (N = 48,587) characteristics after propensity score matching**							
**Demographics**							
Group			Mean ± SD	Patients	% of Group	P-Value	SMD
1<br>2	AI	Age at Index	55.9 +/- 11.8<br>56.1 +/- 12.6	48,587<br>48,587	100%<br>100%	0.005	0.018
1<br>2	2106-3	White		32,319<br>33,024	66.5%<br>68.0%	<0.00<br>1	0.031
1<br>2	UNK	Unknown Race		7,011<br>6,716	14.4%<br>13.8%	0.007	0.017
1<br>2	F	Female		25,779<br>25,775	53.1%<br>53.0%	0.979	<0.001

© 2024 Wang L et al. *JAMA Network Open.*

1<br>2	UN	Unknown Gender		2,186<br>2,139	4.5%<br>4.4%	0.465	0.005
1<br>2	2186-5	Not Hispanic or Latino		32,920<br>33,260	67.8%<br>68.5%	0.019	0.015
1<br>2	2135-2	Hispanic or Latino		4,126<br>3,972	8.5%<br>8.2%	0.074	0.011
1<br>2	2054-5	Black or African American		6,241<br>6,095	12.8%<br>12.5%	0.159	0.009
1<br>2	M	Male		20,622<br>20,673	42.4%<br>42.5%	0.741	0.002
1<br>2	2028-9	Asian		1,199<br>1,091	2.5%<br>2.2%	0.022	0.015

Diagnosis

Group			Mean ± SD	Patients	% of Group	P-Value	SMD
1<br>2	Z55-Z65	Persons with potential health hazards related to socioeconomic and psychosocial circumstances		682<br>596	1.4%<br>1.2%	0.015	0.016
	E66	Overweight and obesity		17,410<br>17,367	36.9%<br>36.8%	0.772	0.002
1<br>2	Z68.3	Body mass index [BMI] 30-39, adult		3,964<br>3,562	8.4%<br>7.5%	<0.001	0.031
1<br>2	Z68.4	Body mass index [BMI] 40 or greater, adult		3,642<br>3,272	7.7%<br>6.9%	<0.001	0.030
1<br>2	Z68.25	Body mass index [BMI] 25.0-25.9, adult		151<br>134	0.3%<br>0.3%	0.313	0.007
1<br>2	Z68.26	Body mass index [BMI] 26.0-26.9, adult		169<br>156	0.4%<br>0.3%	0.470	0.005
1<br>2	Z68.27	Body mass index [BMI] 27.0-27.9, adult		278<br>236	0.6%<br>0.5%	0.063	0.012
1<br>2	Z68.28	Body mass index [BMI] 28.0-28.9, adult		322<br>286	0.7%<br>0.6%	0.143	0.010

© 2024 Wang L et al. *JAMA Network Open.*

1<br>2	Z68.29	Body mass index [BMI] 29.0-29.9, adult		382<br>328	0.8%<br>0.7%	0.042	0.013
1<br>2	F17	Nicotine dependence		3,576<br>3,185	7.4%<br>6.6%	<0.001	0.032
1<br>2	E66.0	Obesity due to excess calories		8,635<br>8,523	18.3%<br>18.0%	0.345	0.006
1<br>2	E66.2	Morbid (severe) obesity with alveolar hypoventilation		139<br>134	0.3%<br>0.3%	0.762	0.002
1<br>2	E66.3	Overweight		941<br>866	2.0%<br>1.8%	0.075	0.012
1<br>2	E66.8	Other obesity		204<br>204	0.4%<br>0.4%	1	<0.001
1<br>2	E66.9	Obesity, unspecified		13,034<br>13,043	27.6%<br>27.6%	0.948	<0.001
1<br>2	Z72.0	Tobacco use		1,044<br>981	2.2%<br>2.0%	0.157	0.009
1<br>2	K82.4	Cholesterolosis of gallbladder		93<br>67	0.2%<br>0.1%	0.040	0.013
1<br>2	K83.01	Primary sclerosing cholangitis		10<br>10	0.0%<br>0.0%	1	<0.001
1<br>2	A01.0	Typhoid fever		10<br>10	0.0%<br>0.0%	1	<0.001
1<br>2	Z85.09	Personal history of malignant neoplasm of other digestive organs		10<br>10	0.0%<br>0.0%	1	<0.001
1<br>2	D13.5	Benign neoplasm of extrahepatic bile ducts		26<br>19	0.1%<br>0.0%	0.297	0.007

Medication

Group			Mean ± SD	Patients	% of Group	P-Value	SMD
1<br>2	A10BA	Biguanides		26,679<br>27,080	54.9%<br>55.7%	0.010	0.017
1<br>2	A10BB	Sulfonylureas		13,835<br>14,192	28.5%<br>29.2%	0.011	0.016
1<br>2	A10BF	Alpha glucosidase inhibitors		219<br>217	0.5%<br>0.4%	0.924	0.001
1<br>2	A10BG	Thiazolidinediones		4,045<br>4,099	8.3%<br>8.4%	0.532	0.004

1<br>2	A10B<br>H	Dipeptidyl peptidase 4 (DPP-4) inhibitors	9,167<br>9,239	18.9%<br>19.0%	0.556	0.004
1<br>2	A10B<br>K	Sodium-glucose co-transporter 2 (SGLT2) inhibitors	4,419<br>4,037	9.1%<br>8.3%	<0.00<br>1	0.028
1<br>2	A10B<br>X	Other blood glucose lowering drugs, excl. insulins	609<br>553	1.3%<br>1.1%	0.098	0.011

© 2024 Wang L et al. *JAMA Network Open.*

eTable 6. Characteristics of the GLP-1RA/no insulin group and insulin/no GLP-1RA group before and after matched for covariates related to stomach cancer for the study populations of patients with T2D and no history of any OAC

Group 1 (N = 48,983) and group 2 (N = 1,044,745) characteristics before propensity score matching

Demographics

Group			Mean ± SD	Patients	% of Group	P-Value	SMD
1<br>2	AI	Age at Index	55.9 +/- 11.7<br>61.8 +/- 15.9	48,983<br>1,044,745	100%<br>100%	<0.001	0.420
1<br>2	2106-3	White		32,592<br>633,989	66.5%<br>60.7%	<0.001	0.122
1<br>2	1002-5	American Indian or Alaska Native		199<br>3,443	0.4%<br>0.3%	0.004	0.013
1<br>2	UNK	Unknown Race		7,099<br>142,470	14.5%<br>13.6%	<0.001	0.025
1<br>2	F	Female		26,011<br>476,110	53.1%<br>45.6%	<0.001	0.151
1<br>2	2076-8	Native Hawaiian or Other Pacific Islander		205<br>10,677	0.4%<br>1.0%	<0.001	0.071
1<br>2	UN	Unknown Gender		2,252<br>27,321	4.6%<br>2.6%	<0.001	0.106
1<br>2	2186-5	Not Hispanic or Latino		33,188<br>659,375	67.8%<br>63.1%	<0.001	0.098
1<br>2	2135-2	Hispanic or Latino		4,151<br>94,136	8.5%<br>9.0%	<0.001	0.019
1<br>2	2054-5	Black or African American		6,265<br>178,267	12.8%<br>17.1%	<0.001	0.120
1<br>2	M	Male		20,720<br>541,314	42.3%<br>51.8%	<0.001	0.191
1<br>2	2028-9	Asian		1,204<br>41,822	2.5%<br>4.0%	<0.001	0.087

Diagnosis

Group			Mean ± SD	Patients	% of Group	P-Value	SMD
1<br>2	Z55-Z65	Persons with potential health hazards related to socioeconomic and		686<br>12,021	1.4%<br>1.2%	<0.001	0.022

© 2024 Wang L et al. *JAMA Network Open.*

		psychosocial circumstances				
1<br>2	E66	Overweight and obesity	18,401<br>166,445	37.6%<br>15.9%	<0.001	0.504
1<br>2	Z68.3	Body mass index [BMI] 30-39, adult	4,204<br>43,128	8.6%<br>4.1%	<0.001	0.183
1<br>2	Z68.4	Body mass index [BMI] 40 or greater, adult	3,886<br>33,396	7.9%<br>3.2%	<0.001	0.208
1<br>2	Z68.25	Body mass index [BMI] 25.0-25.9, adult	157<br>3,510	0.3%<br>0.3%	0.563	0.003
1<br>2	Z68.26	Body mass index [BMI] 26.0-26.9, adult	171<br>3,632	0.3%<br>0.3%	0.957	<0.001
1<br>2	Z68.27	Body mass index [BMI] 27.0-27.9, adult	288<br>4,209	0.6%<br>0.4%	<0.001	0.026
1<br>2	Z68.28	Body mass index [BMI] 28.0-28.9, adult	344<br>4,508	0.7%<br>0.4%	<0.001	0.036
1<br>2	Z68.29	Body mass index [BMI] 29.0-29.9, adult	398<br>4,843	0.8%<br>0.5%	<0.001	0.044
1<br>2	F10	Alcohol related disorders	563<br>28,538	1.1%<br>2.7%	<0.001	0.115
1<br>2	Z80	Family history of primary malignant neoplasm	2,042<br>19,398	4.2%<br>1.9%	<0.001	0.136
1<br>2	Z15.0	Genetic susceptibility to malignant neoplasm	24<br>156	0.0%<br>0.0%	<0.001	0.019
1<br>2	Z12	Encounter for screening for malignant neoplasms	12,272<br>105,217	25.1%<br>10.1%	<0.001	0.402
1<br>2	Z85	Personal history of malignant neoplasm	1,239<br>38,294	2.5%<br>3.7%	<0.001	0.066
1<br>2	Z98.84	Bariatric surgery status	632<br>5,136	1.3%<br>0.5%	<0.001	0.085
1<br>2	F17	Nicotine dependence	3,593<br>96,860	7.3%<br>9.3%	<0.001	0.070

© 2024 Wang L et al. *JAMA Network Open.*

1<br>2	E66.0	Obesity due to excess calories		9,157<br>69,998	18.7%<br>6.7%	<0.001	0.366
1<br>2	E66.2	Morbid (severe) obesity with alveolar hypoventilation		150<br>3,643	0.3%<br>0.3%	0.118	0.007
1<br>2	E66.3	Overweight		1,010<br>6,796	2.1%<br>0.7%	<0.001	0.122
1<br>2	E66.8	Other obesity		235<br>1,298	0.5%<br>0.1%	<0.001	0.065
1<br>2	E66.9	Obesity, unspecified		13,805<br>118,555	28.2%<br>11.3%	<0.001	0.433
1<br>2	Z72.0	Tobacco use		1,064<br>13,876	2.2%<br>1.3%	<0.001	0.064
1<br>2	Z85.09	Personal history of malignant neoplasm of other digestive organs		10<br>256	0.0%<br>0.0%	0.571	0.003
1<br>2	B96.81	Helicobacter pylori [H. pylori] as the cause of diseases classified elsewhere		254<br>3,973	0.5%<br>0.4%	<0.001	0.021
1<br>2	D13.1	Benign neoplasm of stomach		213<br>3,086	0.4%<br>0.3%	<0.001	0.023
1<br>2	K31.7	Polyp of stomach and duodenum		266<br>3,728	0.5%<br>0.4%	<0.001	0.028
1<br>2	D51.0	Vitamin B12 deficiency anemia due to intrinsic factor deficiency		143<br>2,101	0.3%<br>0.2%	<0.001	0.018
1<br>2	Z80.0	Family history of malignant neoplasm of digestive organs		784<br>6,889	1.6%<br>0.7%	<0.001	0.089
1<br>2	D83	Common variable immunodeficiency		19<br>356	0.0%<br>0.0%	0.582	0.002
1<br>2	B27	Infectious mononucleosis		45<br>513	0.1%<br>0.0%	<0.001	0.016
**Procedure**							
Group			Mean ± SD	Patients	% of Group	P-Value	SMD
1<br>2	1007352	Excision Procedures on the Stomach		10<br>115	0.0%<br>0.0%	0.057	0.008
**Medication**							

© 2024 Wang L et al. *JAMA Network Open.*

Group			Mean ± SD	Patients	% of Group	P-Value	SMD
1<br>2	A10BA	Biguanides		27,075<br>199,802	55.3%<br>19.1%	<0.001	0.806
1<br>2	A10BB	Sulfonylureas		14,077<br>125,703	28.7%<br>12.0%	<0.001	0.424
1<br>2	A10BF	Alpha glucosidase inhibitors		229<br>1,718	0.5%<br>0.2%	<0.001	0.054
1<br>2	A10BG	Thiazolidinediones		4,107<br>35,435	8.4%<br>3.4%	<0.001	0.213
1<br>2	A10BH	Dipeptidyl peptidase 4 (DPP-4) inhibitors		9,485<br>44,595	19.4%<br>4.3%	<0.001	0.481
1<br>2	A10BK	Sodium-glucose co-transporter 2 (SGLT2) inhibitors		4,808<br>6,447	9.8%<br>0.6%	<0.001	0.423
1<br>2	A10BX	Other blood glucose lowering drugs, excl. insulins		614<br>7,048	1.3%<br>0.7%	<0.001	0.059

Group 1 (N = 48,449) and group 2 (N = 48,449) characteristics after propensity score matching

Demographics

Group			Mean ± SD	Patients	% of Group	P-Value	SMD
1<br>2	AI	Age at Index	55.9 +/- 11.7<br>56.1 +/- 13.2	48,449<br>48,449	100%<br>100%	0.030	0.014
1<br>2	2106-3	White		32,245<br>33,198	66.6%<br>68.5%	<0.001	0.042
1<br>2	1002-5	American Indian or Alaska Native		196<br>164	0.4%<br>0.3%	0.091	0.011
1<br>2	UNK	Unknown Race		6,980<br>6,650	14.4%<br>13.7%	0.002	0.020
1<br>2	F	Female		25,712<br>26,156	53.1%<br>54.0%	0.004	0.018
1<br>2	2076-8	Native Hawaiian or Other Pacific Islander		204<br>156	0.4%<br>0.3%	0.011	0.016
1<br>2	UN	Unknown Gender		2,170<br>2,038	4.5%<br>4.2%	0.037	0.013
1<br>2	2186-5	Not Hispanic or Latino		32,837<br>33,141	67.8%<br>68.4%	0.036	0.013

© 2024 Wang L et al. *JAMA Network Open.*

1<br>2	2135-2	Hispanic or Latino		4,114<br>4,059	8.5%<br>8.4%	0.525	0.004
1<br>2	2054-5	Black or African American		6,222<br>5,889	12.8%<br>12.2%	0.001	0.021
1<br>2	M	Male		20,567<br>20,255	42.5%<br>41.8%	0.042	0.013
1<br>2	2028-9	Asian		1,196<br>1,055	2.5%<br>2.2%	0.003	0.019
**Diagnosis**							
Group			Mean ± SD	Patients	% of Group	P-Value	SMD
1<br>2	Z55-Z65	Persons with potential health hazards related to socioeconomic and psychosocial circumstances		676<br>574	1.4%<br>1.2%	0.004	0.019
1<br>2	E66	Overweight and obesity		17,989<br>17,978	37.1%<br>37.1%	0.942	<0.001
1<br>2	Z68.3	Body mass index [BMI] 30-39, adult		4,050<br>3,670	8.4%<br>7.6%	<0.001	0.029
1<br>2	Z68.4	Body mass index [BMI] 40 or greater, adult		3,782<br>3,380	7.8%<br>7.0%	<0.001	0.032
1<br>2	Z68.25	Body mass index [BMI] 25.0-25.9, adult		156<br>138	0.3%<br>0.3%	0.293	0.007
1<br>2	Z68.26	Body mass index [BMI] 26.0-26.9, adult		171<br>154	0.4%<br>0.3%	0.345	0.006
1<br>2	Z68.27	Body mass index [BMI] 27.0-27.9, adult		282<br>229	0.6%<br>0.5%	0.019	0.015
1<br>2	Z68.28	Body mass index [BMI] 28.0-28.9, adult		332<br>306	0.7%<br>0.6%	0.302	0.007
1<br>2	Z68.29	Body mass index [BMI] 29.0-29.9, adult		386<br>327	0.8%<br>0.7%	0.027	0.014
1<br>2	F10	Alcohol related disorders		563<br>487	1.2%<br>1.0%	0.018	0.015

1<br>2	Z80	Family history of primary malignant neoplasm	1,992<br>1,834	4.1%<br>3.8%	0.009	0.017
1<br>2	Z15.0	Genetic susceptibility to malignant neoplasm	22<br>18	0.0%<br>0.0%	0.527	0.004
1<br>2	Z12	Encounter for screening for malignant neoplasms	11,930<br>11,522	24.6%<br>23.8%	0.002	0.020
1<br>2	Z85	Personal history of malignant neoplasm	1,232<br>1,090	2.5%<br>2.2%	0.003	0.019
1<br>2	Z98.84	Bariatric surgery status	614<br>605	1.3%<br>1.2%	0.795	0.002
1<br>2	F17	Nicotine dependence	3,576<br>3,185	7.4%<br>6.6%	<0.00<br>1	0.032
1<br>2	E66.0	Obesity due to excess calories	8,912<br>8,907	18.4%<br>18.4%	0.967	<0.001
1<br>2	E66.2	Morbid (severe) obesity with alveolar hypoventilation	148<br>142	0.3%<br>0.3%	0.724	0.002
1<br>2	E66.3	Overweight	976<br>888	2.0%<br>1.8%	0.040	0.013
1<br>2	E66.8	Other obesity	220<br>197	0.5%<br>0.4%	0.259	0.007
1<br>2	E66.9	Obesity, unspecified	13,470<br>13,494	27.8%<br>27.9%	0.863	0.001
1<br>2	Z72.0	Tobacco use	1,044<br>981	2.2%<br>2.0%	0.157	0.009
1<br>2	Z85.09	Personal history of malignant neoplasm of other digestive organs	10<br>10	0.0%<br>0.0%	1	<0.001
1<br>2	B96.8<br>1	Helicobacter pylori [H. pylori] as the cause of diseases classified elsewhere	253<br>228	0.5%<br>0.5%	0.253	0.007
1<br>2	D13.1	Benign neoplasm of stomach	209<br>169	0.4%<br>0.3%	0.039	0.013
1<br>2	K31.7	Polyp of stomach and duodenum	259<br>236	0.5%<br>0.5%	0.300	0.007
1<br>2	D51.0	Vitamin B12 deficiency anemia	139<br>112	0.3%<br>0.2%	0.088	0.011

© 2024 Wang L et al. *JAMA Network Open.*

		due to intrinsic factor deficiency					
1<br>2	Z80.0	Family history of malignant neoplasm of digestive organs		763<br>688	1.6%<br>1.4%	0.047	0.013
1<br>2	D83	Common variable immunodeficiency		18<br>15	0.0%<br>0.0%	0.601	0.003
1<br>2	B27	Infectious mononucleosis		44<br>47	0.1%<br>0.1%	0.753	0.002
**Procedure**							
Group			Mean ± SD	Patients	% of Group	P-Value	SMD
1<br>2	10073<br>52	Excision Procedures on the Stomach		10<br>10	0.0%<br>0.0%	1	<0.001
**Medication**							
Group			Mean ± SD	Patients	% of Group	P-Value	SMD
1<br>2	A10B<br>A	Biguanides		26,543<br>26,933	54.8%<br>55.6%	0.012	0.016
1<br>2	A10B<br>B	Sulfonylureas		13,757<br>14,099	28.4%<br>29.1%	0.015	0.016
1<br>2	A10B<br>F	Alpha glucosidase inhibitors		222<br>192	0.5%<br>0.4%	0.140	0.009
1<br>2	A10B<br>G	Thiazolidinediones		4,018<br>4,115	8.3%<br>8.5%	0.261	0.007
1<br>2	A10B<br>H	Dipeptidyl peptidase 4 (DPP-4) inhibitors		9,089<br>9,203	18.8%<br>19.0%	0.349	0.006
1<br>2	A10B<br>K	Sodium-glucose co-transporter 2 (SGLT2) inhibitors		4,303<br>3,823	8.9%<br>7.9%	<0.001	0.036
1<br>2	A10B<br>X	Other blood glucose lowering drugs, excl. insulins		606<br>583	1.3%<br>1.2%	0.502	0.004

© 2024 Wang L et al. *JAMA Network Open.*

eTable 7. Characteristics of the GLP-1RA/no insulin group and insulin/no GLP-1RA group before and after matched for covariates related to kidney cancer for the study populations of patients with T2D and no history of any OAC

Group 1 (N = 48,983) and group 2 (N = 1,044,745) characteristics before propensity score matching							
**Demographics**							
Group			Mean ± SD	Patients	% of Group	P-Value	SMD
1<br>2	AI	Age at Index	55.9 +/- 11.7<br>61.8 +/- 15.9	48,983<br>1,044,745	100%<br>100%	<0.001	0.420
1<br>2	2106-3	White		32,592<br>633,989	66.5%<br>60.7%	<0.001	0.122
1<br>2	1002-5	American Indian or Alaska Native		199<br>3,443	0.4%<br>0.3%	0.004	0.013
1<br>2	UNK	Unknown Race		7,099<br>142,470	14.5%<br>13.6%	<0.001	0.025
1<br>2	F	Female		26,011<br>476,110	53.1%<br>45.6%	<0.001	0.151
1<br>2	2076-8	Native Hawaiian or Other Pacific Islander		205<br>10,677	0.4%<br>1.0%	<0.001	0.071
1<br>2	UN	Unknown Gender		2,252<br>27,321	4.6%<br>2.6%	<0.001	0.106
1<br>2	2186-5	Not Hispanic or Latino		33,188<br>659,375	67.8%<br>63.1%	<0.001	0.098
1<br>2	2135-2	Hispanic or Latino		4,151<br>94,136	8.5%<br>9.0%	<0.001	0.019
1<br>2	2054-5	Black or African American		6,265<br>178,267	12.8%<br>17.1%	<0.001	0.120
1<br>2	M	Male		20,720<br>541,314	42.3%<br>51.8%	<0.001	0.191
1<br>2	2028-9	Asian		1,204<br>41,822	2.5%<br>4.0%	<0.001	0.087
**Diagnosis**							
Group			Mean ± SD	Patients	% of Group	P-Value	SMD
1<br>2	Z55-Z65	Persons with potential health hazards related to socioeconomic and		686<br>12,021	1.4%<br>1.2%	<0.001	0.022

© 2024 Wang L et al. *JAMA Network Open.*

		psychosocial circumstances				
1<br>2	E66	Overweight and obesity	18,401<br>166,445	37.6%<br>15.9%	<0.001	0.504
1<br>2	Z68.3	Body mass index [BMI] 30-39, adult	4,204<br>43,128	8.6%<br>4.1%	<0.001	0.183
1<br>2	Z68.4	Body mass index [BMI] 40 or greater, adult	3,886<br>33,396	7.9%<br>3.2%	<0.001	0.208
1<br>2	Z68.25	Body mass index [BMI] 25.0-25.9, adult	157<br>3,510	0.3%<br>0.3%	0.563	0.003
1<br>2	Z68.26	Body mass index [BMI] 26.0-26.9, adult	171<br>3,632	0.3%<br>0.3%	0.957	<0.001
1<br>2	Z68.27	Body mass index [BMI] 27.0-27.9, adult	288<br>4,209	0.6%<br>0.4%	<0.001	0.026
1<br>2	Z68.28	Body mass index [BMI] 28.0-28.9, adult	344<br>4,508	0.7%<br>0.4%	<0.001	0.036
1<br>2	Z68.29	Body mass index [BMI] 29.0-29.9, adult	398<br>4,843	0.8%<br>0.5%	<0.001	0.044
1<br>2	Z80	Family history of primary malignant neoplasm	2,042<br>19,398	4.2%<br>1.9%	<0.001	0.136
1<br>2	Z15.0	Genetic susceptibility to malignant neoplasm	24<br>156	0.0%<br>0.0%	<0.001	0.019
1<br>2	Z12	Encounter for screening for malignant neoplasms	12,272<br>105,217	25.1%<br>10.1%	<0.001	0.402
1<br>2	Z85	Personal history of malignant neoplasm	1,239<br>38,294	2.5%<br>3.7%	<0.001	0.066
1<br>2	Z98.84	Bariatric surgery status	632<br>5,136	1.3%<br>0.5%	<0.001	0.085
1<br>2	I10	Essential (primary) hypertension	29,049<br>506,936	59.3%<br>48.5%	<0.001	0.218
1<br>2	F17	Nicotine dependence	3,593<br>96,860	7.3%<br>9.3%	<0.001	0.070

© 2024 Wang L et al. *JAMA Network Open.*

1<br>2	E66.0	Obesity due to excess calories		9,157<br>69,998	18.7%<br>6.7%	<0.001	0.366
1<br>2	E66.2	Morbid (severe) obesity with alveolar hypoventilation		150<br>3,643	0.3%<br>0.3%	0.118	0.007
1<br>2	E66.3	Overweight		1,010<br>6,796	2.1%<br>0.7%	<0.001	0.122
1<br>2	E66.8	Other obesity		235<br>1,298	0.5%<br>0.1%	<0.001	0.065
1<br>2	E66.9	Obesity, unspecified		13,805<br>118,555	28.2%<br>11.3%	<0.001	0.433
1<br>2	Z72.0	Tobacco use		1,064<br>13,876	2.2%<br>1.3%	<0.001	0.064
1<br>2	Z80.51	Family history of malignant neoplasm of kidney		18<br>207	0.0%<br>0.0%	0.011	0.010
1<br>2	N18	Chronic kidney disease (CKD)		2,631<br>143,501	5.4%<br>13.7%	<0.001	0.287
1<br>2	N19	Unspecified kidney failure		98<br>13,506	0.2%<br>1.3%	<0.001	0.127
1<br>2	Z99.2	Dependence on renal dialysis		40<br>17,914	0.1%<br>1.7%	<0.001	0.174
1<br>2	N17-N19	Acute kidney failure and chronic kidney disease		3,016<br>187,587	6.2%<br>18.0%	<0.001	0.368
1<br>2	Q85.83	Von Hippel-Lindau syndrome		0<br>10	0%<br>0.0%	0.494	0.004
1<br>2	Q85.1	Tuberous sclerosis		10<br>30	0.0%<br>0.0%	<0.001	0.016
1<br>2	D57.1	Sickle-cell disease without crisis		10<br>516	0.0%<br>0.0%	0.004	0.016
1<br>2	D30.0	Benign neoplasm of kidney		31<br>451	0.1%<br>0.0%	0.038	0.009
**Medication**							
Group			Mean ± SD	Patients	% of Group	P-Value	SMD
1<br>2	A10BA	Biguanides		27,075<br>199,802	55.3%<br>19.1%	<0.001	0.806
1<br>2	A10BB	Sulfonylureas		14,077<br>125,703	28.7%<br>12.0%	<0.001	0.424

© 2024 Wang L et al. *JAMA Network Open.*

1<br>2	A10BF	Alpha glucosidase inhibitors		229<br>1,718	0.5%<br>0.2%	<0.001	0.054
1<br>2	A10BG	Thiazolidinediones		4,107<br>35,435	8.4%<br>3.4%	<0.001	0.213
1<br>2	A10BH	Dipeptidyl peptidase 4 (DPP-4) inhibitors		9,485<br>44,595	19.4%<br>4.3%	<0.001	0.481
1<br>2	A10BK	Sodium-glucose co-transporter 2 (SGLT2) inhibitors		4,808<br>6,447	9.8%<br>0.6%	<0.001	0.423
1<br>2	A10BX	Other blood glucose lowering drugs, excl. insulins		614<br>7,048	1.3%<br>0.7%	<0.001	0.059
1<br>2	161	acetaminophen		13,013<br>309,279	26.6%<br>29.6%	<0.001	0.068

Group 1 (N = 48,322) and group 2 (N = 48,322) characteristics after propensity score matching

Demographics

Group			Mean ± SD	Patients	% of Group	P-Value	SMD
1<br>2	AI	Age at Index	55.9 +/- 11.8<br>56.3 +/- 13.7	48,322<br>48,322	100%<br>100%	<0.001	0.026
1<br>2	2106-3	White		32,173<br>33,049	66.6%<br>68.4%	<0.001	0.039
1<br>2	1002-5	American Indian or Alaska Native		197<br>197	0.4%<br>0.4%	1	<0.001
1<br>2	UNK	Unknown Race		6,946<br>6,604	14.4%<br>13.7%	0.002	0.020
1<br>2	F	Female		25,662<br>26,111	53.1%<br>54.0%	0.004	0.019
1<br>2	2076-8	Native Hawaiian or Other Pacific Islander		203<br>157	0.4%<br>0.3%	0.015	0.016
1<br>2	UN	Unknown Gender		2,143<br>2,013	4.4%<br>4.2%	0.039	0.013
1<br>2	2186-5	Not Hispanic or Latino		32,743<br>33,107	67.8%<br>68.5%	0.012	0.016
1<br>2	2135-2	Hispanic or Latino		4,109<br>4,041	8.5%<br>8.4%	0.431	0.005
1<br>2	2054-5	Black or African American		6,204<br>5,901	12.8%<br>12.2%	0.003	0.019

© 2024 Wang L et al. *JAMA Network Open.*

1<br>2	M	Male		20,517<br>20,198	42.5%<br>41.8%	0.038	0.013
1<br>2	2028-9	Asian		1,193<br>1,036	2.5%<br>2.1%	0.001	0.022

Diagnosis

Group			Mean ± SD	Patients	% of Group	P-Value	SMD
1<br>2	Z55-Z65	Persons with potential health hazards related to socioeconomic and psychosocial circumstances		675<br>605	1.4%<br>1.3%	0.049	0.013
1<br>2	E66	Overweight and obesity		17,884<br>17,874	37.0%<br>37.0%	0.947	<0.001
1<br>2	Z68.3	Body mass index [BMI] 30-39, adult		4,013<br>3,732	8.3%<br>7.7%	0.001	0.021
1<br>2	Z68.4	Body mass index [BMI] 40 or greater, adult		3,743<br>3,394	7.7%<br>7.0%	<0.001	0.028
1<br>2	Z68.25	Body mass index [BMI] 25.0-25.9, adult		155<br>138	0.3%<br>0.3%	0.320	0.006
1<br>2	Z68.26	Body mass index [BMI] 26.0-26.9, adult		170<br>145	0.4%<br>0.3%	0.158	0.009
1<br>2	Z68.27	Body mass index [BMI] 27.0-27.9, adult		278<br>238	0.6%<br>0.5%	0.077	0.011
1<br>2	Z68.28	Body mass index [BMI] 28.0-28.9, adult		327<br>328	0.7%<br>0.7%	0.969	<0.001
1<br>2	Z68.29	Body mass index [BMI] 29.0-29.9, adult		382<br>346	0.8%<br>0.7%	0.180	0.009
1<br>2	Z80	Family history of primary malignant neoplasm		1,969<br>1,862	4.1%<br>3.9%	0.078	0.011
1<br>2	Z15.0	Genetic susceptibility to malignant neoplasm		22<br>15	0.0%<br>0.0%	0.250	0.007

© 2024 Wang L et al. *JAMA Network Open.*

1<br>2	Z12	Encounter for screening for malignant neoplasms	11,859<br>11,466	24.5%<br>23.7%	0.003	0.019
1<br>2	Z85	Personal history of malignant neoplasm	1,223<br>1,100	2.5%<br>2.3%	0.010	0.017
1<br>2	Z98.84	Bariatric surgery status	615<br>590	1.3%<br>1.2%	0.469	0.005
1<br>2	I10	Essential (primary) hypertension	28,525<br>28,455	59.0%<br>58.9%	0.647	0.003
1<br>2	F17	Nicotine dependence	3,567<br>3,184	7.4%<br>6.6%	<0.001	0.031
1<br>2	E66.0	Obesity due to excess calories	8,844<br>8,667	18.3%<br>17.9%	0.139	0.010
1<br>2	E66.2	Morbid (severe) obesity with alveolar hypoventilation	145<br>128	0.3%<br>0.3%	0.303	0.007
1<br>2	E66.3	Overweight	966<br>857	2.0%<br>1.8%	0.010	0.017
1<br>2	E66.8	Other obesity	224<br>195	0.5%<br>0.4%	0.156	0.009
1<br>2	E66.9	Obesity, unspecified	13,394<br>13,573	27.7%<br>28.1%	0.199	0.008
1<br>2	Z72.0	Tobacco use	1,042<br>932	2.2%<br>1.9%	0.012	0.016
1<br>2	Z80.51	Family history of malignant neoplasm of kidney	17<br>15	0.0%<br>0.0%	0.724	0.002
1<br>2	N18	Chronic kidney disease (CKD)	2,624<br>2,256	5.4%<br>4.7%	<0.001	0.035
1<br>2	N19	Unspecified kidney failure	98<br>73	0.2%<br>0.2%	0.056	0.012
1<br>2	Z99.2	Dependence on renal dialysis	40<br>35	0.1%<br>0.1%	0.564	0.004
1<br>2	N17-N19	Acute kidney failure and chronic kidney disease	3,009<br>2,548	6.2%<br>5.3%	<0.001	0.041
1<br>2	Q85.83	Von Hippel-Lindau syndrome	0<br>0	0%<br>0%	--	--
1<br>2	Q85.1	Tuberous sclerosis	10<br>10	0.0%<br>0.0%	1	<0.001
1<br>2	D57.1	Sickle-cell disease without crisis	10<br>11	0.0%<br>0.0%	0.827	0.001

© 2024 Wang L et al. *JAMA Network Open.*

1<br>2	D30.0	Benign neoplasm of kidney		31<br>23	0.1%<br>0.0%	0.276	0.007
**Medication**							
Group			Mean ± SD	Patients	% of Group	P-Value	SMD
1<br>2	A10B<br>A	Biguanides		26,421<br>26,729	54.7%<br>55.3%	0.046	0.013
1<br>2	A10B<br>B	Sulfonylureas		13,668<br>14,012	28.3%<br>29.0%	0.014	0.016
1<br>2	A10B<br>F	Alpha glucosidase inhibitors		220<br>200	0.5%<br>0.4%	0.328	0.006
1<br>2	A10B<br>G	Thiazolidinediones		4,005<br>4,030	8.3%<br>8.3%	0.771	0.002
1<br>2	A10B<br>H	Dipeptidyl peptidase 4 (DPP-4) inhibitors		9,006<br>8,953	18.6%<br>18.5%	0.661	0.003
1<br>2	A10B<br>K	Sodium-glucose co-transporter 2 (SGLT2) inhibitors		4,203<br>3,583	8.7%<br>7.4%	<0.001	0.047
1<br>2	A10B<br>X	Other blood glucose lowering drugs, excl. insulins		603<br>606	1.2%<br>1.3%	0.931	0.001
1<br>2	161	acetaminophen		12,902<br>12,334	26.7%<br>25.5%	<0.001	0.027

© 2024 Wang L et al. *JAMA Network Open.*

eTable 8. Characteristics of the GLP-1RA/no insulin group and insulin/no GLP-1RA group before and after matched for covariates related to liver cancer for the study populations of patients with T2D and no history of any OAC

Group 1 (N = 48,983) and group 2 (N = 1,044,745) characteristics before propensity score matching

Demographics

Group			Mean ± SD	Patients	% of Group	P-Value	SMD
1<br>2	AI	Age at Index	55.9 +/- 11.7<br>61.8 +/- 15.9	48,983<br>1,044,745	100%<br>100%	<0.001	0.420
1<br>2	2106-3	White		32,592<br>633,989	66.5%<br>60.7%	<0.001	0.122
1<br>2	1002-5	American Indian or Alaska Native		199<br>3,443	0.4%<br>0.3%	0.004	0.013
1<br>2	UNK	Unknown Race		7,099<br>142,470	14.5%<br>13.6%	<0.001	0.025
1<br>2	F	Female		26,011<br>476,110	53.1%<br>45.6%	<0.001	0.151
1<br>2	2076-8	Native Hawaiian or Other Pacific Islander		205<br>10,677	0.4%<br>1.0%	<0.001	0.071
1<br>2	UN	Unknown Gender		2,252<br>27,321	4.6%<br>2.6%	<0.001	0.106
1<br>2	2186-5	Not Hispanic or Latino		33,188<br>659,375	67.8%<br>63.1%	<0.001	0.098
1<br>2	2135-2	Hispanic or Latino		4,151<br>94,136	8.5%<br>9.0%	<0.001	0.019
1<br>2	2054-5	Black or African American		6,265<br>178,267	12.8%<br>17.1%	<0.001	0.120
1<br>2	M	Male		20,720<br>541,314	42.3%<br>51.8%	<0.001	0.191
1<br>2	2028-9	Asian		1,204<br>41,822	2.5%<br>4.0%	<0.001	0.087

Diagnosis

Group			Mean ± SD	Patients	% of Group	P-Value	SMD
1<br>2	Z55-Z65	Persons with potential health hazards related to socioeconomic and		686<br>12,021	1.4%<br>1.2%	<0.001	0.022

© 2024 Wang L et al. *JAMA Network Open.*

		psychosocial circumstances				
1<br>2	E66	Overweight and obesity	18,401<br>166,445	37.6%<br>15.9%	<0.001	0.504
1<br>2	Z68.3	Body mass index [BMI] 30-39, adult	4,204<br>43,128	8.6%<br>4.1%	<0.001	0.183
1<br>2	Z68.4	Body mass index [BMI] 40 or greater, adult	3,886<br>33,396	7.9%<br>3.2%	<0.001	0.208
1<br>2	Z68.25	Body mass index [BMI] 25.0-25.9, adult	157<br>3,510	0.3%<br>0.3%	0.563	0.003
1<br>2	Z68.26	Body mass index [BMI] 26.0-26.9, adult	171<br>3,632	0.3%<br>0.3%	0.957	<0.001
1<br>2	Z68.27	Body mass index [BMI] 27.0-27.9, adult	288<br>4,209	0.6%<br>0.4%	<0.001	0.026
1<br>2	Z68.28	Body mass index [BMI] 28.0-28.9, adult	344<br>4,508	0.7%<br>0.4%	<0.001	0.036
1<br>2	Z68.29	Body mass index [BMI] 29.0-29.9, adult	398<br>4,843	0.8%<br>0.5%	<0.001	0.044
1<br>2	F10	Alcohol related disorders	563<br>28,538	1.1%<br>2.7%	<0.001	0.115
1<br>2	Z80	Family history of primary malignant neoplasm	2,042<br>19,398	4.2%<br>1.9%	<0.001	0.136
1<br>2	Z15.0	Genetic susceptibility to malignant neoplasm	24<br>156	0.0%<br>0.0%	<0.001	0.019
1<br>2	Z12	Encounter for screening for malignant neoplasms	12,272<br>105,217	25.1%<br>10.1%	<0.001	0.402
1<br>2	Z85	Personal history of malignant neoplasm	1,239<br>38,294	2.5%<br>3.7%	<0.001	0.066
1<br>2	Z98.84	Bariatric surgery status	632<br>5,136	1.3%<br>0.5%	<0.001	0.085
1<br>2	E88.81	Metabolic syndrome and other insulin resistance	1,536<br>6,923	3.1%<br>0.7%	<0.001	0.182

© 2024 Wang L et al. *JAMA Network Open.*

1<br>2	E78	Disorders of lipoprotein metabolism and other lipidemias	28,438<br>399,702	58.1%<br>38.3%	<0.001	0.404
1<br>2	I10	Essential (primary) hypertension	29,049<br>506,936	59.3%<br>48.5%	<0.001	0.218
1<br>2	K75.81	Nonalcoholic steatohepatitis (NASH)	475<br>3,277	1.0%<br>0.3%	<0.001	0.082
1<br>2	F17	Nicotine dependence	3,593<br>96,860	7.3%<br>9.3%	<0.001	0.070
1<br>2	E66.0	Obesity due to excess calories	9,157<br>69,998	18.7%<br>6.7%	<0.001	0.366
1<br>2	E66.2	Morbid (severe) obesity with alveolar hypoventilation	150<br>3,643	0.3%<br>0.3%	0.118	0.007
1<br>2	E66.3	Overweight	1,010<br>6,796	2.1%<br>0.7%	<0.001	0.122
1<br>2	E66.8	Other obesity	235<br>1,298	0.5%<br>0.1%	<0.001	0.065
1<br>2	E66.9	Obesity, unspecified	13,805<br>118,555	28.2%<br>11.3%	<0.001	0.433
1<br>2	Z72.0	Tobacco use	1,064<br>13,876	2.2%<br>1.3%	<0.001	0.064
1<br>2	B18	Chronic viral hepatitis	260<br>12,879	0.5%<br>1.2%	<0.001	0.075
1<br>2	K74	Fibrosis and cirrhosis of liver	433<br>19,545	0.9%<br>1.9%	<0.001	0.085
1<br>2	K76.0	Fatty (change of) liver, not elsewhere classified	3,131<br>23,105	6.4%<br>2.2%	<0.001	0.207
1<br>2	K74.3	Primary biliary cirrhosis	20<br>786	0.0%<br>0.1%	0.006	0.014
1<br>2	E83.110	Hereditary hemochromatosis	40<br>251	0.1%<br>0.0%	<0.001	0.025
1<br>2	E70.21	Tyrosinemia	10<br>56	0.0%<br>0.0%	<0.001	0.013
1<br>2	E88.01	Alpha-1-antitrypsin deficiency	16<br>245	0.0%<br>0.0%	0.197	0.006
1<br>2	E80.1	Porphyria cutanea tarda	10<br>27	0.0%<br>0.0%	<0.001	0.017

© 2024 Wang L et al. *JAMA Network Open.*

Group			Mean ± SD	Patients	% of Group	P-Value	SMD
1<br>2	E74.0	Glycogen storage disease		10<br>144	0.0%<br>0.0%	0.227	0.005
1<br>2	E83.00	Disorder of copper metabolism, unspecified		10<br>99	0.0%<br>0.0%	0.018	0.009
1<br>2	T64	Toxic effect of aflatoxin and other mycotoxin food contaminants		10<br>176	0.0%<br>0.0%	0.554	0.003
1<br>2	D13.4	Benign neoplasm of liver		35<br>434	0.1%<br>0.0%	0.002	0.013

Medication

Group			Mean ± SD	Patients	% of Group	P-Value	SMD
1<br>2	A10BA	Biguanides		27,075<br>199,802	55.3%<br>19.1%	<0.001	0.806
1<br>2	A10BB	Sulfonylureas		14,077<br>125,703	28.7%<br>12.0%	<0.001	0.424
1<br>2	A10BF	Alpha glucosidase inhibitors		229<br>1,718	0.5%<br>0.2%	<0.001	0.054
1<br>2	A10BG	Thiazolidinediones		4,107<br>35,435	8.4%<br>3.4%	<0.001	0.213
1<br>2	A10BH	Dipeptidyl peptidase 4 (DPP-4) inhibitors		9,485<br>44,595	19.4%<br>4.3%	<0.001	0.481
1<br>2	A10BK	Sodium-glucose co-transporter 2 (SGLT2) inhibitors		4,808<br>6,447	9.8%<br>0.6%	<0.001	0.423
1<br>2	A10BX	Other blood glucose lowering drugs, excl. insulins		614<br>7,048	1.3%<br>0.7%	<0.001	0.059
1<br>2	A14A	ANABOLIC STEROIDS		60<br>379	0.1%<br>0.0%	<0.001	0.031

Group 1 (N = 48,397) and group 2 (N = 48,397) characteristics after propensity score matching

Demographics

Group			Mean ± SD	Patients	% of Group	P-Value	SMD
1<br>2	AI	Age at Index	55.9 +/- 11.7<br>56.2 +/- 13.7	48,397<br>48,397	100%<br>100%	0.004	0.018
1<br>2	2106-3	White		32,208<br>33,287	66.5%<br>68.8%	<0.001	0.048

© 2024 Wang L et al. *JAMA Network Open.*

1<br>2	1002-5	American Indian or Alaska Native		196<br>171	0.4%<br>0.4%	0.191	0.008
1<br>2	UNK	Unknown Race		6,970<br>6,443	14.4%<br>13.3%	<0.001	0.032
1<br>2	F	Female		25,674<br>26,256	53.0%<br>54.3%	<0.001	0.024
1<br>2	2076-8	Native Hawaiian or Other Pacific Islander		204<br>171	0.4%<br>0.4%	0.088	0.011
1<br>2	UN	Unknown Gender		2,164<br>1,881	4.5%<br>3.9%	<0.001	0.029
1<br>2	2186-5	Not Hispanic or Latino		32,793<br>33,299	67.8%<br>68.8%	<0.001	0.022
1<br>2	2135-2	Hispanic or Latino		4,114<br>3,939	8.5%<br>8.1%	0.042	0.013
1<br>2	2054-5	Black or African American		6,219<br>5,991	12.8%<br>12.4%	0.027	0.014
1<br>2	M	Male		20,559<br>20,260	42.5%<br>41.9%	0.052	0.013
1<br>2	2028-9	Asian		1,193<br>1,012	2.5%<br>2.1%	<0.001	0.025

Diagnosis

Group			Mean ± SD	Patients	% of Group	P-Value	SMD
1<br>2	Z55-Z65	Persons with potential health hazards related to socioeconomic and psychosocial circumstances		677<br>616	1.4%<br>1.3%	0.088	0.011
1<br>2	E66	Overweight and obesity		17,951<br>18,078	37.1%<br>37.4%	0.398	0.005
1<br>2	Z68.3	Body mass index [BMI] 30-39, adult		4,032<br>3,671	8.3%<br>7.6%	<0.001	0.028
1<br>2	Z68.4	Body mass index [BMI] 40 or greater, adult		3,768<br>3,414	7.8%<br>7.1%	<0.001	0.028
1<br>2	Z68.25	Body mass index [BMI] 25.0-25.9, adult		156<br>129	0.3%<br>0.3%	0.109	0.010

1<br>2	Z68.26	Body mass index [BMI] 26.0-26.9, adult	171<br>147	0.4%<br>0.3%	0.178	0.009
1<br>2	Z68.27	Body mass index [BMI] 27.0-27.9, adult	282<br>237	0.6%<br>0.5%	0.048	0.013
1<br>2	Z68.28	Body mass index [BMI] 28.0-28.9, adult	331<br>308	0.7%<br>0.6%	0.361	0.006
1<br>2	Z68.29	Body mass index [BMI] 29.0-29.9, adult	385<br>349	0.8%<br>0.7%	0.182	0.009
1<br>2	F10	Alcohol related disorders	562<br>436	1.2%<br>0.9%	<0.001	0.026
1<br>2	Z80	Family history of primary malignant neoplasm	1,983<br>1,891	4.1%<br>3.9%	0.131	0.010
1<br>2	Z15.0	Genetic susceptibility to malignant neoplasm	22<br>14	0.0%<br>0.0%	0.182	0.009
1<br>2	Z12	Encounter for screening for malignant neoplasms	11,908<br>11,526	24.6%<br>23.8%	0.004	0.018
1<br>2	Z85	Personal history of malignant neoplasm	1,229<br>1,032	2.5%<br>2.1%	<0.001	0.027
1<br>2	Z98.84	Bariatric surgery status	611<br>561	1.3%<br>1.2%	0.142	0.009
1<br>2	E88.81	Metabolic syndrome and other insulin resistance	1,480<br>1,497	3.1%<br>3.1%	0.752	0.002
1<br>2	E78	Disorders of lipoprotein metabolism and other lipidemias	27,908<br>27,922	57.7%<br>57.7%	0.927	0.001
1<br>2	I10	Essential (primary) hypertension	28,605<br>28,625	59.1%<br>59.1%	0.896	0.001
1<br>2	K75.81	Nonalcoholic steatohepatitis (NASH)	445<br>419	0.9%<br>0.9%	0.374	0.006
1<br>2	F17	Nicotine dependence	3,568<br>3,165	7.4%<br>6.5%	<0.001	0.033

© 2024 Wang L et al. *JAMA Network Open.*

1<br>2	E66.0	Obesity due to excess calories	8,878<br>8,836	18.3%<br>18.3%	0.727	0.002
1<br>2	E66.2	Morbid (severe) obesity with alveolar hypoventilation	149<br>129	0.3%<br>0.3%	0.230	0.008
1<br>2	E66.3	Overweight	975<br>900	2.0%<br>1.9%	0.080	0.011
1<br>2	E66.8	Other obesity	218<br>200	0.5%<br>0.4%	0.378	0.006
1<br>2	E66.9	Obesity, unspecified	13,442<br>13,708	27.8%<br>28.3%	0.057	0.012
1<br>2	Z72.0	Tobacco use	1,039<br>989	2.1%<br>2.0%	0.262	0.007
1<br>2	B18	Chronic viral hepatitis	259<br>212	0.5%<br>0.4%	0.030	0.014
1<br>2	K74	Fibrosis and cirrhosis of liver	428<br>337	0.9%<br>0.7%	0.001	0.021
1<br>2	K76.0	Fatty (change of) liver, not elsewhere classified	3,006<br>2,893	6.2%<br>6.0%	0.129	0.010
1<br>2	K74.3	Primary biliary cirrhosis	20<br>15	0.0%<br>0.0%	0.398	0.005
1<br>2	E83.110	Hereditary hemochromatosis	39<br>24	0.1%<br>0.0%	0.059	0.012
1<br>2	E70.21	Tyrosinemia	10<br>10	0.0%<br>0.0%	1	<0.001
1<br>2	E88.01	Alpha-1-antitrypsin deficiency	16<br>17	0.0%<br>0.0%	0.862	0.001
1<br>2	E80.1	Porphyria cutanea tarda	10<br>0	0.0%<br>0%	0.002	0.020
1<br>2	E74.0	Glycogen storage disease	10<br>10	0.0%<br>0.0%	1	<0.001
1<br>2	E83.00	Disorder of copper metabolism, unspecified	10<br>10	0.0%<br>0.0%	1	<0.001
1<br>2	T64	Toxic effect of aflatoxin and other mycotoxin food contaminants	10<br>10	0.0%<br>0.0%	1	<0.001
1<br>2	D13.4	Benign neoplasm of liver	33<br>23	0.1%<br>0.0%	0.181	0.009
**Medication**						

Group			Mean ± SD	Patients	% of Group	P-Value	SMD
1<br>2	A10BA	Biguanides		26,497<br>26,968	54.7%<br>55.7%	0.002	0.020
1<br>2	A10BB	Sulfonylureas		13,741<br>14,100	28.4%<br>29.1%	0.011	0.016
1<br>2	A10BF	Alpha glucosidase inhibitors		223<br>217	0.5%<br>0.4%	0.774	0.002
1<br>2	A10BG	Thiazolidinediones		4,019<br>4,113	8.3%<br>8.5%	0.276	0.007
1<br>2	A10BH	Dipeptidyl peptidase 4 (DPP-4) inhibitors		9,071<br>9,142	18.7%<br>18.9%	0.559	0.004
1<br>2	A10BK	Sodium-glucose co-transporter 2 (SGLT2) inhibitors		4,271<br>3,699	8.8%<br>7.6%	<0.001	0.043
1<br>2	A10BX	Other blood glucose lowering drugs, excl. insulins		606<br>630	1.3%<br>1.3%	0.492	0.004
1<br>2	A14A	ANABOLIC STEROIDS		59<br>46	0.1%<br>0.1%	0.204	0.008

© 2024 Wang L et al. *JAMA Network Open.*

eTable 9. Characteristics of the GLP-1RA/no insulin group and insulin/no GLP-1RA group before and after matched for covariates related to ovarian cancer for the study populations of women with T2D and no history of any OAC

Group 1 (N = 26,011) and group 2 (N = 476,110) characteristics before propensity score matching

Demographics

Group			Mean ± SD	Patients	% of Group	P-Value	SMD
1<br>2	AI	Age at Index	54.9 +/- 11.9<br>61.7 +/- 16.7	26,011<br>476,110	100%<br>100%	<0.001	0.469
1<br>2	2106-3	White		17,165<br>282,423	66.0%<br>59.3%	<0.001	0.138
1<br>2	1002-5	American Indian or Alaska Native		123<br>1,701	0.5%<br>0.4%	0.003	0.018
1<br>2	UNK	Unknown Race		2,772<br>55,979	10.7%<br>11.8%	<0.001	0.035
1<br>2	2076-8	Native Hawaiian or Other Pacific Islander		105<br>4,943	0.4%<br>1.0%	<0.001	0.075
1<br>2	UN	Unknown Ethnicity		5,078<br>124,092	19.5%<br>26.1%	<0.001	0.156
1<br>2	2186-5	Not Hispanic or Latino		18,384<br>304,990	70.7%<br>64.1%	<0.001	0.142
1<br>2	2135-2	Hispanic or Latino		2,549<br>47,028	9.8%<br>9.9%	0.682	0.003
1<br>2	2054-5	Black or African American		4,436<br>95,352	17.1%<br>20.0%	<0.001	0.077
1<br>2	2028-9	Asian		619<br>19,593	2.4%<br>4.1%	<0.001	0.098

Diagnosis

Group			Mean ± SD	Patients	% of Group	P-Value	SMD
1<br>2	Z55-Z65	Persons with potential health hazards related to socioeconomic and psychosocial circumstances		468<br>5,762	1.8%<br>1.2%	<0.001	0.048
1<br>2	E66	Overweight and obesity		10,477<br>87,968	40.3%<br>18.5%	<0.001	0.493

1<br>2	Z68.3	Body mass index [BMI] 30-39, adult	1,815<br>17,667	7.0%<br>3.7%	<0.001	0.146
1<br>2	Z68.4	Body mass index [BMI] 40 or greater, adult	2,154<br>18,645	8.3%<br>3.9%	<0.001	0.183
1<br>2	Z68.25	Body mass index [BMI] 25.0-25.9, adult	56<br>1,000	0.2%<br>0.2%	0.857	0.001
1<br>2	Z68.26	Body mass index [BMI] 26.0-26.9, adult	50<br>959	0.2%<br>0.2%	0.747	0.002
1<br>2	Z68.27	Body mass index [BMI] 27.0-27.9, adult	87<br>1,140	0.3%<br>0.2%	0.003	0.018
1<br>2	Z68.28	Body mass index [BMI] 28.0-28.9, adult	107<br>1,266	0.4%<br>0.3%	<0.001	0.025
1<br>2	Z68.29	Body mass index [BMI] 29.0-29.9, adult	119<br>1,376	0.5%<br>0.3%	<0.001	0.028
1<br>2	F10	Alcohol related disorders	197<br>6,405	0.8%<br>1.3%	<0.001	0.058
1<br>2	Z80	Family history of primary malignant neoplasm	1,430<br>11,367	5.5%<br>2.4%	<0.001	0.160
1<br>2	Z15.0	Genetic susceptibility to malignant neoplasm	18<br>100	0.1%<br>0.0%	<0.001	0.023
1<br>2	Z12	Encounter for screening for malignant neoplasms	7,877<br>65,044	30.3%<br>13.7%	<0.001	0.410
1<br>2	Z85	Personal history of malignant neoplasm	593<br>14,844	2.3%<br>3.1%	<0.001	0.052
1<br>2	Z98.84	Bariatric surgery status	474<br>3,408	1.8%<br>0.7%	<0.001	0.099
1<br>2	F17	Nicotine dependence	1,905<br>39,400	7.3%<br>8.3%	<0.001	0.035
1<br>2	E66.0	Obesity due to excess calories	5,392<br>39,813	20.7%<br>8.4%	<0.001	0.356
1<br>2	E66.2	Morbid (severe) obesity with alveolar hypoventilation	82<br>1,919	0.3%<br>0.4%	0.029	0.015

© 2024 Wang L et al. *JAMA Network Open.*

1<br>2	E66.3	Overweight		535<br>3,181	2.1%<br>0.7%	<0.001	0.120
1<br>2	E66.8	Other obesity		134<br>702	0.5%<br>0.1%	<0.001	0.064
1<br>2	E66.9	Obesity, unspecified		7,879<br>61,596	30.3%<br>12.9%	<0.001	0.431
1<br>2	Z72.0	Tobacco use		572<br>5,857	2.2%<br>1.2%	<0.001	0.075
1<br>2	Z79.890	Hormone replacement therapy		137<br>1,374	0.5%<br>0.3%	<0.001	0.037
1<br>2	Z92.23	Personal history of estrogen therapy		10<br>124	0.0%<br>0.0%	0.233	0.007
1<br>2	Z80.3	Family history of malignant neoplasm of breast		736<br>5,122	2.8%<br>1.1%	<0.001	0.127
1<br>2	E28.2	Polycystic ovarian syndrome		979<br>2,780	3.8%<br>0.6%	<0.001	0.219
1<br>2	Z80.41	Family history of malignant neoplasm of ovary		104<br>554	0.4%<br>0.1%	<0.001	0.056
1<br>2	D27	Benign neoplasm of ovary		95<br>902	0.4%<br>0.2%	<0.001	0.033
**Medication**							
Group			Mean ± SD	Patients	% of Group	P-Value	SMD
1<br>2	A10BA	Biguanides		14,271<br>93,949	54.9%<br>19.7%	<0.001	0.780
1<br>2	A10BB	Sulfonylureas		6,822<br>56,352	26.2%<br>11.8%	<0.001	0.373
1<br>2	A10BF	Alpha glucosidase inhibitors		112<br>784	0.4%<br>0.2%	<0.001	0.049
1<br>2	A10BG	Thiazolidinediones		1,834<br>15,689	7.1%<br>3.3%	<0.001	0.170
1<br>2	A10BH	Dipeptidyl peptidase 4 (DPP-4) inhibitors		4,637<br>20,235	17.8%<br>4.3%	<0.001	0.444
1<br>2	A10BK	Sodium-glucose co-transporter 2 (SGLT2) inhibitors		2,219<br>2,614	8.5%<br>0.5%	<0.001	0.391
1<br>2	A10BX	Other blood glucose lowering drugs, excl. insulins		297<br>3,287	1.1%<br>0.7%	<0.001	0.047

© 2024 Wang L et al. *JAMA Network Open.*

1<br>2	HS200	CONTRACEPTIVES,SYSTEMIC		1,345<br>5,117	5.2%<br>1.1%	<0.001	0.237

Group 1 (N = 25,739) and group 2 (N = 25,739) characteristics after propensity score matching

Demographics

Group			Mean ± SD	Patients	% of Group	P-Value	SMD
1<br>2	AI	Age at Index	55.0 +/- 11.9<br>54.8 +/- 13.9	25,739<br>25,739	100%<br>100%	0.081	0.015
1<br>2	2106-3	White		16,960<br>17,436	65.9%<br>67.7%	<0.001	0.039
1<br>2	1002-5	American Indian or Alaska Native		123<br>108	0.5%<br>0.4%	0.323	0.009
1<br>2	UNK	Unknown Race		2,754<br>2,619	10.7%<br>10.2%	0.052	0.017
1<br>2	2076-8	Native Hawaiian or Other Pacific Islander		105<br>82	0.4%<br>0.3%	0.092	0.015
1<br>2	UN	Unknown Ethnicity		5,052<br>4,906	19.6%<br>19.1%	0.103	0.014
1<br>2	2186-5	Not Hispanic or Latino		18,163<br>18,347	70.6%<br>71.3%	0.074	0.016
1<br>2	2135-2	Hispanic or Latino		2,524<br>2,486	9.8%<br>9.7%	0.572	0.005
1<br>2	2054-5	Black or African American		4,396<br>4,258	17.1%<br>16.5%	0.104	0.014
1<br>2	2028-9	Asian		615<br>539	2.4%<br>2.1%	0.024	0.020

Diagnosis

Group			Mean ± SD	Patients	% of Group	P-Value	SMD
1<br>2	Z55-Z65	Persons with potential health hazards related to socioeconomic and psychosocial circumstances		461<br>418	1.8%<br>1.6%	0.143	0.013
1<br>2	E66	Overweight and obesity		10,260<br>10,332	39.9%<br>40.1%	0.517	0.006
1<br>2	Z68.3	Body mass index [BMI] 30-39, adult		1,779<br>1,559	6.9%<br>6.1%	<0.001	0.035

© 2024 Wang L et al. *JAMA Network Open.*

1<br>2	Z68.4	Body mass index [BMI] 40 or greater, adult	2,100<br>1,922	8.2%<br>7.5%	0.003	0.026
1<br>2	Z68.25	Body mass index [BMI] 25.0-25.9, adult	56<br>50	0.2%<br>0.2%	0.560	0.005
1<br>2	Z68.26	Body mass index [BMI] 26.0-26.9, adult	50<br>58	0.2%<br>0.2%	0.441	0.007
1<br>2	Z68.27	Body mass index [BMI] 27.0-27.9, adult	87<br>69	0.3%<br>0.3%	0.149	0.013
1<br>2	Z68.28	Body mass index [BMI] 28.0-28.9, adult	106<br>85	0.4%<br>0.3%	0.128	0.013
1<br>2	Z68.29	Body mass index [BMI] 29.0-29.9, adult	118<br>91	0.5%<br>0.4%	0.061	0.016
1<br>2	F10	Alcohol related disorders	197<br>155	0.8%<br>0.6%	0.025	0.020
1<br>2	Z80	Family history of primary malignant neoplasm	1,396<br>1,245	5.4%<br>4.8%	0.003	0.027
1<br>2	Z15.0	Genetic susceptibility to malignant neoplasm	17<br>10	0.1%<br>0.0%	0.178	0.012
1<br>2	Z12	Encounter for screening for malignant neoplasms	7,694<br>7,284	29.9%<br>28.3%	<0.001	0.035
1<br>2	Z85	Personal history of malignant neoplasm	590<br>509	2.3%<br>2.0%	0.014	0.022
1<br>2	Z98.84	Bariatric surgery status	459<br>456	1.8%<br>1.8%	0.920	0.001
1<br>2	F17	Nicotine dependence	1,889<br>1,719	7.3%<br>6.7%	0.003	0.026
1<br>2	E66.0	Obesity due to excess calories	5,262<br>5,280	20.4%<br>20.5%	0.844	0.002
1<br>2	E66.2	Morbid (severe) obesity with alveolar hypoventilation	81<br>71	0.3%<br>0.3%	0.417	0.007
1<br>2	E66.3	Overweight	512<br>463	2.0%<br>1.8%	0.113	0.014

© 2024 Wang L et al. *JAMA Network Open.*

1<br>2	E66.8	Other obesity		127<br>129	0.5%<br>0.5%	0.900	0.001
1<br>2	E66.9	Obesity, unspecified		7,706<br>7,822	29.9%<br>30.4%	0.265	0.010
1<br>2	Z72.0	Tobacco use		562<br>532	2.2%<br>2.1%	0.359	0.008
1<br>2	Z79.89<br>0	Hormone replacement therapy		134<br>108	0.5%<br>0.4%	0.094	0.015
1<br>2	Z92.23	Personal history of estrogen therapy		10<br>10	0.0%<br>0.0%	1	<0.001
1<br>2	Z80.3	Family history of malignant neoplasm of breast		720<br>636	2.8%<br>2.5%	0.021	0.020
1<br>2	E28.2	Polycystic ovarian syndrome		938<br>901	3.6%<br>3.5%	0.380	0.008
1<br>2	Z80.41	Family history of malignant neoplasm of ovary		99<br>86	0.4%<br>0.3%	0.338	0.008
1<br>2	D27	Benign neoplasm of ovary		94<br>73	0.4%<br>0.3%	0.104	0.014
**Medication**							
Group			Mean ± SD	Patients	% of Group	P-Value	SMD
1<br>2	A10B<br>A	Biguanides		14,003<br>14,214	54.4%<br>55.2%	0.062	0.016
1<br>2	A10B<br>B	Sulfonylureas		6,697<br>6,766	26.0%<br>26.3%	0.489	0.006
1<br>2	A10B<br>F	Alpha glucosidase inhibitors		109<br>96	0.4%<br>0.4%	0.363	0.008
1<br>2	A10B<br>G	Thiazolidinediones		1,797<br>1,770	7.0%<br>6.9%	0.639	0.004
1<br>2	A10B<br>H	Dipeptidyl peptidase 4 (DPP-4) inhibitors		4,445<br>4,477	17.3%<br>17.4%	0.709	0.003
1<br>2	A10B<br>K	Sodium-glucose co-transporter 2 (SGLT2) inhibitors		1,970<br>1,690	7.7%<br>6.6%	<0.00<br>1	0.042
1<br>2	A10B<br>X	Other blood glucose lowering drugs, excl. insulins		292<br>283	1.1%<br>1.1%	0.706	0.003
1<br>2	HS200	CONTRACEPTIVE S,SYSTEMIC		1,292<br>1,219	5.0%<br>4.7%	0.135	0.013

© 2024 Wang L et al. *JAMA Network Open.*

eTable 10. Characteristics of the GLP-1RA/no insulin group and insulin/no GLP-1RA group before and after matched for covariates related to pancreatic cancer for the study populations of patients with T2D and no history of any OAC

Group 1 (N = 48,983) and group 2 (N = 1,044,745) characteristics before propensity score matching

Demographics

Group			Mean ± SD	Patients	% of Group	P-Value	SMD
1<br>2	AI	Age at Index	55.9 +/- 11.7<br>61.8 +/- 15.9	48,983<br>1,044,745	100%<br>100%	<0.001	0.420
1<br>2	2106-3	White		32,592<br>633,989	66.5%<br>60.7%	<0.001	0.122
1<br>2	1002-5	American Indian or Alaska Native		199<br>3,443	0.4%<br>0.3%	0.004	0.013
1<br>2	UNK	Unknown Race		7,099<br>142,470	14.5%<br>13.6%	<0.001	0.025
1<br>2	F	Female		26,011<br>476,110	53.1%<br>45.6%	<0.001	0.151
1<br>2	2076-8	Native Hawaiian or Other Pacific Islander		205<br>10,677	0.4%<br>1.0%	<0.001	0.071
1<br>2	UN	Unknown Gender		2,252<br>27,321	4.6%<br>2.6%	<0.001	0.106
1<br>2	2186-5	Not Hispanic or Latino		33,188<br>659,375	67.8%<br>63.1%	<0.001	0.098
1<br>2	2135-2	Hispanic or Latino		4,151<br>94,136	8.5%<br>9.0%	<0.001	0.019
1<br>2	2054-5	Black or African American		6,265<br>178,267	12.8%<br>17.1%	<0.001	0.120
1<br>2	M	Male		20,720<br>541,314	42.3%<br>51.8%	<0.001	0.191
1<br>2	2028-9	Asian		1,204<br>41,822	2.5%<br>4.0%	<0.001	0.087

Diagnosis

Group			Mean ± SD	Patients	% of Group	P-Value	SMD
1<br>2	Z55-Z65	Persons with potential health hazards related to socioeconomic and		686<br>12,021	1.4%<br>1.2%	<0.001	0.022

© 2024 Wang L et al. *JAMA Network Open.*

		psychosocial circumstances				
1<br>2	E66	Overweight and obesity	18,401<br>166,445	37.6%<br>15.9%	<0.001	0.504
1<br>2	Z68.3	Body mass index [BMI] 30-39, adult	4,204<br>43,128	8.6%<br>4.1%	<0.001	0.183
1<br>2	Z68.4	Body mass index [BMI] 40 or greater, adult	3,886<br>33,396	7.9%<br>3.2%	<0.001	0.208
1<br>2	Z68.25	Body mass index [BMI] 25.0-25.9, adult	157<br>3,510	0.3%<br>0.3%	0.563	0.003
1<br>2	Z68.26	Body mass index [BMI] 26.0-26.9, adult	171<br>3,632	0.3%<br>0.3%	0.957	<0.001
1<br>2	Z68.27	Body mass index [BMI] 27.0-27.9, adult	288<br>4,209	0.6%<br>0.4%	<0.001	0.026
1<br>2	Z68.28	Body mass index [BMI] 28.0-28.9, adult	344<br>4,508	0.7%<br>0.4%	<0.001	0.036
1<br>2	Z68.29	Body mass index [BMI] 29.0-29.9, adult	398<br>4,843	0.8%<br>0.5%	<0.001	0.044
1<br>2	F10	Alcohol related disorders	563<br>28,538	1.1%<br>2.7%	<0.001	0.115
1<br>2	Z80	Family history of primary malignant neoplasm	2,042<br>19,398	4.2%<br>1.9%	<0.001	0.136
1<br>2	Z15.0	Genetic susceptibility to malignant neoplasm	24<br>156	0.0%<br>0.0%	<0.001	0.019
1<br>2	Z12	Encounter for screening for malignant neoplasms	12,272<br>105,217	25.1%<br>10.1%	<0.001	0.402
1<br>2	Z85	Personal history of malignant neoplasm	1,239<br>38,294	2.5%<br>3.7%	<0.001	0.066
1<br>2	Z98.84	Bariatric surgery status	632<br>5,136	1.3%<br>0.5%	<0.001	0.085
1<br>2	E66.0	Obesity due to excess calories	9,157<br>69,998	18.7%<br>6.7%	<0.001	0.366

© 2024 Wang L et al. *JAMA Network Open.*

1<br>2	E66.2	Morbid (severe) obesity with alveolar hypoventilation		150<br>3,643	0.3%<br>0.3%	0.118	0.007
1<br>2	E66.3	Overweight		1,010<br>6,796	2.1%<br>0.7%	<0.001	0.122
1<br>2	E66.8	Other obesity		235<br>1,298	0.5%<br>0.1%	<0.001	0.065
1<br>2	E66.9	Obesity, unspecified		13,805<br>118,555	28.2%<br>11.3%	<0.001	0.433
1<br>2	Z85.09	Personal history of malignant neoplasm of other digestive organs		10<br>256	0.0%<br>0.0%	0.571	0.003
1<br>2	B96.81	Helicobacter pylori [H. pylori] as the cause of diseases classified elsewhere		254<br>3,973	0.5%<br>0.4%	<0.001	0.021
1<br>2	Z80.0	Family history of malignant neoplasm of digestive organs		784<br>6,889	1.6%<br>0.7%	<0.001	0.089
1<br>2	B18	Chronic viral hepatitis		260<br>12,879	0.5%<br>1.2%	<0.001	0.075
1<br>2	K86.1	Other chronic pancreatitis		75<br>8,232	0.2%<br>0.8%	<0.001	0.093
1<br>2	B16	Acute hepatitis B		55<br>1,537	0.1%<br>0.1%	0.048	0.010
1<br>2	B18.1	Chronic viral hepatitis B without delta-agent		48<br>1,611	0.1%<br>0.2%	0.002	0.016
1<br>2	Z72.0	Tobacco use		1,064<br>13,876	2.2%<br>1.3%	<0.001	0.064
1<br>2	D13.6	Benign neoplasm of pancreas		10<br>360	0.0%<br>0.0%	0.099	0.008

Medication

Group			Mean ± SD	Patients	% of Group	P-Value	SMD
1<br>2	A10BA	Biguanides		27,075<br>199,802	55.3%<br>19.1%	<0.001	0.806
1<br>2	A10BB	Sulfonylureas		14,077<br>125,703	28.7%<br>12.0%	<0.001	0.424
1<br>2	A10BF	Alpha glucosidase inhibitors		229<br>1,718	0.5%<br>0.2%	<0.001	0.054

1<br>2	A10BG	Thiazolidinediones		4,107<br>35,435	8.4%<br>3.4%	<0.001	0.213
1<br>2	A10BH	Dipeptidyl peptidase 4 (DPP-4) inhibitors		9,485<br>44,595	19.4%<br>4.3%	<0.001	0.481
1<br>2	A10BK	Sodium-glucose co-transporter 2 (SGLT2) inhibitors		4,808<br>6,447	9.8%<br>0.6%	<0.001	0.423
1<br>2	A10BX	Other blood glucose lowering drugs, excl. insulins		614<br>7,048	1.3%<br>0.7%	<0.001	0.059
**Group 1 (N = 48,490) and group 2 (N = 48,490) characteristics after propensity score matching**							
**Demographics**							
Group			Mean ± SD	Patients	% of Group	P-Value	SMD
1<br>2	AI	Age at Index	55.9 +/- 11.7<br>56.0 +/- 13.1	48,490<br>48,490	100%<br>100%	0.154	0.009
1<br>2	2106-3	White		32,268<br>33,234	66.5%<br>68.5%	<0.001	0.043
1<br>2	1002-5	American Indian or Alaska Native		197<br>179	0.4%<br>0.4%	0.352	0.006
1<br>2	UNK	Unknown Race		6,991<br>6,652	14.4%<br>13.7%	0.002	0.020
1<br>2	F	Female		25,736<br>26,279	53.1%<br>54.2%	<0.001	0.022
1<br>2	2076-8	Native Hawaiian or Other Pacific Islander		205<br>145	0.4%<br>0.3%	0.001	0.021
1<br>2	UN	Unknown Gender		2,179<br>2,038	4.5%<br>4.2%	0.026	0.014
1<br>2	2186-5	Not Hispanic or Latino		32,860<br>33,363	67.8%<br>68.8%	0.001	0.022
1<br>2	2135-2	Hispanic or Latino		4,119<br>3,937	8.5%<br>8.1%	0.034	0.014
1<br>2	2054-5	Black or African American		6,223<br>5,922	12.8%<br>12.2%	0.003	0.019
1<br>2	M	Male		20,575<br>20,173	42.4%<br>41.6%	0.009	0.017
1<br>2	2028-9	Asian		1,199<br>1,036	2.5%<br>2.1%	<0.001	0.022
**Diagnosis**							

© 2024 Wang L et al. *JAMA Network Open.*

Group			Mean ± SD	Patients	% of Group	P-Value	SMD
1<br>2	Z55-Z65	Persons with potential health hazards related to socioeconomic and psychosocial circumstances		679<br>566	1.4%<br>1.2%	0.001	0.021
1<br>2	E66	Overweight and obesity		18,013<br>17,981	37.1%<br>37.1%	0.832	0.001
1<br>2	Z68.3	Body mass index [BMI] 30-39, adult		4,063<br>3,633	8.4%<br>7.5%	<0.001	0.033
1<br>2	Z68.4	Body mass index [BMI] 40 or greater, adult		3,789<br>3,390	7.8%<br>7.0%	<0.001	0.031
1<br>2	Z68.25	Body mass index [BMI] 25.0-25.9, adult		156<br>150	0.3%<br>0.3%	0.731	0.002
1<br>2	Z68.26	Body mass index [BMI] 26.0-26.9, adult		171<br>149	0.4%<br>0.3%	0.218	0.008
1<br>2	Z68.27	Body mass index [BMI] 27.0-27.9, adult		284<br>233	0.6%<br>0.5%	0.025	0.014
1<br>2	Z68.28	Body mass index [BMI] 28.0-28.9, adult		334<br>283	0.7%<br>0.6%	0.039	0.013
1<br>2	Z68.29	Body mass index [BMI] 29.0-29.9, adult		386<br>351	0.8%<br>0.7%	0.196	0.008
1<br>2	F10	Alcohol related disorders		562<br>460	1.2%<br>0.9%	0.001	0.021
1<br>2	Z80	Family history of primary malignant neoplasm		1,995<br>1,859	4.1%<br>3.8%	0.025	0.014
1<br>2	Z15.0	Genetic susceptibility to malignant neoplasm		22<br>17	0.0%<br>0.0%	0.423	0.005
1<br>2	Z12	Encounter for screening for malignant neoplasms		11,955<br>11,588	24.7%<br>23.9%	0.006	0.018
1<br>2	Z85	Personal history of malignant neoplasm		1,230<br>1,109	2.5%<br>2.3%	0.011	0.016

© 2024 Wang L et al. *JAMA Network Open.*

1<br>2	Z98.84	Bariatric surgery status		613<br>551	1.3%<br>1.1%	0.068	0.012
1<br>2	E66.0	Obesity due to excess calories		8,924<br>8,803	18.4%<br>18.2%	0.315	0.006
1<br>2	E66.2	Morbid (severe) obesity with alveolar hypoventilation		149<br>137	0.3%<br>0.3%	0.477	0.005
1<br>2	E66.3	Overweight		980<br>907	2.0%<br>1.9%	0.090	0.011
1<br>2	E66.8	Other obesity		223<br>196	0.5%<br>0.4%	0.186	0.008
1<br>2	E66.9	Obesity, unspecified		13,488<br>13,624	27.8%<br>28.1%	0.331	0.006
1<br>2	Z85.09	Personal history of malignant neoplasm of other digestive organs		10<br>10	0.0%<br>0.0%	1	<0.001
1<br>2	B96.8<br>1	Helicobacter pylori [H. pylori] as the cause of diseases classified elsewhere		252<br>220	0.5%<br>0.5%	0.140	0.009
1<br>2	Z80.0	Family history of malignant neoplasm of digestive organs		763<br>659	1.6%<br>1.4%	0.005	0.018
1<br>2	B18	Chronic viral hepatitis		260<br>217	0.5%<br>0.4%	0.048	0.013
1<br>2	K86.1	Other chronic pancreatitis		75<br>105	0.2%<br>0.2%	0.025	0.014
1<br>2	B16	Acute hepatitis B		54<br>34	0.1%<br>0.1%	0.033	0.014
1<br>2	B18.1	Chronic viral hepatitis B without delta-agent		48<br>23	0.1%<br>0.0%	0.003	0.019
1<br>2	Z72.0	Tobacco use		1,047<br>959	2.2%<br>2.0%	0.047	0.013
1<br>2	D13.6	Benign neoplasm of pancreas		10<br>11	0.0%<br>0.0%	0.827	0.001
**Medication**							
Group			Mean ± SD	Patients	% of Group	P-Value	SMD
1<br>2	A10B<br>A	Biguanides		26,585<br>27,020	54.8%<br>55.7%	0.005	0.018

© 2024 Wang L et al. *JAMA Network Open.*

1<br>2	A10B<br>B	Sulfonylureas	13,771<br>13,977	28.4%<br>28.8%	0.143	0.009
1<br>2	A10B<br>F	Alpha glucosidase inhibitors	221<br>192	0.5%<br>0.4%	0.153	0.009
1<br>2	A10B<br>G	Thiazolidinediones	4,030<br>4,127	8.3%<br>8.5%	0.262	0.007
1<br>2	A10B<br>H	Dipeptidyl peptidase 4 (DPP-4) inhibitors	9,110<br>9,241	18.8%<br>19.1%	0.283	0.007
1<br>2	A10B<br>K	Sodium-glucose co-transporter 2 (SGLT2) inhibitors	4,335<br>3,863	8.9%<br>8.0%	<0.00<br>1	0.035
1<br>2	A10B<br>X	Other blood glucose lowering drugs, excl. insulins	607<br>545	1.3%<br>1.1%	0.066	0.012

© 2024 Wang L et al. *JAMA Network Open.*

eTable 11. Characteristics of the GLP-1RA/no insulin group and insulin/no GLP-1RA group before and after matched for covariates related to thyroid cancer for the study populations of patients with T2D and no history of any OAC

Group 1 (N = 48,983) and group 2 (N = 1,044,745) characteristics before propensity score matching

Demographics

Group			Mean ± SD	Patients	% of Group	P-Value	SMD
1<br>2	AI	Age at Index	55.9 +/- 11.7<br>61.8 +/- 15.9	48,983<br>1,044,745	100%<br>100%	<0.001	0.420
1<br>2	2106-3	White		32,592<br>633,989	66.5%<br>60.7%	<0.001	0.122
1<br>2	1002-5	American Indian or Alaska Native		199<br>3,443	0.4%<br>0.3%	0.004	0.013
1<br>2	UNK	Unknown Race		7,099<br>142,470	14.5%<br>13.6%	<0.001	0.025
1<br>2	F	Female		26,011<br>476,110	53.1%<br>45.6%	<0.001	0.151
1<br>2	2076-8	Native Hawaiian or Other Pacific Islander		205<br>10,677	0.4%<br>1.0%	<0.001	0.071
1<br>2	UN	Unknown Gender		2,252<br>27,321	4.6%<br>2.6%	<0.001	0.106
1<br>2	2186-5	Not Hispanic or Latino		33,188<br>659,375	67.8%<br>63.1%	<0.001	0.098
1<br>2	2135-2	Hispanic or Latino		4,151<br>94,136	8.5%<br>9.0%	<0.001	0.019
1<br>2	2054-5	Black or African American		6,265<br>178,267	12.8%<br>17.1%	<0.001	0.120
1<br>2	M	Male		20,720<br>541,314	42.3%<br>51.8%	<0.001	0.191
1<br>2	2028-9	Asian		1,204<br>41,822	2.5%<br>4.0%	<0.001	0.087

Diagnosis

Group			Mean ± SD	Patients	% of Group	P-Value	SMD
1<br>2	Z55-Z65	Persons with potential health hazards related to socioeconomic and		686<br>12,021	1.4%<br>1.2%	<0.001	0.022

© 2024 Wang L et al. *JAMA Network Open.*

		psychosocial circumstances				
1<br>2	E66	Overweight and obesity	18,401<br>166,445	37.6%<br>15.9%	<0.001	0.504
1<br>2	Z68.3	Body mass index [BMI] 30-39, adult	4,204<br>43,128	8.6%<br>4.1%	<0.001	0.183
1<br>2	Z68.4	Body mass index [BMI] 40 or greater, adult	3,886<br>33,396	7.9%<br>3.2%	<0.001	0.208
1<br>2	Z68.25	Body mass index [BMI] 25.0-25.9, adult	157<br>3,510	0.3%<br>0.3%	0.563	0.003
1<br>2	Z68.26	Body mass index [BMI] 26.0-26.9, adult	171<br>3,632	0.3%<br>0.3%	0.957	<0.001
1<br>2	Z68.27	Body mass index [BMI] 27.0-27.9, adult	288<br>4,209	0.6%<br>0.4%	<0.001	0.026
1<br>2	Z68.28	Body mass index [BMI] 28.0-28.9, adult	344<br>4,508	0.7%<br>0.4%	<0.001	0.036
1<br>2	Z68.29	Body mass index [BMI] 29.0-29.9, adult	398<br>4,843	0.8%<br>0.5%	<0.001	0.044
1<br>2	Z80	Family history of primary malignant neoplasm	2,042<br>19,398	4.2%<br>1.9%	<0.001	0.136
1<br>2	Z15.0	Genetic susceptibility to malignant neoplasm	24<br>156	0.0%<br>0.0%	<0.001	0.019
1<br>2	Z12	Encounter for screening for malignant neoplasms	12,272<br>105,217	25.1%<br>10.1%	<0.001	0.402
1<br>2	Z85	Personal history of malignant neoplasm	1,239<br>38,294	2.5%<br>3.7%	<0.001	0.066
1<br>2	Z98.84	Bariatric surgery status	632<br>5,136	1.3%<br>0.5%	<0.001	0.085
1<br>2	E66.0	Obesity due to excess calories	9,157<br>69,998	18.7%<br>6.7%	<0.001	0.366
1<br>2	E66.2	Morbid (severe) obesity with alveolar hypoventilation	150<br>3,643	0.3%<br>0.3%	0.118	0.007

© 2024 Wang L et al. *JAMA Network Open.*

Group			Mean ± SD	Patients	% of Group	P-Value	SMD
1<br>2	E66.3	Overweight		1,010<br>6,796	2.1%<br>0.7%	<0.001	0.122
1<br>2	E66.8	Other obesity		235<br>1,298	0.5%<br>0.1%	<0.001	0.065
1<br>2	E66.9	Obesity, unspecified		13,805<br>118,555	28.2%<br>11.3%	<0.001	0.433
1<br>2	D34	Benign neoplasm of thyroid gland		65<br>765	0.1%<br>0.1%	<0.001	0.019
**Procedure**							
Group			Mean ± SD	Patients	% of Group	P-Value	SMD
1<br>2	1027853	Hereditary neuroendocrine tumor disorders (eg, medullary thyroid carcinoma, parathyroid carcinoma, malignant pheochromocytoma or paraganglioma)		0<br>0	0%<br>0%	--	--
1<br>2	1001	Radiation		138<br>7,649	0.3%<br>0.7%	<0.001	0.063
**Medication**							
Group			Mean ± SD	Patients	% of Group	P-Value	SMD
1<br>2	A10BA	Biguanides		27,075<br>199,802	55.3%<br>19.1%	<0.001	0.806
1<br>2	A10BB	Sulfonylureas		14,077<br>125,703	28.7%<br>12.0%	<0.001	0.424
1<br>2	A10BF	Alpha glucosidase inhibitors		229<br>1,718	0.5%<br>0.2%	<0.001	0.054
1<br>2	A10BG	Thiazolidinediones		4,107<br>35,435	8.4%<br>3.4%	<0.001	0.213
1<br>2	A10BH	Dipeptidyl peptidase 4 (DPP-4) inhibitors		9,485<br>44,595	19.4%<br>4.3%	<0.001	0.481
1<br>2	A10BK	Sodium-glucose co-transporter 2 (SGLT2) inhibitors		4,808<br>6,447	9.8%<br>0.6%	<0.001	0.423
1<br>2	A10BX	Other blood glucose lowering drugs, excl. insulins		614<br>7,048	1.3%<br>0.7%	<0.001	0.059

© 2024 Wang L et al. *JAMA Network Open.*

Group 1 (N = 48,527) and group 2 (N = 48,527) characteristics after propensity score matching

Demographics

Group			Mean ± SD	Patients	% of Group	P-Value	SMD
1<br>2	AI	Age at Index	55.9 +/- 11.7<br>56.0 +/- 13.0	48,527<br>48,527	100%<br>100%	0.154	0.009
1<br>2	2106-3	White		32,294<br>33,289	66.5%<br>68.6%	<0.001	0.044
1<br>2	1002-5	American Indian or Alaska Native		197<br>165	0.4%<br>0.3%	0.092	0.011
1<br>2	UNK	Unknown Race		6,995<br>6,615	14.4%<br>13.6%	<0.001	0.023
1<br>2	F	Female		25,749<br>26,218	53.1%<br>54.0%	0.003	0.019
1<br>2	2076-8	Native Hawaiian or Other Pacific Islander		205<br>163	0.4%<br>0.3%	0.028	0.014
1<br>2	UN	Unknown Gender		2,183<br>2,043	4.5%<br>4.2%	0.028	0.014
1<br>2	2186-5	Not Hispanic or Latino		32,880<br>33,324	67.8%<br>68.7%	0.002	0.020
1<br>2	2135-2	Hispanic or Latino		4,125<br>3,889	8.5%<br>8.0%	0.006	0.018
1<br>2	2054-5	Black or African American		6,231<br>6,048	12.8%<br>12.5%	0.077	0.011
1<br>2	M	Male		20,595<br>20,266	42.4%<br>41.8%	0.032	0.014
1<br>2	2028-9	Asian		1,196<br>985	2.5%<br>2.0%	<0.001	0.029

Diagnosis

Group			Mean ± SD	Patients	% of Group	P-Value	SMD
1<br>2	Z55-Z65	Persons with potential health hazards related to socioeconomic and psychosocial circumstances		682<br>560	1.4%<br>1.2%	<0.001	0.022
1<br>2	E66	Overweight and obesity		18,048<br>18,155	37.2%<br>37.4%	0.478	0.005

© 2024 Wang L et al. *JAMA Network Open.*

1<br>2	Z68.3	Body mass index [BMI] 30-39, adult	4,070<br>3,762	8.4%<br>7.8%	<0.00<br>1	0.023
1<br>2	Z68.4	Body mass index [BMI] 40 or greater, adult	3,792<br>3,443	7.8%<br>7.1%	<0.00<br>1	0.027
1<br>2	Z68.25	Body mass index [BMI] 25.0-25.9, adult	156<br>151	0.3%<br>0.3%	0.775	0.002
1<br>2	Z68.26	Body mass index [BMI] 26.0-26.9, adult	171<br>172	0.4%<br>0.4%	0.957	<0.001
1<br>2	Z68.27	Body mass index [BMI] 27.0-27.9, adult	285<br>222	0.6%<br>0.5%	0.005	0.018
1<br>2	Z68.28	Body mass index [BMI] 28.0-28.9, adult	335<br>277	0.7%<br>0.6%	0.019	0.015
1<br>2	Z68.29	Body mass index [BMI] 29.0-29.9, adult	385<br>341	0.8%<br>0.7%	0.101	0.011
1<br>2	Z80	Family history of primary malignant neoplasm	1,997<br>1,898	4.1%<br>3.9%	0.105	0.010
1<br>2	Z15.0	Genetic susceptibility to malignant neoplasm	22<br>11	0.0%<br>0.0%	0.055	0.012
1<br>2	Z12	Encounter for screening for malignant neoplasms	11,975<br>11,599	24.7%<br>23.9%	0.005	0.018
1<br>2	Z85	Personal history of malignant neoplasm	1,232<br>1,034	2.5%<br>2.1%	<0.00<br>1	0.027
1<br>2	Z98.84	Bariatric surgery status	614<br>616	1.3%<br>1.3%	0.954	<0.001
1<br>2	E66.0	Obesity due to excess calories	8,948<br>8,864	18.4%<br>18.3%	0.486	0.004
1<br>2	E66.2	Morbid (severe) obesity with alveolar hypoventilation	147<br>151	0.3%<br>0.3%	0.816	0.001
1<br>2	E66.3	Overweight	982<br>870	2.0%<br>1.8%	0.009	0.017
1<br>2	E66.8	Other obesity	226<br>175	0.5%<br>0.4%	0.011	0.016

© 2024 Wang L et al. *JAMA Network Open.*

Group	Code	Name	Mean ± SD	Patients	% of Group	P-Value	SMD
1<br>2	E66.9	Obesity, unspecified		13,514<br>13,850	27.8%<br>28.5%	0.017	0.015
1<br>2	D34	Benign neoplasm of thyroid gland		62<br>56	0.1%<br>0.1%	0.580	0.004

Procedure

Group			Mean ± SD	Patients	% of Group	P-Value	SMD
1<br>2	1027853	Hereditary neuroendocrine tumor disorders (eg, medullary thyroid carcinoma, parathyroid carcinoma, malignant pheochromocytoma or paraganglioma)		0<br>0	0%<br>0%	--	--
1<br>2	1001	Radiation		138<br>155	0.3%<br>0.3%	0.320	0.006

Medication

Group			Mean ± SD	Patients	% of Group	P-Value	SMD
1<br>2	A10BA	Biguanides		26,621<br>26,945	54.9%<br>55.5%	0.036	0.013
1<br>2	A10BB	Sulfonylureas		13,798<br>14,163	28.4%<br>29.2%	0.010	0.017
1<br>2	A10BF	Alpha glucosidase inhibitors		222<br>186	0.5%<br>0.4%	0.074	0.011
1<br>2	A10BG	Thiazolidinediones		4,033<br>4,134	8.3%<br>8.5%	0.243	0.007
1<br>2	A10BH	Dipeptidyl peptidase 4 (DPP-4) inhibitors		9,131<br>9,213	18.8%<br>19.0%	0.501	0.004
1<br>2	A10BK	Sodium-glucose co-transporter 2 (SGLT2) inhibitors		4,366<br>3,968	9.0%<br>8.2%	<0.001	0.029
1<br>2	A10BX	Other blood glucose lowering drugs, excl. insulins		607<br>550	1.3%<br>1.1%	0.092	0.011

© 2024 Wang L et al. *JAMA Network Open.*

eTable 12. Characteristics of the GLP-1RA/no insulin group and insulin/no GLP-1RA group before and after matched for covariates related to meningioma for the study populations of patients with T2D and no history of any OAC

Group 1 (N = 48,983) and group 2 (N = 1,044,745) characteristics before propensity score matching

Demographics

Group			Mean ± SD	Patients	% of Group	P-Value	SMD
1<br>2	AI	Age at Index	55.9 +/- 11.7<br>61.8 +/- 15.9	48,983<br>1,044,745	100%<br>100%	<0.001	0.420
1<br>2	2106-3	White		32,592<br>633,989	66.5%<br>60.7%	<0.001	0.122
1<br>2	1002-5	American Indian or Alaska Native		199<br>3,443	0.4%<br>0.3%	0.004	0.013
1<br>2	UNK	Unknown Race		7,099<br>142,470	14.5%<br>13.6%	<0.001	0.025
1<br>2	F	Female		26,011<br>476,110	53.1%<br>45.6%	<0.001	0.151
1<br>2	2076-8	Native Hawaiian or Other Pacific Islander		205<br>10,677	0.4%<br>1.0%	<0.001	0.071
1<br>2	UN	Unknown Gender		2,252<br>27,321	4.6%<br>2.6%	<0.001	0.106
1<br>2	2186-5	Not Hispanic or Latino		33,188<br>659,375	67.8%<br>63.1%	<0.001	0.098
1<br>2	2135-2	Hispanic or Latino		4,151<br>94,136	8.5%<br>9.0%	<0.001	0.019
1<br>2	2054-5	Black or African American		6,265<br>178,267	12.8%<br>17.1%	<0.001	0.120
1<br>2	M	Male		20,720<br>541,314	42.3%<br>51.8%	<0.001	0.191
1<br>2	2028-9	Asian		1,204<br>41,822	2.5%<br>4.0%	<0.001	0.087

Diagnosis

Group			Mean ± SD	Patients	% of Group	P-Value	SMD
1<br>2	Z55-Z65	Persons with potential health hazards related to socioeconomic and		686<br>12,021	1.4%<br>1.2%	<0.001	0.022

© 2024 Wang L et al. *JAMA Network Open.*

		psychosocial circumstances				
1<br>2	E66	Overweight and obesity	18,401<br>166,445	37.6%<br>15.9%	<0.001	0.504
1<br>2	Z68.3	Body mass index [BMI] 30-39, adult	4,204<br>43,128	8.6%<br>4.1%	<0.001	0.183
1<br>2	Z68.4	Body mass index [BMI] 40 or greater, adult	3,886<br>33,396	7.9%<br>3.2%	<0.001	0.208
1<br>2	Z68.25	Body mass index [BMI] 25.0-25.9, adult	157<br>3,510	0.3%<br>0.3%	0.563	0.003
1<br>2	Z68.26	Body mass index [BMI] 26.0-26.9, adult	171<br>3,632	0.3%<br>0.3%	0.957	<0.001
1<br>2	Z68.27	Body mass index [BMI] 27.0-27.9, adult	288<br>4,209	0.6%<br>0.4%	<0.001	0.026
1<br>2	Z68.28	Body mass index [BMI] 28.0-28.9, adult	344<br>4,508	0.7%<br>0.4%	<0.001	0.036
1<br>2	Z68.29	Body mass index [BMI] 29.0-29.9, adult	398<br>4,843	0.8%<br>0.5%	<0.001	0.044
1<br>2	Z80	Family history of primary malignant neoplasm	2,042<br>19,398	4.2%<br>1.9%	<0.001	0.136
1<br>2	Z15.0	Genetic susceptibility to malignant neoplasm	24<br>156	0.0%<br>0.0%	<0.001	0.019
1<br>2	Z12	Encounter for screening for malignant neoplasms	12,272<br>105,217	25.1%<br>10.1%	<0.001	0.402
1<br>2	Z85	Personal history of malignant neoplasm	1,239<br>38,294	2.5%<br>3.7%	<0.001	0.066
1<br>2	Z98.84	Bariatric surgery status	632<br>5,136	1.3%<br>0.5%	<0.001	0.085
1<br>2	E66.0	Obesity due to excess calories	9,157<br>69,998	18.7%<br>6.7%	<0.001	0.366
1<br>2	E66.2	Morbid (severe) obesity with alveolar hypoventilation	150<br>3,643	0.3%<br>0.3%	0.118	0.007

1<br>2	E66.3	Overweight		1,010<br>6,796	2.1%<br>0.7%	<0.00<br>1	0.122
1<br>2	E66.8	Other obesity		235<br>1,298	0.5%<br>0.1%	<0.00<br>1	0.065
1<br>2	E66.9	Obesity, unspecified		13,805<br>118,555	28.2%<br>11.3%	<0.00<br>1	0.433
1<br>2	Z79.89<br>0	Hormone replacement therapy		147<br>1,533	0.3%<br>0.1%	<0.00<br>1	0.032
1<br>2	Z92.23	Personal history of estrogen therapy		10<br>128	0.0%<br>0.0%	0.116	0.006
1<br>2	D32	Benign neoplasm of meninges		90<br>3,214	0.2%<br>0.3%	<0.00<br>1	0.025
1<br>2	S00-<br>S09	Injuries to the head		2,121<br>58,791	4.3%<br>5.6%	<0.00<br>1	0.060
1<br>2	Z80.3	Family history of malignant neoplasm of breast		804<br>5,952	1.6%<br>0.6%	<0.00<br>1	0.103
**Procedure**							
Group			Mean ± SD	Patients	% of Group	P-Value	SMD
1<br>2	1001	Radiation		138<br>7,649	0.3%<br>0.7%	<0.00<br>1	0.063
**Medication**							
Group			Mean ± SD	Patients	% of Group	P-Value	SMD
1<br>2	A10B<br>A	Biguanides		27,075<br>199,802	55.3%<br>19.1%	<0.00<br>1	0.806
1<br>2	A10B<br>B	Sulfonylureas		14,077<br>125,703	28.7%<br>12.0%	<0.00<br>1	0.424
1<br>2	A10B<br>F	Alpha glucosidase inhibitors		229<br>1,718	0.5%<br>0.2%	<0.00<br>1	0.054
1<br>2	A10B<br>G	Thiazolidinediones		4,107<br>35,435	8.4%<br>3.4%	<0.00<br>1	0.213
1<br>2	A10B<br>H	Dipeptidyl peptidase 4 (DPP-4) inhibitors		9,485<br>44,595	19.4%<br>4.3%	<0.00<br>1	0.481
1<br>2	A10B<br>K	Sodium-glucose co-transporter 2 (SGLT2) inhibitors		4,808<br>6,447	9.8%<br>0.6%	<0.00<br>1	0.423
1<br>2	A10B<br>X	Other blood glucose lowering drugs, excl. insulins		614<br>7,048	1.3%<br>0.7%	<0.00<br>1	0.059

© 2024 Wang L et al. *JAMA Network Open.*

1<br>2	HS200	CONTRACEPTIVES,SYSTEMIC		1,436<br>5,500	2.9%<br>0.5%	<0.001	0.185

Group 1 (N = 48,518) and group 2 (N = 48,518) characteristics after propensity score matching

Demographics

Group			Mean ± SD	Patients	% of Group	P-Value	SMD
1<br>2	AI	Age at Index	55.9 +/- 11.7<br>56.0 +/- 13.2	48,518<br>48,518	100%<br>100%	0.361	0.006
1<br>2	2106-3	White		32,296<br>33,257	66.6%<br>68.5%	<0.001	0.042
1<br>2	1002-5	American Indian or Alaska Native		197<br>177	0.4%<br>0.4%	0.300	0.007
1<br>2	UNK	Unknown Race		6,995<br>6,626	14.4%<br>13.7%	0.001	0.022
1<br>2	F	Female		25,736<br>26,259	53.0%<br>54.1%	0.001	0.022
1<br>2	2076-8	Native Hawaiian or Other Pacific Islander		205<br>154	0.4%<br>0.3%	0.007	0.017
1<br>2	UN	Unknown Gender		2,182<br>2,018	4.5%<br>4.2%	0.010	0.017
1<br>2	2186-5	Not Hispanic or Latino		32,879<br>33,219	67.8%<br>68.5%	0.019	0.015
1<br>2	2135-2	Hispanic or Latino		4,118<br>4,081	8.5%<br>8.4%	0.669	0.003
1<br>2	2054-5	Black or African American		6,222<br>5,915	12.8%<br>12.2%	0.003	0.019
1<br>2	M	Male		20,600<br>20,241	42.5%<br>41.7%	0.020	0.015
1<br>2	2028-9	Asian		1,195<br>1,067	2.5%<br>2.2%	0.006	0.017

Diagnosis

Group			Mean ± SD	Patients	% of Group	P-Value	SMD
1<br>2	Z55-Z65	Persons with potential health hazards related to socioeconomic and psychosocial circumstances		681<br>570	1.4%<br>1.2%	0.002	0.020

© 2024 Wang L et al. *JAMA Network Open.*

1<br>2	E66	Overweight and obesity	18,033<br>18,054	37.2%<br>37.2%	0.889	0.001
1<br>2	Z68.3	Body mass index [BMI] 30-39, adult	4,068<br>3,696	8.4%<br>7.6%	<0.001	0.028
1<br>2	Z68.4	Body mass index [BMI] 40 or greater, adult	3,789<br>3,420	7.8%<br>7.0%	<0.001	0.029
1<br>2	Z68.25	Body mass index [BMI] 25.0-25.9, adult	156<br>140	0.3%<br>0.3%	0.352	0.006
1<br>2	Z68.26	Body mass index [BMI] 26.0-26.9, adult	171<br>153	0.4%<br>0.3%	0.317	0.006
1<br>2	Z68.27	Body mass index [BMI] 27.0-27.9, adult	283<br>237	0.6%<br>0.5%	0.043	0.013
1<br>2	Z68.28	Body mass index [BMI] 28.0-28.9, adult	334<br>290	0.7%<br>0.6%	0.077	0.011
1<br>2	Z68.29	Body mass index [BMI] 29.0-29.9, adult	386<br>354	0.8%<br>0.7%	0.238	0.008
1<br>2	Z80	Family history of primary malignant neoplasm	1,995<br>1,848	4.1%<br>3.8%	0.016	0.016
1<br>2	Z15.0	Genetic susceptibility to malignant neoplasm	23<br>10	0.0%<br>0.0%	0.024	0.015
1<br>2	Z12	Encounter for screening for malignant neoplasms	11,961<br>11,622	24.7%<br>24.0%	0.011	0.016
1<br>2	Z85	Personal history of malignant neoplasm	1,232<br>1,084	2.5%<br>2.2%	0.002	0.020
1<br>2	Z98.84	Bariatric surgery status	614<br>566	1.3%<br>1.2%	0.160	0.009
1<br>2	E66.0	Obesity due to excess calories	8,936<br>8,880	18.4%<br>18.3%	0.642	0.003
1<br>2	E66.2	Morbid (severe) obesity with alveolar hypoventilation	149<br>115	0.3%<br>0.2%	0.036	0.013
1<br>2	E66.3	Overweight	981<br>888	2.0%<br>1.8%	0.030	0.014

1<br>2	E66.8	Other obesity		222<br>208	0.5%<br>0.4%	0.499	0.004
1<br>2	E66.9	Obesity, unspecified		13,501<br>13,594	27.8%<br>28.0%	0.506	0.004
1<br>2	Z79.89<br>0	Hormone replacement therapy		146<br>124	0.3%<br>0.3%	0.180	0.009
1<br>2	Z92.23	Personal history of estrogen therapy		10<br>13	0.0%<br>0.0%	0.532	0.004
1<br>2	D32	Benign neoplasm of meninges		90<br>79	0.2%<br>0.2%	0.397	0.005
1<br>2	S00-<br>S09	Injuries to the head		2,108<br>1,800	4.3%<br>3.7%	<0.00<br>1	0.032
1<br>2	Z80.3	Family history of malignant neoplasm of breast		783<br>747	1.6%<br>1.5%	0.354	0.006
**Procedure**							
Group			Mean ± SD	Patients	% of Group	P-Value	SMD
1<br>2	1001	Radiation		138<br>96	0.3%<br>0.2%	0.006	0.018
**Medication**							
Group			Mean ± SD	Patients	% of Group	P-Value	SMD
1<br>2	A10B<br>A	Biguanides		26,612<br>27,037	54.8%<br>55.7%	0.006	0.018
1<br>2	A10B<br>B	Sulfonylureas		13,794<br>14,089	28.4%<br>29.0%	0.036	0.013
1<br>2	A10B<br>F	Alpha glucosidase inhibitors		221<br>187	0.5%<br>0.4%	0.092	0.011
1<br>2	A10B<br>G	Thiazolidinediones		4,037<br>4,134	8.3%<br>8.5%	0.262	0.007
1<br>2	A10B<br>H	Dipeptidyl peptidase 4 (DPP-4) inhibitors		9,126<br>9,152	18.8%<br>18.9%	0.831	0.001
1<br>2	A10B<br>K	Sodium-glucose co-transporter 2 (SGLT2) inhibitors		4,364<br>3,874	9.0%<br>8.0%	<0.00<br>1	0.036
1<br>2	A10B<br>X	Other blood glucose lowering drugs, excl. insulins		606<br>591	1.2%<br>1.2%	0.663	0.003
1<br>2	HS200	CONTRACEPTIVE S,SYSTEMIC		1,385<br>1,273	2.9%<br>2.6%	0.028	0.014

© 2024 Wang L et al. *JAMA Network Open.*

eTable 13. Characteristics of the GLP-1RA/no insulin group and insulin/no GLP-1RA group before and after matched for covariates related to multiple myeloma for the study populations of patients with T2D and no history of any OAC

Group 1 (N = 48,983) and group 2 (N = 1,044,745) characteristics before propensity score matching							
**Demographics**							
Group			Mean ± SD	Patients	% of Group	P-Value	SMD
1<br>2	AI	Age at Index	55.9 +/- 11.7<br>61.8 +/- 15.9	48,983<br>1,044,745	100%<br>100%	<0.001	0.420
1<br>2	2106-3	White		32,592<br>633,989	66.5%<br>60.7%	<0.001	0.122
1<br>2	1002-5	American Indian or Alaska Native		199<br>3,443	0.4%<br>0.3%	0.004	0.013
1<br>2	UNK	Unknown Race		7,099<br>142,470	14.5%<br>13.6%	<0.001	0.025
1<br>2	F	Female		26,011<br>476,110	53.1%<br>45.6%	<0.001	0.151
1<br>2	2076-8	Native Hawaiian or Other Pacific Islander		205<br>10,677	0.4%<br>1.0%	<0.001	0.071
1<br>2	UN	Unknown Gender		2,252<br>27,321	4.6%<br>2.6%	<0.001	0.106
1<br>2	2186-5	Not Hispanic or Latino		33,188<br>659,375	67.8%<br>63.1%	<0.001	0.098
1<br>2	2135-2	Hispanic or Latino		4,151<br>94,136	8.5%<br>9.0%	<0.001	0.019
1<br>2	2054-5	Black or African American		6,265<br>178,267	12.8%<br>17.1%	<0.001	0.120
1<br>2	M	Male		20,720<br>541,314	42.3%<br>51.8%	<0.001	0.191
1<br>2	2028-9	Asian		1,204<br>41,822	2.5%<br>4.0%	<0.001	0.087
**Diagnosis**							
Group			Mean ± SD	Patients	% of Group	P-Value	SMD
1<br>2	Z55-Z65	Persons with potential health hazards related to socioeconomic and		686<br>12,021	1.4%<br>1.2%	<0.001	0.022

© 2024 Wang L et al. *JAMA Network Open.*

		psychosocial circumstances				
1<br>2	E66	Overweight and obesity	18,401<br>166,445	37.6%<br>15.9%	<0.001	0.504
1<br>2	Z68.3	Body mass index [BMI] 30-39, adult	4,204<br>43,128	8.6%<br>4.1%	<0.001	0.183
1<br>2	Z68.4	Body mass index [BMI] 40 or greater, adult	3,886<br>33,396	7.9%<br>3.2%	<0.001	0.208
1<br>2	Z68.25	Body mass index [BMI] 25.0-25.9, adult	157<br>3,510	0.3%<br>0.3%	0.563	0.003
1<br>2	Z68.26	Body mass index [BMI] 26.0-26.9, adult	171<br>3,632	0.3%<br>0.3%	0.957	<0.001
1<br>2	Z68.27	Body mass index [BMI] 27.0-27.9, adult	288<br>4,209	0.6%<br>0.4%	<0.001	0.026
1<br>2	Z68.28	Body mass index [BMI] 28.0-28.9, adult	344<br>4,508	0.7%<br>0.4%	<0.001	0.036
1<br>2	Z68.29	Body mass index [BMI] 29.0-29.9, adult	398<br>4,843	0.8%<br>0.5%	<0.001	0.044
1<br>2	Z80	Family history of primary malignant neoplasm	2,042<br>19,398	4.2%<br>1.9%	<0.001	0.136
1<br>2	Z15.0	Genetic susceptibility to malignant neoplasm	24<br>156	0.0%<br>0.0%	<0.001	0.019
1<br>2	Z12	Encounter for screening for malignant neoplasms	12,272<br>105,217	25.1%<br>10.1%	<0.001	0.402
1<br>2	Z85	Personal history of malignant neoplasm	1,239<br>38,294	2.5%<br>3.7%	<0.001	0.066
1<br>2	Z98.84	Bariatric surgery status	632<br>5,136	1.3%<br>0.5%	<0.001	0.085
1<br>2	E66.0	Obesity due to excess calories	9,157<br>69,998	18.7%<br>6.7%	<0.001	0.366
1<br>2	E66.2	Morbid (severe) obesity with alveolar hypoventilation	150<br>3,643	0.3%<br>0.3%	0.118	0.007

© 2024 Wang L et al. *JAMA Network Open.*

1<br>2	E66.3	Overweight		1,010<br>6,796	2.1%<br>0.7%	<0.001	0.122
1<br>2	E66.8	Other obesity		235<br>1,298	0.5%<br>0.1%	<0.001	0.065
1<br>2	E66.9	Obesity, unspecified		13,805<br>118,555	28.2%<br>11.3%	<0.001	0.433
1<br>2	Z80.7	Family history of other malignant neoplasms of lymphoid, hematopoietic and related tissues		20<br>319	0.0%<br>0.0%	0.206	0.005
1<br>2	D47.2	Monoclonal gammopathy		96<br>2,627	0.2%<br>0.3%	0.016	0.012
1<br>2	C90.3	Solitary plasmacytoma		10<br>50	0.0%<br>0.0%	<0.001	0.014
1<br>2	D45	Polycythemia vera		82<br>1,367	0.2%<br>0.1%	0.030	0.009
1<br>2	D46	Myelodysplastic syndromes		19<br>2,139	0.0%<br>0.2%	<0.001	0.048
1<br>2	D47	Other neoplasms of uncertain behavior of lymphoid, hematopoietic and related tissue		281<br>7,366	0.6%<br>0.7%	0.001	0.016
**Medication**							
Group			Mean ± SD	Patients	% of Group	P-Value	SMD
1<br>2	A10BA	Biguanides		27,075<br>199,802	55.3%<br>19.1%	<0.001	0.806
1<br>2	A10BB	Sulfonylureas		14,077<br>125,703	28.7%<br>12.0%	<0.001	0.424
1<br>2	A10BF	Alpha glucosidase inhibitors		229<br>1,718	0.5%<br>0.2%	<0.001	0.054
1<br>2	A10BG	Thiazolidinediones		4,107<br>35,435	8.4%<br>3.4%	<0.001	0.213
1<br>2	A10BH	Dipeptidyl peptidase 4 (DPP-4) inhibitors		9,485<br>44,595	19.4%<br>4.3%	<0.001	0.481
1<br>2	A10BK	Sodium-glucose co-transporter 2 (SGLT2) inhibitors		4,808<br>6,447	9.8%<br>0.6%	<0.001	0.423

© 2024 Wang L et al. *JAMA Network Open.*

1<br>2	A10B<br>X	Other blood glucose lowering drugs, excl. insulins		614<br>7,048	1.3%<br>0.7%	<0.00<br>1	0.059

Group 1 (N = 48,527) and group 2 (N = 48,527) characteristics after propensity score matching

Demographics

Group			Mean ± SD	Patients	% of Group	P-Value	SMD
1<br>2	AI	Age at Index	55.9 +/- 11.7<br>56.1 +/- 13.0	48,527<br>48,527	100%<br>100%	0.057	0.012
1<br>2	2106-3	White		32,296<br>33,417	66.6%<br>68.9%	<0.00<br>1	0.049
1<br>2	1002-5	American Indian or Alaska Native		197<br>150	0.4%<br>0.3%	0.011	0.016
1<br>2	UNK	Unknown Race		6,995<br>6,585	14.4%<br>13.6%	<0.00<br>1	0.024
1<br>2	F	Female		25,751<br>26,135	53.1%<br>53.9%	0.013	0.016
1<br>2	2076-8	Native Hawaiian or Other Pacific Islander		205<br>147	0.4%<br>0.3%	0.002	0.020
1<br>2	UN	Unknown Gender		2,183<br>2,029	4.5%<br>4.2%	0.015	0.016
1<br>2	2186-5	Not Hispanic or Latino		32,881<br>33,326	67.8%<br>68.7%	0.002	0.020
1<br>2	2135-2	Hispanic or Latino		4,122<br>3,926	8.5%<br>8.1%	0.023	0.015
1<br>2	2054-5	Black or African American		6,230<br>5,926	12.8%<br>12.2%	0.003	0.019
1<br>2	M	Male		20,593<br>20,363	42.4%<br>42.0%	0.135	0.010
1<br>2	2028-9	Asian		1,197<br>1,015	2.5%<br>2.1%	<0.00<br>1	0.025

Diagnosis

Group			Mean ± SD	Patients	% of Group	P-Value	SMD
1<br>2	Z55-<br>Z65	Persons with potential health hazards related to socioeconomic and psychosocial circumstances		682<br>582	1.4%<br>1.2%	0.005	0.018

© 2024 Wang L et al. *JAMA Network Open.*

1<br>2	E66	Overweight and obesity	18,046<br>18,090	37.2%<br>37.3%	0.770	0.002
1<br>2	Z68.3	Body mass index [BMI] 30-39, adult	4,072<br>3,739	8.4%<br>7.7%	<0.001	0.025
1<br>2	Z68.4	Body mass index [BMI] 40 or greater, adult	3,794<br>3,424	7.8%<br>7.1%	<0.001	0.029
1<br>2	Z68.25	Body mass index [BMI] 25.0-25.9, adult	156<br>135	0.3%<br>0.3%	0.218	0.008
1<br>2	Z68.26	Body mass index [BMI] 26.0-26.9, adult	171<br>160	0.4%<br>0.3%	0.545	0.004
1<br>2	Z68.27	Body mass index [BMI] 27.0-27.9, adult	284<br>226	0.6%<br>0.5%	0.010	0.017
1<br>2	Z68.28	Body mass index [BMI] 28.0-28.9, adult	334<br>282	0.7%<br>0.6%	0.036	0.013
1<br>2	Z68.29	Body mass index [BMI] 29.0-29.9, adult	385<br>305	0.8%<br>0.6%	0.002	0.020
1<br>2	Z80	Family history of primary malignant neoplasm	1,997<br>1,823	4.1%<br>3.8%	0.004	0.018
1<br>2	Z15.0	Genetic susceptibility to malignant neoplasm	22<br>12	0.0%<br>0.0%	0.086	0.011
1<br>2	Z12	Encounter for screening for malignant neoplasms	11,973<br>11,574	24.7%<br>23.9%	0.003	0.019
1<br>2	Z85	Personal history of malignant neoplasm	1,232<br>1,052	2.5%<br>2.2%	<0.001	0.024
1<br>2	Z98.84	Bariatric surgery status	614<br>568	1.3%<br>1.2%	0.178	0.009
1<br>2	E66.0	Obesity due to excess calories	8,943<br>8,911	18.4%<br>18.4%	0.791	0.002
1<br>2	E66.2	Morbid (severe) obesity with alveolar hypoventilation	147<br>157	0.3%<br>0.3%	0.566	0.004
1<br>2	E66.3	Overweight	984<br>850	2.0%<br>1.8%	0.002	0.020

1<br>2	E66.8	Other obesity		226<br>175	0.5%<br>0.4%	0.011	0.016
1<br>2	E66.9	Obesity, unspecified		13,515<br>13,699	27.9%<br>28.2%	0.189	0.008
1<br>2	Z80.7	Family history of other malignant neoplasms of lymphoid, hematopoietic and related tissues		19<br>20	0.0%<br>0.0%	0.873	0.001
1<br>2	D47.2	Monoclonal gammopathy		96<br>71	0.2%<br>0.1%	0.053	0.012
1<br>2	C90.3	Solitary plasmacytoma		10<br>10	0.0%<br>0.0%	1	<0.001
1<br>2	D45	Polycythemia vera		79<br>81	0.2%<br>0.2%	0.874	0.001
1<br>2	D46	Myelodysplastic syndromes		19<br>43	0.0%<br>0.1%	0.002	0.020
1<br>2	D47	Other neoplasms of uncertain behavior of lymphoid, hematopoietic and related tissue		278<br>238	0.6%<br>0.5%	0.077	0.011
**Medication**							
Group			Mean ± SD	Patients	% of Group	P-Value	SMD
1<br>2	A10B<br>A	Biguanides		26,622<br>27,044	54.9%<br>55.7%	0.006	0.017
1<br>2	A10B<br>B	Sulfonylureas		13,797<br>14,109	28.4%<br>29.1%	0.027	0.014
1<br>2	A10B<br>F	Alpha glucosidase inhibitors		222<br>179	0.5%<br>0.4%	0.031	0.014
1<br>2	A10B<br>G	Thiazolidinediones		4,037<br>4,097	8.3%<br>8.4%	0.487	0.004
1<br>2	A10B<br>H	Dipeptidyl peptidase 4 (DPP-4) inhibitors		9,133<br>9,339	18.8%<br>19.2%	0.092	0.011
1<br>2	A10B<br>K	Sodium-glucose co-transporter 2 (SGLT2) inhibitors		4,366<br>3,914	9.0%<br>8.1%	<0.001	0.033
1<br>2	A10B<br>X	Other blood glucose lowering drugs, excl. insulins		606<br>572	1.2%<br>1.2%	0.319	0.006

© 2024 Wang L et al. *JAMA Network Open.*

eTable 14. Characteristics of the GLP-1RA/no metformin group and metformin/no GLP-1RA group before and after matched for covariates related to esophageal cancer for the study populations of patients with T2D and no history of any OAC

Group 1 (N = 32,365) and group 2 (N = 856,160) characteristics before propensity score matching							
**Demographics**							
Group			Mean ± SD	Patients	% of Group	P-Value	SMD
1<br>2	AI	Age at Index	59.0 +/- 12.4<br>60.1 +/- 14.0	32,365<br>856,160	100%<br>100%	<0.001	0.080
1<br>2	2106-3	White		21,231<br>515,868	65.6%<br>60.3%	<0.001	0.111
1<br>2	1002-5	American Indian or Alaska Native		98<br>3,080	0.3%<br>0.4%	0.092	0.010
1<br>2	UNK	Unknown Race		4,272<br>119,036	13.2%<br>13.9%	<0.001	0.021
1<br>2	F	Female		17,278<br>399,923	53.4%<br>46.7%	<0.001	0.134
1<br>2	2076-8	Native Hawaiian or Other Pacific Islander		172<br>5,884	0.5%<br>0.7%	0.001	0.020
1<br>2	UN	Unknown Gender		1,670<br>19,592	5.2%<br>2.3%	<0.001	0.152
1<br>2	2186-5	Not Hispanic or Latino		22,299<br>528,686	68.9%<br>61.8%	<0.001	0.151
1<br>2	2135-2	Hispanic or Latino		2,660<br>91,303	8.2%<br>10.7%	<0.001	0.084
1<br>2	2054-5	Black or African American		5,107<br>141,606	15.8%<br>16.5%	<0.001	0.021
1<br>2	M	Male		13,417<br>436,645	41.5%<br>51.0%	<0.001	0.192
1<br>2	2028-9	Asian		681<br>35,540	2.1%<br>4.2%	<0.001	0.118
**Diagnosis**							
Group			Mean ± SD	Patients	% of Group	P-Value	SMD
1<br>2	Z55-Z65	Persons with potential health hazards related to socioeconomic and		422<br>10,310	1.3%<br>1.2%	0.107	0.009

© 2024 Wang L et al. *JAMA Network Open.*

		psychosocial circumstances				
1<br>2	E66	Overweight and obesity	11,418<br>149,974	35.3%<br>17.5%	<0.00<br>1	0.411
1<br>2	Z68.3	Body mass index [BMI] 30-39, adult	3,285<br>32,977	10.1%<br>3.9%	<0.00<br>1	0.249
1<br>2	Z68.4	Body mass index [BMI] 40 or greater, adult	3,012<br>23,535	9.3%<br>2.7%	<0.00<br>1	0.278
1<br>2	Z68.25	Body mass index [BMI] 25.0-25.9, adult	119<br>2,293	0.4%<br>0.3%	0.001	0.018
1<br>2	Z68.26	Body mass index [BMI] 26.0-26.9, adult	150<br>2,433	0.5%<br>0.3%	<0.00<br>1	0.029
1<br>2	Z68.27	Body mass index [BMI] 27.0-27.9, adult	209<br>2,942	0.6%<br>0.3%	<0.00<br>1	0.043
1<br>2	Z68.28	Body mass index [BMI] 28.0-28.9, adult	264<br>3,317	0.8%<br>0.4%	<0.00<br>1	0.055
1<br>2	Z68.29	Body mass index [BMI] 29.0-29.9, adult	299<br>3,753	0.9%<br>0.4%	<0.00<br>1	0.059
1<br>2	F10	Alcohol related disorders	460<br>21,150	1.4%<br>2.5%	<0.00<br>1	0.076
1<br>2	Z80	Family history of primary malignant neoplasm	1,222<br>16,133	3.8%<br>1.9%	<0.00<br>1	0.114
1<br>2	Z15.0	Genetic susceptibility to malignant neoplasm	18<br>140	0.1%<br>0.0%	<0.00<br>1	0.021
1<br>2	Z12	Encounter for screening for malignant neoplasms	6,223<br>114,996	19.2%<br>13.4%	<0.00<br>1	0.157
1<br>2	Z85	Personal history of malignant neoplasm	1,111<br>23,231	3.4%<br>2.7%	<0.00<br>1	0.042
1<br>2	Z98.84	Bariatric surgery status	534<br>2,989	1.6%<br>0.3%	<0.00<br>1	0.131
1<br>2	F17	Nicotine dependence	2,399<br>77,335	7.4%<br>9.0%	<0.00<br>1	0.059

© 2024 Wang L et al. *JAMA Network Open.*

1<br>2	E66.0	Obesity due to excess calories	5,910<br>56,178	18.3%<br>6.6%	<0.001	0.361
1<br>2	E66.2	Morbid (severe) obesity with alveolar hypoventilation	230<br>2,051	0.7%<br>0.2%	<0.001	0.069
1<br>2	E66.3	Overweight	535<br>7,794	1.7%<br>0.9%	<0.001	0.066
1<br>2	E66.8	Other obesity	193<br>1,084	0.6%<br>0.1%	<0.001	0.078
1<br>2	E66.9	Obesity, unspecified	8,150<br>113,380	25.2%<br>13.2%	<0.001	0.307
1<br>2	Z72.0	Tobacco use	588<br>11,927	1.8%<br>1.4%	<0.001	0.034
1<br>2	K21	Gastro-esophageal reflux disease	6,710<br>115,166	20.7%<br>13.5%	<0.001	0.194
1<br>2	K22.7	Barrett's esophagus	244<br>3,863	0.8%<br>0.5%	<0.001	0.039
1<br>2	K22.0	Achalasia of cardia	30<br>433	0.1%<br>0.1%	0.001	0.016
1<br>2	D69.41	Evans syndrome	0<br>18	0%<br>0.0%	0.409	0.006
1<br>2	D50.1	Sideropenic dysphagia	57<br>573	0.2%<br>0.1%	<0.001	0.031
1<br>2	S27.81	Injury of esophagus (thoracic part)	10<br>33	0.0%<br>0.0%	<0.001	0.021
1<br>2	C34	Malignant neoplasm of bronchus and lung	99<br>4,460	0.3%<br>0.5%	<0.001	0.034
1<br>2	C06	Malignant neoplasm of other and unspecified parts of mouth	10<br>451	0.0%<br>0.1%	0.091	0.011
1<br>2	C32	Malignant neoplasm of larynx	13<br>741	0.0%<br>0.1%	0.005	0.018
1<br>2	B97.7	Papillomavirus as the cause of diseases classified elsewhere	56<br>908	0.2%<br>0.1%	<0.001	0.018
1<br>2	R87.81	High risk human papillomavirus (HPV) DNA test positive from female genital organs	59<br>928	0.2%<br>0.1%	<0.001	0.019

© 2024 Wang L et al. *JAMA Network Open.*

1<br>2	Z80.0	Family history of malignant neoplasm of digestive organs		413<br>6,255	1.3%<br>0.7%	<0.00<br>1	0.055
1<br>2	D10	Benign neoplasm of mouth and pharynx		38<br>1,091	0.1%<br>0.1%	0.619	0.003
1<br>2	D13.0	Benign neoplasm of esophagus		10<br>171	0.0%<br>0.0%	0.176	0.007

Medication

Group			Mean ± SD	Patients	% of Group	P-Value	SMD
1<br>2	A10B<br>B	Sulfonylureas		5,464<br>69,045	16.9%<br>8.1%	<0.00<br>1	0.269
1<br>2	A10B<br>F	Alpha glucosidase inhibitors		124<br>876	0.4%<br>0.1%	<0.00<br>1	0.057
1<br>2	A10B<br>G	Thiazolidinediones		1,960<br>20,400	6.1%<br>2.4%	<0.00<br>1	0.183
1<br>2	A10B<br>H	Dipeptidyl peptidase 4 (DPP-4) inhibitors		3,547<br>17,113	11.0%<br>2.0%	<0.00<br>1	0.370
1<br>2	A10B<br>K	Sodium-glucose co-transporter 2 (SGLT2) inhibitors		1,839<br>3,371	5.7%<br>0.4%	<0.00<br>1	0.312
1<br>2	A10B<br>X	Other blood glucose lowering drugs, excl. insulins		672<br>3,839	2.1%<br>0.4%	<0.00<br>1	0.146
1<br>2	A10A	INSULINS AND ANALOGUES		14,131<br>159,901	43.7%<br>18.7%	<0.00<br>1	0.560

Group 1 (N = 32,263) and group 2 (N = 32,263) characteristics after propensity score matching

Demographics

Group			Mean ± SD	Patients	% of Group	P-Value	SMD
1<br>2	AI	Age at Index	59.0 +/- 12.4<br>59.0 +/- 13.2	32,263<br>32,263	100%<br>100%	0.721	0.003
1<br>2	2106-3	White		21,161<br>21,731	65.6%<br>67.4%	<0.00<br>1	0.037
1<br>2	1002-5	American Indian or Alaska Native		98<br>100	0.3%<br>0.3%	0.887	0.001
1<br>2	UNK	Unknown Race		4,255<br>4,179	13.2%<br>13.0%	0.375	0.007
1<br>2	F	Female		17,209<br>17,472	53.3%<br>54.2%	0.038	0.016

© 2024 Wang L et al. *JAMA Network Open.*

1<br>2	2076-8	Native Hawaiian or Other Pacific Islander		172<br>147	0.5%<br>0.5%	0.161	0.011
1<br>2	UN	Unknown Gender		1,656<br>1,696	5.1%<br>5.3%	0.478	0.006
1<br>2	2186-5	Not Hispanic or Latino		22,225<br>22,564	68.9%<br>69.9%	0.004	0.023
1<br>2	2135-2	Hispanic or Latino		2,651<br>2,490	8.2%<br>7.7%	0.019	0.018
1<br>2	2054-5	Black or African American		5,092<br>4,779	15.8%<br>14.8%	0.001	0.027
1<br>2	M	Male		13,398<br>13,095	41.5%<br>40.6%	0.015	0.019
1<br>2	2028-9	Asian		681<br>590	2.1%<br>1.8%	0.010	0.020

Diagnosis

Group			Mean ± SD	Patients	% of Group	P-Value	SMD
1<br>2	Z55-<br>Z65	Persons with potential health hazards related to socioeconomic and psychosocial circumstances		419<br>339	1.3%<br>1.1%	0.003	0.023
1<br>2	E66	Overweight and obesity		11,341<br>11,246	35.2%<br>34.9%	0.433	0.006
1<br>2	Z68.3	Body mass index [BMI] 30-39, adult		3,247<br>3,224	10.1%<br>10.0%	0.763	0.002
1<br>2	Z68.4	Body mass index [BMI] 40 or greater, adult		2,969<br>2,835	9.2%<br>8.8%	0.065	0.015
1<br>2	Z68.25	Body mass index [BMI] 25.0-25.9, adult		118<br>107	0.4%<br>0.3%	0.463	0.006
1<br>2	Z68.26	Body mass index [BMI] 26.0-26.9, adult		149<br>115	0.5%<br>0.4%	0.036	0.017
1<br>2	Z68.27	Body mass index [BMI] 27.0-27.9, adult		207<br>180	0.6%<br>0.6%	0.169	0.011

© 2024 Wang L et al. *JAMA Network Open.*

1<br>2	Z68.28	Body mass index [BMI] 28.0-28.9, adult	261<br>231	0.8%<br>0.7%	0.175	0.011
1<br>2	Z68.29	Body mass index [BMI] 29.0-29.9, adult	297<br>256	0.9%<br>0.8%	0.080	0.014
1<br>2	F10	Alcohol related disorders	459<br>323	1.4%<br>1.0%	<0.00<br>1	0.039
1<br>2	Z80	Family history of primary malignant neoplasm	1,205<br>1,120	3.7%<br>3.5%	0.073	0.014
1<br>2	Z15.0	Genetic susceptibility to malignant neoplasm	18<br>11	0.1%<br>0.0%	0.194	0.010
1<br>2	Z12	Encounter for screening for malignant neoplasms	6,177<br>5,550	19.1%<br>17.2%	<0.00<br>1	0.050
1<br>2	Z85	Personal history of malignant neoplasm	1,106<br>1,039	3.4%<br>3.2%	0.141	0.012
1<br>2	Z98.84	Bariatric surgery status	520<br>527	1.6%<br>1.6%	0.827	0.002
1<br>2	F17	Nicotine dependence	2,388<br>2,081	7.4%<br>6.5%	<0.00<br>1	0.037
1<br>2	E66.0	Obesity due to excess calories	5,847<br>5,817	18.1%<br>18.0%	0.759	0.002
1<br>2	E66.2	Morbid (severe) obesity with alveolar hypoventilation	227<br>208	0.7%<br>0.6%	0.361	0.007
1<br>2	E66.3	Overweight	529<br>478	1.6%<br>1.5%	0.105	0.013
1<br>2	E66.8	Other obesity	190<br>190	0.6%<br>0.6%	1	<0.001
1<br>2	E66.9	Obesity, unspecified	8,097<br>7,950	25.1%<br>24.6%	0.181	0.011
1<br>2	Z72.0	Tobacco use	584<br>507	1.8%<br>1.6%	0.019	0.019
1<br>2	K21	Gastro-esophageal reflux disease	6,661<br>6,366	20.6%<br>19.7%	0.004	0.023
1<br>2	K22.7	Barrett's esophagus	243<br>231	0.8%<br>0.7%	0.580	0.004
1<br>2	K22.0	Achalasia of cardia	30<br>21	0.1%<br>0.1%	0.207	0.010

© 2024 Wang L et al. *JAMA Network Open.*

1<br>2	D69.41	Evans syndrome		0<br>10	0%<br>0.0%	0.002	0.025
1<br>2	D50.1	Sideropenic dysphagia		56<br>51	0.2%<br>0.2%	0.629	0.004
1<br>2	S27.81	Injury of esophagus (thoracic part)		10<br>10	0.0%<br>0.0%	1	<0.001
1<br>2	C34	Malignant neoplasm of bronchus and lung		99<br>91	0.3%<br>0.3%	0.561	0.005
1<br>2	C06	Malignant neoplasm of other and unspecified parts of mouth		10<br>11	0.0%<br>0.0%	0.827	0.002
1<br>2	C32	Malignant neoplasm of larynx		13<br>10	0.0%<br>0.0%	0.532	0.005
1<br>2	B97.7	Papillomavirus as the cause of diseases classified elsewhere		56<br>37	0.2%<br>0.1%	0.049	0.016
1<br>2	R87.81	High risk human papillomavirus (HPV) DNA test positive from female genital organs		58<br>50	0.2%<br>0.2%	0.441	0.006
1<br>2	Z80.0	Family history of malignant neoplasm of digestive organs		408<br>356	1.3%<br>1.1%	0.058	0.015
1<br>2	D10	Benign neoplasm of mouth and pharynx		38<br>17	0.1%<br>0.1%	0.005	0.022
1<br>2	D13.0	Benign neoplasm of esophagus		10<br>10	0.0%<br>0.0%	1	<0.001
**Medication**							
Group			Mean ± SD	Patients	% of Group	P-Value	SMD
1<br>2	A10BB	Sulfonylureas		5,424<br>5,348	16.8%<br>16.6%	0.422	0.006
1<br>2	A10BF	Alpha glucosidase inhibitors		124<br>116	0.4%<br>0.4%	0.605	0.004
1<br>2	A10BG	Thiazolidinediones		1,930<br>1,906	6.0%<br>5.9%	0.689	0.003
1<br>2	A10BH	Dipeptidyl peptidase 4 (DPP-4) inhibitors		3,465<br>3,384	10.7%<br>10.5%	0.301	0.008

© 2024 Wang L et al. *JAMA Network Open.*

1<br>2	A10B<br>K	Sodium-glucose co-transporter 2 (SGLT2) inhibitors	1,747<br>1,612	5.4%<br>5.0%	0.017	0.019
1<br>2	A10B<br>X	Other blood glucose lowering drugs, excl. insulins	658<br>603	2.0%<br>1.9%	0.118	0.012
1<br>2	A10A	INSULINS AND ANALOGUES	14,037<br>13,941	43.5%<br>43.2%	0.446	0.006

© 2024 Wang L et al. *JAMA Network Open.*

eTable 15. Characteristics of the GLP-1RA/no metformin group and metformin/no GLP-1RA group before and after matched for covariates related to breast cancer for the study populations of women (age 55 and older) with T2D and no history of any OAC

Group 1 (N = 10,497) and group 2 (N = 245,112) characteristics before propensity score matching

Demographics

Group			Mean ± SD	Patients	% of Group	P-Value	SMD
1<br>2	AI	Age at Index	65.1 +/- 6.8<br>66.2 +/- 7.2	10,497<br>245,112	100%<br>100%	<0.001	0.155
1<br>2	2106-3	White		7,228<br>148,499	68.9%<br>60.6%	<0.001	0.174
1<br>2	1002-5	American Indian or Alaska Native		25<br>782	0.2%<br>0.3%	0.148	0.015
1<br>2	UNK	Unknown Race		883<br>27,764	8.4%<br>11.3%	<0.001	0.098
1<br>2	2076-8	Native Hawaiian or Other Pacific Islander		43<br>1,458	0.4%<br>0.6%	0.015	0.026
1<br>2	UN	Unknown Ethnicity		2,019<br>65,536	19.2%<br>26.7%	<0.001	0.179
1<br>2	2186-5	Not Hispanic or Latino		7,582<br>155,608	72.2%<br>63.5%	<0.001	0.188
1<br>2	2135-2	Hispanic or Latino		896<br>23,968	8.5%<br>9.8%	<0.001	0.043
1<br>2	2054-5	Black or African American		1,881<br>45,751	17.9%<br>18.7%	0.055	0.019
1<br>2	2028-9	Asian		178<br>11,425	1.7%<br>4.7%	<0.001	0.170

Diagnosis

Group			Mean ± SD	Patients	% of Group	P-Value	SMD
1<br>2	Z55-Z65	Persons with potential health hazards related to socioeconomic and psychosocial circumstances		135<br>2,329	1.3%<br>1.0%	0.001	0.032
1<br>2	E66	Overweight and obesity		3,810<br>42,178	36.3%<br>17.2%	<0.001	0.442

© 2024 Wang L et al. *JAMA Network Open.*

1<br>2	Z68.3	Body mass index [BMI] 30-39, adult	859<br>8,046	8.2%<br>3.3%	<0.001	0.212
1<br>2	Z68.4	Body mass index [BMI] 40 or greater, adult	948<br>6,215	9.0%<br>2.5%	<0.001	0.281
1<br>2	Z68.25	Body mass index [BMI] 25.0-25.9, adult	30<br>448	0.3%<br>0.2%	0.017	0.021
1<br>2	Z68.26	Body mass index [BMI] 26.0-26.9, adult	44<br>450	0.4%<br>0.2%	<0.001	0.043
1<br>2	Z68.27	Body mass index [BMI] 27.0-27.9, adult	42<br>567	0.4%<br>0.2%	0.001	0.030
1<br>2	Z68.28	Body mass index [BMI] 28.0-28.9, adult	51<br>611	0.5%<br>0.2%	<0.001	0.039
1<br>2	Z68.29	Body mass index [BMI] 29.0-29.9, adult	51<br>743	0.5%<br>0.3%	0.001	0.029
1<br>2	F10	Alcohol related disorders	67<br>2,158	0.6%<br>0.9%	0.009	0.028
1<br>2	Z80	Family history of primary malignant neoplasm	527<br>6,537	5.0%<br>2.7%	<0.001	0.123
1<br>2	Z15.0	Genetic susceptibility to malignant neoplasm	10<br>52	0.1%<br>0.0%	<0.001	0.031
1<br>2	Z12	Encounter for screening for malignant neoplasms	2,745<br>48,467	26.2%<br>19.8%	<0.001	0.152
1<br>2	Z85	Personal history of malignant neoplasm	369<br>6,840	3.5%<br>2.8%	<0.001	0.041
1<br>2	Z98.84	Bariatric surgery status	212<br>1,194	2.0%<br>0.5%	<0.001	0.138
1<br>2	E66.0	Obesity due to excess calories	1,970<br>15,667	18.8%<br>6.4%	<0.001	0.380
1<br>2	E66.2	Morbid (severe) obesity with alveolar hypoventilation	85<br>642	0.8%<br>0.3%	<0.001	0.075
1<br>2	E66.3	Overweight	169<br>2,054	1.6%<br>0.8%	<0.001	0.070

1<br>2	E66.8	Other obesity		61<br>308	0.6%<br>0.1%	<0.001	0.077
1<br>2	E66.9	Obesity, unspecified		2,717<br>31,871	25.9%<br>13.0%	<0.001	0.330
1<br>2	O92.7	Other and unspecified disorders of lactation		0<br>10	0%<br>0.0%	0.513	0.009
1<br>2	Z79.890	Hormone replacement therapy		62<br>1,053	0.6%<br>0.4%	0.014	0.023
1<br>2	Z92.23	Personal history of estrogen therapy		10<br>105	0.1%<br>0.0%	0.013	0.020
1<br>2	Z98.82	Breast implant status		10<br>69	0.1%<br>0.0%	<0.001	0.027
1<br>2	Z80.3	Family history of malignant neoplasm of breast		253<br>3,111	2.4%<br>1.3%	<0.001	0.085
1<br>2	R92	Abnormal and inconclusive findings on diagnostic imaging of breast		663<br>13,119	6.3%<br>5.4%	<0.001	0.041
1<br>2	D24	Benign neoplasm of breast		57<br>1,303	0.5%<br>0.5%	0.875	0.002
1<br>2	N60	Benign mammary dysplasia		228<br>4,931	2.2%<br>2.0%	0.253	0.011
1<br>2	N62	Hypertrophy of breast		30<br>574	0.3%<br>0.2%	0.286	0.010
1<br>2	N63	Unspecified lump in breast		351<br>7,951	3.3%<br>3.2%	0.571	0.006
1<br>2	N64	Other disorders of breast		425<br>7,633	4.0%<br>3.1%	<0.001	0.050
1<br>2	N61	Inflammatory disorders of breast		43<br>716	0.4%<br>0.3%	0.030	0.020
1<br>2	D05	Carcinoma in situ of breast		14<br>386	0.1%<br>0.2%	0.541	0.006
**Procedure**							
Group			Mean ± SD	Patients	% of Group	P-Value	SMD
1<br>2	C1789	Prosthesis, breast (implantable)		10<br>10	0.1%<br>0.0%	<0.001	0.041

© 2024 Wang L et al. *JAMA Network Open.*

1<br>2	58300	Insertion of intrauterine device (IUD)		10<br>61	0.1%<br>0.0%	<0.00<br>1	0.029
1<br>2	1001	Radiation		28<br>902	0.3%<br>0.4%	0.092	0.018

Medication

Group			Mean ± SD	Patients	% of Group	P-Value	SMD
1<br>2	A10A	INSULINS AND ANALOGUES		4,577<br>42,173	43.6%<br>17.2%	<0.00<br>1	0.599
1<br>2	A10B<br>B	Sulfonylureas		1,853<br>19,472	17.7%<br>7.9%	<0.00<br>1	0.294
1<br>2	A10B<br>F	Alpha glucosidase inhibitors		35<br>272	0.3%<br>0.1%	<0.00<br>1	0.047
1<br>2	A10B<br>G	Thiazolidinediones		643<br>5,777	6.1%<br>2.4%	<0.00<br>1	0.188
1<br>2	A10B<br>H	Dipeptidyl peptidase 4 (DPP-4) inhibitors		1,273<br>5,583	12.1%<br>2.3%	<0.00<br>1	0.388
1<br>2	A10B<br>K	Sodium-glucose co-transporter 2 (SGLT2) inhibitors		569<br>907	5.4%<br>0.4%	<0.00<br>1	0.305
1<br>2	A10B<br>X	Other blood glucose lowering drugs, excl. insulins		229<br>1,121	2.2%<br>0.5%	<0.00<br>1	0.152
1<br>2	HS200	CONTRACEPTIVE S,SYSTEMIC		189<br>2,971	1.8%<br>1.2%	<0.00<br>1	0.048

Group 1 (N = 10,419) and group 2 (N = 10,419) characteristics after propensity score matching

Demographics

Group			Mean ± SD	Patients	% of Group	P-Value	SMD
1<br>2	AI	Age at Index	65.2 +/- 6.8<br>65.0 +/- 6.8	10,419<br>10,419	100%<br>100%	0.069	0.025
1<br>2	2106-3	White		7,159<br>7,326	68.7%<br>70.3%	0.012	0.035
1<br>2	1002-5	American Indian or Alaska Native		25<br>29	0.2%<br>0.3%	0.586	0.008
1<br>2	UNK	Unknown Race		881<br>825	8.5%<br>7.9%	0.157	0.020

© 2024 Wang L et al. *JAMA Network Open.*

Group			Mean ± SD	Patients	% of Group	P-Value	SMD
1<br>2	2076-8	Native Hawaiian or Other Pacific Islander		43<br>36	0.4%<br>0.3%	0.430	0.011
1<br>2	UN	Unknown Ethnicity		2,016<br>1,895	19.3%<br>18.2%	0.032	0.030
1<br>2	2186-5	Not Hispanic or Latino		7,515<br>7,696	72.1%<br>73.9%	0.005	0.039
1<br>2	2135-2	Hispanic or Latino		888<br>828	8.5%<br>7.9%	0.131	0.021
1<br>2	2054-5	Black or African American		1,875<br>1,804	18.0%<br>17.3%	0.197	0.018
1<br>2	2028-9	Asian		178<br>157	1.7%<br>1.5%	0.247	0.016

Diagnosis

Group			Mean ± SD	Patients	% of Group	P-Value	SMD
1<br>2	Z55-Z65	Persons with potential health hazards related to socioeconomic and psychosocial circumstances		132<br>127	1.3%<br>1.2%	0.755	0.004
1<br>2	E66	Overweight and obesity		3,757<br>3,796	36.1%<br>36.4%	0.574	0.008
1<br>2	Z68.3	Body mass index [BMI] 30-39, adult		840<br>844	8.1%<br>8.1%	0.919	0.001
1<br>2	Z68.4	Body mass index [BMI] 40 or greater, adult		921<br>874	8.8%<br>8.4%	0.246	0.016
1<br>2	Z68.25	Body mass index [BMI] 25.0-25.9, adult		30<br>23	0.3%<br>0.2%	0.336	0.013
1<br>2	Z68.26	Body mass index [BMI] 26.0-26.9, adult		42<br>41	0.4%<br>0.4%	0.912	0.002
1<br>2	Z68.27	Body mass index [BMI] 27.0-27.9, adult		40<br>44	0.4%<br>0.4%	0.662	0.006
1<br>2	Z68.28	Body mass index [BMI] 28.0-28.9, adult		50<br>47	0.5%<br>0.5%	0.760	0.004

© 2024 Wang L et al. *JAMA Network Open.*

1<br>2	Z68.29	Body mass index [BMI] 29.0-29.9, adult	51<br>42	0.5%<br>0.4%	0.350	0.013
1<br>2	F10	Alcohol related disorders	65<br>44	0.6%<br>0.4%	0.044	0.028
1<br>2	Z80	Family history of primary malignant neoplasm	512<br>429	4.9%<br>4.1%	0.006	0.038
1<br>2	Z15.0	Genetic susceptibility to malignant neoplasm	10<br>10	0.1%<br>0.1%	1	<0.001
1<br>2	Z12	Encounter for screening for malignant neoplasms	2,695<br>2,467	25.9%<br>23.7%	<0.00<br>1	0.051
1<br>2	Z85	Personal history of malignant neoplasm	365<br>325	3.5%<br>3.1%	0.121	0.021
1<br>2	Z98.84	Bariatric surgery status	200<br>202	1.9%<br>1.9%	0.920	0.001
1<br>2	E66.0	Obesity due to excess calories	1,930<br>1,928	18.5%<br>18.5%	0.972	<0.001
1<br>2	E66.2	Morbid (severe) obesity with alveolar hypoventilation	81<br>79	0.8%<br>0.8%	0.874	0.002
1<br>2	E66.3	Overweight	165<br>159	1.6%<br>1.5%	0.737	0.005
1<br>2	E66.8	Other obesity	58<br>60	0.6%<br>0.6%	0.854	0.003
1<br>2	E66.9	Obesity, unspecified	2,678<br>2,701	25.7%<br>25.9%	0.716	0.005
1<br>2	O92.7	Other and unspecified disorders of lactation	0<br>0	0%<br>0%	--	--
1<br>2	Z79.89<br>0	Hormone replacement therapy	59<br>56	0.6%<br>0.5%	0.779	0.004
1<br>2	Z92.23	Personal history of estrogen therapy	10<br>10	0.1%<br>0.1%	1	<0.001
1<br>2	Z98.82	Breast implant status	10<br>10	0.1%<br>0.1%	1	<0.001
1<br>2	Z80.3	Family history of malignant neoplasm of breast	246<br>206	2.4%<br>2.0%	0.057	0.026

© 2024 Wang L et al. *JAMA Network Open.*

1<br>2	R92	Abnormal and inconclusive findings on diagnostic imaging of breast		653<br>530	6.3%<br>5.1%	<0.001	0.051
1<br>2	D24	Benign neoplasm of breast		57<br>48	0.5%<br>0.5%	0.379	0.012
1<br>2	N60	Benign mammary dysplasia		225<br>171	2.2%<br>1.6%	0.006	0.038
1<br>2	N62	Hypertrophy of breast		29<br>26	0.3%<br>0.2%	0.685	0.006
1<br>2	N63	Unspecified lump in breast		344<br>260	3.3%<br>2.5%	0.001	0.048
1<br>2	N64	Other disorders of breast		414<br>350	4.0%<br>3.4%	0.018	0.033
1<br>2	N61	Inflammatory disorders of breast		41<br>31	0.4%<br>0.3%	0.238	0.016
1<br>2	D05	Carcinoma in situ of breast		13<br>10	0.1%<br>0.1%	0.531	0.009
**Procedure**							
Group			Mean ± SD	Patients	% of Group	P-Value	SMD
1<br>2	C1789	Prosthesis, breast (implantable)		10<br>10	0.1%<br>0.1%	1	<0.001
1<br>2	58300	Insertion of intrauterine device (IUD)		10<br>10	0.1%<br>0.1%	1	<0.001
1<br>2	1001	Radiation		28<br>25	0.3%<br>0.2%	0.680	0.006
**Medication**							
Group			Mean ± SD	Patients	% of Group	P-Value	SMD
1<br>2	A10A	INSULINS AND ANALOGUES		4,506<br>4,458	43.2%<br>42.8%	0.502	0.009
1<br>2	A10BB	Sulfonylureas		1,814<br>1,830	17.4%<br>17.6%	0.770	0.004
1<br>2	A10BF	Alpha glucosidase inhibitors		35<br>32	0.3%<br>0.3%	0.714	0.005
1<br>2	A10BG	Thiazolidinediones		624<br>662	6.0%<br>6.4%	0.274	0.015

© 2024 Wang L et al. *JAMA Network Open.*

1<br>2	A10B<br>H	Dipeptidyl peptidase 4 (DPP-4) inhibitors	1,220<br>1,222	11.7%<br>11.7%	0.966	0.001
1<br>2	A10B<br>K	Sodium-glucose co-transporter 2 (SGLT2) inhibitors	496<br>428	4.8%<br>4.1%	0.022	0.032
1<br>2	A10B<br>X	Other blood glucose lowering drugs, excl. insulins	222<br>223	2.1%<br>2.1%	0.962	0.001
1<br>2	HS200	CONTRACEPTIVE S,SYSTEMIC	187<br>169	1.8%<br>1.6%	0.336	0.013

© 2024 Wang L et al. *JAMA Network Open.*

eTable 16. Characteristics of the GLP-1RA/no metformin group and metformin/no GLP-1RA group before and after matched for covariates related to colorectal cancer for the study populations of patients with T2D and no history of any OAC

Group 1 (N = 32,365) and group 2 (N = 856,160) characteristics before propensity score matching							
**Demographics**							
Group			Mean ± SD	Patients	% of Group	P-Value	SMD
1<br>2	AI	Age at Index	59.0 +/- 12.4<br>60.1 +/- 14.0	32,365<br>856,160	100%<br>100%	<0.001	0.080
1<br>2	2106-3	White		21,231<br>515,868	65.6%<br>60.3%	<0.001	0.111
1<br>2	1002-5	American Indian or Alaska Native		98<br>3,080	0.3%<br>0.4%	0.092	0.010
1<br>2	UNK	Unknown Race		4,272<br>119,036	13.2%<br>13.9%	<0.001	0.021
1<br>2	F	Female		17,278<br>399,923	53.4%<br>46.7%	<0.001	0.134
1<br>2	2076-8	Native Hawaiian or Other Pacific Islander		172<br>5,884	0.5%<br>0.7%	0.001	0.020
1<br>2	UN	Unknown Gender		1,670<br>19,592	5.2%<br>2.3%	<0.001	0.152
1<br>2	2186-5	Not Hispanic or Latino		22,299<br>528,686	68.9%<br>61.8%	<0.001	0.151
1<br>2	2135-2	Hispanic or Latino		2,660<br>91,303	8.2%<br>10.7%	<0.001	0.084
1<br>2	2054-5	Black or African American		5,107<br>141,606	15.8%<br>16.5%	<0.001	0.021
1<br>2	M	Male		13,417<br>436,645	41.5%<br>51.0%	<0.001	0.192
1<br>2	2028-9	Asian		681<br>35,540	2.1%<br>4.2%	<0.001	0.118
**Diagnosis**							
Group			Mean ± SD	Patients	% of Group	P-Value	SMD
1<br>2	Z55-Z65	Persons with potential health hazards related to socioeconomic and		422<br>10,310	1.3%<br>1.2%	0.107	0.009

		psychosocial circumstances				
1<br>2	E66	Overweight and obesity	11,418<br>149,974	35.3%<br>17.5%	<0.00<br>1	0.411
1<br>2	Z68.3	Body mass index [BMI] 30-39, adult	3,285<br>32,977	10.1%<br>3.9%	<0.00<br>1	0.249
1<br>2	Z68.4	Body mass index [BMI] 40 or greater, adult	3,012<br>23,535	9.3%<br>2.7%	<0.00<br>1	0.278
1<br>2	Z68.25	Body mass index [BMI] 25.0-25.9, adult	119<br>2,293	0.4%<br>0.3%	0.001	0.018
1<br>2	Z68.26	Body mass index [BMI] 26.0-26.9, adult	150<br>2,433	0.5%<br>0.3%	<0.00<br>1	0.029
1<br>2	Z68.27	Body mass index [BMI] 27.0-27.9, adult	209<br>2,942	0.6%<br>0.3%	<0.00<br>1	0.043
1<br>2	Z68.28	Body mass index [BMI] 28.0-28.9, adult	264<br>3,317	0.8%<br>0.4%	<0.00<br>1	0.055
1<br>2	Z68.29	Body mass index [BMI] 29.0-29.9, adult	299<br>3,753	0.9%<br>0.4%	<0.00<br>1	0.059
1<br>2	F10	Alcohol related disorders	460<br>21,150	1.4%<br>2.5%	<0.00<br>1	0.076
1<br>2	Z80	Family history of primary malignant neoplasm	1,222<br>16,133	3.8%<br>1.9%	<0.00<br>1	0.114
1<br>2	Z15.0	Genetic susceptibility to malignant neoplasm	18<br>140	0.1%<br>0.0%	<0.00<br>1	0.021
1<br>2	Z12	Encounter for screening for malignant neoplasms	6,223<br>114,996	19.2%<br>13.4%	<0.00<br>1	0.157
1<br>2	Z85	Personal history of malignant neoplasm	1,111<br>23,231	3.4%<br>2.7%	<0.00<br>1	0.042
1<br>2	Z98.84	Bariatric surgery status	534<br>2,989	1.6%<br>0.3%	<0.00<br>1	0.131
1<br>2	F17	Nicotine dependence	2,399<br>77,335	7.4%<br>9.0%	<0.00<br>1	0.059

© 2024 Wang L et al. *JAMA Network Open.*

1<br>2	E66.0	Obesity due to excess calories		5,910<br>56,178	18.3%<br>6.6%	<0.001	0.361
1<br>2	E66.2	Morbid (severe) obesity with alveolar hypoventilation		230<br>2,051	0.7%<br>0.2%	<0.001	0.069
1<br>2	E66.3	Overweight		535<br>7,794	1.7%<br>0.9%	<0.001	0.066
1<br>2	E66.8	Other obesity		193<br>1,084	0.6%<br>0.1%	<0.001	0.078
1<br>2	E66.9	Obesity, unspecified		8,150<br>113,380	25.2%<br>13.2%	<0.001	0.307
1<br>2	K50	Crohn's disease [regional enteritis]		149<br>2,039	0.5%<br>0.2%	<0.001	0.038
1<br>2	K51	Ulcerative colitis		126<br>2,339	0.4%<br>0.3%	<0.001	0.020
1<br>2	K63.5	Polyp of colon		1,321<br>31,663	4.1%<br>3.7%	<0.001	0.020
1<br>2	Z72.0	Tobacco use		588<br>11,927	1.8%<br>1.4%	<0.001	0.034
1<br>2	Z83.71	Family history of colonic polyps		48<br>606	0.1%<br>0.1%	<0.001	0.023
1<br>2	E84	Cystic fibrosis		10<br>362	0.0%<br>0.0%	0.326	0.006
1<br>2	Q85.8	Other phakomatoses, not elsewhere classified		10<br>117	0.0%<br>0.0%	0.011	0.012
1<br>2	Z80.0	Family history of malignant neoplasm of digestive organs		413<br>6,255	1.3%<br>0.7%	<0.001	0.055
1<br>2	D12	Benign neoplasm of colon, rectum, anus and anal canal		1,692<br>36,157	5.2%<br>4.2%	<0.001	0.047
**Procedure**							
Group			Mean ± SD	Patients	% of Group	P-Value	SMD
1<br>2	1022231	Colonoscopy, flexible		1,885<br>38,210	5.8%<br>4.5%	<0.001	0.062
**Medication**							
Group			Mean ± SD	Patients	% of Group	P-Value	SMD

© 2024 Wang L et al. *JAMA Network Open.*

1<br>2	A10A	INSULINS AND ANALOGUES		14,131<br>159,901	43.7%<br>18.7%	<0.001	0.560
1<br>2	A10BB	Sulfonylureas		5,464<br>69,045	16.9%<br>8.1%	<0.001	0.269
1<br>2	A10BF	Alpha glucosidase inhibitors		124<br>876	0.4%<br>0.1%	<0.001	0.057
1<br>2	A10BG	Thiazolidinediones		1,960<br>20,400	6.1%<br>2.4%	<0.001	0.183
1<br>2	A10BH	Dipeptidyl peptidase 4 (DPP-4) inhibitors		3,547<br>17,113	11.0%<br>2.0%	<0.001	0.370
1<br>2	A10BK	Sodium-glucose co-transporter 2 (SGLT2) inhibitors		1,839<br>3,371	5.7%<br>0.4%	<0.001	0.312
1<br>2	A10BX	Other blood glucose lowering drugs, excl. insulins		672<br>3,839	2.1%<br>0.4%	<0.001	0.146

Group 1 (N = 32,275) and group 2 (N = 32,275) characteristics after propensity score matching

Demographics

Group			Mean ± SD	Patients	% of Group	P-Value	SMD
1<br>2	AI	Age at Index	59.0 +/- 12.4<br>58.9 +/- 13.2	32,275<br>32,275	100%<br>100%	0.287	0.008
1<br>2	2106-3	White		21,169<br>21,665	65.6%<br>67.1%	<0.001	0.033
1<br>2	1002-5	American Indian or Alaska Native		98<br>100	0.3%<br>0.3%	0.887	0.001
1<br>2	UNK	Unknown Race		4,258<br>4,137	13.2%<br>12.8%	0.157	0.011
1<br>2	F	Female		17,215<br>17,440	53.3%<br>54.0%	0.076	0.014
1<br>2	2076-8	Native Hawaiian or Other Pacific Islander		172<br>147	0.5%<br>0.5%	0.161	0.011
1<br>2	UN	Unknown Gender		1,658<br>1,745	5.1%<br>5.4%	0.125	0.012
1<br>2	2186-5	Not Hispanic or Latino		22,237<br>22,623	68.9%<br>70.1%	0.001	0.026
1<br>2	2135-2	Hispanic or Latino		2,652<br>2,443	8.2%<br>7.6%	0.002	0.024

© 2024 Wang L et al. *JAMA Network Open.*

1<br>2	2054-5	Black or African American		5,093<br>4,870	15.8%<br>15.1%	0.015	0.019
1<br>2	M	Male		13,402<br>13,090	41.5%<br>40.6%	0.013	0.020
1<br>2	2028-9	Asian		681<br>613	2.1%<br>1.9%	0.056	0.015

Diagnosis

Group			Mean ± SD	Patients	% of Group	P-Value	SMD
1<br>2	Z55-Z65	Persons with potential health hazards related to socioeconomic and psychosocial circumstances		419<br>341	1.3%<br>1.1%	0.004	0.022
1<br>2	E66	Overweight and obesity		11,353<br>11,321	35.2%<br>35.1%	0.792	0.002
1<br>2	Z68.3	Body mass index [BMI] 30-39, adult		3,252<br>3,157	10.1%<br>9.8%	0.211	0.010
1<br>2	Z68.4	Body mass index [BMI] 40 or greater, adult		2,976<br>2,889	9.2%<br>9.0%	0.233	0.009
1<br>2	Z68.25	Body mass index [BMI] 25.0-25.9, adult		119<br>95	0.4%<br>0.3%	0.100	0.013
1<br>2	Z68.26	Body mass index [BMI] 26.0-26.9, adult		150<br>126	0.5%<br>0.4%	0.148	0.011
1<br>2	Z68.27	Body mass index [BMI] 27.0-27.9, adult		209<br>203	0.6%<br>0.6%	0.767	0.002
1<br>2	Z68.28	Body mass index [BMI] 28.0-28.9, adult		264<br>265	0.8%<br>0.8%	0.965	<0.001
1<br>2	Z68.29	Body mass index [BMI] 29.0-29.9, adult		299<br>275	0.9%<br>0.9%	0.314	0.008
1<br>2	F10	Alcohol related disorders		458<br>339	1.4%<br>1.1%	<0.001	0.033
1<br>2	Z80	Family history of primary malignant neoplasm		1,208<br>1,125	3.7%<br>3.5%	0.080	0.014

© 2024 Wang L et al. *JAMA Network Open.*

1<br>2	Z15.0	Genetic susceptibility to malignant neoplasm	18<br>10	0.1%<br>0.0%	0.130	0.012
1<br>2	Z12	Encounter for screening for malignant neoplasms	6,177<br>5,545	19.1%<br>17.2%	<0.001	0.051
1<br>2	Z85	Personal history of malignant neoplasm	1,108<br>1,059	3.4%<br>3.3%	0.284	0.008
1<br>2	Z98.84	Bariatric surgery status	519<br>503	1.6%<br>1.6%	0.614	0.004
1<br>2	F17	Nicotine dependence	2,390<br>2,101	7.4%<br>6.5%	<0.001	0.035
1<br>2	E66.0	Obesity due to excess calories	5,854<br>5,858	18.1%<br>18.2%	0.967	<0.001
1<br>2	E66.2	Morbid (severe) obesity with alveolar hypoventilation	227<br>224	0.7%<br>0.7%	0.887	0.001
1<br>2	E66.3	Overweight	530<br>434	1.6%<br>1.3%	0.002	0.025
1<br>2	E66.8	Other obesity	191<br>172	0.6%<br>0.5%	0.317	0.008
1<br>2	E66.9	Obesity, unspecified	8,109<br>7,992	25.1%<br>24.8%	0.287	0.008
1<br>2	K50	Crohn's disease [regional enteritis]	147<br>131	0.5%<br>0.4%	0.336	0.008
1<br>2	K51	Ulcerative colitis	126<br>103	0.4%<br>0.3%	0.128	0.012
1<br>2	K63.5	Polyp of colon	1,307<br>1,060	4.0%<br>3.3%	<0.001	0.041
1<br>2	Z72.0	Tobacco use	583<br>511	1.8%<br>1.6%	0.028	0.017
1<br>2	Z83.71	Family history of colonic polyps	46<br>52	0.1%<br>0.2%	0.544	0.005
1<br>2	E84	Cystic fibrosis	10<br>15	0.0%<br>0.0%	0.317	0.008
1<br>2	Q85.8	Other phakomatoses, not elsewhere classified	10<br>10	0.0%<br>0.0%	1	<0.001
1<br>2	Z80.0	Family history of malignant neoplasm of digestive organs	408<br>355	1.3%<br>1.1%	0.054	0.015

© 2024 Wang L et al. *JAMA Network Open.*

1<br>2	D12	Benign neoplasm of colon, rectum, anus and anal canal		1,675<br>1,345	5.2%<br>4.2%	<0.00<br>1	0.048

Procedure

Group			Mean ± SD	Patients	% of Group	P-Value	SMD
1<br>2	10222<br>31	Colonoscopy, flexible		1,869<br>1,530	5.8%<br>4.7%	<0.00<br>1	0.047

Medication

Group			Mean ± SD	Patients	% of Group	P-Value	SMD
1<br>2	A10A	INSULINS AND ANALOGUES		14,048<br>13,850	43.5%<br>42.9%	0.116	0.012
1<br>2	A10B<br>B	Sulfonylureas		5,426<br>5,301	16.8%<br>16.4%	0.186	0.010
1<br>2	A10B<br>F	Alpha glucosidase inhibitors		124<br>124	0.4%<br>0.4%	1	<0.001
1<br>2	A10B<br>G	Thiazolidinediones		1,931<br>1,945	6.0%<br>6.0%	0.817	0.002
1<br>2	A10B<br>H	Dipeptidyl peptidase 4 (DPP-4) inhibitors		3,474<br>3,386	10.8%<br>10.5%	0.261	0.009
1<br>2	A10B<br>K	Sodium-glucose co-transporter 2 (SGLT2) inhibitors		1,756<br>1,623	5.4%<br>5.0%	0.019	0.019
1<br>2	A10B<br>X	Other blood glucose lowering drugs, excl. insulins		660<br>667	2.0%<br>2.1%	0.846	0.002

© 2024 Wang L et al. *JAMA Network Open.*

eTable 17. Characteristics of the GLP-1RA/no metformin group and metformin/no GLP-1RA group before and after matched for covariates related to endometrial cancer for the study populations of women with T2D and no history of any OAC

Group 1 (N = 17,278) and group 2 (N = 399,923) characteristics before propensity score matching

Demographics

Group			Mean ± SD	Patients	% of Group	P-Value	SMD
1<br>2	AI	Age at Index	57.7 +/- 12.8<br>60.0 +/- 14.7	17,278<br>399,923	100%<br>100%	<0.001	0.160
1<br>2	2106-3	White		11,336<br>231,828	65.6%<br>58.0%	<0.001	0.158
1<br>2	1002-5	American Indian or Alaska Native		59<br>1,552	0.3%<br>0.4%	0.334	0.008
1<br>2	UNK	Unknown Race		1,563<br>49,384	9.0%<br>12.3%	<0.001	0.107
1<br>2	2076-8	Native Hawaiian or Other Pacific Islander		80<br>2,869	0.5%<br>0.7%	<0.001	0.033
1<br>2	UN	Unknown Ethnicity		3,145<br>103,904	18.2%<br>26.0%	<0.001	0.188
1<br>2	2186-5	Not Hispanic or Latino		12,481<br>247,896	72.2%<br>62.0%	<0.001	0.219
1<br>2	2135-2	Hispanic or Latino		1,652<br>48,123	9.6%<br>12.0%	<0.001	0.080
1<br>2	2054-5	Black or African American		3,476<br>80,016	20.1%<br>20.0%	0.723	0.003
1<br>2	2028-9	Asian		311<br>17,483	1.8%<br>4.4%	<0.001	0.149

Diagnosis

Group			Mean ± SD	Patients	% of Group	P-Value	SMD
1<br>2	Z55-Z65	Persons with potential health hazards related to socioeconomic and psychosocial circumstances		270<br>5,204	1.6%<br>1.3%	0.003	0.022
1<br>2	E66	Overweight and obesity		6,461<br>78,874	37.4%<br>19.7%	<0.001	0.399

© 2024 Wang L et al. *JAMA Network Open.*

1<br>2	Z68.3	Body mass index [BMI] 30-39, adult	1,360<br>13,007	7.9%<br>3.3%	<0.00<br>1	0.203
1<br>2	Z68.4	Body mass index [BMI] 40 or greater, adult	1,671<br>12,991	9.7%<br>3.2%	<0.00<br>1	0.264
1<br>2	Z68.25	Body mass index [BMI] 25.0-25.9, adult	39<br>643	0.2%<br>0.2%	0.039	0.015
1<br>2	Z68.26	Body mass index [BMI] 26.0-26.9, adult	52<br>616	0.3%<br>0.2%	<0.00<br>1	0.031
1<br>2	Z68.27	Body mass index [BMI] 27.0-27.9, adult	59<br>760	0.3%<br>0.2%	<0.00<br>1	0.029
1<br>2	Z68.28	Body mass index [BMI] 28.0-28.9, adult	69<br>862	0.4%<br>0.2%	<0.00<br>1	0.033
1<br>2	Z68.29	Body mass index [BMI] 29.0-29.9, adult	76<br>1,029	0.4%<br>0.3%	<0.00<br>1	0.031
1<br>2	Z80	Family history of primary malignant neoplasm	821<br>9,798	4.8%<br>2.4%	<0.00<br>1	0.124
1<br>2	Z15.0	Genetic susceptibility to malignant neoplasm	15<br>91	0.1%<br>0.0%	<0.00<br>1	0.027
1<br>2	Z12	Encounter for screening for malignant neoplasms	4,113<br>71,089	23.8%<br>17.8%	<0.00<br>1	0.149
1<br>2	Z85	Personal history of malignant neoplasm	492<br>9,342	2.8%<br>2.3%	<0.00<br>1	0.032
1<br>2	Z98.84	Bariatric surgery status	378<br>2,056	2.2%<br>0.5%	<0.00<br>1	0.145
1<br>2	E66.0	Obesity due to excess calories	3,456<br>31,701	20.0%<br>7.9%	<0.00<br>1	0.354
1<br>2	E66.2	Morbid (severe) obesity with alveolar hypoventilation	132<br>1,060	0.8%<br>0.3%	<0.00<br>1	0.070
1<br>2	E66.3	Overweight	280<br>3,619	1.6%<br>0.9%	<0.00<br>1	0.064
1<br>2	E66.8	Other obesity	118<br>574	0.7%<br>0.1%	<0.00<br>1	0.084

© 2024 Wang L et al. *JAMA Network Open.*

1<br>2	E66.9	Obesity, unspecified		4,568<br>59,095	26.4%<br>14.8%	<0.001	0.291
1<br>2	Z72.3	Lack of physical exercise		10<br>138	0.1%<br>0.0%	0.110	0.011
1<br>2	Z72.4	Inappropriate diet and eating habits		30<br>73	0.2%<br>0.0%	<0.001	0.050
1<br>2	Z79.890	Hormone replacement therapy		85<br>1,319	0.5%<br>0.3%	<0.001	0.025
1<br>2	Z92.23	Personal history of estrogen therapy		10<br>115	0.1%<br>0.0%	0.030	0.014
1<br>2	E28.2	Polycystic ovarian syndrome		264<br>3,504	1.5%<br>0.9%	<0.001	0.060
1<br>2	Z80.41	Family history of malignant neoplasm of ovary		50<br>568	0.3%<br>0.1%	<0.001	0.032
1<br>2	Z80.49	Family history of malignant neoplasm of other genital organs		27<br>261	0.2%<br>0.1%	<0.001	0.027
1<br>2	N85.0	Endometrial hyperplasia		94<br>1,487	0.5%<br>0.4%	<0.001	0.026
1<br>2	D25	Leiomyoma of uterus		470<br>9,804	2.7%<br>2.5%	0.026	0.017
1<br>2	D26	Other benign neoplasms of uterus		20<br>505	0.1%<br>0.1%	0.703	0.003
1<br>2	D27	Benign neoplasm of ovary		39<br>879	0.2%<br>0.2%	0.871	0.001
1<br>2	Z80.3	Family history of malignant neoplasm of breast		390<br>4,796	2.3%<br>1.2%	<0.001	0.081
**Procedure**							
Group			Mean ± SD	Patients	% of Group	P-Value	SMD
1<br>2	58300	Insertion of intrauterine device (IUD)		122<br>1,090	0.7%<br>0.3%	<0.001	0.062
1<br>2	1001	Radiation		43<br>1,352	0.2%<br>0.3%	0.047	0.016
**Medication**							
Group			Mean ± SD	Patients	% of Group	P-Value	SMD

© 2024 Wang L et al. *JAMA Network Open.*

1<br>2	A10A	INSULINS AND ANALOGUES		7,387<br>72,077	42.8%<br>18.0%	<0.001	0.558
1<br>2	A10BB	Sulfonylureas		2,808<br>31,308	16.3%<br>7.8%	<0.001	0.261
1<br>2	A10BF	Alpha glucosidase inhibitors		60<br>436	0.3%<br>0.1%	<0.001	0.050
1<br>2	A10BG	Thiazolidinediones		974<br>9,195	5.6%<br>2.3%	<0.001	0.172
1<br>2	A10BH	Dipeptidyl peptidase 4 (DPP-4) inhibitors		1,906<br>7,852	11.0%<br>2.0%	<0.001	0.374
1<br>2	A10BK	Sodium-glucose co-transporter 2 (SGLT2) inhibitors		918<br>1,296	5.3%<br>0.3%	<0.001	0.305
1<br>2	A10BX	Other blood glucose lowering drugs, excl. insulins		349<br>1,821	2.0%<br>0.5%	<0.001	0.142
1<br>2	HS200	CONTRACEPTIVES,SYSTEMIC		718<br>10,566	4.2%<br>2.6%	<0.001	0.084
1<br>2	10324	tamoxifen		17<br>295	0.1%<br>0.1%	0.246	0.008

Group 1 (N = 17,168) and group 2 (N = 17,168) characteristics after propensity score matching

Demographics

Group			Mean ± SD	Patients	% of Group	P-Value	SMD
1<br>2	AI	Age at Index	57.8 +/- 12.8<br>57.5 +/- 13.8	17,168<br>17,168	100%<br>100%	0.118	0.017
1<br>2	2106-3	White		11,256<br>11,549	65.6%<br>67.3%	0.001	0.036
1<br>2	1002-5	American Indian or Alaska Native		59<br>58	0.3%<br>0.3%	0.926	0.001
1<br>2	UNK	Unknown Race		1,559<br>1,504	9.1%<br>8.8%	0.298	0.011
1<br>2	2076-8	Native Hawaiian or Other Pacific Islander		80<br>79	0.5%<br>0.5%	0.937	0.001
1<br>2	UN	Unknown Ethnicity		3,140<br>2,997	18.3%<br>17.5%	0.044	0.022
1<br>2	2186-5	Not Hispanic or Latino		12,387<br>12,659	72.2%<br>73.7%	0.001	0.036

© 2024 Wang L et al. *JAMA Network Open.*

Group			Mean ± SD	Patients	% of Group	P-Value	SMD
1<br>2	2135-2	Hispanic or Latino		1,641<br>1,512	9.6%<br>8.8%	0.016	0.026
1<br>2	2054-5	Black or African American		3,454<br>3,321	20.1%<br>19.3%	0.071	0.019
1<br>2	2028-9	Asian		310<br>257	1.8%<br>1.5%	0.025	0.024

Diagnosis

Group			Mean ± SD	Patients	% of Group	P-Value	SMD
1<br>2	Z55-Z65	Persons with potential health hazards related to socioeconomic and psychosocial circumstances		268<br>236	1.6%<br>1.4%	0.151	0.015
1<br>2	E66	Overweight and obesity		6,388<br>6,343	37.2%<br>36.9%	0.615	0.005
1<br>2	Z68.3	Body mass index [BMI] 30-39, adult		1,331<br>1,299	7.8%<br>7.6%	0.516	0.007
1<br>2	Z68.4	Body mass index [BMI] 40 or greater, adult		1,636<br>1,580	9.5%<br>9.2%	0.300	0.011
1<br>2	Z68.25	Body mass index [BMI] 25.0-25.9, adult		38<br>24	0.2%<br>0.1%	0.075	0.019
1<br>2	Z68.26	Body mass index [BMI] 26.0-26.9, adult		51<br>42	0.3%<br>0.2%	0.350	0.010
1<br>2	Z68.27	Body mass index [BMI] 27.0-27.9, adult		58<br>56	0.3%<br>0.3%	0.851	0.002
1<br>2	Z68.28	Body mass index [BMI] 28.0-28.9, adult		66<br>56	0.4%<br>0.3%	0.364	0.010
1<br>2	Z68.29	Body mass index [BMI] 29.0-29.9, adult		75<br>58	0.4%<br>0.3%	0.140	0.016
1<br>2	Z80	Family history of primary malignant neoplasm		798<br>716	4.6%<br>4.2%	0.031	0.023

© 2024 Wang L et al. *JAMA Network Open.*

1<br>2	Z15.0	Genetic susceptibility to malignant neoplasm	15<br>11	0.1%<br>0.1%	0.433	0.008
1<br>2	Z12	Encounter for screening for malignant neoplasms	4,054<br>3,674	23.6%<br>21.4%	<0.001	0.053
1<br>2	Z85	Personal history of malignant neoplasm	488<br>435	2.8%<br>2.5%	0.077	0.019
1<br>2	Z98.84	Bariatric surgery status	365<br>352	2.1%<br>2.1%	0.624	0.005
1<br>2	E66.0	Obesity due to excess calories	3,402<br>3,400	19.8%<br>19.8%	0.978	<0.001
1<br>2	E66.2	Morbid (severe) obesity with alveolar hypoventilation	131<br>111	0.8%<br>0.6%	0.197	0.014
1<br>2	E66.3	Overweight	274<br>235	1.6%<br>1.4%	0.082	0.019
1<br>2	E66.8	Other obesity	112<br>113	0.7%<br>0.7%	0.947	0.001
1<br>2	E66.9	Obesity, unspecified	4,514<br>4,433	26.3%<br>25.8%	0.319	0.011
1<br>2	Z72.3	Lack of physical exercise	10<br>10	0.1%<br>0.1%	1	<0.001
1<br>2	Z72.4	Inappropriate diet and eating habits	25<br>30	0.1%<br>0.2%	0.500	0.007
1<br>2	Z79.890	Hormone replacement therapy	83<br>67	0.5%<br>0.4%	0.190	0.014
1<br>2	Z92.23	Personal history of estrogen therapy	10<br>10	0.1%<br>0.1%	1	<0.001
1<br>2	E28.2	Polycystic ovarian syndrome	260<br>243	1.5%<br>1.4%	0.445	0.008
1<br>2	Z80.41	Family history of malignant neoplasm of ovary	47<br>45	0.3%<br>0.3%	0.835	0.002
1<br>2	Z80.49	Family history of malignant neoplasm of other genital organs	27<br>21	0.2%<br>0.1%	0.386	0.009
1<br>2	N85.0	Endometrial hyperplasia	93<br>85	0.5%<br>0.5%	0.548	0.006
1<br>2	D25	Leiomyoma of uterus	465<br>400	2.7%<br>2.3%	0.025	0.024

© 2024 Wang L et al. *JAMA Network Open.*

1<br>2	D26	Other benign neoplasms of uterus		20<br>16	0.1%<br>0.1%	0.505	0.007
1<br>2	D27	Benign neoplasm of ovary		38<br>26	0.2%<br>0.2%	0.133	0.016
1<br>2	Z80.3	Family history of malignant neoplasm of breast		380<br>325	2.2%<br>1.9%	0.036	0.023
**Procedure**							
Group			Mean ± SD	Patients	% of Group	P-Value	SMD
1<br>2	58300	Insertion of intrauterine device (IUD)		118<br>114	0.7%<br>0.7%	0.792	0.003
1<br>2	1001	Radiation		43<br>38	0.3%<br>0.2%	0.578	0.006
**Medication**							
Group			Mean ± SD	Patients	% of Group	P-Value	SMD
1<br>2	A10A	INSULINS AND ANALOGUES		7,290<br>7,253	42.5%<br>42.2%	0.686	0.004
1<br>2	A10B B	Sulfonylureas		2,753<br>2,748	16.0%<br>16.0%	0.941	0.001
1<br>2	A10B F	Alpha glucosidase inhibitors		59<br>53	0.3%<br>0.3%	0.570	0.006
1<br>2	A10B G	Thiazolidinediones		949<br>909	5.5%<br>5.3%	0.340	0.010
1<br>2	A10B H	Dipeptidyl peptidase 4 (DPP-4) inhibitors		1,827<br>1,853	10.6%<br>10.8%	0.650	0.005
1<br>2	A10B K	Sodium-glucose co-transporter 2 (SGLT2) inhibitors		817<br>728	4.8%<br>4.2%	0.021	0.025
1<br>2	A10B X	Other blood glucose lowering drugs, excl. insulins		328<br>340	1.9%<br>2.0%	0.639	0.005
1<br>2	HS200	CONTRACEPTIVE S,SYSTEMIC		704<br>623	4.1%<br>3.6%	0.023	0.024
1<br>2	10324	tamoxifen		17<br>16	0.1%<br>0.1%	0.862	0.002

© 2024 Wang L et al. *JAMA Network Open.*

eTable 18. Characteristics of the GLP-1RA/no metformin group and metformin/no GLP-1RA group before and after matched for covariates related to gallbladder cancer for the study populations of patients with T2D and no history of any OAC

Group 1 (N = 32,365) and group 2 (N = 856,160) characteristics before propensity score matching							
**Demographics**							
Group			Mean ± SD	Patients	% of Group	P-Value	SMD
1<br>2	AI	Age at Index	59.0 +/- 12.4<br>60.1 +/- 14.0	32,365<br>856,160	100%<br>100%	<0.001	0.080
1<br>2	2106-3	White		21,231<br>515,868	65.6%<br>60.3%	<0.001	0.111
1<br>2	UNK	Unknown Race		4,272<br>119,036	13.2%<br>13.9%	<0.001	0.021
1<br>2	F	Female		17,278<br>399,923	53.4%<br>46.7%	<0.001	0.134
1<br>2	UN	Unknown Gender		1,670<br>19,592	5.2%<br>2.3%	<0.001	0.152
1<br>2	2186-5	Not Hispanic or Latino		22,299<br>528,686	68.9%<br>61.8%	<0.001	0.151
1<br>2	2135-2	Hispanic or Latino		2,660<br>91,303	8.2%<br>10.7%	<0.001	0.084
1<br>2	2054-5	Black or African American		5,107<br>141,606	15.8%<br>16.5%	<0.001	0.021
1<br>2	M	Male		13,417<br>436,645	41.5%<br>51.0%	<0.001	0.192
1<br>2	2028-9	Asian		681<br>35,540	2.1%<br>4.2%	<0.001	0.118
**Diagnosis**							
Group			Mean ± SD	Patients	% of Group	P-Value	SMD
1<br>2	Z55-Z65	Persons with potential health hazards related to socioeconomic and psychosocial circumstances		422<br>10,310	1.3%<br>1.2%	0.107	0.009
1<br>2	E66	Overweight and obesity		11,418<br>149,974	35.3%<br>17.5%	<0.001	0.411
1<br>2	Z68.3	Body mass index [BMI] 30-39, adult		3,285<br>32,977	10.1%<br>3.9%	<0.001	0.249

© 2024 Wang L et al. *JAMA Network Open.*

1<br>2	Z68.4	Body mass index [BMI] 40 or greater, adult	3,012<br>23,535	9.3%<br>2.7%	<0.00<br>1	0.278
1<br>2	Z68.25	Body mass index [BMI] 25.0-25.9, adult	119<br>2,293	0.4%<br>0.3%	0.001	0.018
1<br>2	Z68.26	Body mass index [BMI] 26.0-26.9, adult	150<br>2,433	0.5%<br>0.3%	<0.00<br>1	0.029
1<br>2	Z68.27	Body mass index [BMI] 27.0-27.9, adult	209<br>2,942	0.6%<br>0.3%	<0.00<br>1	0.043
1<br>2	Z68.28	Body mass index [BMI] 28.0-28.9, adult	264<br>3,317	0.8%<br>0.4%	<0.00<br>1	0.055
1<br>2	Z68.29	Body mass index [BMI] 29.0-29.9, adult	299<br>3,753	0.9%<br>0.4%	<0.00<br>1	0.059
1<br>2	F10	Alcohol related disorders	460<br>21,150	1.4%<br>2.5%	<0.00<br>1	0.076
1<br>2	Z80	Family history of primary malignant neoplasm	1,222<br>16,133	3.8%<br>1.9%	<0.00<br>1	0.114
1<br>2	Z15.0	Genetic susceptibility to malignant neoplasm	18<br>140	0.1%<br>0.0%	<0.00<br>1	0.021
1<br>2	Z12	Encounter for screening for malignant neoplasms	6,223<br>114,996	19.2%<br>13.4%	<0.00<br>1	0.157
1<br>2	Z85	Personal history of malignant neoplasm	1,111<br>23,231	3.4%<br>2.7%	<0.00<br>1	0.042
1<br>2	Z98.84	Bariatric surgery status	534<br>2,989	1.6%<br>0.3%	<0.00<br>1	0.131
1<br>2	F17	Nicotine dependence	2,399<br>77,335	7.4%<br>9.0%	<0.00<br>1	0.059
1<br>2	E66.0	Obesity due to excess calories	5,910<br>56,178	18.3%<br>6.6%	<0.00<br>1	0.361
1<br>2	E66.2	Morbid (severe) obesity with alveolar hypoventilation	230<br>2,051	0.7%<br>0.2%	<0.00<br>1	0.069
1<br>2	E66.3	Overweight	535<br>7,794	1.7%<br>0.9%	<0.00<br>1	0.066

© 2024 Wang L et al. *JAMA Network Open.*

1<br>2	E66.8	Other obesity		193<br>1,084	0.6%<br>0.1%	<0.00<br>1	0.078
1<br>2	E66.9	Obesity, unspecified		8,150<br>113,380	25.2%<br>13.2%	<0.00<br>1	0.307
1<br>2	Z72.0	Tobacco use		588<br>11,927	1.8%<br>1.4%	<0.00<br>1	0.034
1<br>2	K80	Cholelithiasis		871<br>17,909	2.7%<br>2.1%	<0.00<br>1	0.039
1<br>2	K82.8	Other specified diseases of gallbladder		129<br>2,130	0.4%<br>0.2%	<0.00<br>1	0.026
1<br>2	Q44.4	Choledochal cyst		10<br>160	0.0%<br>0.0%	0.119	0.008
1<br>2	Q44.5	Other congenital malformations of bile ducts		10<br>232	0.0%<br>0.0%	0.684	0.002
1<br>2	K82.4	Cholesterolosis of gallbladder		55<br>998	0.2%<br>0.1%	0.006	0.014
1<br>2	K83.0<br>1	Primary sclerosing cholangitis		10<br>36	0.0%<br>0.0%	<0.00<br>1	0.020
1<br>2	A01.0	Typhoid fever		10<br>17	0.0%<br>0.0%	<0.00<br>1	0.023
1<br>2	Z85.09	Personal history of malignant neoplasm of other digestive organs		10<br>133	0.0%<br>0.0%	0.032	0.010
1<br>2	D13.5	Benign neoplasm of extrahepatic bile ducts		12<br>311	0.0%<br>0.0%	0.944	<0.001
**Medication**							
Group			Mean ± SD	Patients	% of Group	P-Value	SMD
1<br>2	A10A	INSULINS AND ANALOGUES		14,131<br>159,901	43.7%<br>18.7%	<0.00<br>1	0.560
1<br>2	A10B<br>B	Sulfonylureas		5,464<br>69,045	16.9%<br>8.1%	<0.00<br>1	0.269
1<br>2	A10B<br>F	Alpha glucosidase inhibitors		124<br>876	0.4%<br>0.1%	<0.00<br>1	0.057
1<br>2	A10B<br>G	Thiazolidinediones		1,960<br>20,400	6.1%<br>2.4%	<0.00<br>1	0.183
1<br>2	A10B<br>H	Dipeptidyl peptidase 4 (DPP-4) inhibitors		3,547<br>17,113	11.0%<br>2.0%	<0.00<br>1	0.370

1<br>2	A10BK	Sodium-glucose co-transporter 2 (SGLT2) inhibitors		1,839<br>3,371	5.7%<br>0.4%	<0.001	0.312
1<br>2	A10BX	Other blood glucose lowering drugs, excl. insulins		672<br>3,839	2.1%<br>0.4%	<0.001	0.146

Group 1 (N = 32,261) and group 2 (N = 32,261) characteristics after propensity score matching

Demographics

Group			Mean ± SD	Patients	% of Group	P-Value	SMD
1<br>2	AI	Age at Index	59.0 +/- 12.4<br>58.9 +/- 13.3	32,261<br>32,261	100%<br>100%	0.493	0.005
1<br>2	2106-3	White		21,159<br>21,672	65.6%<br>67.2%	<0.001	0.034
1<br>2	UNK	Unknown Race		4,253<br>4,152	13.2%<br>12.9%	0.237	0.009
1<br>2	F	Female		17,211<br>17,401	53.3%<br>53.9%	0.134	0.012
1<br>2	UN	Unknown Gender		1,654<br>1,725	5.1%<br>5.3%	0.210	0.010
1<br>2	2186-5	Not Hispanic or Latino		22,225<br>22,522	68.9%<br>69.8%	0.011	0.020
1<br>2	2135-2	Hispanic or Latino		2,653<br>2,505	8.2%<br>7.8%	0.032	0.017
1<br>2	2054-5	Black or African American		5,095<br>4,817	15.8%<br>14.9%	0.002	0.024
1<br>2	M	Male		13,396<br>13,135	41.5%<br>40.7%	0.037	0.016
1<br>2	2028-9	Asian		681<br>591	2.1%<br>1.8%	0.011	0.020

Diagnosis

Group			Mean ± SD	Patients	% of Group	P-Value	SMD
1<br>2	Z55-Z65	Persons with potential health hazards related to socioeconomic and psychosocial circumstances		420<br>356	1.3%<br>1.1%	0.021	0.018
1<br>2	E66	Overweight and obesity		11,341<br>11,362	35.2%<br>35.2%	0.863	0.001

© 2024 Wang L et al. *JAMA Network Open.*

1<br>2	Z68.3	Body mass index [BMI] 30-39, adult	3,255<br>3,223	10.1%<br>10.0%	0.675	0.003
1<br>2	Z68.4	Body mass index [BMI] 40 or greater, adult	2,967<br>2,840	9.2%<br>8.8%	0.081	0.014
1<br>2	Z68.25	Body mass index [BMI] 25.0-25.9, adult	119<br>112	0.4%<br>0.3%	0.645	0.004
1<br>2	Z68.26	Body mass index [BMI] 26.0-26.9, adult	149<br>117	0.5%<br>0.4%	0.049	0.015
1<br>2	Z68.27	Body mass index [BMI] 27.0-27.9, adult	209<br>174	0.6%<br>0.5%	0.073	0.014
1<br>2	Z68.28	Body mass index [BMI] 28.0-28.9, adult	263<br>243	0.8%<br>0.8%	0.372	0.007
1<br>2	Z68.29	Body mass index [BMI] 29.0-29.9, adult	298<br>314	0.9%<br>1.0%	0.516	0.005
1<br>2	F10	Alcohol related disorders	459<br>356	1.4%<br>1.1%	<0.001	0.029
1<br>2	Z80	Family history of primary malignant neoplasm	1,210<br>1,076	3.8%<br>3.3%	0.004	0.022
1<br>2	Z15.0	Genetic susceptibility to malignant neoplasm	18<br>19	0.1%<br>0.1%	0.869	0.001
1<br>2	Z12	Encounter for screening for malignant neoplasms	6,177<br>5,562	19.1%<br>17.2%	<0.001	0.049
1<br>2	Z85	Personal history of malignant neoplasm	1,106<br>1,050	3.4%<br>3.3%	0.220	0.010
1<br>2	Z98.84	Bariatric surgery status	517<br>571	1.6%<br>1.8%	0.099	0.013
1<br>2	F17	Nicotine dependence	2,392<br>2,115	7.4%<br>6.6%	<0.001	0.034
1<br>2	E66.0	Obesity due to excess calories	5,850<br>5,854	18.1%<br>18.1%	0.967	<0.001
1<br>2	E66.2	Morbid (severe) obesity with alveolar hypoventilation	228<br>213	0.7%<br>0.7%	0.474	0.006

© 2024 Wang L et al. *JAMA Network Open.*

1<br>2	E66.3	Overweight		531<br>473	1.6%<br>1.5%	0.065	0.015
1<br>2	E66.8	Other obesity		191<br>189	0.6%<br>0.6%	0.918	0.001
1<br>2	E66.9	Obesity, unspecified		8,099<br>8,093	25.1%<br>25.1%	0.957	<0.001
1<br>2	Z72.0	Tobacco use		585<br>477	1.8%<br>1.5%	0.001	0.026
1<br>2	K80	Cholelithiasis		866<br>758	2.7%<br>2.3%	0.007	0.021
1<br>2	K82.8	Other specified diseases of gallbladder		127<br>118	0.4%<br>0.4%	0.565	0.005
1<br>2	Q44.4	Choledochal cyst		10<br>10	0.0%<br>0.0%	1	<0.001
1<br>2	Q44.5	Other congenital malformations of bile ducts		10<br>10	0.0%<br>0.0%	1	<0.001
1<br>2	K82.4	Cholesterolosis of gallbladder		55<br>46	0.2%<br>0.1%	0.370	0.007
1<br>2	K83.01	Primary sclerosing cholangitis		10<br>10	0.0%<br>0.0%	1	<0.001
1<br>2	A01.0	Typhoid fever		10<br>10	0.0%<br>0.0%	1	<0.001
1<br>2	Z85.09	Personal history of malignant neoplasm of other digestive organs		10<br>10	0.0%<br>0.0%	1	<0.001
1<br>2	D13.5	Benign neoplasm of extrahepatic bile ducts		12<br>10	0.0%<br>0.0%	0.670	0.003

Medication

Group			Mean ± SD	Patients	% of Group	P-Value	SMD
1<br>2	A10A	INSULINS AND ANALOGUES		14,038<br>13,911	43.5%<br>43.1%	0.313	0.008
1<br>2	A10BB	Sulfonylureas		5,421<br>5,369	16.8%<br>16.6%	0.583	0.004
1<br>2	A10BF	Alpha glucosidase inhibitors		122<br>114	0.4%<br>0.4%	0.602	0.004
1<br>2	A10BG	Thiazolidinediones		1,932<br>1,896	6.0%<br>5.9%	0.549	0.005

© 2024 Wang L et al. *JAMA Network Open.*

1<br>2	A10B<br>H	Dipeptidyl peptidase 4 (DPP-4) inhibitors	3,467<br>3,412	10.7%<br>10.6%	0.483	0.006
1<br>2	A10B<br>K	Sodium-glucose co-transporter 2 (SGLT2) inhibitors	1,746<br>1,578	5.4%<br>4.9%	0.003	0.024
1<br>2	A10B<br>X	Other blood glucose lowering drugs, excl. insulins	657<br>650	2.0%<br>2.0%	0.845	0.002

© 2024 Wang L et al. *JAMA Network Open.*

eTable 19. Characteristics of the GLP-1RA/no metformin group and metformin/no GLP-1RA group before and after matched for covariates related to stomach cancer for the study populations of patients with T2D and no history of any OAC

Group 1 (N = 32,365) and group 2 (N = 856,160) characteristics before propensity score matching

Demographics

Group			Mean ± SD	Patients	% of Group	P-Value	SMD
1<br>2	AI	Age at Index	59.0 +/- 12.4<br>60.1 +/- 14.0	32,365<br>856,160	100%<br>100%	<0.001	0.080
1<br>2	2106-3	White		21,231<br>515,868	65.6%<br>60.3%	<0.001	0.111
1<br>2	1002-5	American Indian or Alaska Native		98<br>3,080	0.3%<br>0.4%	0.092	0.010
1<br>2	UNK	Unknown Race		4,272<br>119,036	13.2%<br>13.9%	<0.001	0.021
1<br>2	F	Female		17,278<br>399,923	53.4%<br>46.7%	<0.001	0.134
1<br>2	2076-8	Native Hawaiian or Other Pacific Islander		172<br>5,884	0.5%<br>0.7%	0.001	0.020
1<br>2	UN	Unknown Gender		1,670<br>19,592	5.2%<br>2.3%	<0.001	0.152
1<br>2	2186-5	Not Hispanic or Latino		22,299<br>528,686	68.9%<br>61.8%	<0.001	0.151
1<br>2	2135-2	Hispanic or Latino		2,660<br>91,303	8.2%<br>10.7%	<0.001	0.084
1<br>2	2054-5	Black or African American		5,107<br>141,606	15.8%<br>16.5%	<0.001	0.021
1<br>2	M	Male		13,417<br>436,645	41.5%<br>51.0%	<0.001	0.192
1<br>2	2028-9	Asian		681<br>35,540	2.1%<br>4.2%	<0.001	0.118

Diagnosis

Group			Mean ± SD	Patients	% of Group	P-Value	SMD
1<br>2	Z55-Z65	Persons with potential health hazards related to socioeconomic and		422<br>10,310	1.3%<br>1.2%	0.107	0.009

© 2024 Wang L et al. *JAMA Network Open.*

		psychosocial circumstances				
1<br>2	E66	Overweight and obesity	11,418<br>149,974	35.3%<br>17.5%	<0.00<br>1	0.411
1<br>2	Z68.3	Body mass index [BMI] 30-39, adult	3,285<br>32,977	10.1%<br>3.9%	<0.00<br>1	0.249
1<br>2	Z68.4	Body mass index [BMI] 40 or greater, adult	3,012<br>23,535	9.3%<br>2.7%	<0.00<br>1	0.278
1<br>2	Z68.25	Body mass index [BMI] 25.0-25.9, adult	119<br>2,293	0.4%<br>0.3%	0.001	0.018
1<br>2	Z68.26	Body mass index [BMI] 26.0-26.9, adult	150<br>2,433	0.5%<br>0.3%	<0.00<br>1	0.029
1<br>2	Z68.27	Body mass index [BMI] 27.0-27.9, adult	209<br>2,942	0.6%<br>0.3%	<0.00<br>1	0.043
1<br>2	Z68.28	Body mass index [BMI] 28.0-28.9, adult	264<br>3,317	0.8%<br>0.4%	<0.00<br>1	0.055
1<br>2	Z68.29	Body mass index [BMI] 29.0-29.9, adult	299<br>3,753	0.9%<br>0.4%	<0.00<br>1	0.059
1<br>2	F10	Alcohol related disorders	460<br>21,150	1.4%<br>2.5%	<0.00<br>1	0.076
1<br>2	Z80	Family history of primary malignant neoplasm	1,222<br>16,133	3.8%<br>1.9%	<0.00<br>1	0.114
1<br>2	Z15.0	Genetic susceptibility to malignant neoplasm	18<br>140	0.1%<br>0.0%	<0.00<br>1	0.021
1<br>2	Z12	Encounter for screening for malignant neoplasms	6,223<br>114,996	19.2%<br>13.4%	<0.00<br>1	0.157
1<br>2	Z85	Personal history of malignant neoplasm	1,111<br>23,231	3.4%<br>2.7%	<0.00<br>1	0.042
1<br>2	Z98.84	Bariatric surgery status	534<br>2,989	1.6%<br>0.3%	<0.00<br>1	0.131
1<br>2	F17	Nicotine dependence	2,399<br>77,335	7.4%<br>9.0%	<0.00<br>1	0.059

1<br>2	E66.0	Obesity due to excess calories		5,910<br>56,178	18.3%<br>6.6%	<0.00<br>1	0.361
1<br>2	E66.2	Morbid (severe) obesity with alveolar hypoventilation		230<br>2,051	0.7%<br>0.2%	<0.00<br>1	0.069
1<br>2	E66.3	Overweight		535<br>7,794	1.7%<br>0.9%	<0.00<br>1	0.066
1<br>2	E66.8	Other obesity		193<br>1,084	0.6%<br>0.1%	<0.00<br>1	0.078
1<br>2	E66.9	Obesity, unspecified		8,150<br>113,380	25.2%<br>13.2%	<0.00<br>1	0.307
1<br>2	Z72.0	Tobacco use		588<br>11,927	1.8%<br>1.4%	<0.00<br>1	0.034
1<br>2	Z85.09	Personal history of malignant neoplasm of other digestive organs		10<br>133	0.0%<br>0.0%	0.032	0.010
1<br>2	B96.8<br>1	Helicobacter pylori [H. pylori] as the cause of diseases classified elsewhere		141<br>3,386	0.4%<br>0.4%	0.259	0.006
1<br>2	D13.1	Benign neoplasm of stomach		163<br>2,312	0.5%<br>0.3%	<0.00<br>1	0.038
1<br>2	K31.7	Polyp of stomach and duodenum		216<br>2,635	0.7%<br>0.3%	<0.00<br>1	0.052
1<br>2	D51.0	Vitamin B12 deficiency anemia due to intrinsic factor deficiency		103<br>1,532	0.3%<br>0.2%	<0.00<br>1	0.028
1<br>2	Z80.0	Family history of malignant neoplasm of digestive organs		413<br>6,255	1.3%<br>0.7%	<0.00<br>1	0.055
1<br>2	D83	Common variable immunodeficiency		21<br>206	0.1%<br>0.0%	<0.00<br>1	0.019
1<br>2	B27	Infectious mononucleosis		30<br>409	0.1%<br>0.0%	<0.00<br>1	0.017
**Procedure**							
Group			Mean ± SD	Patients	% of Group	P-Value	SMD
1<br>2	10073<br>52	Excision Procedures on the Stomach		10<br>76	0.0%<br>0.0%	<0.00<br>1	0.016
**Medication**							

Group			Mean ± SD	Patients	% of Group	P-Value	SMD
1<br>2	A10A	INSULINS AND ANALOGUES		14,131<br>159,901	43.7%<br>18.7%	<0.001	0.560
1<br>2	A10BB	Sulfonylureas		5,464<br>69,045	16.9%<br>8.1%	<0.001	0.269
1<br>2	A10BF	Alpha glucosidase inhibitors		124<br>876	0.4%<br>0.1%	<0.001	0.057
1<br>2	A10BG	Thiazolidinediones		1,960<br>20,400	6.1%<br>2.4%	<0.001	0.183
1<br>2	A10BH	Dipeptidyl peptidase 4 (DPP-4) inhibitors		3,547<br>17,113	11.0%<br>2.0%	<0.001	0.370
1<br>2	A10BK	Sodium-glucose co-transporter 2 (SGLT2) inhibitors		1,839<br>3,371	5.7%<br>0.4%	<0.001	0.312
1<br>2	A10BX	Other blood glucose lowering drugs, excl. insulins		672<br>3,839	2.1%<br>0.4%	<0.001	0.146

Group 1 (N = 32,268) and group 2 (N = 32,268) characteristics after propensity score matching

Demographics

Group			Mean ± SD	Patients	% of Group	P-Value	SMD
1<br>2	AI	Age at Index	59.0 +/- 12.4<br>58.8 +/- 13.3	32,268<br>32,268	100%<br>100%	0.025	0.018
1<br>2	2106-3	White		21,162<br>21,585	65.6%<br>66.9%	<0.001	0.028
1<br>2	1002-5	American Indian or Alaska Native		98<br>103	0.3%<br>0.3%	0.724	0.003
1<br>2	UNK	Unknown Race		4,259<br>4,178	13.2%<br>12.9%	0.344	0.007
1<br>2	F	Female		17,213<br>17,550	53.3%<br>54.4%	0.008	0.021
1<br>2	2076-8	Native Hawaiian or Other Pacific Islander		172<br>165	0.5%<br>0.5%	0.702	0.003
1<br>2	UN	Unknown Gender		1,659<br>1,684	5.1%<br>5.2%	0.657	0.003
1<br>2	2186-5	Not Hispanic or Latino		22,225<br>22,662	68.9%<br>70.2%	<0.001	0.029

© 2024 Wang L et al. *JAMA Network Open.*

1<br>2	2135-2	Hispanic or Latino		2,654<br>2,468	8.2%<br>7.6%	0.007	0.021
1<br>2	2054-5	Black or African American		5,092<br>4,873	15.8%<br>15.1%	0.017	0.019
1<br>2	M	Male		13,396<br>13,034	41.5%<br>40.4%	0.004	0.023
1<br>2	2028-9	Asian		681<br>582	2.1%<br>1.8%	0.005	0.022

Diagnosis

Group			Mean ± SD	Patients	% of Group	P-Value	SMD
1<br>2	Z55-Z65	Persons with potential health hazards related to socioeconomic and psychosocial circumstances		419<br>328	1.3%<br>1.0%	0.001	0.026
1<br>2	E66	Overweight and obesity		11,344<br>11,249	35.2%<br>34.9%	0.433	0.006
1<br>2	Z68.3	Body mass index [BMI] 30-39, adult		3,252<br>3,141	10.1%<br>9.7%	0.144	0.012
1<br>2	Z68.4	Body mass index [BMI] 40 or greater, adult		2,970<br>2,850	9.2%<br>8.8%	0.099	0.013
1<br>2	Z68.25	Body mass index [BMI] 25.0-25.9, adult		119<br>96	0.4%<br>0.3%	0.116	0.012
1<br>2	Z68.26	Body mass index [BMI] 26.0-26.9, adult		150<br>109	0.5%<br>0.3%	0.011	0.020
1<br>2	Z68.27	Body mass index [BMI] 27.0-27.9, adult		209<br>179	0.6%<br>0.6%	0.127	0.012
1<br>2	Z68.28	Body mass index [BMI] 28.0-28.9, adult		263<br>224	0.8%<br>0.7%	0.076	0.014
1<br>2	Z68.29	Body mass index [BMI] 29.0-29.9, adult		299<br>250	0.9%<br>0.8%	0.036	0.017
1<br>2	F10	Alcohol related disorders		459<br>352	1.4%<br>1.1%	<0.001	0.030

© 2024 Wang L et al. *JAMA Network Open.*

1<br>2	Z80	Family history of primary malignant neoplasm	1,210<br>1,090	3.7%<br>3.4%	0.011	0.020
1<br>2	Z15.0	Genetic susceptibility to malignant neoplasm	18<br>18	0.1%<br>0.1%	1	<0.001
1<br>2	Z12	Encounter for screening for malignant neoplasms	6,175<br>5,575	19.1%<br>17.3%	<0.00<br>1	0.048
1<br>2	Z85	Personal history of malignant neoplasm	1,105<br>1,051	3.4%<br>3.3%	0.237	0.009
1<br>2	Z98.84	Bariatric surgery status	519<br>529	1.6%<br>1.6%	0.755	0.002
1<br>2	F17	Nicotine dependence	2,392<br>2,137	7.4%<br>6.6%	<0.00<br>1	0.031
1<br>2	E66.0	Obesity due to excess calories	5,850<br>5,811	18.1%<br>18.0%	0.690	0.003
1<br>2	E66.2	Morbid (severe) obesity with alveolar hypoventilation	228<br>202	0.7%<br>0.6%	0.208	0.010
1<br>2	E66.3	Overweight	531<br>467	1.6%<br>1.4%	0.041	0.016
1<br>2	E66.8	Other obesity	190<br>174	0.6%<br>0.5%	0.400	0.007
1<br>2	E66.9	Obesity, unspecified	8,103<br>8,038	25.1%<br>24.9%	0.555	0.005
1<br>2	Z72.0	Tobacco use	585<br>491	1.8%<br>1.5%	0.004	0.023
1<br>2	Z85.09	Personal history of malignant neoplasm of other digestive organs	10<br>10	0.0%<br>0.0%	1	<0.001
1<br>2	B96.8<br>1	Helicobacter pylori [H. pylori] as the cause of diseases classified elsewhere	141<br>97	0.4%<br>0.3%	0.004	0.022
1<br>2	D13.1	Benign neoplasm of stomach	163<br>134	0.5%<br>0.4%	0.092	0.013
1<br>2	K31.7	Polyp of stomach and duodenum	213<br>186	0.7%<br>0.6%	0.175	0.011
1<br>2	D51.0	Vitamin B12 deficiency anemia	103<br>86	0.3%<br>0.3%	0.216	0.010

© 2024 Wang L et al. *JAMA Network Open.*

		due to intrinsic factor deficiency					
1<br>2	Z80.0	Family history of malignant neoplasm of digestive organs		409<br>345	1.3%<br>1.1%	0.019	0.018
1<br>2	D83	Common variable immunodeficiency		21<br>18	0.1%<br>0.1%	0.631	0.004
1<br>2	B27	Infectious mononucleosis		30<br>28	0.1%<br>0.1%	0.793	0.002
**Procedure**							
Group			Mean ± SD	Patients	% of Group	P-Value	SMD
1<br>2	10073<br>52	Excision Procedures on the Stomach		10<br>10	0.0%<br>0.0%	1	<0.001
**Medication**							
Group			Mean ± SD	Patients	% of Group	P-Value	SMD
1<br>2	A10A	INSULINS AND ANALOGUES		14,042<br>13,998	43.5%<br>43.4%	0.727	0.003
1<br>2	A10B<br>B	Sulfonylureas		5,424<br>5,277	16.8%<br>16.4%	0.120	0.012
1<br>2	A10B<br>F	Alpha glucosidase inhibitors		124<br>110	0.4%<br>0.3%	0.359	0.007
1<br>2	A10B<br>G	Thiazolidinediones		1,932<br>1,926	6.0%<br>6.0%	0.921	0.001
1<br>2	A10B<br>H	Dipeptidyl peptidase 4 (DPP-4) inhibitors		3,470<br>3,356	10.8%<br>10.4%	0.145	0.011
1<br>2	A10B<br>K	Sodium-glucose co-transporter 2 (SGLT2) inhibitors		1,751<br>1,614	5.4%<br>5.0%	0.015	0.019
1<br>2	A10B<br>X	Other blood glucose lowering drugs, excl. insulins		660<br>664	2.0%<br>2.1%	0.912	0.001

© 2024 Wang L et al. *JAMA Network Open.*

eTable 20. Characteristics of the GLP-1RA/no metformin group and metformin/no GLP-1RA group before and after matched for covariates related to kidney cancer for the study populations of patients with T2D and no history of any OAC

Group 1 (N = 32,365) and group 2 (N = 856,160) characteristics before propensity score matching							
**Demographics**							
Group			Mean ± SD	Patients	% of Group	P-Value	SMD
1<br>2	AI	Age at Index	59.0 +/- 12.4<br>60.1 +/- 14.0	32,365<br>856,160	100%<br>100%	<0.001	0.080
1<br>2	2106-3	White		21,231<br>515,868	65.6%<br>60.3%	<0.001	0.111
1<br>2	1002-5	American Indian or Alaska Native		98<br>3,080	0.3%<br>0.4%	0.092	0.010
1<br>2	UNK	Unknown Race		4,272<br>119,036	13.2%<br>13.9%	<0.001	0.021
1<br>2	F	Female		17,278<br>399,923	53.4%<br>46.7%	<0.001	0.134
1<br>2	2076-8	Native Hawaiian or Other Pacific Islander		172<br>5,884	0.5%<br>0.7%	0.001	0.020
1<br>2	UN	Unknown Gender		1,670<br>19,592	5.2%<br>2.3%	<0.001	0.152
1<br>2	2186-5	Not Hispanic or Latino		22,299<br>528,686	68.9%<br>61.8%	<0.001	0.151
1<br>2	2135-2	Hispanic or Latino		2,660<br>91,303	8.2%<br>10.7%	<0.001	0.084
1<br>2	2054-5	Black or African American		5,107<br>141,606	15.8%<br>16.5%	<0.001	0.021
1<br>2	M	Male		13,417<br>436,645	41.5%<br>51.0%	<0.001	0.192
1<br>2	2028-9	Asian		681<br>35,540	2.1%<br>4.2%	<0.001	0.118
**Diagnosis**							
Group			Mean ± SD	Patients	% of Group	P-Value	SMD
1<br>2	Z55-Z65	Persons with potential health hazards related to socioeconomic and		422<br>10,310	1.3%<br>1.2%	0.107	0.009

© 2024 Wang L et al. *JAMA Network Open.*

		psychosocial circumstances				
1<br>2	E66	Overweight and obesity	11,418<br>149,974	35.3%<br>17.5%	<0.001	0.411
1<br>2	Z68.3	Body mass index [BMI] 30-39, adult	3,285<br>32,977	10.1%<br>3.9%	<0.001	0.249
1<br>2	Z68.4	Body mass index [BMI] 40 or greater, adult	3,012<br>23,535	9.3%<br>2.7%	<0.001	0.278
1<br>2	Z68.25	Body mass index [BMI] 25.0-25.9, adult	119<br>2,293	0.4%<br>0.3%	0.001	0.018
1<br>2	Z68.26	Body mass index [BMI] 26.0-26.9, adult	150<br>2,433	0.5%<br>0.3%	<0.001	0.029
1<br>2	Z68.27	Body mass index [BMI] 27.0-27.9, adult	209<br>2,942	0.6%<br>0.3%	<0.001	0.043
1<br>2	Z68.28	Body mass index [BMI] 28.0-28.9, adult	264<br>3,317	0.8%<br>0.4%	<0.001	0.055
1<br>2	Z68.29	Body mass index [BMI] 29.0-29.9, adult	299<br>3,753	0.9%<br>0.4%	<0.001	0.059
1<br>2	Z80	Family history of primary malignant neoplasm	1,222<br>16,133	3.8%<br>1.9%	<0.001	0.114
1<br>2	Z15.0	Genetic susceptibility to malignant neoplasm	18<br>140	0.1%<br>0.0%	<0.001	0.021
1<br>2	Z12	Encounter for screening for malignant neoplasms	6,223<br>114,996	19.2%<br>13.4%	<0.001	0.157
1<br>2	Z85	Personal history of malignant neoplasm	1,111<br>23,231	3.4%<br>2.7%	<0.001	0.042
1<br>2	Z98.84	Bariatric surgery status	534<br>2,989	1.6%<br>0.3%	<0.001	0.131
1<br>2	I10	Essential (primary) hypertension	19,775<br>413,462	61.1%<br>48.3%	<0.001	0.259
1<br>2	F17	Nicotine dependence	2,399<br>77,335	7.4%<br>9.0%	<0.001	0.059

© 2024 Wang L et al. *JAMA Network Open.*

1<br>2	E66.0	Obesity due to excess calories		5,910<br>56,178	18.3%<br>6.6%	<0.001	0.361
1<br>2	E66.2	Morbid (severe) obesity with alveolar hypoventilation		230<br>2,051	0.7%<br>0.2%	<0.001	0.069
1<br>2	E66.3	Overweight		535<br>7,794	1.7%<br>0.9%	<0.001	0.066
1<br>2	E66.8	Other obesity		193<br>1,084	0.6%<br>0.1%	<0.001	0.078
1<br>2	E66.9	Obesity, unspecified		8,150<br>113,380	25.2%<br>13.2%	<0.001	0.307
1<br>2	Z72.0	Tobacco use		588<br>11,927	1.8%<br>1.4%	<0.001	0.034
1<br>2	Z80.51	Family history of malignant neoplasm of kidney		12<br>129	0.0%<br>0.0%	0.002	0.014
1<br>2	N18	Chronic kidney disease (CKD)		6,496<br>35,429	20.1%<br>4.1%	<0.001	0.504
1<br>2	N19	Unspecified kidney failure		530<br>2,836	1.6%<br>0.3%	<0.001	0.133
1<br>2	Z99.2	Dependence on renal dialysis		548<br>979	1.7%<br>0.1%	<0.001	0.167
1<br>2	N17-N19	Acute kidney failure and chronic kidney disease		7,323<br>56,470	22.6%<br>6.6%	<0.001	0.466
1<br>2	Q85.83	Von Hippel-Lindau syndrome		0<br>10	0%<br>0.0%	0.539	0.005
1<br>2	Q85.1	Tuberous sclerosis		0<br>37	0%<br>0.0%	0.237	0.009
1<br>2	D57.1	Sickle-cell disease without crisis		10<br>289	0.0%<br>0.0%	0.783	0.002
1<br>2	D30.0	Benign neoplasm of kidney		33<br>301	0.1%<br>0.0%	<0.001	0.026
**Medication**							
Group			Mean ± SD	Patients	% of Group	P-Value	SMD
1<br>2	A10A	INSULINS AND ANALOGUES		14,131<br>159,901	43.7%<br>18.7%	<0.001	0.560
1<br>2	A10BB	Sulfonylureas		5,464<br>69,045	16.9%<br>8.1%	<0.001	0.269

© 2024 Wang L et al. *JAMA Network Open.*

1<br>2	A10BF	Alpha glucosidase inhibitors		124<br>876	0.4%<br>0.1%	<0.001	0.057
1<br>2	A10BG	Thiazolidinediones		1,960<br>20,400	6.1%<br>2.4%	<0.001	0.183
1<br>2	A10BH	Dipeptidyl peptidase 4 (DPP-4) inhibitors		3,547<br>17,113	11.0%<br>2.0%	<0.001	0.370
1<br>2	A10BK	Sodium-glucose co-transporter 2 (SGLT2) inhibitors		1,839<br>3,371	5.7%<br>0.4%	<0.001	0.312
1<br>2	A10BX	Other blood glucose lowering drugs, excl. insulins		672<br>3,839	2.1%<br>0.4%	<0.001	0.146
1<br>2	161	acetaminophen		10,835<br>238,323	33.5%<br>27.8%	<0.001	0.123

Group 1 (N = 32,162) and group 2 (N = 32,162) characteristics after propensity score matching

Demographics

Group			Mean ± SD	Patients	% of Group	P-Value	SMD
1<br>2	AI	Age at Index	59.0 +/- 12.4<br>59.0 +/- 13.7	32,162<br>32,162	100%<br>100%	0.912	0.001
1<br>2	2106-3	White		21,094<br>21,683	65.6%<br>67.4%	<0.001	0.039
1<br>2	1002-5	American Indian or Alaska Native		98<br>102	0.3%<br>0.3%	0.777	0.002
1<br>2	UNK	Unknown Race		4,244<br>4,043	13.2%<br>12.6%	0.018	0.019
1<br>2	F	Female		17,170<br>17,352	53.4%<br>54.0%	0.150	0.011
1<br>2	2076-8	Native Hawaiian or Other Pacific Islander		172<br>153	0.5%<br>0.5%	0.291	0.008
1<br>2	UN	Unknown Gender		1,648<br>1,693	5.1%<br>5.3%	0.424	0.006
1<br>2	2186-5	Not Hispanic or Latino		22,153<br>22,767	68.9%<br>70.8%	<0.001	0.042
1<br>2	2135-2	Hispanic or Latino		2,642<br>2,283	8.2%<br>7.1%	<0.001	0.042
1<br>2	2054-5	Black or African American		5,076<br>4,856	15.8%<br>15.1%	0.016	0.019

1<br>2	M	Male		13,344<br>13,117	41.5%<br>40.8%	0.069	0.014
1<br>2	2028-9	Asian		677<br>581	2.1%<br>1.8%	0.006	0.022

Diagnosis

Group			Mean ± SD	Patients	% of Group	P-Value	SMD
1<br>2	Z55-Z65	Persons with potential health hazards related to socioeconomic and psychosocial circumstances		417<br>370	1.3%<br>1.2%	0.092	0.013
1<br>2	E66	Overweight and obesity		11,266<br>11,139	35.0%<br>34.6%	0.293	0.008
1<br>2	Z68.3	Body mass index [BMI] 30-39, adult		3,223<br>3,159	10.0%<br>9.8%	0.399	0.007
1<br>2	Z68.4	Body mass index [BMI] 40 or greater, adult		2,929<br>2,900	9.1%<br>9.0%	0.690	0.003
1<br>2	Z68.25	Body mass index [BMI] 25.0-25.9, adult		118<br>96	0.4%<br>0.3%	0.132	0.012
1<br>2	Z68.26	Body mass index [BMI] 26.0-26.9, adult		148<br>121	0.5%<br>0.4%	0.099	0.013
1<br>2	Z68.27	Body mass index [BMI] 27.0-27.9, adult		207<br>159	0.6%<br>0.5%	0.012	0.020
1<br>2	Z68.28	Body mass index [BMI] 28.0-28.9, adult		259<br>249	0.8%<br>0.8%	0.656	0.004
1<br>2	Z68.29	Body mass index [BMI] 29.0-29.9, adult		296<br>283	0.9%<br>0.9%	0.587	0.004
1<br>2	Z80	Family history of primary malignant neoplasm		1,195<br>1,136	3.7%<br>3.5%	0.213	0.010
1<br>2	Z15.0	Genetic susceptibility to malignant neoplasm		18<br>13	0.1%<br>0.0%	0.369	0.007

© 2024 Wang L et al. *JAMA Network Open.*

1<br>2	Z12	Encounter for screening for malignant neoplasms	6,128<br>5,534	19.1%<br>17.2%	<0.001	0.048
1<br>2	Z85	Personal history of malignant neoplasm	1,096<br>1,003	3.4%<br>3.1%	0.039	0.016
1<br>2	Z98.84	Bariatric surgery status	510<br>506	1.6%<br>1.6%	0.899	0.001
1<br>2	I10	Essential (primary) hypertension	19,593<br>19,079	60.9%<br>59.3%	<0.001	0.033
1<br>2	F17	Nicotine dependence	2,384<br>2,112	7.4%<br>6.6%	<0.001	0.033
1<br>2	E66.0	Obesity due to excess calories	5,798<br>5,762	18.0%<br>17.9%	0.712	0.003
1<br>2	E66.2	Morbid (severe) obesity with alveolar hypoventilation	226<br>189	0.7%<br>0.6%	0.068	0.014
1<br>2	E66.3	Overweight	529<br>478	1.6%<br>1.5%	0.105	0.013
1<br>2	E66.8	Other obesity	190<br>170	0.6%<br>0.5%	0.290	0.008
1<br>2	E66.9	Obesity, unspecified	8,037<br>7,931	25.0%<br>24.7%	0.333	0.008
1<br>2	Z72.0	Tobacco use	579<br>507	1.8%<br>1.6%	0.028	0.017
1<br>2	Z80.51	Family history of malignant neoplasm of kidney	12<br>10	0.0%<br>0.0%	0.670	0.003
1<br>2	N18	Chronic kidney disease (CKD)	6,366<br>6,320	19.8%<br>19.7%	0.649	0.004
1<br>2	N19	Unspecified kidney failure	510<br>492	1.6%<br>1.5%	0.567	0.005
1<br>2	Z99.2	Dependence on renal dialysis	514<br>477	1.6%<br>1.5%	0.236	0.009
1<br>2	N17-N19	Acute kidney failure and chronic kidney disease	7,189<br>7,105	22.4%<br>22.1%	0.426	0.006
1<br>2	Q85.83	Von Hippel-Lindau syndrome	0<br>0	0%<br>0%	--	--
1<br>2	Q85.1	Tuberous sclerosis	0<br>0	0%<br>0%	--	--
1<br>2	D57.1	Sickle-cell disease without crisis	10<br>11	0.0%<br>0.0%	0.827	0.002

© 2024 Wang L et al. *JAMA Network Open.*

1<br>2	D30.0	Benign neoplasm of kidney		33<br>21	0.1%<br>0.1%	0.102	0.013

Medication

Group			Mean ± SD	Patients	% of Group	P-Value	SMD
1<br>2	A10A	INSULINS AND ANALOGUES		13,947<br>13,899	43.4%<br>43.2%	0.702	0.003
1<br>2	A10BB	Sulfonylureas		5,373<br>5,299	16.7%<br>16.5%	0.433	0.006
1<br>2	A10BF	Alpha glucosidase inhibitors		120<br>100	0.4%<br>0.3%	0.177	0.011
1<br>2	A10BG	Thiazolidinediones		1,907<br>1,800	5.9%<br>5.6%	0.070	0.014
1<br>2	A10BH	Dipeptidyl peptidase 4 (DPP-4) inhibitors		3,406<br>3,241	10.6%<br>10.1%	0.033	0.017
1<br>2	A10BK	Sodium-glucose co-transporter 2 (SGLT2) inhibitors		1,696<br>1,560	5.3%<br>4.9%	0.014	0.019
1<br>2	A10BX	Other blood glucose lowering drugs, excl. insulins		650<br>569	2.0%<br>1.8%	0.019	0.018
1<br>2	161	acetaminophen		10,744<br>10,591	33.4%<br>32.9%	0.200	0.010

© 2024 Wang L et al. *JAMA Network Open.*

eTable 21. Characteristics of the GLP-1RA/no metformin group and metformin/no GLP-1RA group before and after matched for covariates related to liver cancer for the study populations of patients with T2D and no history of any OAC

Group 1 (N = 32,365) and group 2 (N = 856,160) characteristics before propensity score matching							
**Demographics**							
Group			Mean ± SD	Patients	% of Group	P-Value	SMD
1<br>2	AI	Age at Index	59.0 +/- 12.4<br>60.1 +/- 14.0	32,365<br>856,160	100%<br>100%	<0.001	0.080
1<br>2	2106-3	White		21,231<br>515,868	65.6%<br>60.3%	<0.001	0.111
1<br>2	1002-5	American Indian or Alaska Native		98<br>3,080	0.3%<br>0.4%	0.092	0.010
1<br>2	UNK	Unknown Race		4,272<br>119,036	13.2%<br>13.9%	<0.001	0.021
1<br>2	F	Female		17,278<br>399,923	53.4%<br>46.7%	<0.001	0.134
1<br>2	2076-8	Native Hawaiian or Other Pacific Islander		172<br>5,884	0.5%<br>0.7%	0.001	0.020
1<br>2	UN	Unknown Gender		1,670<br>19,592	5.2%<br>2.3%	<0.001	0.152
1<br>2	2186-5	Not Hispanic or Latino		22,299<br>528,686	68.9%<br>61.8%	<0.001	0.151
1<br>2	2135-2	Hispanic or Latino		2,660<br>91,303	8.2%<br>10.7%	<0.001	0.084
1<br>2	2054-5	Black or African American		5,107<br>141,606	15.8%<br>16.5%	<0.001	0.021
1<br>2	M	Male		13,417<br>436,645	41.5%<br>51.0%	<0.001	0.192
1<br>2	2028-9	Asian		681<br>35,540	2.1%<br>4.2%	<0.001	0.118
**Diagnosis**							
Group			Mean ± SD	Patients	% of Group	P-Value	SMD
1<br>2	Z55-Z65	Persons with potential health hazards related to socioeconomic and		422<br>10,310	1.3%<br>1.2%	0.107	0.009

© 2024 Wang L et al. *JAMA Network Open.*

		psychosocial circumstances				
1<br>2	E66	Overweight and obesity	11,418<br>149,974	35.3%<br>17.5%	<0.00<br>1	0.411
1<br>2	Z68.3	Body mass index [BMI] 30-39, adult	3,285<br>32,977	10.1%<br>3.9%	<0.00<br>1	0.249
1<br>2	Z68.4	Body mass index [BMI] 40 or greater, adult	3,012<br>23,535	9.3%<br>2.7%	<0.00<br>1	0.278
1<br>2	Z68.25	Body mass index [BMI] 25.0-25.9, adult	119<br>2,293	0.4%<br>0.3%	0.001	0.018
1<br>2	Z68.26	Body mass index [BMI] 26.0-26.9, adult	150<br>2,433	0.5%<br>0.3%	<0.00<br>1	0.029
1<br>2	Z68.27	Body mass index [BMI] 27.0-27.9, adult	209<br>2,942	0.6%<br>0.3%	<0.00<br>1	0.043
1<br>2	Z68.28	Body mass index [BMI] 28.0-28.9, adult	264<br>3,317	0.8%<br>0.4%	<0.00<br>1	0.055
1<br>2	Z68.29	Body mass index [BMI] 29.0-29.9, adult	299<br>3,753	0.9%<br>0.4%	<0.00<br>1	0.059
1<br>2	F10	Alcohol related disorders	460<br>21,150	1.4%<br>2.5%	<0.00<br>1	0.076
1<br>2	Z80	Family history of primary malignant neoplasm	1,222<br>16,133	3.8%<br>1.9%	<0.00<br>1	0.114
1<br>2	Z15.0	Genetic susceptibility to malignant neoplasm	18<br>140	0.1%<br>0.0%	<0.00<br>1	0.021
1<br>2	Z12	Encounter for screening for malignant neoplasms	6,223<br>114,996	19.2%<br>13.4%	<0.00<br>1	0.157
1<br>2	Z85	Personal history of malignant neoplasm	1,111<br>23,231	3.4%<br>2.7%	<0.00<br>1	0.042
1<br>2	Z98.84	Bariatric surgery status	534<br>2,989	1.6%<br>0.3%	<0.00<br>1	0.131
1<br>2	E88.81	Metabolic syndrome and other insulin resistance	705<br>8,208	2.2%<br>1.0%	<0.00<br>1	0.098

© 2024 Wang L et al. *JAMA Network Open.*

1<br>2	E78	Disorders of lipoprotein metabolism and other lipidemias	18,042<br>341,946	55.7%<br>39.9%	<0.001	0.320
1<br>2	I10	Essential (primary) hypertension	19,775<br>413,462	61.1%<br>48.3%	<0.001	0.259
1<br>2	K75.81	Nonalcoholic steatohepatitis (NASH)	305<br>1,860	0.9%<br>0.2%	<0.001	0.096
1<br>2	F17	Nicotine dependence	2,399<br>77,335	7.4%<br>9.0%	<0.001	0.059
1<br>2	E66.0	Obesity due to excess calories	5,910<br>56,178	18.3%<br>6.6%	<0.001	0.361
1<br>2	E66.2	Morbid (severe) obesity with alveolar hypoventilation	230<br>2,051	0.7%<br>0.2%	<0.001	0.069
1<br>2	E66.3	Overweight	535<br>7,794	1.7%<br>0.9%	<0.001	0.066
1<br>2	E66.8	Other obesity	193<br>1,084	0.6%<br>0.1%	<0.001	0.078
1<br>2	E66.9	Obesity, unspecified	8,150<br>113,380	25.2%<br>13.2%	<0.001	0.307
1<br>2	Z72.0	Tobacco use	588<br>11,927	1.8%<br>1.4%	<0.001	0.034
1<br>2	Z72.3	Lack of physical exercise	20<br>251	0.1%<br>0.0%	0.001	0.015
1<br>2	Z72.4	Inappropriate diet and eating habits	43<br>118	0.1%<br>0.0%	<0.001	0.044
1<br>2	B18	Chronic viral hepatitis	352<br>7,813	1.1%<br>0.9%	0.001	0.018
1<br>2	K74	Fibrosis and cirrhosis of liver	681<br>7,900	2.1%<br>0.9%	<0.001	0.097
1<br>2	K76.0	Fatty (change of) liver, not elsewhere classified	1,474<br>21,684	4.6%<br>2.5%	<0.001	0.110
1<br>2	K74.3	Primary biliary cirrhosis	28<br>287	0.1%<br>0.0%	<0.001	0.022
1<br>2	E83.110	Hereditary hemochromatosis	24<br>266	0.1%<br>0.0%	<0.001	0.019
1<br>2	E70.21	Tyrosinemia	10<br>46	0.0%<br>0.0%	<0.001	0.019

© 2024 Wang L et al. *JAMA Network Open.*

1<br>2	E88.01	Alpha-1-antitrypsin deficiency		15<br>104	0.0%<br>0.0%	<0.00<br>1	0.020
1<br>2	E80.1	Porphyria cutanea tarda		10<br>20	0.0%<br>0.0%	<0.00<br>1	0.022
1<br>2	E74.0	Glycogen storage disease		10<br>60	0.0%<br>0.0%	<0.00<br>1	0.017
1<br>2	E83.00	Disorder of copper metabolism, unspecified		10<br>61	0.0%<br>0.0%	<0.00<br>1	0.017
1<br>2	T64	Toxic effect of aflatoxin and other mycotoxin food contaminants		10<br>132	0.0%<br>0.0%	0.031	0.010
1<br>2	D13.4	Benign neoplasm of liver		16<br>305	0.0%<br>0.0%	0.199	0.007
**Medication**							
Group			Mean ± SD	Patients	% of Group	P-Value	SMD
1<br>2	A10A	INSULINS AND ANALOGUES		14,131<br>159,901	43.7%<br>18.7%	<0.00<br>1	0.560
1<br>2	A10B<br>B	Sulfonylureas		5,464<br>69,045	16.9%<br>8.1%	<0.00<br>1	0.269
1<br>2	A10B<br>F	Alpha glucosidase inhibitors		124<br>876	0.4%<br>0.1%	<0.00<br>1	0.057
1<br>2	A10B<br>G	Thiazolidinediones		1,960<br>20,400	6.1%<br>2.4%	<0.00<br>1	0.183
1<br>2	A10B<br>H	Dipeptidyl peptidase 4 (DPP-4) inhibitors		3,547<br>17,113	11.0%<br>2.0%	<0.00<br>1	0.370
1<br>2	A10B<br>K	Sodium-glucose co-transporter 2 (SGLT2) inhibitors		1,839<br>3,371	5.7%<br>0.4%	<0.00<br>1	0.312
1<br>2	A10B<br>X	Other blood glucose lowering drugs, excl. insulins		672<br>3,839	2.1%<br>0.4%	<0.00<br>1	0.146
1<br>2	A14A	ANABOLIC STEROIDS		17<br>399	0.1%<br>0.0%	0.629	0.003
**Group 1 (N = 32,267) and group 2 (N = 32,267) characteristics after propensity score matching**							
**Demographics**							
Group			Mean ± SD	Patients	% of Group	P-Value	SMD

© 2024 Wang L et al. *JAMA Network Open.*

1<br>2	AI	Age at Index	59.0 +/- 12.4<br>58.8 +/- 13.4	32,267<br>32,267	100%<br>100%	0.088	0.013
1<br>2	2106-3	White		21,161<br>21,688	65.6%<br>67.2%	<0.00<br>1	0.035
1<br>2	1002-5	American Indian or Alaska Native		98<br>100	0.3%<br>0.3%	0.887	0.001
1<br>2	UNK	Unknown Race		4,256<br>4,149	13.2%<br>12.9%	0.211	0.010
1<br>2	F	Female		17,218<br>17,367	53.4%<br>53.8%	0.240	0.009
1<br>2	2076-8	Native Hawaiian or Other Pacific Islander		172<br>173	0.5%<br>0.5%	0.957	<0.001
1<br>2	UN	Unknown Gender		1,658<br>1,752	5.1%<br>5.4%	0.098	0.013
1<br>2	2186-5	Not Hispanic or Latino		22,227<br>22,704	68.9%<br>70.4%	<0.00<br>1	0.032
1<br>2	2135-2	Hispanic or Latino		2,652<br>2,438	8.2%<br>7.6%	0.002	0.025
1<br>2	2054-5	Black or African American		5,095<br>4,824	15.8%<br>15.0%	0.003	0.023
1<br>2	M	Male		13,391<br>13,148	41.5%<br>40.7%	0.052	0.015
1<br>2	2028-9	Asian		681<br>588	2.1%<br>1.8%	0.008	0.021

Diagnosis

Group			Mean ± SD	Patients	% of Group	P-Value	SMD
1<br>2	Z55-Z65	Persons with potential health hazards related to socioeconomic and psychosocial circumstances		417<br>355	1.3%<br>1.1%	0.025	0.018
1<br>2	E66	Overweight and obesity		11,344<br>11,263	35.2%<br>34.9%	0.504	0.005
1<br>2	Z68.3	Body mass index [BMI] 30-39, adult		3,251<br>3,270	10.1%<br>10.1%	0.804	0.002
1<br>2	Z68.4	Body mass index [BMI] 40 or greater, adult		2,968<br>2,851	9.2%<br>8.8%	0.108	0.013

© 2024 Wang L et al. *JAMA Network Open.*

1<br>2	Z68.25	Body mass index [BMI] 25.0-25.9, adult	118<br>89	0.4%<br>0.3%	0.043	0.016
1<br>2	Z68.26	Body mass index [BMI] 26.0-26.9, adult	149<br>118	0.5%<br>0.4%	0.057	0.015
1<br>2	Z68.27	Body mass index [BMI] 27.0-27.9, adult	208<br>172	0.6%<br>0.5%	0.064	0.015
1<br>2	Z68.28	Body mass index [BMI] 28.0-28.9, adult	262<br>232	0.8%<br>0.7%	0.175	0.011
1<br>2	Z68.29	Body mass index [BMI] 29.0-29.9, adult	298<br>292	0.9%<br>0.9%	0.804	0.002
1<br>2	F10	Alcohol related disorders	460<br>343	1.4%<br>1.1%	<0.001	0.033
1<br>2	Z80	Family history of primary malignant neoplasm	1,207<br>1,089	3.7%<br>3.4%	0.012	0.020
1<br>2	Z15.0	Genetic susceptibility to malignant neoplasm	18<br>13	0.1%<br>0.0%	0.369	0.007
1<br>2	Z12	Encounter for screening for malignant neoplasms	6,179<br>5,497	19.1%<br>17.0%	<0.001	0.055
1<br>2	Z85	Personal history of malignant neoplasm	1,106<br>992	3.4%<br>3.1%	0.011	0.020
1<br>2	Z98.84	Bariatric surgery status	519<br>521	1.6%<br>1.6%	0.950	<0.001
1<br>2	E88.81	Metabolic syndrome and other insulin resistance	695<br>624	2.2%<br>1.9%	0.048	0.016
1<br>2	E78	Disorders of lipoprotein metabolism and other lipidemias	17,957<br>17,449	55.7%<br>54.1%	<0.001	0.032
1<br>2	I10	Essential (primary) hypertension	19,692<br>19,128	61.0%<br>59.3%	<0.001	0.036
1<br>2	K75.81	Nonalcoholic steatohepatitis (NASH)	293<br>305	0.9%<br>0.9%	0.622	0.004

© 2024 Wang L et al. *JAMA Network Open.*

1<br>2	F17	Nicotine dependence	2,394<br>2,074	7.4%<br>6.4%	<0.00<br>1	0.039
1<br>2	E66.0	Obesity due to excess calories	5,852<br>5,865	18.1%<br>18.2%	0.894	0.001
1<br>2	E66.2	Morbid (severe) obesity with alveolar hypoventilation	227<br>224	0.7%<br>0.7%	0.887	0.001
1<br>2	E66.3	Overweight	534<br>456	1.7%<br>1.4%	0.012	0.020
1<br>2	E66.8	Other obesity	189<br>195	0.6%<br>0.6%	0.759	0.002
1<br>2	E66.9	Obesity, unspecified	8,104<br>7,965	25.1%<br>24.7%	0.206	0.010
1<br>2	Z72.0	Tobacco use	584<br>514	1.8%<br>1.6%	0.033	0.017
1<br>2	Z72.3	Lack of physical exercise	20<br>10	0.1%<br>0.0%	0.068	0.014
1<br>2	Z72.4	Inappropriate diet and eating habits	42<br>23	0.1%<br>0.1%	0.018	0.019
1<br>2	B18	Chronic viral hepatitis	350<br>301	1.1%<br>0.9%	0.054	0.015
1<br>2	K74	Fibrosis and cirrhosis of liver	666<br>652	2.1%<br>2.0%	0.697	0.003
1<br>2	K76.0	Fatty (change of) liver, not elsewhere classified	1,454<br>1,304	4.5%<br>4.0%	0.004	0.023
1<br>2	K74.3	Primary biliary cirrhosis	27<br>33	0.1%<br>0.1%	0.438	0.006
1<br>2	E83.11<br>0	Hereditary hemochromatosis	24<br>18	0.1%<br>0.1%	0.354	0.007
1<br>2	E70.21	Tyrosinemia	10<br>10	0.0%<br>0.0%	1	<0.001
1<br>2	E88.01	Alpha-1-antitrypsin deficiency	14<br>15	0.0%<br>0.0%	0.853	0.001
1<br>2	E80.1	Porphyria cutanea tarda	10<br>10	0.0%<br>0.0%	1	<0.001
1<br>2	E74.0	Glycogen storage disease	10<br>10	0.0%<br>0.0%	1	<0.001
1<br>2	E83.00	Disorder of copper metabolism, unspecified	10<br>10	0.0%<br>0.0%	1	<0.001

© 2024 Wang L et al. *JAMA Network Open.*

1<br>2	T64	Toxic effect of aflatoxin and other mycotoxin food contaminants		10<br>10	0.0%<br>0.0%	1	<0.001
1<br>2	D13.4	Benign neoplasm of liver		16<br>14	0.0%<br>0.0%	0.715	0.003
**Medication**							
Group			Mean ± SD	Patients	% of Group	P-Value	SMD
1<br>2	A10A	INSULINS AND ANALOGUES		14,044<br>14,092	43.5%<br>43.7%	0.703	0.003
1<br>2	A10B<br>B	Sulfonylureas		5,425<br>5,258	16.8%<br>16.3%	0.077	0.014
1<br>2	A10B<br>F	Alpha glucosidase inhibitors		122<br>123	0.4%<br>0.4%	0.949	0.001
1<br>2	A10B<br>G	Thiazolidinediones		1,933<br>1,850	6.0%<br>5.7%	0.164	0.011
1<br>2	A10B<br>H	Dipeptidyl peptidase 4 (DPP-4) inhibitors		3,476<br>3,326	10.8%<br>10.3%	0.054	0.015
1<br>2	A10B<br>K	Sodium-glucose co-transporter 2 (SGLT2) inhibitors		1,749<br>1,559	5.4%<br>4.8%	0.001	0.027
1<br>2	A10B<br>X	Other blood glucose lowering drugs, excl. insulins		656<br>644	2.0%<br>2.0%	0.737	0.003
1<br>2	A14A	ANABOLIC STEROIDS		17<br>25	0.1%<br>0.1%	0.217	0.010

© 2024 Wang L et al. *JAMA Network Open.*

eTable 22. Characteristics of the GLP-1RA/no metformin group and metformin/no GLP-1RA group before and after matched for covariates related to ovarian cancer for the study populations of women with T2D and no history of any OAC

Group 1 (N = 17,278) and group 2 (N = 399,923) characteristics before propensity score matching

Demographics

Group			Mean ± SD	Patients	% of Group	P-Value	SMD
1<br>2	AI	Age at Index	57.7 +/- 12.8<br>60.0 +/- 14.7	17,278<br>399,923	100%<br>100%	<0.001	0.160
1<br>2	2106-3	White		11,336<br>231,828	65.6%<br>58.0%	<0.001	0.158
1<br>2	1002-5	American Indian or Alaska Native		59<br>1,552	0.3%<br>0.4%	0.334	0.008
1<br>2	UNK	Unknown Race		1,563<br>49,384	9.0%<br>12.3%	<0.001	0.107
1<br>2	2076-8	Native Hawaiian or Other Pacific Islander		80<br>2,869	0.5%<br>0.7%	<0.001	0.033
1<br>2	UN	Unknown Ethnicity		3,145<br>103,904	18.2%<br>26.0%	<0.001	0.188
1<br>2	2186-5	Not Hispanic or Latino		12,481<br>247,896	72.2%<br>62.0%	<0.001	0.219
1<br>2	2135-2	Hispanic or Latino		1,652<br>48,123	9.6%<br>12.0%	<0.001	0.080
1<br>2	2054-5	Black or African American		3,476<br>80,016	20.1%<br>20.0%	0.723	0.003
1<br>2	2028-9	Asian		311<br>17,483	1.8%<br>4.4%	<0.001	0.149

Diagnosis

Group			Mean ± SD	Patients	% of Group	P-Value	SMD
1<br>2	Z55-Z65	Persons with potential health hazards related to socioeconomic and psychosocial circumstances		270<br>5,204	1.6%<br>1.3%	0.003	0.022
1<br>2	E66	Overweight and obesity		6,461<br>78,874	37.4%<br>19.7%	<0.001	0.399

© 2024 Wang L et al. *JAMA Network Open.*

1<br>2	Z68.3	Body mass index [BMI] 30-39, adult	1,360<br>13,007	7.9%<br>3.3%	<0.001	0.203
1<br>2	Z68.4	Body mass index [BMI] 40 or greater, adult	1,671<br>12,991	9.7%<br>3.2%	<0.001	0.264
1<br>2	Z68.25	Body mass index [BMI] 25.0-25.9, adult	39<br>643	0.2%<br>0.2%	0.039	0.015
1<br>2	Z68.26	Body mass index [BMI] 26.0-26.9, adult	52<br>616	0.3%<br>0.2%	<0.001	0.031
1<br>2	Z68.27	Body mass index [BMI] 27.0-27.9, adult	59<br>760	0.3%<br>0.2%	<0.001	0.029
1<br>2	Z68.28	Body mass index [BMI] 28.0-28.9, adult	69<br>862	0.4%<br>0.2%	<0.001	0.033
1<br>2	Z68.29	Body mass index [BMI] 29.0-29.9, adult	76<br>1,029	0.4%<br>0.3%	<0.001	0.031
1<br>2	F10	Alcohol related disorders	128<br>4,677	0.7%<br>1.2%	<0.001	0.044
1<br>2	Z80	Family history of primary malignant neoplasm	821<br>9,798	4.8%<br>2.4%	<0.001	0.124
1<br>2	Z15.0	Genetic susceptibility to malignant neoplasm	15<br>91	0.1%<br>0.0%	<0.001	0.027
1<br>2	Z12	Encounter for screening for malignant neoplasms	4,113<br>71,089	23.8%<br>17.8%	<0.001	0.149
1<br>2	Z85	Personal history of malignant neoplasm	492<br>9,342	2.8%<br>2.3%	<0.001	0.032
1<br>2	Z98.84	Bariatric surgery status	378<br>2,056	2.2%<br>0.5%	<0.001	0.145
1<br>2	F17	Nicotine dependence	1,246<br>31,324	7.2%<br>7.8%	0.003	0.024
1<br>2	E66.0	Obesity due to excess calories	3,456<br>31,701	20.0%<br>7.9%	<0.001	0.354
1<br>2	E66.2	Morbid (severe) obesity with alveolar hypoventilation	132<br>1,060	0.8%<br>0.3%	<0.001	0.070

© 2024 Wang L et al. *JAMA Network Open.*

1<br>2	E66.3	Overweight		280<br>3,619	1.6%<br>0.9%	<0.001	0.064
1<br>2	E66.8	Other obesity		118<br>574	0.7%<br>0.1%	<0.001	0.084
1<br>2	E66.9	Obesity, unspecified		4,568<br>59,095	26.4%<br>14.8%	<0.001	0.291
1<br>2	Z72.0	Tobacco use		334<br>4,947	1.9%<br>1.2%	<0.001	0.056
1<br>2	Z79.890	Hormone replacement therapy		85<br>1,319	0.5%<br>0.3%	<0.001	0.025
1<br>2	Z92.23	Personal history of estrogen therapy		10<br>115	0.1%<br>0.0%	0.030	0.014
1<br>2	Z80.3	Family history of malignant neoplasm of breast		390<br>4,796	2.3%<br>1.2%	<0.001	0.081
1<br>2	E28.2	Polycystic ovarian syndrome		264<br>3,504	1.5%<br>0.9%	<0.001	0.060
1<br>2	Z80.41	Family history of malignant neoplasm of ovary		50<br>568	0.3%<br>0.1%	<0.001	0.032
1<br>2	D27	Benign neoplasm of ovary		39<br>879	0.2%<br>0.2%	0.871	0.001
**Medication**							
Group			Mean ± SD	Patients	% of Group	P-Value	SMD
1<br>2	A10BA	Biguanides		145<br>9,080	0.8%<br>2.3%	<0.001	0.116
1<br>2	A10BB	Sulfonylureas		2,808<br>31,308	16.3%<br>7.8%	<0.001	0.261
1<br>2	A10BF	Alpha glucosidase inhibitors		60<br>436	0.3%<br>0.1%	<0.001	0.050
1<br>2	A10BG	Thiazolidinediones		974<br>9,195	5.6%<br>2.3%	<0.001	0.172
1<br>2	A10BH	Dipeptidyl peptidase 4 (DPP-4) inhibitors		1,906<br>7,852	11.0%<br>2.0%	<0.001	0.374
1<br>2	A10BK	Sodium-glucose co-transporter 2 (SGLT2) inhibitors		918<br>1,296	5.3%<br>0.3%	<0.001	0.305
1<br>2	A10BX	Other blood glucose lowering drugs, excl. insulins		349<br>1,821	2.0%<br>0.5%	<0.001	0.142

© 2024 Wang L et al. *JAMA Network Open.*

1<br>2	HS200	CONTRACEPTIVES,SYSTEMIC		718<br>10,566	4.2%<br>2.6%	<0.001	0.084

Group 1 (N = 17,197) and group 2 (N = 17,197) characteristics after propensity score matching

Demographics

Group			Mean ± SD	Patients	% of Group	P-Value	SMD
1<br>2	AI	Age at Index	57.8 +/- 12.8<br>57.5 +/- 13.7	17,197<br>17,197	100%<br>100%	0.072	0.019
1<br>2	2106-3	White		11,276<br>11,525	65.6%<br>67.0%	0.005	0.031
1<br>2	1002-5	American Indian or Alaska Native		59<br>51	0.3%<br>0.3%	0.445	0.008
1<br>2	UNK	Unknown Race		1,560<br>1,453	9.1%<br>8.4%	0.041	0.022
1<br>2	2076-8	Native Hawaiian or Other Pacific Islander		80<br>74	0.5%<br>0.4%	0.628	0.005
1<br>2	UN	Unknown Ethnicity		3,140<br>2,967	18.3%<br>17.3%	0.015	0.026
1<br>2	2186-5	Not Hispanic or Latino		12,416<br>12,670	72.2%<br>73.7%	0.002	0.033
1<br>2	2135-2	Hispanic or Latino		1,641<br>1,560	9.5%<br>9.1%	0.133	0.016
1<br>2	2054-5	Black or African American		3,459<br>3,385	20.1%<br>19.7%	0.318	0.011
1<br>2	2028-9	Asian		311<br>297	1.8%<br>1.7%	0.567	0.006

Diagnosis

Group			Mean ± SD	Patients	% of Group	P-Value	SMD
1<br>2	Z55-Z65	Persons with potential health hazards related to socioeconomic and psychosocial circumstances		269<br>232	1.6%<br>1.3%	0.096	0.018
1<br>2	E66	Overweight and obesity		6,398<br>6,326	37.2%<br>36.8%	0.421	0.009
1<br>2	Z68.3	Body mass index [BMI] 30-39, adult		1,331<br>1,290	7.7%<br>7.5%	0.405	0.009

© 2024 Wang L et al. *JAMA Network Open.*

1<br>2	Z68.4	Body mass index [BMI] 40 or greater, adult	1,643<br>1,621	9.6%<br>9.4%	0.686	0.004
1<br>2	Z68.25	Body mass index [BMI] 25.0-25.9, adult	38<br>25	0.2%<br>0.1%	0.101	0.018
1<br>2	Z68.26	Body mass index [BMI] 26.0-26.9, adult	50<br>47	0.3%<br>0.3%	0.760	0.003
1<br>2	Z68.27	Body mass index [BMI] 27.0-27.9, adult	58<br>54	0.3%<br>0.3%	0.705	0.004
1<br>2	Z68.28	Body mass index [BMI] 28.0-28.9, adult	64<br>57	0.4%<br>0.3%	0.524	0.007
1<br>2	Z68.29	Body mass index [BMI] 29.0-29.9, adult	75<br>77	0.4%<br>0.4%	0.871	0.002
1<br>2	F10	Alcohol related disorders	126<br>87	0.7%<br>0.5%	0.007	0.029
1<br>2	Z80	Family history of primary malignant neoplasm	802<br>742	4.7%<br>4.3%	0.118	0.017
1<br>2	Z15.0	Genetic susceptibility to malignant neoplasm	15<br>10	0.1%<br>0.1%	0.317	0.011
1<br>2	Z12	Encounter for screening for malignant neoplasms	4,067<br>3,779	23.6%<br>22.0%	<0.001	0.040
1<br>2	Z85	Personal history of malignant neoplasm	487<br>441	2.8%<br>2.6%	0.126	0.017
1<br>2	Z98.84	Bariatric surgery status	364<br>367	2.1%<br>2.1%	0.911	0.001
1<br>2	F17	Nicotine dependence	1,237<br>1,126	7.2%<br>6.5%	0.018	0.026
1<br>2	E66.0	Obesity due to excess calories	3,409<br>3,333	19.8%<br>19.4%	0.302	0.011
1<br>2	E66.2	Morbid (severe) obesity with alveolar hypoventilation	130<br>120	0.8%<br>0.7%	0.526	0.007
1<br>2	E66.3	Overweight	276<br>268	1.6%<br>1.6%	0.730	0.004

© 2024 Wang L et al. *JAMA Network Open.*

1<br>2	E66.8	Other obesity		116<br>93	0.7%<br>0.5%	0.111	0.017
1<br>2	E66.9	Obesity, unspecified		4,520<br>4,388	26.3%<br>25.5%	0.104	0.018
1<br>2	Z72.0	Tobacco use		330<br>295	1.9%<br>1.7%	0.158	0.015
1<br>2	Z79.890	Hormone replacement therapy		83<br>65	0.5%<br>0.4%	0.138	0.016
1<br>2	Z92.23	Personal history of estrogen therapy		10<br>10	0.1%<br>0.1%	1	<0.001
1<br>2	Z80.3	Family history of malignant neoplasm of breast		381<br>345	2.2%<br>2.0%	0.177	0.015
1<br>2	E28.2	Polycystic ovarian syndrome		263<br>241	1.5%<br>1.4%	0.324	0.011
1<br>2	Z80.41	Family history of malignant neoplasm of ovary		48<br>45	0.3%<br>0.3%	0.755	0.003
1<br>2	D27	Benign neoplasm of ovary		39<br>39	0.2%<br>0.2%	1	<0.001
**Medication**							
Group			Mean ± SD	Patients	% of Group	P-Value	SMD
1<br>2	A10BA	Biguanides		145<br>112	0.8%<br>0.7%	0.039	0.022
1<br>2	A10BB	Sulfonylureas		2,762<br>2,712	16.1%<br>15.8%	0.461	0.008
1<br>2	A10BF	Alpha glucosidase inhibitors		59<br>44	0.3%<br>0.3%	0.139	0.016
1<br>2	A10BG	Thiazolidinediones		946<br>909	5.5%<br>5.3%	0.377	0.010
1<br>2	A10BH	Dipeptidyl peptidase 4 (DPP-4) inhibitors		1,834<br>1,843	10.7%<br>10.7%	0.875	0.002
1<br>2	A10BK	Sodium-glucose co-transporter 2 (SGLT2) inhibitors		847<br>769	4.9%<br>4.5%	0.047	0.021
1<br>2	A10BX	Other blood glucose lowering drugs, excl. insulins		329<br>360	1.9%<br>2.1%	0.233	0.013
1<br>2	HS200	CONTRACEPTIVES,SYSTEMIC		706<br>653	4.1%<br>3.8%	0.142	0.016

© 2024 Wang L et al. *JAMA Network Open.*

© 2024 Wang L et al. *JAMA Network Open*.

eTable 23. Characteristics of the GLP-1RA/no metformin group and metformin/no GLP-1RA group before and after matched for covariates related to pancreatic cancer for the study populations of patients with T2D and no history of any OAC

Group 1 (N = 32,365) and group 2 (N = 856,160) characteristics before propensity score matching

Demographics

Group			Mean ± SD	Patients	% of Group	P-Value	SMD
1<br>2	AI	Age at Index	59.0 +/- 12.4<br>60.1 +/- 14.0	32,365<br>856,160	100%<br>100%	<0.001	0.080
1<br>2	2106-3	White		21,231<br>515,868	65.6%<br>60.3%	<0.001	0.111
1<br>2	1002-5	American Indian or Alaska Native		98<br>3,080	0.3%<br>0.4%	0.092	0.010
1<br>2	UNK	Unknown Race		4,272<br>119,036	13.2%<br>13.9%	<0.001	0.021
1<br>2	F	Female		17,278<br>399,923	53.4%<br>46.7%	<0.001	0.134
1<br>2	2076-8	Native Hawaiian or Other Pacific Islander		172<br>5,884	0.5%<br>0.7%	0.001	0.020
1<br>2	UN	Unknown Gender		1,670<br>19,592	5.2%<br>2.3%	<0.001	0.152
1<br>2	2186-5	Not Hispanic or Latino		22,299<br>528,686	68.9%<br>61.8%	<0.001	0.151
1<br>2	2135-2	Hispanic or Latino		2,660<br>91,303	8.2%<br>10.7%	<0.001	0.084
1<br>2	2054-5	Black or African American		5,107<br>141,606	15.8%<br>16.5%	<0.001	0.021
1<br>2	M	Male		13,417<br>436,645	41.5%<br>51.0%	<0.001	0.192
1<br>2	2028-9	Asian		681<br>35,540	2.1%<br>4.2%	<0.001	0.118

Diagnosis

Group			Mean ± SD	Patients	% of Group	P-Value	SMD
1<br>2	Z55-Z65	Persons with potential health hazards related to socioeconomic and		422<br>10,310	1.3%<br>1.2%	0.107	0.009

© 2024 Wang L et al. *JAMA Network Open.*

		psychosocial circumstances				
1<br>2	E66	Overweight and obesity	11,418<br>149,974	35.3%<br>17.5%	<0.00<br>1	0.411
1<br>2	Z68.3	Body mass index [BMI] 30-39, adult	3,285<br>32,977	10.1%<br>3.9%	<0.00<br>1	0.249
1<br>2	Z68.4	Body mass index [BMI] 40 or greater, adult	3,012<br>23,535	9.3%<br>2.7%	<0.00<br>1	0.278
1<br>2	Z68.25	Body mass index [BMI] 25.0-25.9, adult	119<br>2,293	0.4%<br>0.3%	0.001	0.018
1<br>2	Z68.26	Body mass index [BMI] 26.0-26.9, adult	150<br>2,433	0.5%<br>0.3%	<0.00<br>1	0.029
1<br>2	Z68.27	Body mass index [BMI] 27.0-27.9, adult	209<br>2,942	0.6%<br>0.3%	<0.00<br>1	0.043
1<br>2	Z68.28	Body mass index [BMI] 28.0-28.9, adult	264<br>3,317	0.8%<br>0.4%	<0.00<br>1	0.055
1<br>2	Z68.29	Body mass index [BMI] 29.0-29.9, adult	299<br>3,753	0.9%<br>0.4%	<0.00<br>1	0.059
1<br>2	F10	Alcohol related disorders	460<br>21,150	1.4%<br>2.5%	<0.00<br>1	0.076
1<br>2	Z80	Family history of primary malignant neoplasm	1,222<br>16,133	3.8%<br>1.9%	<0.00<br>1	0.114
1<br>2	Z15.0	Genetic susceptibility to malignant neoplasm	18<br>140	0.1%<br>0.0%	<0.00<br>1	0.021
1<br>2	Z12	Encounter for screening for malignant neoplasms	6,223<br>114,996	19.2%<br>13.4%	<0.00<br>1	0.157
1<br>2	Z85	Personal history of malignant neoplasm	1,111<br>23,231	3.4%<br>2.7%	<0.00<br>1	0.042
1<br>2	Z98.84	Bariatric surgery status	534<br>2,989	1.6%<br>0.3%	<0.00<br>1	0.131
1<br>2	E66.0	Obesity due to excess calories	5,910<br>56,178	18.3%<br>6.6%	<0.00<br>1	0.361

© 2024 Wang L et al. *JAMA Network Open.*

1<br>2	E66.2	Morbid (severe) obesity with alveolar hypoventilation		230<br>2,051	0.7%<br>0.2%	<0.00<br>1	0.069
1<br>2	E66.3	Overweight		535<br>7,794	1.7%<br>0.9%	<0.00<br>1	0.066
1<br>2	E66.8	Other obesity		193<br>1,084	0.6%<br>0.1%	<0.00<br>1	0.078
1<br>2	E66.9	Obesity, unspecified		8,150<br>113,380	25.2%<br>13.2%	<0.00<br>1	0.307
1<br>2	Z72.3	Lack of physical exercise		20<br>251	0.1%<br>0.0%	0.001	0.015
1<br>2	Z72.4	Inappropriate diet and eating habits		43<br>118	0.1%<br>0.0%	<0.00<br>1	0.044
1<br>2	Z85.09	Personal history of malignant neoplasm of other digestive organs		10<br>133	0.0%<br>0.0%	0.032	0.010
1<br>2	B96.8<br>1	Helicobacter pylori [H. pylori] as the cause of diseases classified elsewhere		141<br>3,386	0.4%<br>0.4%	0.259	0.006
1<br>2	Z80.0	Family history of malignant neoplasm of digestive organs		413<br>6,255	1.3%<br>0.7%	<0.00<br>1	0.055
1<br>2	B18	Chronic viral hepatitis		352<br>7,813	1.1%<br>0.9%	0.001	0.018
1<br>2	K86.1	Other chronic pancreatitis		96<br>4,175	0.3%<br>0.5%	<0.00<br>1	0.031
1<br>2	B16	Acute hepatitis B		50<br>1,095	0.2%<br>0.1%	0.191	0.007
1<br>2	B18.1	Chronic viral hepatitis B without delta-agent		58<br>1,158	0.2%<br>0.1%	0.036	0.011
1<br>2	Z72.0	Tobacco use		588<br>11,927	1.8%<br>1.4%	<0.00<br>1	0.034
1<br>2	D13.6	Benign neoplasm of pancreas		10<br>225	0.0%<br>0.0%	0.616	0.003
**Medication**							
Group			Mean ± SD	Patients	% of Group	P-Value	SMD
1<br>2	A10A	INSULINS AND ANALOGUES		14,131<br>159,901	43.7%<br>18.7%	<0.00<br>1	0.560

© 2024 Wang L et al. *JAMA Network Open.*

1<br>2	A10B<br>B	Sulfonylureas		5,464<br>69,045	16.9%<br>8.1%	<0.00<br>1	0.269
1<br>2	A10B<br>F	Alpha glucosidase inhibitors		124<br>876	0.4%<br>0.1%	<0.00<br>1	0.057
1<br>2	A10B<br>G	Thiazolidinediones		1,960<br>20,400	6.1%<br>2.4%	<0.00<br>1	0.183
1<br>2	A10B<br>H	Dipeptidyl peptidase 4 (DPP-4) inhibitors		3,547<br>17,113	11.0%<br>2.0%	<0.00<br>1	0.370
1<br>2	A10B<br>K	Sodium-glucose co-transporter 2 (SGLT2) inhibitors		1,839<br>3,371	5.7%<br>0.4%	<0.00<br>1	0.312
1<br>2	A10B<br>X	Other blood glucose lowering drugs, excl. insulins		672<br>3,839	2.1%<br>0.4%	<0.00<br>1	0.146

Group 1 (N = 32,271) and group 2 (N = 32,271) characteristics after propensity score matching

Demographics

Group			Mean ± SD	Patients	% of Group	P-Value	SMD
1<br>2	AI	Age at Index	59.0 +/- 12.4<br>58.9 +/- 13.1	32,271<br>32,271	100%<br>100%	0.197	0.010
1<br>2	2106-3	White		21,164<br>21,603	65.6%<br>66.9%	<0.00<br>1	0.029
1<br>2	1002-5	American Indian or Alaska Native		98<br>111	0.3%<br>0.3%	0.368	0.007
1<br>2	UNK	Unknown Race		4,259<br>4,244	13.2%<br>13.2%	0.861	0.001
1<br>2	F	Female		17,215<br>17,471	53.3%<br>54.1%	0.043	0.016
1<br>2	2076-8	Native Hawaiian or Other Pacific Islander		172<br>158	0.5%<br>0.5%	0.440	0.006
1<br>2	UN	Unknown Gender		1,658<br>1,746	5.1%<br>5.4%	0.121	0.012
1<br>2	2186-5	Not Hispanic or Latino		22,229<br>22,576	68.9%<br>70.0%	0.003	0.023
1<br>2	2135-2	Hispanic or Latino		2,652<br>2,444	8.2%<br>7.6%	0.002	0.024
1<br>2	2054-5	Black or African American		5,093<br>4,838	15.8%<br>15.0%	0.005	0.022

© 2024 Wang L et al. *JAMA Network Open.*

1<br>2	M	Male		13,398<br>13,054	41.5%<br>40.5%	0.006	0.022
1<br>2	2028-9	Asian		681<br>604	2.1%<br>1.9%	0.030	0.017

Diagnosis

Group			Mean ± SD	Patients	% of Group	P-Value	SMD
1<br>2	Z55-Z65	Persons with potential health hazards related to socioeconomic and psychosocial circumstances		419<br>367	1.3%<br>1.1%	0.062	0.015
1<br>2	E66	Overweight and obesity		11,342<br>11,254	35.1%<br>34.9%	0.468	0.006
1<br>2	Z68.3	Body mass index [BMI] 30-39, adult		3,247<br>3,166	10.1%<br>9.8%	0.287	0.008
1<br>2	Z68.4	Body mass index [BMI] 40 or greater, adult		2,972<br>2,933	9.2%<br>9.1%	0.594	0.004
1<br>2	Z68.25	Body mass index [BMI] 25.0-25.9, adult		119<br>88	0.4%<br>0.3%	0.031	0.017
1<br>2	Z68.26	Body mass index [BMI] 26.0-26.9, adult		150<br>119	0.5%<br>0.4%	0.058	0.015
1<br>2	Z68.27	Body mass index [BMI] 27.0-27.9, adult		208<br>184	0.6%<br>0.6%	0.224	0.010
1<br>2	Z68.28	Body mass index [BMI] 28.0-28.9, adult		262<br>250	0.8%<br>0.8%	0.594	0.004
1<br>2	Z68.29	Body mass index [BMI] 29.0-29.9, adult		298<br>283	0.9%<br>0.9%	0.532	0.005
1<br>2	F10	Alcohol related disorders		458<br>352	1.4%<br>1.1%	<0.001	0.030
1<br>2	Z80	Family history of primary malignant neoplasm		1,207<br>1,071	3.7%<br>3.3%	0.004	0.023

© 2024 Wang L et al. *JAMA Network Open.*

1<br>2	Z15.0	Genetic susceptibility to malignant neoplasm	18<br>15	0.1%<br>0.0%	0.601	0.004
1<br>2	Z12	Encounter for screening for malignant neoplasms	6,176<br>5,576	19.1%<br>17.3%	<0.001	0.048
1<br>2	Z85	Personal history of malignant neoplasm	1,108<br>1,055	3.4%<br>3.3%	0.246	0.009
1<br>2	Z98.84	Bariatric surgery status	520<br>546	1.6%<br>1.7%	0.422	0.006
1<br>2	E66.0	Obesity due to excess calories	5,853<br>5,900	18.1%<br>18.3%	0.632	0.004
1<br>2	E66.2	Morbid (severe) obesity with alveolar hypoventilation	227<br>207	0.7%<br>0.6%	0.335	0.008
1<br>2	E66.3	Overweight	531<br>474	1.6%<br>1.5%	0.070	0.014
1<br>2	E66.8	Other obesity	188<br>194	0.6%<br>0.6%	0.758	0.002
1<br>2	E66.9	Obesity, unspecified	8,094<br>7,929	25.1%<br>24.6%	0.133	0.012
1<br>2	Z72.3	Lack of physical exercise	19<br>21	0.1%<br>0.1%	0.752	0.002
1<br>2	Z72.4	Inappropriate diet and eating habits	41<br>54	0.1%<br>0.2%	0.182	0.011
1<br>2	Z85.09	Personal history of malignant neoplasm of other digestive organs	10<br>10	0.0%<br>0.0%	1	<0.001
1<br>2	B96.81	Helicobacter pylori [H. pylori] as the cause of diseases classified elsewhere	140<br>100	0.4%<br>0.3%	0.010	0.020
1<br>2	Z80.0	Family history of malignant neoplasm of digestive organs	410<br>336	1.3%<br>1.0%	0.006	0.021
1<br>2	B18	Chronic viral hepatitis	352<br>295	1.1%<br>0.9%	0.024	0.018
1<br>2	K86.1	Other chronic pancreatitis	96<br>60	0.3%<br>0.2%	0.004	0.023
1<br>2	B16	Acute hepatitis B	50<br>43	0.2%<br>0.1%	0.468	0.006

© 2024 Wang L et al. *JAMA Network Open.*

1<br>2	B18.1	Chronic viral hepatitis B without delta-agent		58<br>54	0.2%<br>0.2%	0.705	0.003
1<br>2	Z72.0	Tobacco use		584<br>506	1.8%<br>1.6%	0.017	0.019
1<br>2	D13.6	Benign neoplasm of pancreas		10<br>10	0.0%<br>0.0%	1	<0.001
**Medication**							
Group			Mean ± SD	Patients	% of Group	P-Value	SMD
1<br>2	A10A	INSULINS AND ANALOGUES		14,044<br>13,993	43.5%<br>43.4%	0.685	0.003
1<br>2	A10B<br>B	Sulfonylureas		5,422<br>5,287	16.8%<br>16.4%	0.153	0.011
1<br>2	A10B<br>F	Alpha glucosidase inhibitors		121<br>114	0.4%<br>0.4%	0.647	0.004
1<br>2	A10B<br>G	Thiazolidinediones		1,930<br>1,922	6.0%<br>6.0%	0.894	0.001
1<br>2	A10B<br>H	Dipeptidyl peptidase 4 (DPP-4) inhibitors		3,470<br>3,367	10.8%<br>10.4%	0.188	0.010
1<br>2	A10B<br>K	Sodium-glucose co-transporter 2 (SGLT2) inhibitors		1,756<br>1,603	5.4%<br>5.0%	0.007	0.021
1<br>2	A10B<br>X	Other blood glucose lowering drugs, excl. insulins		656<br>637	2.0%<br>2.0%	0.594	0.004

eTable 24. Characteristics of the GLP-1RA/no metformin group and metformin/no GLP-1RA group before and after matched for covariates related to thyroid cancer for the study populations of patients with T2D and no history of any OAC

Group 1 (N = 32,365) and group 2 (N = 856,160) characteristics before propensity score matching							
**Demographics**							
Group			Mean ± SD	Patients	% of Group	P-Value	SMD
1<br>2	AI	Age at Index	59.0 +/- 12.4<br>60.1 +/- 14.0	32,365<br>856,160	100%<br>100%	<0.001	0.080
1<br>2	2106-3	White		21,231<br>515,868	65.6%<br>60.3%	<0.001	0.111
1<br>2	1002-5	American Indian or Alaska Native		98<br>3,080	0.3%<br>0.4%	0.092	0.010
1<br>2	UNK	Unknown Race		4,272<br>119,036	13.2%<br>13.9%	<0.001	0.021
1<br>2	F	Female		17,278<br>399,923	53.4%<br>46.7%	<0.001	0.134
1<br>2	2076-8	Native Hawaiian or Other Pacific Islander		172<br>5,884	0.5%<br>0.7%	0.001	0.020
1<br>2	UN	Unknown Gender		1,670<br>19,592	5.2%<br>2.3%	<0.001	0.152
1<br>2	2186-5	Not Hispanic or Latino		22,299<br>528,686	68.9%<br>61.8%	<0.001	0.151
1<br>2	2135-2	Hispanic or Latino		2,660<br>91,303	8.2%<br>10.7%	<0.001	0.084
1<br>2	2054-5	Black or African American		5,107<br>141,606	15.8%<br>16.5%	<0.001	0.021
1<br>2	M	Male		13,417<br>436,645	41.5%<br>51.0%	<0.001	0.192
1<br>2	2028-9	Asian		681<br>35,540	2.1%<br>4.2%	<0.001	0.118
**Diagnosis**							
Group			Mean ± SD	Patients	% of Group	P-Value	SMD
1<br>2	Z55-Z65	Persons with potential health hazards related to socioeconomic and		422<br>10,310	1.3%<br>1.2%	0.107	0.009

© 2024 Wang L et al. *JAMA Network Open.*

		psychosocial circumstances				
1<br>2	E66	Overweight and obesity	11,418<br>149,974	35.3%<br>17.5%	<0.00<br>1	0.411
1<br>2	Z68.3	Body mass index [BMI] 30-39, adult	3,285<br>32,977	10.1%<br>3.9%	<0.00<br>1	0.249
1<br>2	Z68.4	Body mass index [BMI] 40 or greater, adult	3,012<br>23,535	9.3%<br>2.7%	<0.00<br>1	0.278
1<br>2	Z68.25	Body mass index [BMI] 25.0-25.9, adult	119<br>2,293	0.4%<br>0.3%	0.001	0.018
1<br>2	Z68.26	Body mass index [BMI] 26.0-26.9, adult	150<br>2,433	0.5%<br>0.3%	<0.00<br>1	0.029
1<br>2	Z68.27	Body mass index [BMI] 27.0-27.9, adult	209<br>2,942	0.6%<br>0.3%	<0.00<br>1	0.043
1<br>2	Z68.28	Body mass index [BMI] 28.0-28.9, adult	264<br>3,317	0.8%<br>0.4%	<0.00<br>1	0.055
1<br>2	Z68.29	Body mass index [BMI] 29.0-29.9, adult	299<br>3,753	0.9%<br>0.4%	<0.00<br>1	0.059
1<br>2	Z80	Family history of primary malignant neoplasm	1,222<br>16,133	3.8%<br>1.9%	<0.00<br>1	0.114
1<br>2	Z15.0	Genetic susceptibility to malignant neoplasm	18<br>140	0.1%<br>0.0%	<0.00<br>1	0.021
1<br>2	Z12	Encounter for screening for malignant neoplasms	6,223<br>114,996	19.2%<br>13.4%	<0.00<br>1	0.157
1<br>2	Z85	Personal history of malignant neoplasm	1,111<br>23,231	3.4%<br>2.7%	<0.00<br>1	0.042
1<br>2	Z98.84	Bariatric surgery status	534<br>2,989	1.6%<br>0.3%	<0.00<br>1	0.131
1<br>2	E66.0	Obesity due to excess calories	5,910<br>56,178	18.3%<br>6.6%	<0.00<br>1	0.361
1<br>2	E66.2	Morbid (severe) obesity with alveolar hypoventilation	230<br>2,051	0.7%<br>0.2%	<0.00<br>1	0.069

© 2024 Wang L et al. *JAMA Network Open.*

1<br>2	E66.3	Overweight		535<br>7,794	1.7%<br>0.9%	<0.001	0.066
1<br>2	E66.8	Other obesity		193<br>1,084	0.6%<br>0.1%	<0.001	0.078
1<br>2	E66.9	Obesity, unspecified		8,150<br>113,380	25.2%<br>13.2%	<0.001	0.307
1<br>2	D34	Benign neoplasm of thyroid gland		46<br>715	0.1%<br>0.1%	<0.001	0.017
**Procedure**							
Group			Mean ± SD	Patients	% of Group	P-Value	SMD
1<br>2	1027853	Hereditary neuroendocrine tumor disorders (eg, medullary thyroid carcinoma, parathyroid carcinoma, malignant pheochromocytoma or paraganglioma)		0<br>0	0%<br>0%	--	--
1<br>2	1001	Radiation		114<br>4,550	0.4%<br>0.5%	<0.001	0.027
**Medication**							
Group			Mean ± SD	Patients	% of Group	P-Value	SMD
1<br>2	A10A	INSULINS AND ANALOGUES		14,131<br>159,901	43.7%<br>18.7%	<0.001	0.560
1<br>2	A10BB	Sulfonylureas		5,464<br>69,045	16.9%<br>8.1%	<0.001	0.269
1<br>2	A10BF	Alpha glucosidase inhibitors		124<br>876	0.4%<br>0.1%	<0.001	0.057
1<br>2	A10BG	Thiazolidinediones		1,960<br>20,400	6.1%<br>2.4%	<0.001	0.183
1<br>2	A10BH	Dipeptidyl peptidase 4 (DPP-4) inhibitors		3,547<br>17,113	11.0%<br>2.0%	<0.001	0.370
1<br>2	A10BK	Sodium-glucose co-transporter 2 (SGLT2) inhibitors		1,839<br>3,371	5.7%<br>0.4%	<0.001	0.312
1<br>2	A10BX	Other blood glucose lowering drugs, excl. insulins		672<br>3,839	2.1%<br>0.4%	<0.001	0.146

© 2024 Wang L et al. *JAMA Network Open.*

Group 1 (N = 32,259) and group 2 (N = 32,259) characteristics after propensity score matching							
**Demographics**							
Group			Mean ± SD	Patients	% of Group	P-Value	SMD
1<br>2	AI	Age at Index	59.0 +/- 12.4<br>59.0 +/- 13.0	32,259<br>32,259	100%<br>100%	0.810	0.002
1<br>2	2106-3	White		21,154<br>21,663	65.6%<br>67.2%	<0.001	0.033
1<br>2	1002-5	American Indian or Alaska Native		98<br>88	0.3%<br>0.3%	0.463	0.006
1<br>2	UNK	Unknown Race		4,259<br>4,183	13.2%<br>13.0%	0.375	0.007
1<br>2	F	Female		17,209<br>17,330	53.3%<br>53.7%	0.340	0.008
1<br>2	2076-8	Native Hawaiian or Other Pacific Islander		171<br>140	0.5%<br>0.4%	0.078	0.014
1<br>2	UN	Unknown Gender		1,659<br>1,757	5.1%<br>5.4%	0.085	0.014
1<br>2	2186-5	Not Hispanic or Latino		22,219<br>22,637	68.9%<br>70.2%	<0.001	0.028
1<br>2	2135-2	Hispanic or Latino		2,651<br>2,430	8.2%<br>7.5%	0.001	0.025
1<br>2	2054-5	Black or African American		5,093<br>4,871	15.8%<br>15.1%	0.016	0.019
1<br>2	M	Male		13,391<br>13,172	41.5%<br>40.8%	0.080	0.014
1<br>2	2028-9	Asian		681<br>594	2.1%<br>1.8%	0.014	0.019
**Diagnosis**							
Group			Mean ± SD	Patients	% of Group	P-Value	SMD
1<br>2	Z55-Z65	Persons with potential health hazards related to socioeconomic and psychosocial circumstances		420<br>328	1.3%<br>1.0%	0.001	0.027
1<br>2	E66	Overweight and obesity		11,340<br>11,209	35.2%<br>34.7%	0.279	0.009

© 2024 Wang L et al. *JAMA Network Open.*

1<br>2	Z68.3	Body mass index [BMI] 30-39, adult	3,247<br>3,163	10.1%<br>9.8%	0.269	0.009
1<br>2	Z68.4	Body mass index [BMI] 40 or greater, adult	2,969<br>2,840	9.2%<br>8.8%	0.076	0.014
1<br>2	Z68.25	Body mass index [BMI] 25.0-25.9, adult	118<br>98	0.4%<br>0.3%	0.173	0.011
1<br>2	Z68.26	Body mass index [BMI] 26.0-26.9, adult	149<br>112	0.5%<br>0.3%	0.022	0.018
1<br>2	Z68.27	Body mass index [BMI] 27.0-27.9, adult	207<br>165	0.6%<br>0.5%	0.029	0.017
1<br>2	Z68.28	Body mass index [BMI] 28.0-28.9, adult	261<br>230	0.8%<br>0.7%	0.160	0.011
1<br>2	Z68.29	Body mass index [BMI] 29.0-29.9, adult	296<br>307	0.9%<br>1.0%	0.653	0.004
1<br>2	Z80	Family history of primary malignant neoplasm	1,204<br>1,098	3.7%<br>3.4%	0.024	0.018
1<br>2	Z15.0	Genetic susceptibility to malignant neoplasm	18<br>10	0.1%<br>0.0%	0.130	0.012
1<br>2	Z12	Encounter for screening for malignant neoplasms	6,173<br>5,681	19.1%<br>17.6%	<0.001	0.039
1<br>2	Z85	Personal history of malignant neoplasm	1,106<br>1,049	3.4%<br>3.3%	0.212	0.010
1<br>2	Z98.84	Bariatric surgery status	518<br>504	1.6%<br>1.6%	0.659	0.003
1<br>2	E66.0	Obesity due to excess calories	5,849<br>5,756	18.1%<br>17.8%	0.340	0.008
1<br>2	E66.2	Morbid (severe) obesity with alveolar hypoventilation	228<br>189	0.7%<br>0.6%	0.055	0.015
1<br>2	E66.3	Overweight	532<br>450	1.6%<br>1.4%	0.008	0.021
1<br>2	E66.8	Other obesity	191<br>182	0.6%<br>0.6%	0.640	0.004

1<br>2	E66.9	Obesity, unspecified		8,095<br>7,920	25.1%<br>24.6%	0.111	0.013
1<br>2	D34	Benign neoplasm of thyroid gland		45<br>34	0.1%<br>0.1%	0.216	0.010
**Procedure**							
Group			Mean ± SD	Patients	% of Group	P-Value	SMD
1<br>2	1027853	Hereditary neuroendocrine tumor disorders (eg, medullary thyroid carcinoma, parathyroid carcinoma, malignant pheochromocytoma or paraganglioma)		0<br>0	0%<br>0%	--	--
1<br>2	1001	Radiation		114<br>96	0.4%<br>0.3%	0.213	0.010
**Medication**							
Group			Mean ± SD	Patients	% of Group	P-Value	SMD
1<br>2	A10A	INSULINS AND ANALOGUES		14,033<br>13,951	43.5%<br>43.2%	0.515	0.005
1<br>2	A10BB	Sulfonylureas		5,417<br>5,341	16.8%<br>16.6%	0.422	0.006
1<br>2	A10BF	Alpha glucosidase inhibitors		123<br>113	0.4%<br>0.4%	0.514	0.005
1<br>2	A10BG	Thiazolidinediones		1,926<br>1,876	6.0%<br>5.8%	0.403	0.007
1<br>2	A10BH	Dipeptidyl peptidase 4 (DPP-4) inhibitors		3,469<br>3,506	10.8%<br>10.9%	0.639	0.004
1<br>2	A10BK	Sodium-glucose co-transporter 2 (SGLT2) inhibitors		1,740<br>1,641	5.4%<br>5.1%	0.080	0.014
1<br>2	A10BX	Other blood glucose lowering drugs, excl. insulins		654<br>628	2.0%<br>1.9%	0.463	0.006

© 2024 Wang L et al. *JAMA Network Open.*

eTable 25. Characteristics of the GLP-1RA/no metformin group and metformin/no GLP-1RA group before and after matched for covariates related to meningioma for the study populations of patients with T2D and no history of any OAC

Group 1 (N = 32,365) and group 2 (N = 856,160) characteristics before propensity score matching							
**Demographics**							
Group			Mean ± SD	Patients	% of Group	P-Value	SMD
1<br>2	AI	Age at Index	59.0 +/- 12.4<br>60.1 +/- 14.0	32,365<br>856,160	100%<br>100%	<0.001	0.080
1<br>2	2106-3	White		21,231<br>515,868	65.6%<br>60.3%	<0.001	0.111
1<br>2	1002-5	American Indian or Alaska Native		98<br>3,080	0.3%<br>0.4%	0.092	0.010
1<br>2	UNK	Unknown Race		4,272<br>119,036	13.2%<br>13.9%	<0.001	0.021
1<br>2	F	Female		17,278<br>399,923	53.4%<br>46.7%	<0.001	0.134
1<br>2	2076-8	Native Hawaiian or Other Pacific Islander		172<br>5,884	0.5%<br>0.7%	0.001	0.020
1<br>2	UN	Unknown Gender		1,670<br>19,592	5.2%<br>2.3%	<0.001	0.152
1<br>2	2186-5	Not Hispanic or Latino		22,299<br>528,686	68.9%<br>61.8%	<0.001	0.151
1<br>2	2135-2	Hispanic or Latino		2,660<br>91,303	8.2%<br>10.7%	<0.001	0.084
1<br>2	2054-5	Black or African American		5,107<br>141,606	15.8%<br>16.5%	<0.001	0.021
1<br>2	M	Male		13,417<br>436,645	41.5%<br>51.0%	<0.001	0.192
1<br>2	2028-9	Asian		681<br>35,540	2.1%<br>4.2%	<0.001	0.118
**Diagnosis**							
Group			Mean ± SD	Patients	% of Group	P-Value	SMD
1<br>2	Z55-Z65	Persons with potential health hazards related to socioeconomic and		422<br>10,310	1.3%<br>1.2%	0.107	0.009

© 2024 Wang L et al. *JAMA Network Open.*

		psychosocial circumstances				
1<br>2	E66	Overweight and obesity	11,418<br>149,974	35.3%<br>17.5%	<0.001	0.411
1<br>2	Z68.3	Body mass index [BMI] 30-39, adult	3,285<br>32,977	10.1%<br>3.9%	<0.001	0.249
1<br>2	Z68.4	Body mass index [BMI] 40 or greater, adult	3,012<br>23,535	9.3%<br>2.7%	<0.001	0.278
1<br>2	Z68.25	Body mass index [BMI] 25.0-25.9, adult	119<br>2,293	0.4%<br>0.3%	0.001	0.018
1<br>2	Z68.26	Body mass index [BMI] 26.0-26.9, adult	150<br>2,433	0.5%<br>0.3%	<0.001	0.029
1<br>2	Z68.27	Body mass index [BMI] 27.0-27.9, adult	209<br>2,942	0.6%<br>0.3%	<0.001	0.043
1<br>2	Z68.28	Body mass index [BMI] 28.0-28.9, adult	264<br>3,317	0.8%<br>0.4%	<0.001	0.055
1<br>2	Z68.29	Body mass index [BMI] 29.0-29.9, adult	299<br>3,753	0.9%<br>0.4%	<0.001	0.059
1<br>2	Z80	Family history of primary malignant neoplasm	1,222<br>16,133	3.8%<br>1.9%	<0.001	0.114
1<br>2	Z15.0	Genetic susceptibility to malignant neoplasm	18<br>140	0.1%<br>0.0%	<0.001	0.021
1<br>2	Z12	Encounter for screening for malignant neoplasms	6,223<br>114,996	19.2%<br>13.4%	<0.001	0.157
1<br>2	Z85	Personal history of malignant neoplasm	1,111<br>23,231	3.4%<br>2.7%	<0.001	0.042
1<br>2	Z98.84	Bariatric surgery status	534<br>2,989	1.6%<br>0.3%	<0.001	0.131
1<br>2	E66.0	Obesity due to excess calories	5,910<br>56,178	18.3%<br>6.6%	<0.001	0.361
1<br>2	E66.2	Morbid (severe) obesity with alveolar hypoventilation	230<br>2,051	0.7%<br>0.2%	<0.001	0.069

© 2024 Wang L et al. *JAMA Network Open.*

1<br>2	E66.3	Overweight		535<br>7,794	1.7%<br>0.9%	<0.00<br>1	0.066
1<br>2	E66.8	Other obesity		193<br>1,084	0.6%<br>0.1%	<0.00<br>1	0.078
1<br>2	E66.9	Obesity, unspecified		8,150<br>113,380	25.2%<br>13.2%	<0.00<br>1	0.307
1<br>2	Z79.89<br>0	Hormone replacement therapy		94<br>1,381	0.3%<br>0.2%	<0.00<br>1	0.027
1<br>2	Z92.23	Personal history of estrogen therapy		10<br>117	0.0%<br>0.0%	0.011	0.012
1<br>2	D32	Benign neoplasm of meninges		84<br>2,087	0.3%<br>0.2%	0.573	0.003
1<br>2	S00-<br>S09	Injuries to the head		1,546<br>41,909	4.8%<br>4.9%	0.333	0.006
1<br>2	Z80.3	Family history of malignant neoplasm of breast		438<br>5,375	1.4%<br>0.6%	<0.00<br>1	0.073

Procedure

Group			Mean ± SD	Patients	% of Group	P-Value	SMD
1<br>2	1001	Radiation		114<br>4,550	0.4%<br>0.5%	<0.00<br>1	0.027

Medication

Group			Mean ± SD	Patients	% of Group	P-Value	SMD
1<br>2	A10A	INSULINS AND ANALOGUES		14,131<br>159,901	43.7%<br>18.7%	<0.00<br>1	0.560
1<br>2	A10B<br>B	Sulfonylureas		5,464<br>69,045	16.9%<br>8.1%	<0.00<br>1	0.269
1<br>2	A10B<br>F	Alpha glucosidase inhibitors		124<br>876	0.4%<br>0.1%	<0.00<br>1	0.057
1<br>2	A10B<br>G	Thiazolidinediones		1,960<br>20,400	6.1%<br>2.4%	<0.00<br>1	0.183
1<br>2	A10B<br>H	Dipeptidyl peptidase 4 (DPP-4) inhibitors		3,547<br>17,113	11.0%<br>2.0%	<0.00<br>1	0.370
1<br>2	A10B<br>K	Sodium-glucose co-transporter 2 (SGLT2) inhibitors		1,839<br>3,371	5.7%<br>0.4%	<0.00<br>1	0.312
1<br>2	A10B<br>X	Other blood glucose lowering drugs, excl. insulins		672<br>3,839	2.1%<br>0.4%	<0.00<br>1	0.146

1<br>2	HS200	CONTRACEPTIVES,SYSTEMIC		1,069<br>17,029	3.3%<br>2.0%	<0.001	0.082

Group 1 (N = 32,263) and group 2 (N = 32,263) characteristics after propensity score matching

Demographics

Group			Mean ± SD	Patients	% of Group	P-Value	SMD
1<br>2	AI	Age at Index	59.0 +/- 12.4<br>58.9 +/- 13.2	32,263<br>32,263	100%<br>100%	0.166	0.011
1<br>2	2106-3	White		21,158<br>21,735	65.6%<br>67.4%	<0.001	0.038
1<br>2	1002-5	American Indian or Alaska Native		98<br>100	0.3%<br>0.3%	0.887	0.001
1<br>2	UNK	Unknown Race		4,255<br>4,150	13.2%<br>12.9%	0.219	0.010
1<br>2	F	Female		17,216<br>17,415	53.4%<br>54.0%	0.116	0.012
1<br>2	2076-8	Native Hawaiian or Other Pacific Islander		172<br>152	0.5%<br>0.5%	0.265	0.009
1<br>2	UN	Unknown Gender		1,655<br>1,734	5.1%<br>5.4%	0.163	0.011
1<br>2	2186-5	Not Hispanic or Latino		22,224<br>22,513	68.9%<br>69.8%	0.014	0.019
1<br>2	2135-2	Hispanic or Latino		2,653<br>2,442	8.2%<br>7.6%	0.002	0.024
1<br>2	2054-5	Black or African American		5,095<br>4,800	15.8%<br>14.9%	0.001	0.025
1<br>2	M	Male		13,392<br>13,114	41.5%<br>40.6%	0.026	0.018
1<br>2	2028-9	Asian		681<br>576	2.1%<br>1.8%	0.003	0.024

Diagnosis

Group			Mean ± SD	Patients	% of Group	P-Value	SMD
1<br>2	Z55-Z65	Persons with potential health hazards related to socioeconomic and psychosocial circumstances		420<br>372	1.3%<br>1.2%	0.086	0.014

© 2024 Wang L et al. *JAMA Network Open.*

1<br>2	E66	Overweight and obesity	11,346<br>11,303	35.2%<br>35.0%	0.723	0.003
1<br>2	Z68.3	Body mass index [BMI] 30-39, adult	3,247<br>3,143	10.1%<br>9.7%	0.170	0.011
1<br>2	Z68.4	Body mass index [BMI] 40 or greater, adult	2,968<br>2,925	9.2%<br>9.1%	0.557	0.005
1<br>2	Z68.25	Body mass index [BMI] 25.0-25.9, adult	118<br>96	0.4%<br>0.3%	0.132	0.012
1<br>2	Z68.26	Body mass index [BMI] 26.0-26.9, adult	149<br>104	0.5%<br>0.3%	0.005	0.022
1<br>2	Z68.27	Body mass index [BMI] 27.0-27.9, adult	208<br>163	0.6%<br>0.5%	0.019	0.018
1<br>2	Z68.28	Body mass index [BMI] 28.0-28.9, adult	261<br>220	0.8%<br>0.7%	0.061	0.015
1<br>2	Z68.29	Body mass index [BMI] 29.0-29.9, adult	297<br>263	0.9%<br>0.8%	0.149	0.011
1<br>2	Z80	Family history of primary malignant neoplasm	1,206<br>1,103	3.7%<br>3.4%	0.029	0.017
1<br>2	Z15.0	Genetic susceptibility to malignant neoplasm	18<br>11	0.1%<br>0.0%	0.194	0.010
1<br>2	Z12	Encounter for screening for malignant neoplasms	6,176<br>5,548	19.1%<br>17.2%	<0.001	0.050
1<br>2	Z85	Personal history of malignant neoplasm	1,105<br>1,057	3.4%<br>3.3%	0.294	0.008
1<br>2	Z98.84	Bariatric surgery status	518<br>560	1.6%<br>1.7%	0.197	0.010
1<br>2	E66.0	Obesity due to excess calories	5,856<br>5,952	18.2%<br>18.4%	0.328	0.008
1<br>2	E66.2	Morbid (severe) obesity with alveolar hypoventilation	228<br>206	0.7%<br>0.6%	0.289	0.008
1<br>2	E66.3	Overweight	532<br>455	1.6%<br>1.4%	0.014	0.019

© 2024 Wang L et al. *JAMA Network Open.*

1<br>2	E66.8	Other obesity		191<br>177	0.6%<br>0.5%	0.464	0.006
1<br>2	E66.9	Obesity, unspecified		8,098<br>7,991	25.1%<br>24.8%	0.330	0.008
1<br>2	Z79.890	Hormone replacement therapy		93<br>70	0.3%<br>0.2%	0.071	0.014
1<br>2	Z92.23	Personal history of estrogen therapy		10<br>10	0.0%<br>0.0%	1	<0.001
1<br>2	D32	Benign neoplasm of meninges		84<br>72	0.3%<br>0.2%	0.336	0.008
1<br>2	S00-S09	Injuries to the head		1,533<br>1,329	4.8%<br>4.1%	<0.001	0.031
1<br>2	Z80.3	Family history of malignant neoplasm of breast		431<br>402	1.3%<br>1.2%	0.312	0.008
**Procedure**							
Group			Mean ± SD	Patients	% of Group	P-Value	SMD
1<br>2	1001	Radiation		114<br>88	0.4%<br>0.3%	0.067	0.014
**Medication**							
Group			Mean ± SD	Patients	% of Group	P-Value	SMD
1<br>2	A10A	INSULINS AND ANALOGUES		14,036<br>13,882	43.5%<br>43.0%	0.221	0.010
1<br>2	A10BB	Sulfonylureas		5,418<br>5,268	16.8%<br>16.3%	0.112	0.013
1<br>2	A10BF	Alpha glucosidase inhibitors		122<br>135	0.4%<br>0.4%	0.416	0.006
1<br>2	A10BG	Thiazolidinediones		1,933<br>1,861	6.0%<br>5.8%	0.228	0.009
1<br>2	A10BH	Dipeptidyl peptidase 4 (DPP-4) inhibitors		3,468<br>3,437	10.7%<br>10.7%	0.693	0.003
1<br>2	A10BK	Sodium-glucose co-transporter 2 (SGLT2) inhibitors		1,745<br>1,616	5.4%<br>5.0%	0.022	0.018
1<br>2	A10BX	Other blood glucose lowering drugs, excl. insulins		657<br>615	2.0%<br>1.9%	0.234	0.009
1<br>2	HS200	CONTRACEPTIVES,SYSTEMIC		1,058<br>923	3.3%<br>2.9%	0.002	0.024

© 2024 Wang L et al. *JAMA Network Open.*

eTable 26. Characteristics of the GLP-1RA/no metformin group and metformin/no GLP-1RA group before and after matched for covariates related to multiple myeloma for the study populations of patients with T2D and no history of any OAC

Group 1 (N = 32,365) and group 2 (N = 856,160) characteristics before propensity score matching

Demographics

Group			Mean ± SD	Patients	% of Group	P-Value	SMD
1<br>2	AI	Age at Index	59.0 +/- 12.4<br>60.1 +/- 14.0	32,365<br>856,160	100%<br>100%	<0.001	0.080
1<br>2	2106-3	White		21,231<br>515,868	65.6%<br>60.3%	<0.001	0.111
1<br>2	1002-5	American Indian or Alaska Native		98<br>3,080	0.3%<br>0.4%	0.092	0.010
1<br>2	UNK	Unknown Race		4,272<br>119,036	13.2%<br>13.9%	<0.001	0.021
1<br>2	F	Female		17,278<br>399,923	53.4%<br>46.7%	<0.001	0.134
1<br>2	2076-8	Native Hawaiian or Other Pacific Islander		172<br>5,884	0.5%<br>0.7%	0.001	0.020
1<br>2	UN	Unknown Gender		1,670<br>19,592	5.2%<br>2.3%	<0.001	0.152
1<br>2	2186-5	Not Hispanic or Latino		22,299<br>528,686	68.9%<br>61.8%	<0.001	0.151
1<br>2	2135-2	Hispanic or Latino		2,660<br>91,303	8.2%<br>10.7%	<0.001	0.084
1<br>2	2054-5	Black or African American		5,107<br>141,606	15.8%<br>16.5%	<0.001	0.021
1<br>2	M	Male		13,417<br>436,645	41.5%<br>51.0%	<0.001	0.192
1<br>2	2028-9	Asian		681<br>35,540	2.1%<br>4.2%	<0.001	0.118

Diagnosis

Group			Mean ± SD	Patients	% of Group	P-Value	SMD
1<br>2	Z55-Z65	Persons with potential health hazards related to		422<br>10,310	1.3%<br>1.2%	0.107	0.009

© 2024 Wang L et al. *JAMA Network Open.*

		socioeconomic and psychosocial circumstances				
1<br>2	E66	Overweight and obesity	11,418<br>149,974	35.3%<br>17.5%	<0.001	0.411
1<br>2	Z68.3	Body mass index [BMI] 30-39, adult	3,285<br>32,977	10.1%<br>3.9%	<0.001	0.249
1<br>2	Z68.4	Body mass index [BMI] 40 or greater, adult	3,012<br>23,535	9.3%<br>2.7%	<0.001	0.278
1<br>2	Z68.25	Body mass index [BMI] 25.0-25.9, adult	119<br>2,293	0.4%<br>0.3%	0.001	0.018
1<br>2	Z68.26	Body mass index [BMI] 26.0-26.9, adult	150<br>2,433	0.5%<br>0.3%	<0.001	0.029
1<br>2	Z68.27	Body mass index [BMI] 27.0-27.9, adult	209<br>2,942	0.6%<br>0.3%	<0.001	0.043
1<br>2	Z68.28	Body mass index [BMI] 28.0-28.9, adult	264<br>3,317	0.8%<br>0.4%	<0.001	0.055
1<br>2	Z68.29	Body mass index [BMI] 29.0-29.9, adult	299<br>3,753	0.9%<br>0.4%	<0.001	0.059
1<br>2	Z80	Family history of primary malignant neoplasm	1,222<br>16,133	3.8%<br>1.9%	<0.001	0.114
1<br>2	Z15.0	Genetic susceptibility to malignant neoplasm	18<br>140	0.1%<br>0.0%	<0.001	0.021
1<br>2	Z12	Encounter for screening for malignant neoplasms	6,223<br>114,996	19.2%<br>13.4%	<0.001	0.157
1<br>2	Z85	Personal history of malignant neoplasm	1,111<br>23,231	3.4%<br>2.7%	<0.001	0.042
1<br>2	Z98.84	Bariatric surgery status	534<br>2,989	1.6%<br>0.3%	<0.001	0.131
1<br>2	E66.0	Obesity due to excess calories	5,910<br>56,178	18.3%<br>6.6%	<0.001	0.361

© 2024 Wang L et al. *JAMA Network Open.*

1<br>2	E66.2	Morbid (severe) obesity with alveolar hypoventilation		230<br>2,051	0.7%<br>0.2%	<0.001	0.069
1<br>2	E66.3	Overweight		535<br>7,794	1.7%<br>0.9%	<0.001	0.066
1<br>2	E66.8	Other obesity		193<br>1,084	0.6%<br>0.1%	<0.001	0.078
1<br>2	E66.9	Obesity, unspecified		8,150<br>113,380	25.2%<br>13.2%	<0.001	0.307
1<br>2	Z80.7	Family history of other malignant neoplasms of lymphoid, hematopoietic and related tissues		15<br>201	0.0%<br>0.0%	0.010	0.012
1<br>2	D47.2	Monoclonal gammopathy		111<br>1,346	0.3%<br>0.2%	<0.001	0.037
1<br>2	C90.3	Solitary plasmacytoma		10<br>30	0.0%<br>0.0%	<0.001	0.021
1<br>2	D45	Polycythemia vera		67<br>1,167	0.2%<br>0.1%	0.001	0.017
1<br>2	D46	Myelodysplastic syndromes		31<br>811	0.1%<br>0.1%	0.952	<0.001
1<br>2	D47	Other neoplasms of uncertain behavior of lymphoid, hematopoietic and related tissue		234<br>4,409	0.7%<br>0.5%	<0.001	0.027
**Medication**							
Group			Mean ± SD	Patients	% of Group	P-Value	SMD
1<br>2	A10A	INSULINS AND ANALOGUES		14,131<br>159,901	43.7%<br>18.7%	<0.001	0.560
1<br>2	A10BB	Sulfonylureas		5,464<br>69,045	16.9%<br>8.1%	<0.001	0.269
1<br>2	A10BF	Alpha glucosidase inhibitors		124<br>876	0.4%<br>0.1%	<0.001	0.057
1<br>2	A10BG	Thiazolidinediones		1,960<br>20,400	6.1%<br>2.4%	<0.001	0.183
1<br>2	A10BH	Dipeptidyl peptidase 4 (DPP-4) inhibitors		3,547<br>17,113	11.0%<br>2.0%	<0.001	0.370

© 2024 Wang L et al. *JAMA Network Open.*

1<br>2	A10B<br>K	Sodium-glucose co-transporter 2 (SGLT2) inhibitors		1,839<br>3,371	5.7%<br>0.4%	<0.00<br>1	0.312
1<br>2	A10B<br>X	Other blood glucose lowering drugs, excl. insulins		672<br>3,839	2.1%<br>0.4%	<0.00<br>1	0.146

Group 1 (N = 32,256) and group 2 (N = 32,256) characteristics after propensity score matching

Demographics

Group			Mean ± SD	Patients	% of Group	P-Value	SMD
1<br>2	AI	Age at Index	59.0 +/- 12.4<br>58.9 +/- 13.1	32,256<br>32,256	100%<br>100%	0.248	0.009
1<br>2	2106-3	White		21,155<br>21,672	65.6%<br>67.2%	<0.00<br>1	0.034
1<br>2	1002-5	American Indian or Alaska Native		98<br>98	0.3%<br>0.3%	1	<0.001
1<br>2	UNK	Unknown Race		4,252<br>4,090	13.2%<br>12.7%	0.057	0.015
1<br>2	F	Female		17,214<br>17,371	53.4%<br>53.9%	0.215	0.010
1<br>2	2076-8	Native Hawaiian or Other Pacific Islander		172<br>143	0.5%<br>0.4%	0.101	0.013
1<br>2	UN	Unknown Gender		1,652<br>1,674	5.1%<br>5.2%	0.695	0.003
1<br>2	2186-5	Not Hispanic or Latino		22,221<br>22,596	68.9%<br>70.1%	0.001	0.025
1<br>2	2135-2	Hispanic or Latino		2,652<br>2,460	8.2%<br>7.6%	0.005	0.022
1<br>2	2054-5	Black or African American		5,095<br>4,873	15.8%<br>15.1%	0.016	0.019
1<br>2	M	Male		13,390<br>13,211	41.5%<br>41.0%	0.152	0.011
1<br>2	2028-9	Asian		681<br>636	2.1%<br>2.0%	0.210	0.010

Diagnosis

Group			Mean ± SD	Patients	% of Group	P-Value	SMD
1<br>2	Z55-<br>Z65	Persons with potential health		419<br>333	1.3%<br>1.0%	0.002	0.025

© 2024 Wang L et al. *JAMA Network Open.*

		hazards related to socioeconomic and psychosocial circumstances				
1<br>2	E66	Overweight and obesity	11,336<br>11,284	35.1%<br>35.0%	0.668	0.003
1<br>2	Z68.3	Body mass index [BMI] 30-39, adult	3,244<br>3,159	10.1%<br>9.8%	0.263	0.009
1<br>2	Z68.4	Body mass index [BMI] 40 or greater, adult	2,968<br>2,894	9.2%<br>9.0%	0.311	0.008
1<br>2	Z68.25	Body mass index [BMI] 25.0-25.9, adult	119<br>85	0.4%<br>0.3%	0.017	0.019
1<br>2	Z68.26	Body mass index [BMI] 26.0-26.9, adult	150<br>101	0.5%<br>0.3%	0.002	0.024
1<br>2	Z68.27	Body mass index [BMI] 27.0-27.9, adult	208<br>162	0.6%<br>0.5%	0.016	0.019
1<br>2	Z68.28	Body mass index [BMI] 28.0-28.9, adult	262<br>218	0.8%<br>0.7%	0.044	0.016
1<br>2	Z68.29	Body mass index [BMI] 29.0-29.9, adult	297<br>269	0.9%<br>0.8%	0.237	0.009
1<br>2	Z80	Family history of primary malignant neoplasm	1,203<br>1,121	3.7%<br>3.5%	0.083	0.014
1<br>2	Z15.0	Genetic susceptibility to malignant neoplasm	18<br>11	0.1%<br>0.0%	0.194	0.010
1<br>2	Z12	Encounter for screening for malignant neoplasms	6,176<br>5,635	19.1%<br>17.5%	<0.001	0.043
1<br>2	Z85	Personal history of malignant neoplasm	1,107<br>1,014	3.4%<br>3.1%	0.040	0.016
1<br>2	Z98.84	Bariatric surgery status	517<br>541	1.6%<br>1.7%	0.457	0.006
1<br>2	E66.0	Obesity due to excess calories	5,848<br>5,885	18.1%<br>18.2%	0.706	0.003

© 2024 Wang L et al. *JAMA Network Open.*

1<br>2	E66.2	Morbid (severe) obesity with alveolar hypoventilation		227<br>213	0.7%<br>0.7%	0.503	0.005
1<br>2	E66.3	Overweight		531<br>466	1.6%<br>1.4%	0.038	0.016
1<br>2	E66.8	Other obesity		190<br>159	0.6%<br>0.5%	0.096	0.013
1<br>2	E66.9	Obesity, unspecified		8,093<br>8,035	25.1%<br>24.9%	0.598	0.004
1<br>2	Z80.7	Family history of other malignant neoplasms of lymphoid, hematopoietic and related tissues		15<br>16	0.0%<br>0.0%	0.857	0.001
1<br>2	D47.2	Monoclonal gammopathy		110<br>102	0.3%<br>0.3%	0.582	0.004
1<br>2	C90.3	Solitary plasmacytoma		10<br>10	0.0%<br>0.0%	1	<0.001
1<br>2	D45	Polycythemia vera		64<br>54	0.2%<br>0.2%	0.357	0.007
1<br>2	D46	Myelodysplastic syndromes		31<br>19	0.1%<br>0.1%	0.090	0.013
1<br>2	D47	Other neoplasms of uncertain behavior of lymphoid, hematopoietic and related tissue		233<br>201	0.7%<br>0.6%	0.123	0.012
**Medication**							
Group			Mean ± SD	Patients	% of Group	P-Value	SMD
1<br>2	A10A	INSULINS AND ANALOGUES		14,032<br>13,985	43.5%<br>43.4%	0.709	0.003
1<br>2	A10B<br>B	Sulfonylureas		5,412<br>5,332	16.8%<br>16.5%	0.398	0.007
1<br>2	A10B<br>F	Alpha glucosidase inhibitors		121<br>121	0.4%<br>0.4%	1	<0.001
1<br>2	A10B<br>G	Thiazolidinediones		1,930<br>1,898	6.0%<br>5.9%	0.594	0.004
1<br>2	A10B<br>H	Dipeptidyl peptidase 4 (DPP-4) inhibitors		3,465<br>3,454	10.7%<br>10.7%	0.889	0.001

© 2024 Wang L et al. *JAMA Network Open.*

1<br>2	A10B<br>K	Sodium-glucose co-transporter 2 (SGLT2) inhibitors	1,742<br>1,632	5.4%<br>5.1%	0.052	0.015
1<br>2	A10B<br>X	Other blood glucose lowering drugs, excl. insulins	654<br>650	2.0%<br>2.0%	0.911	0.001

© 2024 Wang L et al. *JAMA Network Open.*
