# Supplementary material for: Glucagon-Like Peptide 1 Receptor Agonists and 13 Obesity-Associated Cancers in Patients With Type 2 Diabetes
Source: JAMA Netw Open. 2024 Jul 5;7(7):e2421305. doi: 10.1001/jamanetworkopen.2024.21305 (PMC11227080; doi:10.1001/jamanetworkopen.2024.21305)
Supplement: Supplement 2. — Data Sharing Statement [file jamanetwopen-e2421305-s002.pdf]

Data Sharing Statement

Wang. Glucagon-Like Peptide 1 Receptor Agonists and 13 Obesity-Associated Cancers in Patients With Type 2 Diabetes. *JAMA Netw Open*. Published July 11, 2024. doi:10.1001/jamanetworkopen.2024.21305

Data
Data available: No

Additional Information
Explanation for why data not available: This study used population-level aggregate and de-identified data collected by the TriNetX Platform and are available from TriNetX, LLC (https://trinetx.com/) but third-party restrictions apply to the availability of these data. The data were used under license for this study with restrictions that do not allow for the data to be redistributed or made publicly available. To gain access to the data, a request can be made to TriNetX (join@trinetx.com), but costs might be incurred, and a data-sharing agreement would be necessary. Data specific to this study including diagnosis codes and cohort characteristics in aggregated format are included in the manuscript as tables, figures, and supplementary files.
